# Effectiveness of a Cloud-Based Telepathology System in China: Large-Sample Observational Study

**DOI:** 10.2196/23799

**Published:** 2021-07-29

**Authors:** Xianying He, Linlin Wang, Li Wang, Jinghong Gao, Fangfang Cui, Qianqian Ma, Wenjie Zhang, Lin Wang, Yunkai Zhai, Jie Zhao

**Affiliations:** 1 National Telemedicine Center of China The First Affiliated Hospital of Zhengzhou University Zhengzhou China; 2 Department of Pathology The First Affiliated Hospital of Zhengzhou University Zhengzhou China; 3 National Engineering Laboratory for Internet Medical Systems and Applications Zhengzhou China; 4 School of Management Engineering Zhengzhou University Zhengzhou China

**Keywords:** telepathology, cloud-based system, whole-slide imaging, turnaround time, diagnostic accuracy, economic benefits

## Abstract

**Background:**

Whole-slide imaging allows the entire slide to be viewed in a manner that simulates microscopy; therefore, it is widely used in telepathology. However, managing the large digital files needed for whole-slide imaging is difficult. To solve this problem, we set up the Chinese National Cloud-Based Telepathology System (CNCTPS). CNCTPS has been running for more than 4 years and has accumulated a large amount of data.

**Objective:**

The main purpose of this study was to comprehensively evaluate the effectiveness of the CNCTPS based on a large sample. The evaluation indicators included service volume, turnaround time, diagnosis accuracy, and economic benefits.

**Methods:**

Details of 23,167 cases submitted to the CNCTPS from January 2016 to December 2019 were collected to analyze the service volume, turnaround time, and economic benefits. A total of 564 patients who visited the First Affiliated Hospital of Zhengzhou University and obtained final diagnoses were followed up to analyze the diagnostic accuracy of the CNCTPS.

**Results:**

From 2016 to 2019, the service volume of the CNCTPS increased from 2335 to 9240, and the number of participating hospitals increased from 60 to 74. Consultation requests from county-level hospitals accounted for 86.57% (20,287/23,167). A total of 17,495 of 23,167 cases (75.52%) were confirmed, including 12,088 benign lesions, 5217 malignant lesions, and 190 borderline lesions. Of the cases, 3.85% (893/23,167) failed to be diagnosed for reasons such as poor slice quality and incomplete sampling. The median turnaround time was 16.93 hours and was shortened yearly (between 2018 and 2019: adjusted *P*=.01; other groups: adjusted *P*<.001); 82.88% cases were diagnosed in 48 hours. There was a discrepancy between the diagnosis and final diagnosis for 11 cases, including 4 false-positive cases and 7 false-negative cases. The sensitivity and specificity were 97.66% and 98.49%, respectively. The diagnostic accuracy of the system was 98.05%, with no statistical difference from the final diagnosis in the hospital (*P*=.55). By using this system, a total of US $300,000 was saved for patients every year.

**Conclusions:**

The novel cloud-based telepathology system has the potential to relieve the shortage of pathologists in primary hospitals. It can also simultaneously reduce medical costs for patients in China. It should, therefore, be further promoted to enhance the efficiency, quantity, and quality of telepathology diagnoses.

## Introduction

Pathology diagnoses have been widely recognized as a gold standard for confirming diseases [1]. A precise and timely diagnosis is an indispensable precondition for further therapies [2]. However, there is a critical shortage and misdistribution of senior pathologists in resource-limited countries; China faces this challenge. According to statistics from the National Ministry of Health, there are 9841 licensed pathologists in China, but nearly 70% work in tertiary hospitals located in large cities [3]. Pathologists, especially senior and professional ones, are urgently needed in rural and remote areas [4-6]. To obtain a confirmed diagnosis and key guidance for subsequent therapies, undiagnosed pathology sections in county-level hospitals are usually mailed or personally transported to senior pathologists in tertiary hospitals. The procedure is complicated and costly. Furthermore, the valuable pathology sections are also at risk of being destroyed or lost.

Telepathology is a powerful tool that can be used to address this challenge by transmitting pathology images through telecommunication [7-9]. The first use of telepathology can be traced back to the 1960s in the United States, in which real-time black-and-white images were sent for interpretation. After a half a century of development, many uses of telepathology have been developed, with powerful features that can promptly transmit static, dynamic, and whole-slide images. The whole-slide imaging system is the most advanced means to view scanned and digitized slides in their entirety, with high-resolution digital images and superior zoom capability [10-12]. Whole-slide imaging has therefore been considered to be an ideal method for telepathology [13]. Despite considerable advancements, whole-slide imaging has several drawbacks [14], such as the need for large local storage space, network bandwidth constraints, cumbersome operation, occupied computing resources and large idle space, insufficient utilization rate, and difficulty in managing large digital files [15], which limits the application of whole-slide imaging.

To compensate for the shortcomings of whole-slide imaging, we established a Chinese National Cloud-Based Telepathology system (CNCTPS) based on an existing, mature telemedicine system of the National Telemedicine Center of China, with dual video and data drives, which solved the difficulty of telemedicine data interaction [16]. The CNCTPS was equipped with a deeply optimized storage model and analytical algorithm, which solved the problems of archiving classification and integration of large amounts of pathology data. This novel system can facilitate the prompt extraction and utilization of pathology data by doctors. The CNCTPS was deployed in December 2015; 83 hospitals were connected in total, making it the largest remote pathology network in China.

Previous studies have mainly focused on the construction and optimization of telepathology systems or analysis of the effect of system use, with a limited sample [17-22], and to date, there are no unified criteria to evaluate a telepathology system. Perron et al [23] evaluated diagnostic concordance and the turnaround time of a telepathology system. Chong et al [24] showed that telepathology shortened turnaround time and provided significant financial savings. Similarly, Zhou et al [6] reported service volume, turnaround time, and the concordance rate of a telepathology consultation service. However, these studies were mainly focused on one or some small and isolated aspects and did not comprehensively evaluate the service effect of the telepathology system. Thus, the aim of this study was to comprehensively evaluate the CNCTPS by evaluating 4 aspects—service volume, turnaround time, diagnosis accuracy, and economic benefits—which we chose after reviewing the literature on telepathology systems evaluation.

## Methods

### The Cloud-Based Telepathology System

#### Digitization of Pathology Sections

Participating hospitals were equipped with digital slide scanners and matched computer workstations (KF-PRO-005, Konfoong Biotech International Co Ltd), for converting traditional glass slices into whole-slide imaging. Whole-slide imaging of a slide could be completed within 40 seconds under a ×20 objective (0.47 µm/pixel) and within 100 seconds under a ×40 objective (0.5 µm/pixel). Scanning control software (K-Scanner 1.6.0.14, Konfoong Biotech International Co Ltd) and image browsing and management software (K-Viewer 1.5.3.1, Konfoong Biotech International Co Ltd) were used to control scanning and viewing in whole-slide imaging.

#### Data Storage and Transport

**Figure 1:**
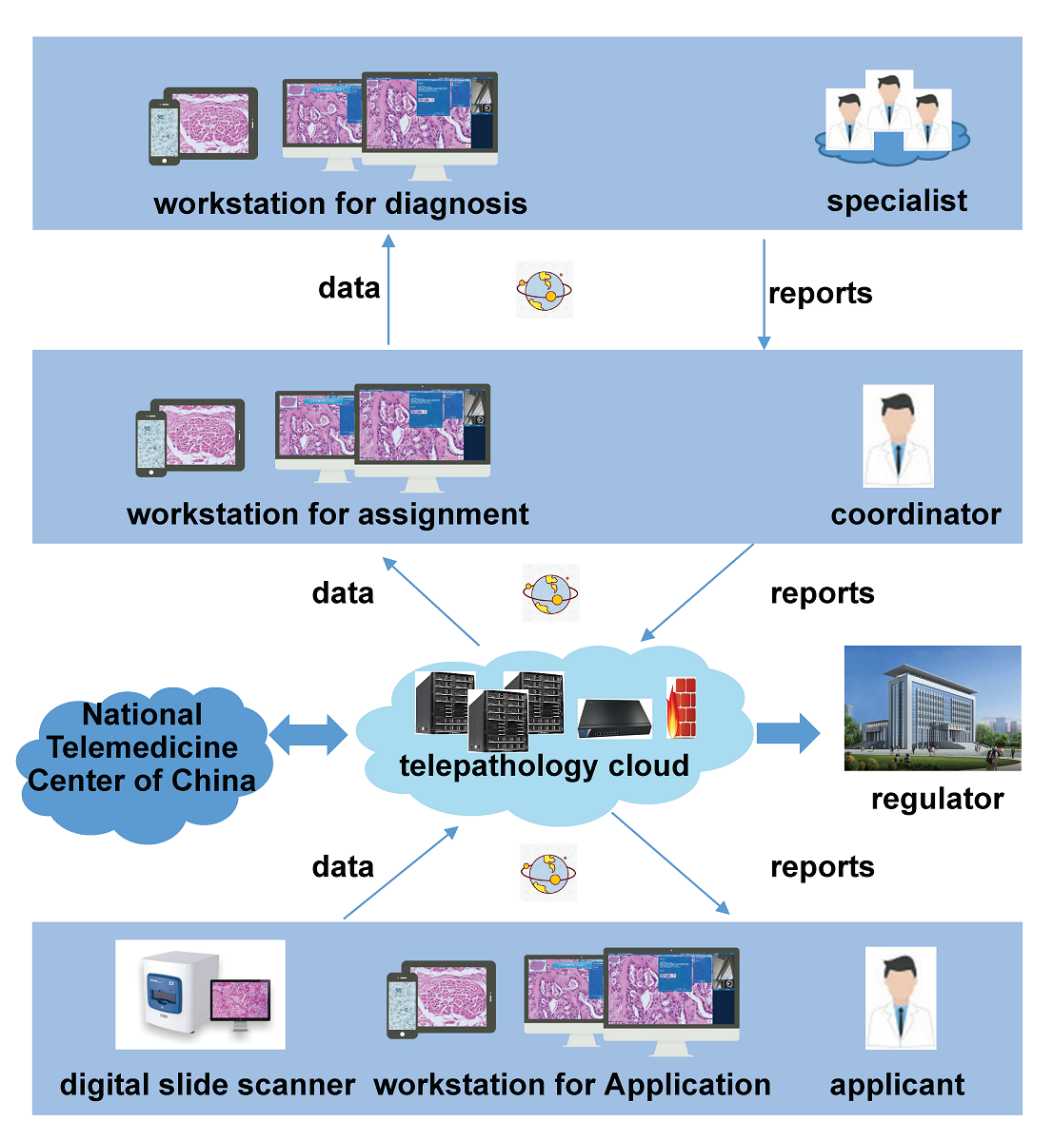
Telepathology data storage and transmission.

Whole-slide imaging and all other telepathology data were stored in dedicated servers located at the National Telemedicine Center of China to ensure the safety and speed of data storage, as well as the efficiency of data access by users. The overall design was based on a cloud-computing infrastructure service system, which was characterized by elastic expansion, high availability, and high stability. The system is equipped with wide-area and multilayer architecture, including access, application service, and data center layers (Figure 1). A private network with a bandwidth of up to 20 MB was used for data transmission.

#### Telepathology Management

A web-based telepathology consultation system and mobile app were developed. Each has different functions for applicants, coordinators, and specialists. The web version was embedded in the telemedicine collaborative service platform of the National Telemedicine Center of China [25]; the app was independently developed and adapted for Android and iOS mobile phones and tablets (Multimedia Appendix 1).

#### Telepathology Consultation

**Figure 2:**
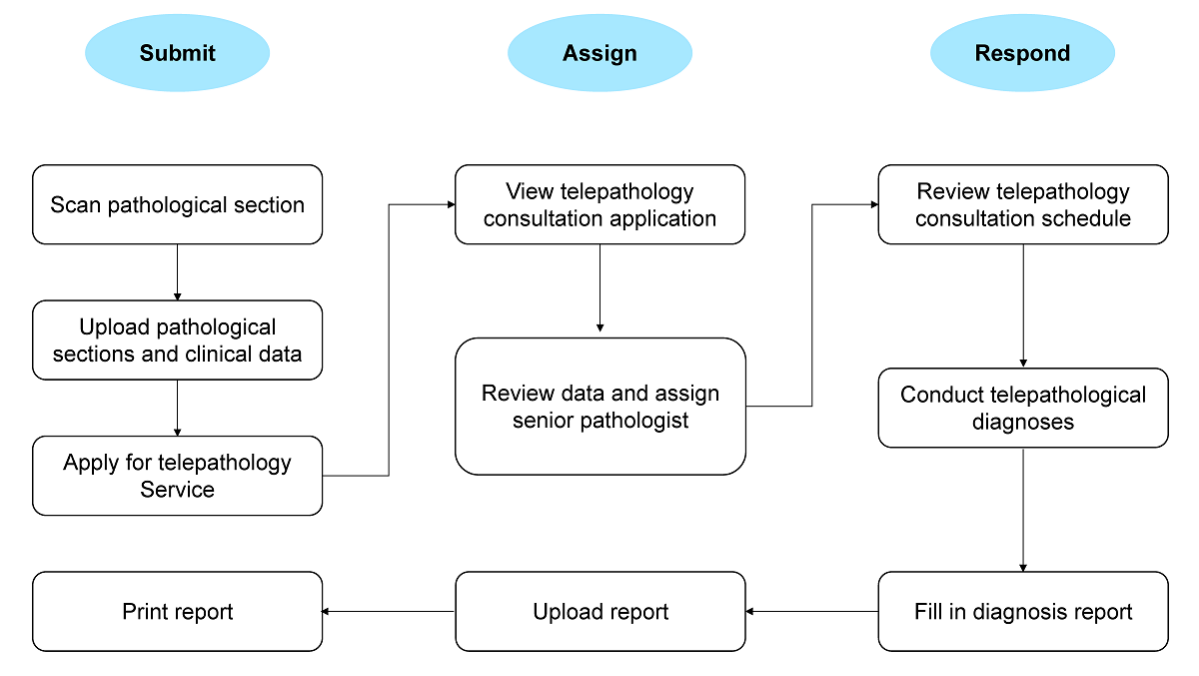
Telepathology consultation process.

There are 17 specialists from the Department of Pathology of the First Affiliated Hospital of Zhengzhou University who currently participate in telepathology consultation, including 8 professors and 9 associate professors specializing in different fields. The consultation is a voluntary activity with no charge. Pathologists from participating hospitals scanned and uploaded the slides to be diagnosed with patient information to the cloud platform. Coordinators from the National Telemedicine Center of China then assigned these cases to specialists (based on their specialties and fields), who are very likely to be able to provide confirmed diagnoses and valuable suggestions for corresponding therapies (Figure 2).

#### CNCTPS Implementation Stages

The system was implemented in 3 stages. First, the participating hospitals were selected, starting in August 2015, based on medical service quality, readiness of their pathology departments and telemedicine services, and their willingness to use telepathology. Second, system hardware and software were deployed. Starting in January 2016, our technicians installed and debugged the equipment in participating hospitals. Third, personnel training and system maintenance were conducted. This included intensive training at the National Telemedicine Center of China (Multimedia Appendix 2) and on-site training in their hospitals. In addition, to ensure the normal operation of the system, technicians provide regular maintenance of the hardware and software in participating hospitals. System operation guides were also provided to the participating hospitals (Multimedia Appendix 3).

### Data Collection

To analyze the service volume, turnaround time, and economic benefits of the CNCTPS, we collected all case data submitted from January 2016 to December 2019, which included demographic and clinical data, submitted hospital, case submission time, report issuance time, telepathology diagnosis, and specialist who made the diagnosis. After removing test cases, there were 23,167 cases. Specimens had been taken from multiple organs, which were divided into 26 groups.

To analyze the diagnostic accuracy of the CNCTPS, we followed up the final diagnosis of all the 23,167 cases through the hospital information system of the First Affiliated Hospital of Zhengzhou University. We searched and found that 564 cases had also been diagnosed directly in the First Affiliated Hospital of Zhengzhou University. The diagnostic accuracy of telepathology was calculated by using the final diagnosis in the First Affiliated Hospital of Zhengzhou University as the reference.

### Statistical Analysis

Descriptive statistics were used to analyze characterize case data, including demographic characteristics of patients from whom samples were taken, diagnosis, histopathology type, and turnaround time. The median value and interquartile range are reported for continuous data, and percentages are reported for categorical data. The Kruskal–Wallis H test was used to compare turnaround time in different years, and the Nemenyi test was used for further multiple comparisons. The concordance between CNCTPS and final diagnoses was analyzed (complete concordance or variance with no clinical significance). The consistency was determined by the McNemar test and consistency check. All statistical analyses were performed using R software (version 4.0.0; R Foundation for Statistical Computing). All tests were 2-tailed, and *P*<.05 is considered statistically significant.

## Results

### CNCTPS Service Volume

**Figure 3:**
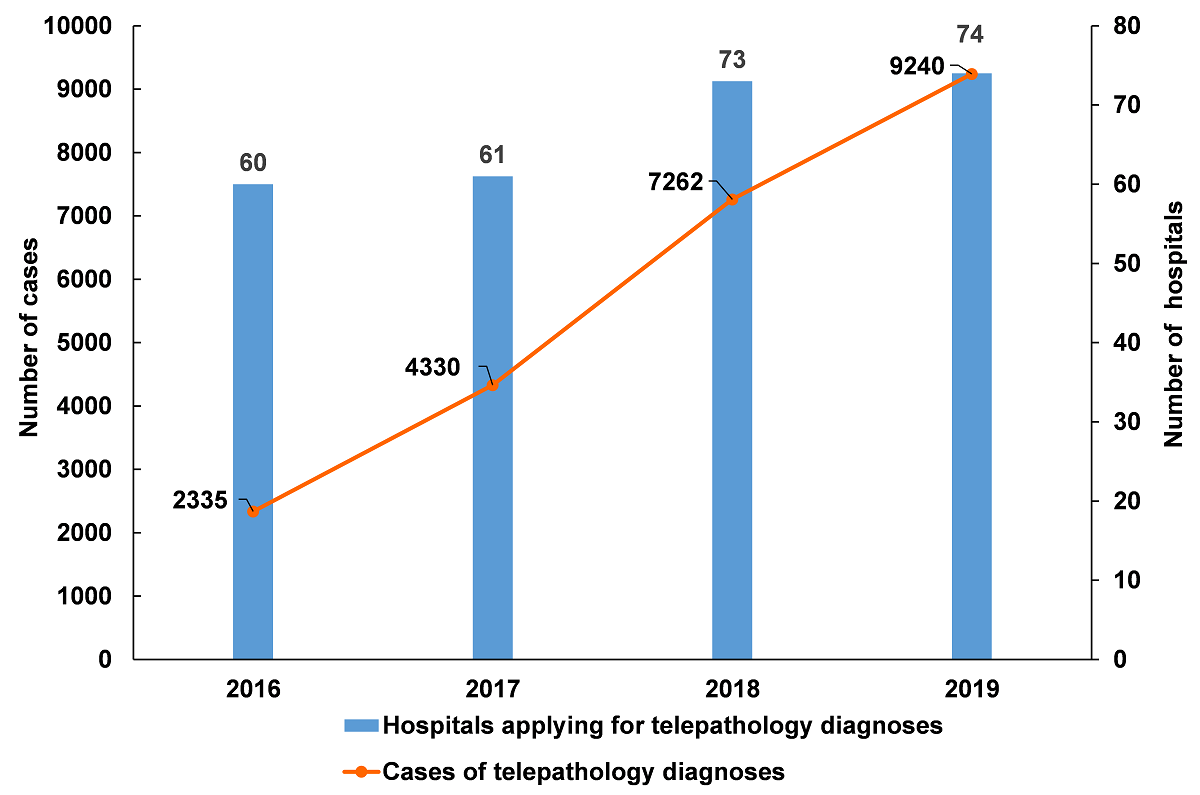
Participating hospitals and submitted cases from 2016 to 2019.

During the 4-year study period from 2016 to 2019, 23,167 cases were submitted to the CNCTPS for consultation. The service volume of the CNCTPS was n=2335 in 2016; n=4330 in 2017; n=7262 in 2018; and n=9240 in 2019, with an average annual growth rate of 41.04%. A total of 83 hospitals participated in the telepathology consultation service. The number of participating hospitals also grew, from n=60 in 2016 to n=74 in 2019 (Figure 3).

Hospitals of different levels have joined the CNCTPS, including 17 city-level and 66 county-level hospitals. Among 2016 and 2019, city-level hospitals and county-level hospitals applied for 2880 (2880/23,167, 12.43%) and 20,287 (20,287/23,167, 87.57%) consultations, respectively. The number of county-level hospitals applying for consultation increased from n=49 in 2016 to n=63 in 2019, and the service volume also increased from n=2095 in 2016 to n=8317 in 2019. In city-level hospitals, the number of hospitals applying for consultations did not change, while the service volume showed an overall increasing trend (Table 1).

**Table 1:** Number of participating hospitals and service volume in different levels of hospitals from 2016 to 2019.

Hospital level			2016, n		2017, n		2018, n		2019, n
**City-level**									
	Hospitals	11		9		14		11	
	Service volume	240		657		1060		923	
**County-level**									
	Hospitals	49		52		59		63	
	Service volume	2095		3673		6202		8317	

### Characteristics of Cases Submitted to the CNCTPS

The locations, from which specimens had been taken, were divided into 26 groups (Table 2).

Of the 23,167 patients represented by case data, 9519 (41.09%) were male and 13,648 (58.91%) were female (Table 3). The median age of the patients, from whom specimens were taken, was 53 years (mean 52.86 years, range 1 day to 98 years). There were 17,495 out of 23,167 cases (75.52%) with confirmed diagnoses; 4779 out of 23,167 cases (20.63%) needed further examination, and most (4007/4779, 83.85%) required immunohistochemical examination. The other 893 (893/23,167, 3.85%) cases failed to be diagnosed, and poor slice quality and incomplete sampling were the main reasons thereof.

**Figure 4:**
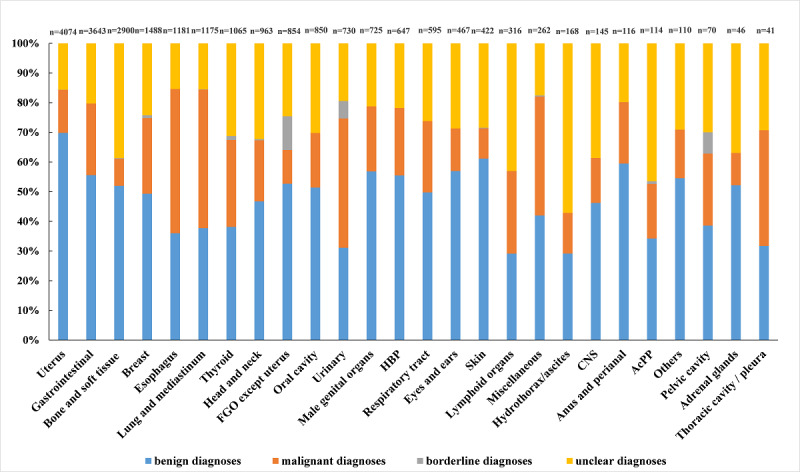
Histopathology distribution for 26 anatomic locations. AcPP: abdominal cavity/peritoneum/postperitoneum; CNS: central nervous system; HBP: hepatobiliary and pancreas; FGO: female genital organs.

Among 17,495 confirmed cases, 12,088 were benign lesions, 5217 were malignant lesions, and 190 were borderline lesions. In total, 52.18% (12,088/23,167) benign cases and 22.52% (5217/23,167) malignant cases had been confirmed. The proportion of malignant lesions in the esophagus, lung/mediastinum, urinary, and thoracic cavity/pleura was higher than that of benign lesions (Figure 4). In the other 22 tissue types, the proportion of benign lesions was higher than that of malignant lesions.

**Table 2:** Anatomic sites of specimens.

Anatomic site	Value (n=23,167), n (%)
Uterus	4074 (17.59)
Gastrointestinal	3643 (15.72)
Bone and soft tissue	2900 (12.52)
Breast	1488 (6.42)
Esophagus	1181 (5.10)
Lung/mediastinum	1175 (5.07)
Thyroid	1065 (4.60)
Head and neck	963 (4.16)
Female genital organs except for uterus	854 (3.69)
Oral cavity	850 (3.67)
Urinary	730 (3.15)
Male genital organs	725 (3.13)
Hepatobiliary and pancreas	647 (2.79)
Respiratory tract	595 (2.57)
Eyes and ears	467 (2.02)
Skin	422 (1.82)
Lymphoid organs	316 (1.36)
Miscellaneous	262 (1.13)
Hydrothorax/ascites	168 (0.73)
Central nervous system	145 (0.63)
Anus and perianal	116 (0.50)
Abdominal cavity/peritoneum/postperitoneum	114 (0.49)
Others	110 (0.47)
Pelvic cavity	70 (0.30)
Adrenal glands	46 (0.20)
Thoracic cavity/ pleura	41 (0.18)

**Table 3:** Case and patient characteristics.

Variables		Values
**Sex, n (%)**		
	Male	9519 (41.09)
	Female	13,648 (58.91)
Age, mean (range)		52.86 (1 day to 98 years)
**Diagnosis types, n (%)**		
	Confirmed	17,495 (75.52)
	Needed further examination^a^	4779 (20.63)
	Failed to be diagnosed^b^	893 (3.85)
**Histopathology types, n (%)**		
	Benign	12,088 (52.18)
	Borderline	190 (0.82)
	Malignant	5217 (22.52)
	Unclear	5672 (24.48)

^a^Further examinations included immunohistochemistry assay (4007/4779, 83.85%), clinical examinations (675/4779, 14.12%), gene detection (88/4779, 1.84%), and special staining (9/4779, 0.19%).

^b^The reasons included poor slice quality (531/893, 59.46%), incomplete sampling (336/893, 37.63%) and intractable cases (26/893, 2.91%).

### CNCTPS Turnaround Time

The turnaround time, the time from transmitting whole-slide images to the issuance of diagnostic reports, was a median of 16.93 hours (IQR 32.59; mean 24.93 hours, range 100 seconds to 167.97 hours). Experts’ opinion reports were released within 12 hours in 10,244 of the 23,167 cases (10,244/23,167, 44.05%) and within 72 hours in 21,286 cases (21,286/23,167, 91.88%) (Table 4).

**Table 4:** Turnaround time for expert reports.

Time required (hours)	Cases, n (%)	Cumulative %
Time≤12	10,204 (44.05)	44.05
12<time≤24	4631 (19.99)	64.04
24<time≤48	4366 (18.85)	82.88
48<time≤72	2085 (9.00)	91.88
Time>72	1881 (8.12)	100

**Figure 5:**
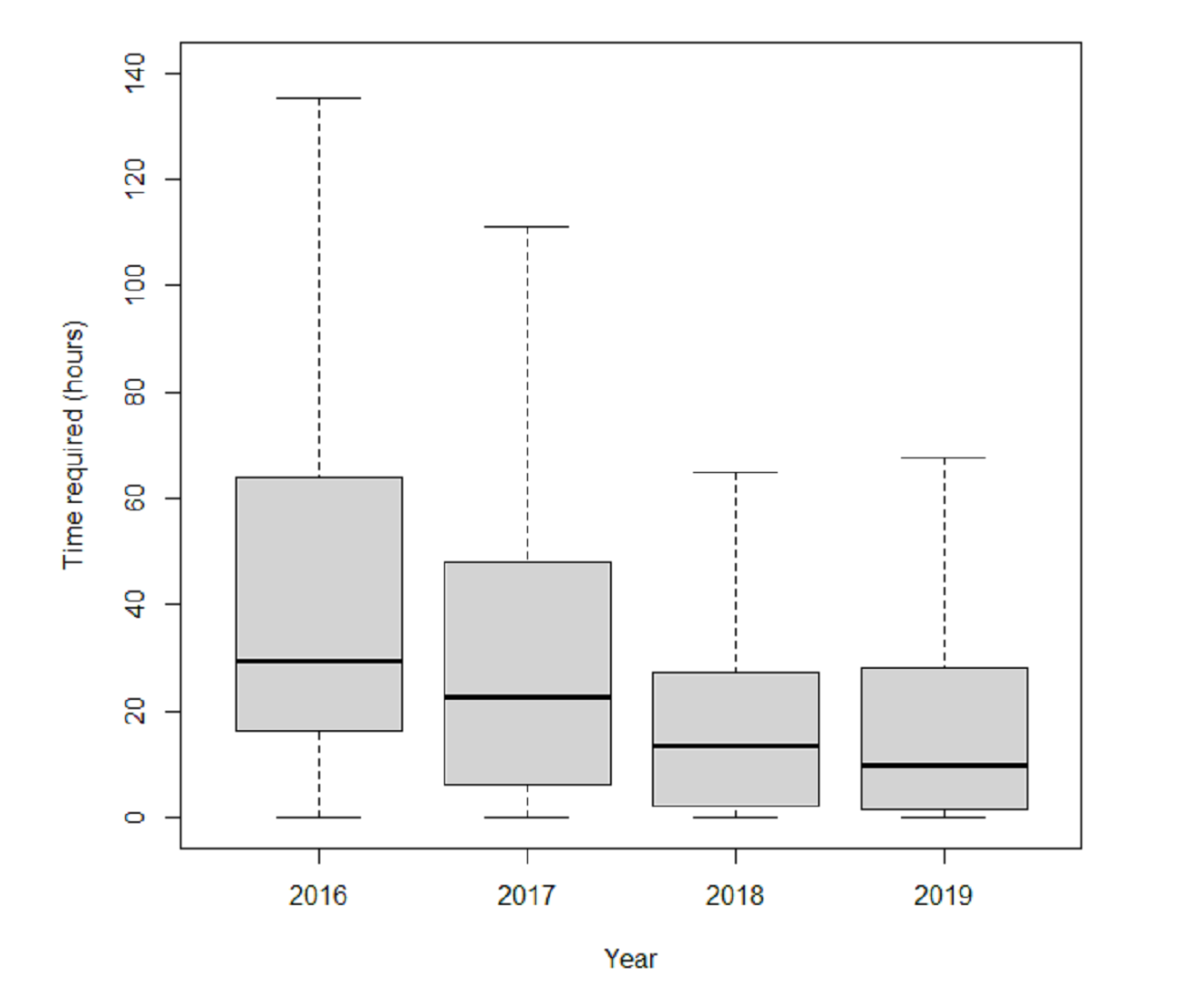
Turnaround time distribution.

The difference in distribution of turnaround time in different years (Figure 5), was statistically significant (H=1433.62, *P*<.001). The median turnaround time gradually decreased in turn, from 29.36 hours in 2016 to 9.75 hours in 2019, and differences between subsequent years were statistically significant with pairwise comparison (between 2018 and 2019 adjusted *P*=.01, other groups adjusted *P*<.001).

### CNCTPS Diagnostic Accuracy

Of 564 diagnosed by both the CNCTPS and pathologists in the hospital, 553 cases diagnosed by the CNCTPS were consistent with the final diagnosis made by pathologists in hospital; that is, the accuracy rate was 98.05%. In the other 11 cases—4 false-positive cases and 7 false-negative cases—5 of the 11 cases occurred in the uterus (Table 5).

The sensitivity and specificity were 97.66% and 98.49%, respectively (Table 6). The Youden index was 0.96. The positive and negative predictive values were 98.65% and 97.39%, respectively. No statistical difference was observed between telepathology diagnosis and final diagnosis (*P*=.55), which showed good consistency (κ=0.96, *P*<.001).

**Table 5:** Discordant cases (between telepathology and final diagnoses).

Type			Sample source		Telepathology diagnosis		Final diagnosis		Annotation
**False positive^a^**									
	Case 1	Lung/mediastinum		Poorly differentiated carcinoma		Immunoglobulin M–positive lymphoproliferative disease with alveolar epithelial atypical hyperplasia		Unlabeled immunohistochemistry results	
	Case 2	Respiratory tract		Highly differentiated squamous cell carcinoma		Squamous papillary hyperplasia with local typical hyperplasia		Intractable case	
	Case 3	Uterus		Endometrial complex hyperplasia, local atypical hyperplasia, focal canceration		Simple endometrial hyperplasia		N/A^b^	
	Case 4	Uterus		Papillary squamous cell carcinoma		High-grade SIL^c^ involving glands		A bleeding background on the section	
**False negative^d^**									
	Case 1	Female genital organs		Vulva: chronic inflammation with low-grade SIL Cervix: chronic inflammation with high-grade SIL		Vulva: highly differentiated squamous cell carcinoma with local superficial infiltration (depth of infiltration <1 mm) Cervix: chronic cervicitis, focal high-grade SIL, and involving glands		Unlabeled immunohistochemistry results	
	Case 2	Skin		Chronic inflammation, squamous epithelial hyperplasia with hyperkeratosis and parakeratosis		Superficial spreading malignant melanoma		Intractable case	
	Case 3	Uterus		Chronic inflammation, glandular hyperplasia		Minimal deviation adenocarcinoma		N/A	
	Case 4	Uterus		Chronic cervicitis with focal high-grade SIL and involving glands		High-grade SIL involving glands and squamous cell carcinoma in situ		N/A	
	Case 5	Thyroid		Adenomatous nodular goiter with fibrosis and chronic lymphocytic thyroiditis around		Follicular carcinoma		Unlabeled immunohistochemistry results	
	Case 6	Urinary		Mucosal polypoid hyperplasia with atypical urothelial hyperplasia		High-grade urothelial carcinoma		N/A	
	Case 7	Uterus		SIL		Squamous cell carcinoma		A bleeding background on the section	

^a^Cases that were malignant in telepathology diagnosis but benign in final diagnosis were considered false positive.

^b^N/A: not applicable.

^c^SIL: squamous intraepithelial lesion.

^d^Cases that were malignant in the final diagnosis but benign in telepathology diagnosis were considered false negative.

**Table 6:** Validity of CNCTPS diagnoses.

Telepathology diagnosis	Final diagnosis		
	Positive, n	Negative, n	Total, n
Positive	292	4	296
Negative	7	261	268
Total	299	265	564

### Economic Benefits of the CNCTPS

Telepathology consultation is free and avoids the need for patients having to visit higher-level hospitals. Therefore, consultation and travel costs were saved. At the same time, food costs were lower in the local area. Thus, compared with the traditional pathology consultation, diagnosis via the CNCTPS results in cost-savings of 378.5 RMB (approximately US $50) per patient (Table 7). In terms of the annual telepathology consultation cases, the total amount is substantial—approximately $300,000 per year.

**Table 7:** Cost savings for each patient applied for telepathology consultation.

Type of costs	Telepathology consultation cost (RMB^a^)	Traditional pathology consultation cost (RMB)	Costs savings (RMB)
Consultation costs	0	148.5^b^	148.5
Travel costs^c^	0	200	200
Food costs^c^	25	55	30
Total	25	403.5	378.5

^a^RMB: Renminbi; an approximate exchange rate of 6.46 RMB = US $1 is applicable.

^b^Traditional consultation costs referred to the pathology consultation charges in Henan Province.

^c^For each patient, one person going for a consultation was assumed. Travel and food costs were calculated using estimates of local corresponding average expenses.

## Discussion

### Principal Results

The cloud-based system can quickly process data with large memory requirements, thereby overcoming the difficulties of large whole-slide imaging file management. This study reported on one of the largest cloud-based telepathology systems in China and evaluated its operation results. This study used a large sample size, which provides an in-depth practical understanding of the cloud-based telepathology system in China, and gives suggestions for further evaluations and improvements of the telepathology system. This system served 23,167 cases from 2016 to 2019. The median turnaround time was 16.93 hours, which decreased from 29.36 hours in 2016 to 9.75 hours in 2019. The diagnostic accuracy was 98.05%, and approximately $300,000 were collectively saved by patients each year. The CNCTPS has proven to be highly reliable and plays an important role in facilitating the distribution of limited senior pathologist resources in China.

A total of 83 hospitals are covered by the CNCTPS, which is the largest telepathology network in China. Compared with the 6, 24, and 60 workstations in other reported telepathology networks [23,24,26], the CNCTPS covers more medical institutions. Case data for more than 20,000 patients were diagnosed by the CNCTPS in 4 years. To the best of our knowledge, this is the largest sample size in a study on telepathology system use and operation and is much higher than those in similar literature [24,26-28]. The amount of case data reviewed and the number of participating hospitals increased each year, consistent with findings reported by Chen et al [26] and Zhou et al [6]. Most case data (20,287/23,167, 87.57%) had been submitted by county-level hospitals because the shortage of pathologists in China’s county-level hospitals is more severe than that in city-level hospitals.

A total of 893 cases failed to be diagnosed by the CNCTPS, of which only 26 cases were complicated enough that needed to be consulted in a higher-level hospital, while others were due to incomplete sampling and poor slice quality. Standard materials and good slice preparation are the main factors affecting telepathology diagnosis [29], which require experienced pathology technicians. Although we had conducted theory and practical operation training for pathology technicians in the early stage of CNCTPS construction, incomplete sampling and poor slice quality were still the main reasons for failed diagnoses. Strengthening the training of telepathology staff in the later stages of system operation is still needed. In terms of histopathology type, an analysis of ten-year telepathology cases in Tanzania showed a higher proportion of benign cases, which reported 56.1% benign and 40.8% malignant diseases [27]. We reached a similar conclusion: the proportion of benign cases (12,088/23,167, 52.18%) was higher than that of malignant cases (5217/23,167, 22.52%).

The average turnaround time of the CNCTPS was 24.93 hours, which is shorter than the 38 hours reported by Zhou et al [6] and 66 hours reported by Völker et al [27] but is slightly higher than the 0.7 days (ie, 16.8 hours) reported by Chong et al [24]. The majority (14,835/23,167, 64.04%) of cases were diagnosed within 24 hours, which is higher than the 61.5% reported by Chen et al [26] and slightly lower than the 64% reported by Perron et al [23], but the proportion within 48 hours (82.88% vs 70.00%) and 72 hours (91.88% vs 80.00%) were higher than those reported by Perron et al [23]. Nonetheless, the median turnaround time decreased annually during the 4 years, indicating that the CNCTPS operates well.

Compared to static images in the early stage of telepathology, whole-slide imaging allows the entire slide to be viewed in a manner that simulates microscopy [13]. A recent meta-analysis [30] shows that the weighted mean of the concordance rates between telepathology and conventional microscopy was 91.1% up to 2000, and from 2000 onward, the weighted mean of the concordance rates was 97.2%. It has been asserted that the reasons for increased consistency rate in recent years should be attributed to the increased use of whole-slide imaging [30]. The range of diagnostic concordance rates between whole-slide imaging and traditional electron microscopy is 89% to 100% [31-35], and the average value is 96.9%. Our study demonstrated similar results (98.05%). Moreover, no statistically significant differences were found (*P*=.55) between whole-slide imaging and traditional pathology diagnosis, and the consistency of diagnostic results was excellent, which further confirmed the accuracy of whole-slide imaging.

Some cost-effectiveness studies have demonstrated that telemedicine can reduce costs [36], but not all [37,38]. Cost-utility and cost-effectiveness studies for telepathology are rare. Meléndez-Álvarez et al [17] only evaluated the cost of their telepathology system, which saved US $410. Vosoughi et al [39] evaluated the cost-efficiency of their real-time nonrobotic telepathology system, which saved US $10,767.10 per year. In our study, cost savings for patients were estimated. During the 4 years of the telepathology system operation, approximately US $300,000 per year was saved by patients.

### Limitations

To the best of our knowledge, this is the first study to comprehensively evaluate the operation of a telepathology system based on a large sample. The CNCTPS showed fast responsiveness and high accuracy. However, owing to the limited information collected by the CNCTPS, this study did not analyze the reasons for cases with long turnaround time or the reasons for false positives and false negatives. In addition, only the costs saved for patients were evaluated in the economic benefits of the CNCTPS. The economic impact of telemedicine is a collaborative and complex process in which different economic, social, and political actors can be involved [38], and the construction of our system is a public welfare project initiated by the government and a powerful hospital. Most of the digital slide scanners were donated to the participating hospitals, and the private network was free.

### Future Work

Turnaround time and diagnostic accuracy are the main criteria used to evaluate a telepathology system, and further work is required to explore the factors that influence turnaround time and diagnostic accuracy. First, it is necessary to analyze the causes of cases with long turnaround time through a survey of pathologists, especially those with turnaround times longer than 72 hours. Second, more investigation for incorrectly diagnosed images is needed. In addition, adding a follow-up module to the CNCTPS is necessary to allow the final diagnosis result of each case to be easily followed up. Finally, a user satisfaction survey should be conducted, with thorough questionnaires or in-depth interviews, in a subsequent study to improve the system.

### Conclusions

The CNCTPS has proven to be highly reliable. It can provide rapid telepathology diagnoses to participating hospitals that are consistent with the final diagnosis. The application of this system reduces financial costs and time for patients, facilitating the distribution of limited senior pathologist resources in China. Therefore, we believe telepathology services will become more widespread, in more regions worldwide, especially those with insufficient medical resources.

## Appendix

Multimedia Appendix 1 The web and app versions of the cloud-based telepathology system.

Multimedia Appendix 2 Training documents.

Multimedia Appendix 3 System operation guide for the cloud-based telepathology system.

## Abbreviations

CNCTPS: Chinese National Cloud-Based Telepathology System
RMB: Renminbi
SIL: squamous intraepithelial lesion
