# Supplementary material for: Effectiveness of a Cloud-Based Telepathology System in China: Large-Sample Observational Study
Source: J Med Internet Res. 2021 Jul 29;23(7):e23799. doi: 10.2196/23799 (PMC8367172; doi:10.2196/23799)
Supplement: Multimedia Appendix 1 [file jmir_v23i7e23799_app1.pptx]

## Slide 1
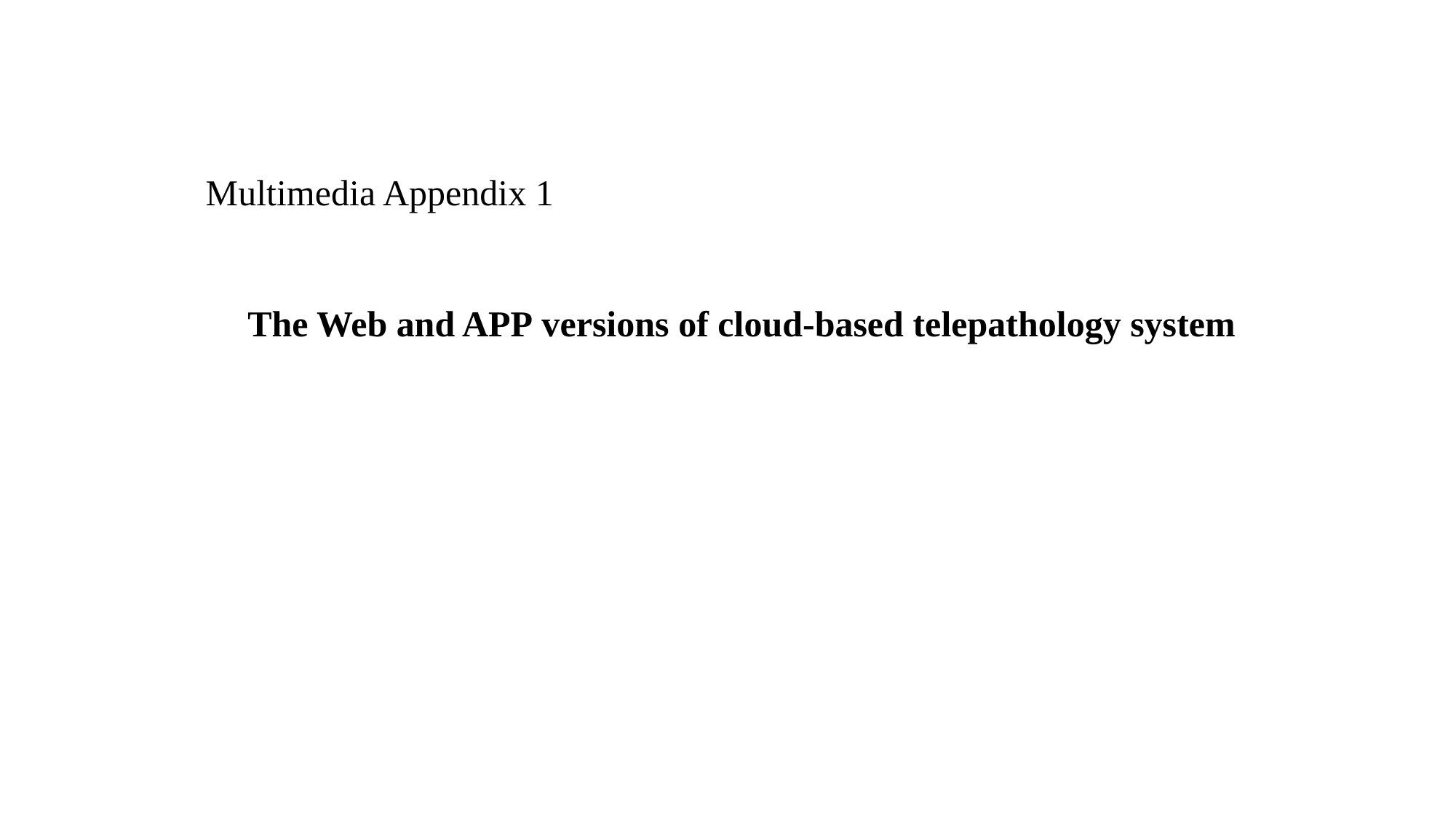

Multimedia Appendix 1
The Web and APP versions of cloud-based telepathology system

## Slide 2
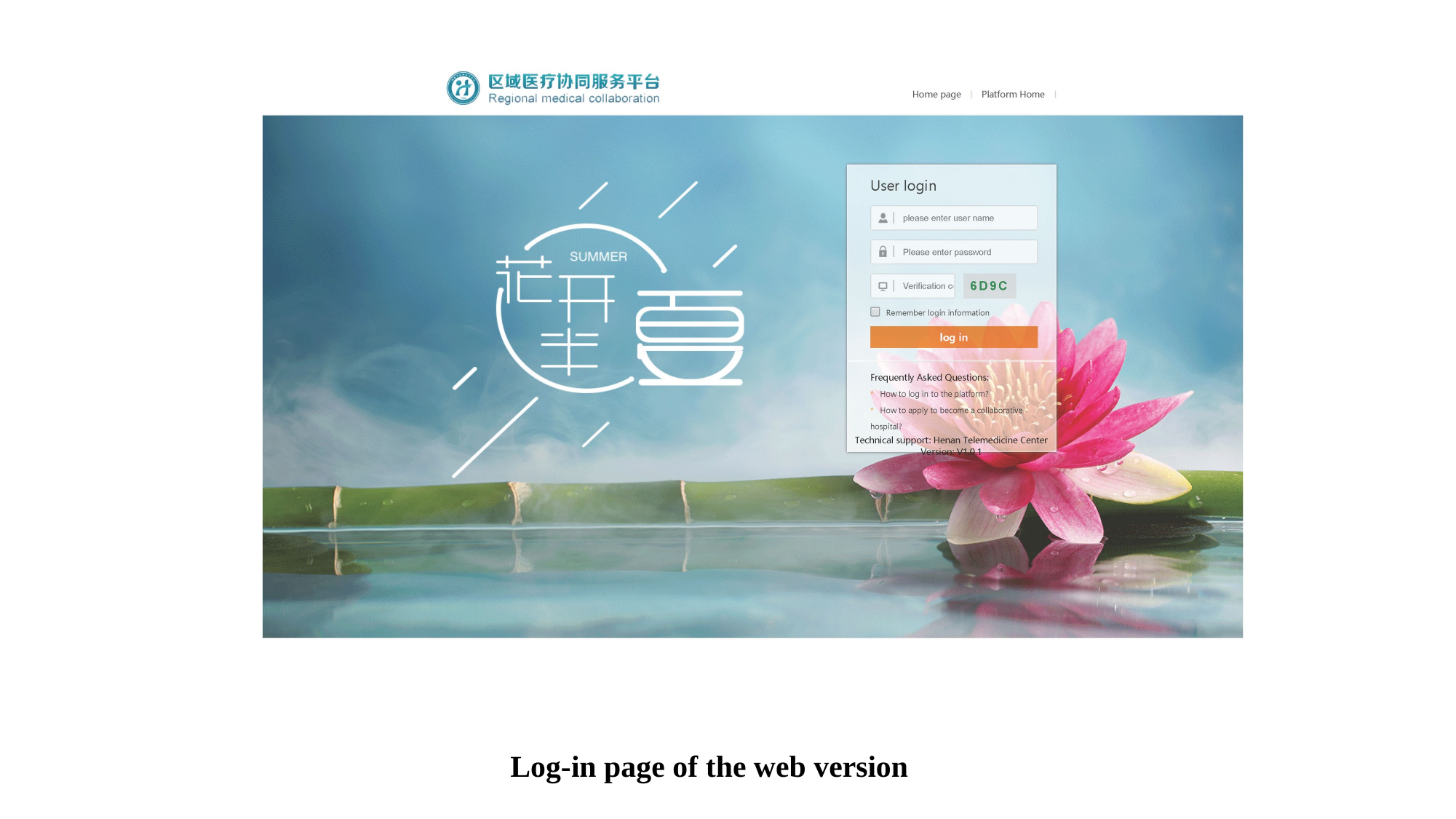

Log-in page of the web version

## Slide 3
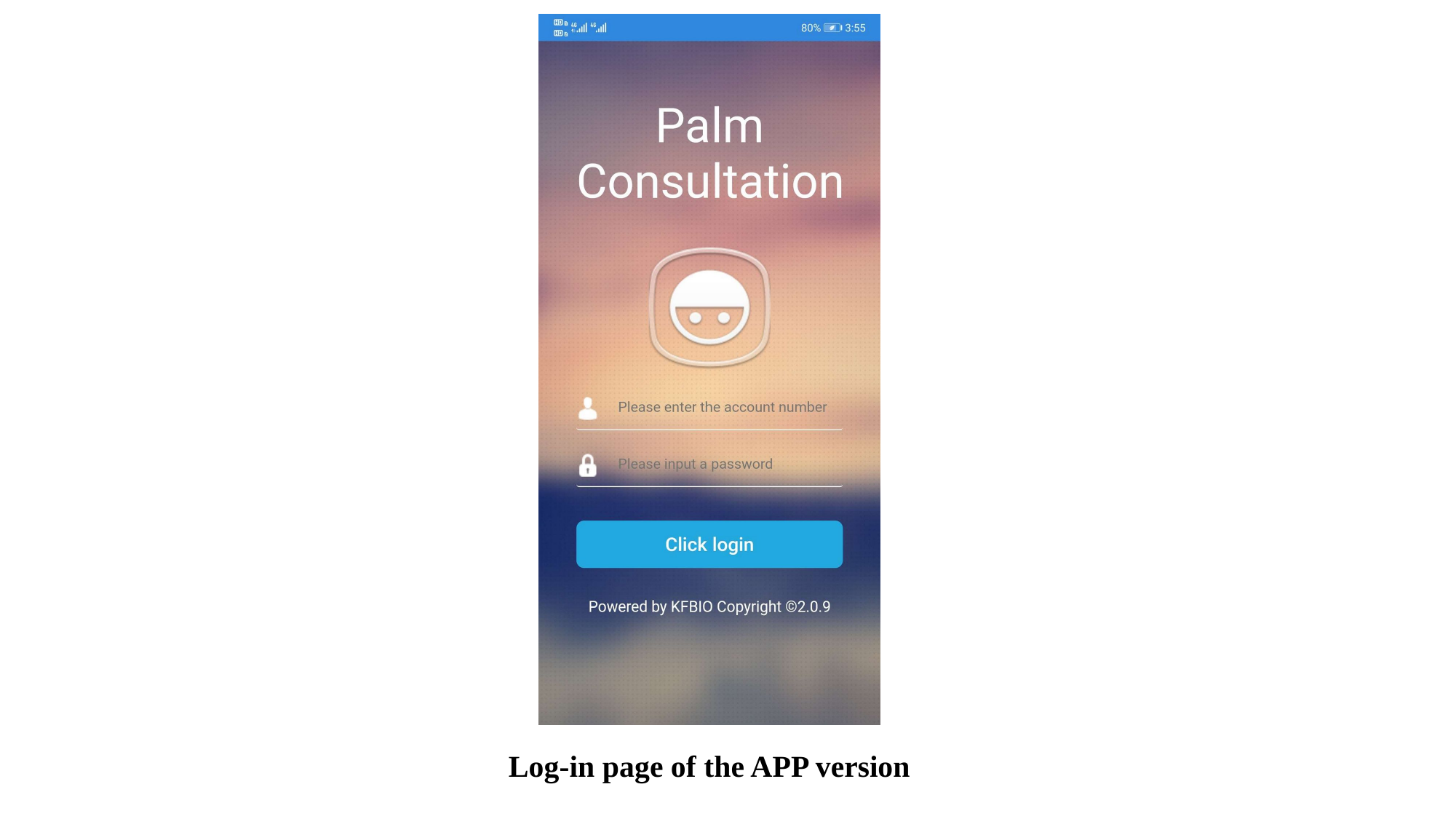

Log-in page of the APP version

## Slide 4
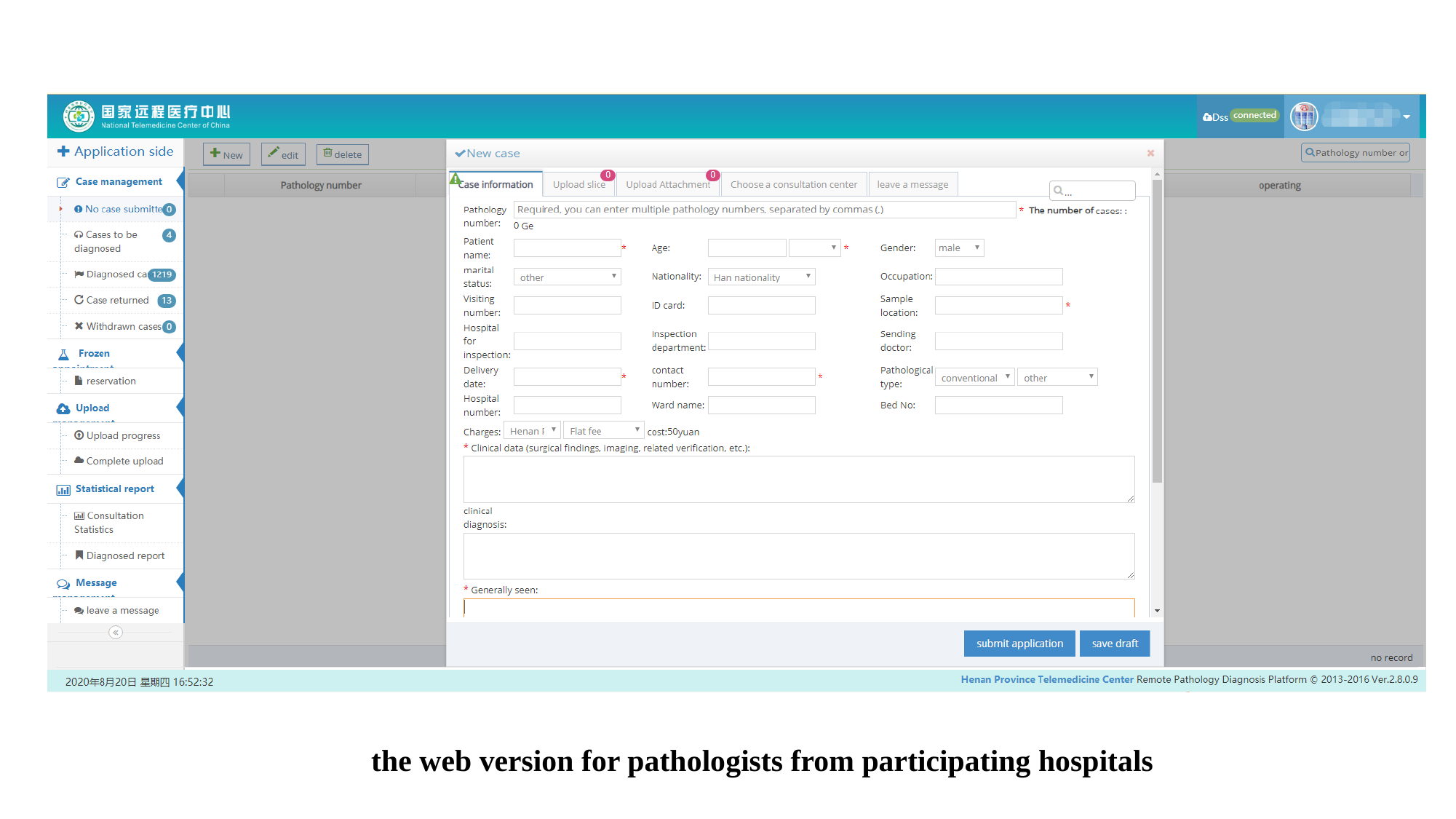

the web version for pathologists from participating hospitals

## Slide 5
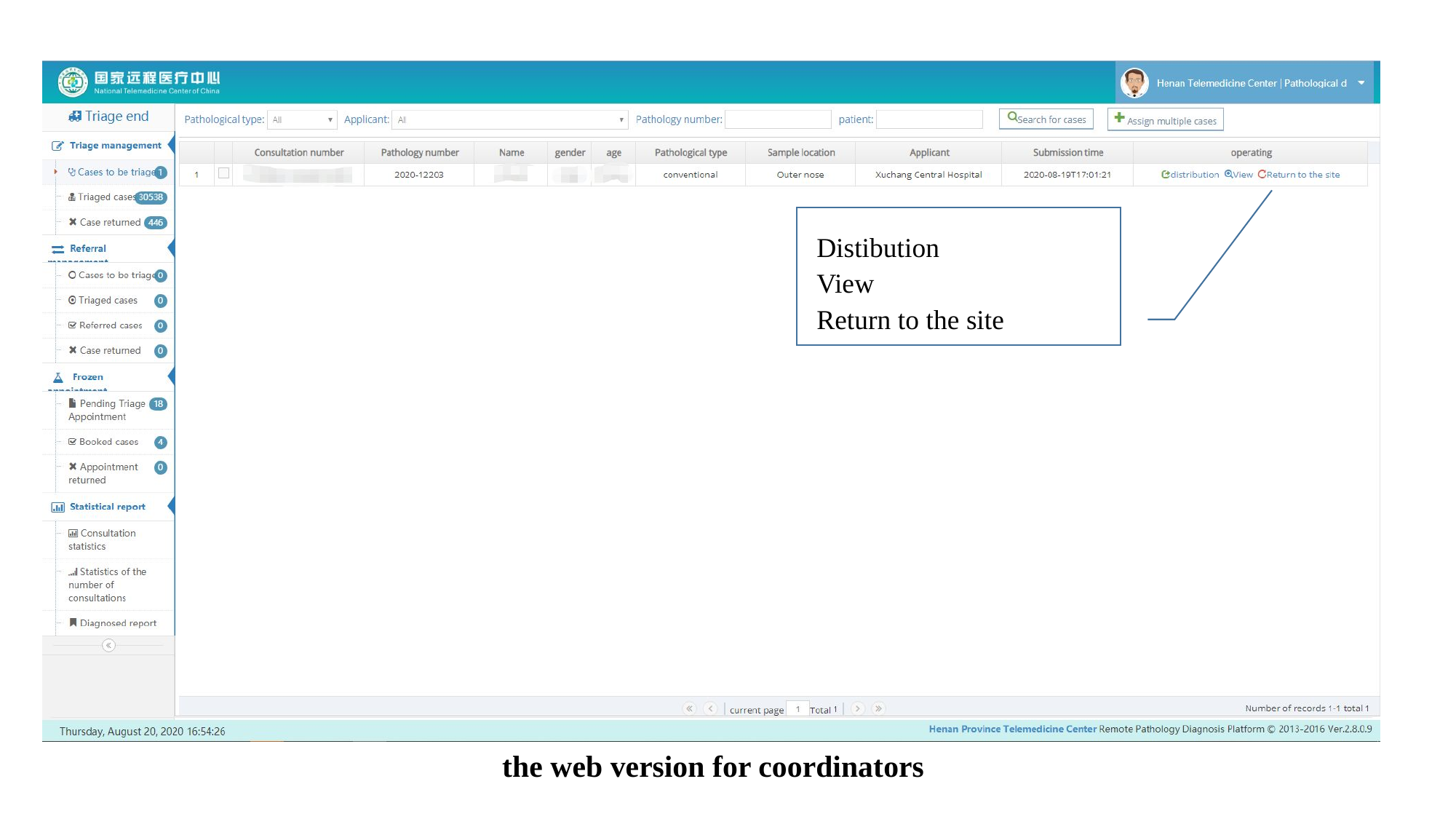

Distibution
View
Return to the site
 the web version for coordinators

## Slide 6
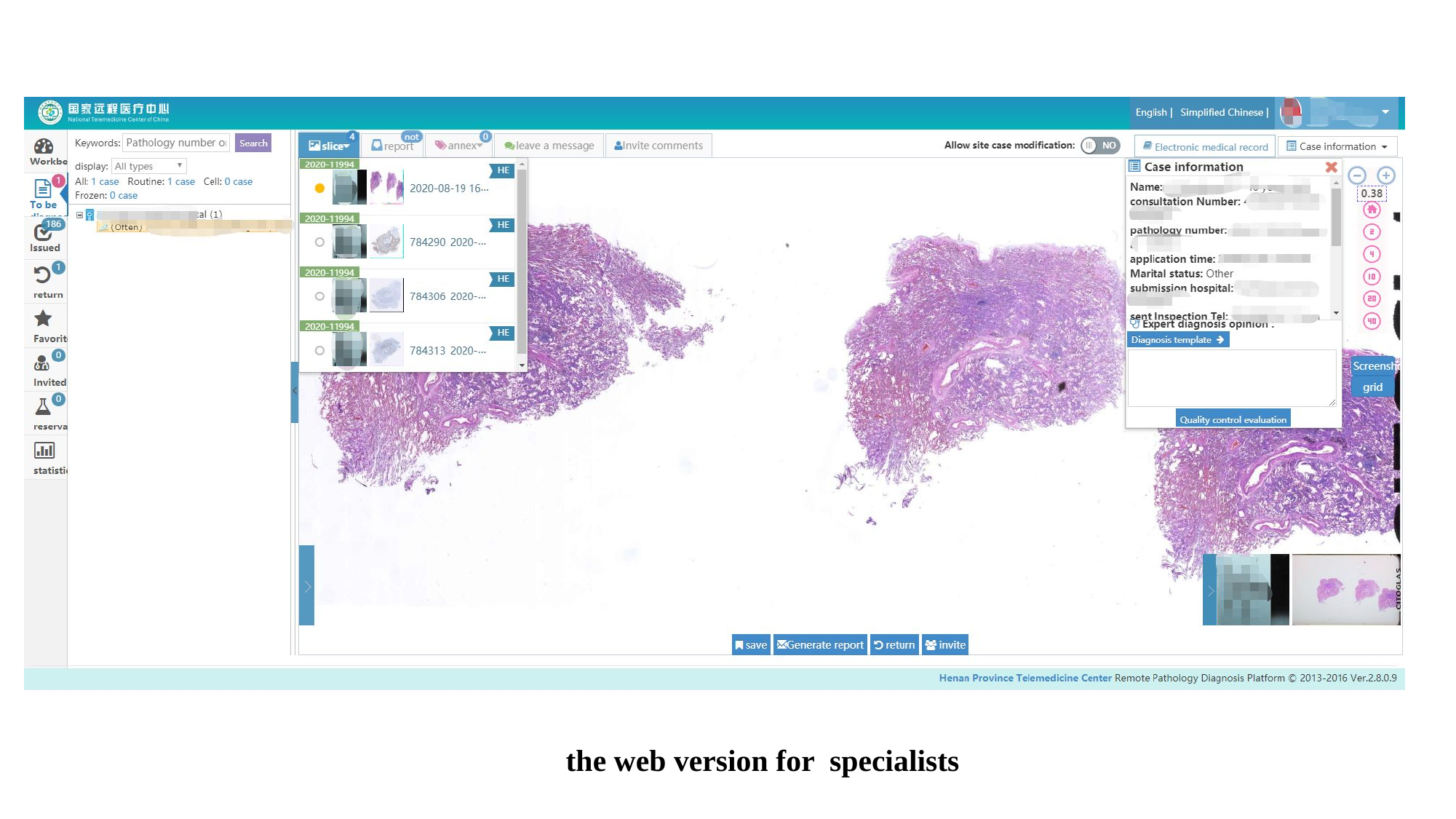

the web version for specialists

## Slide 7
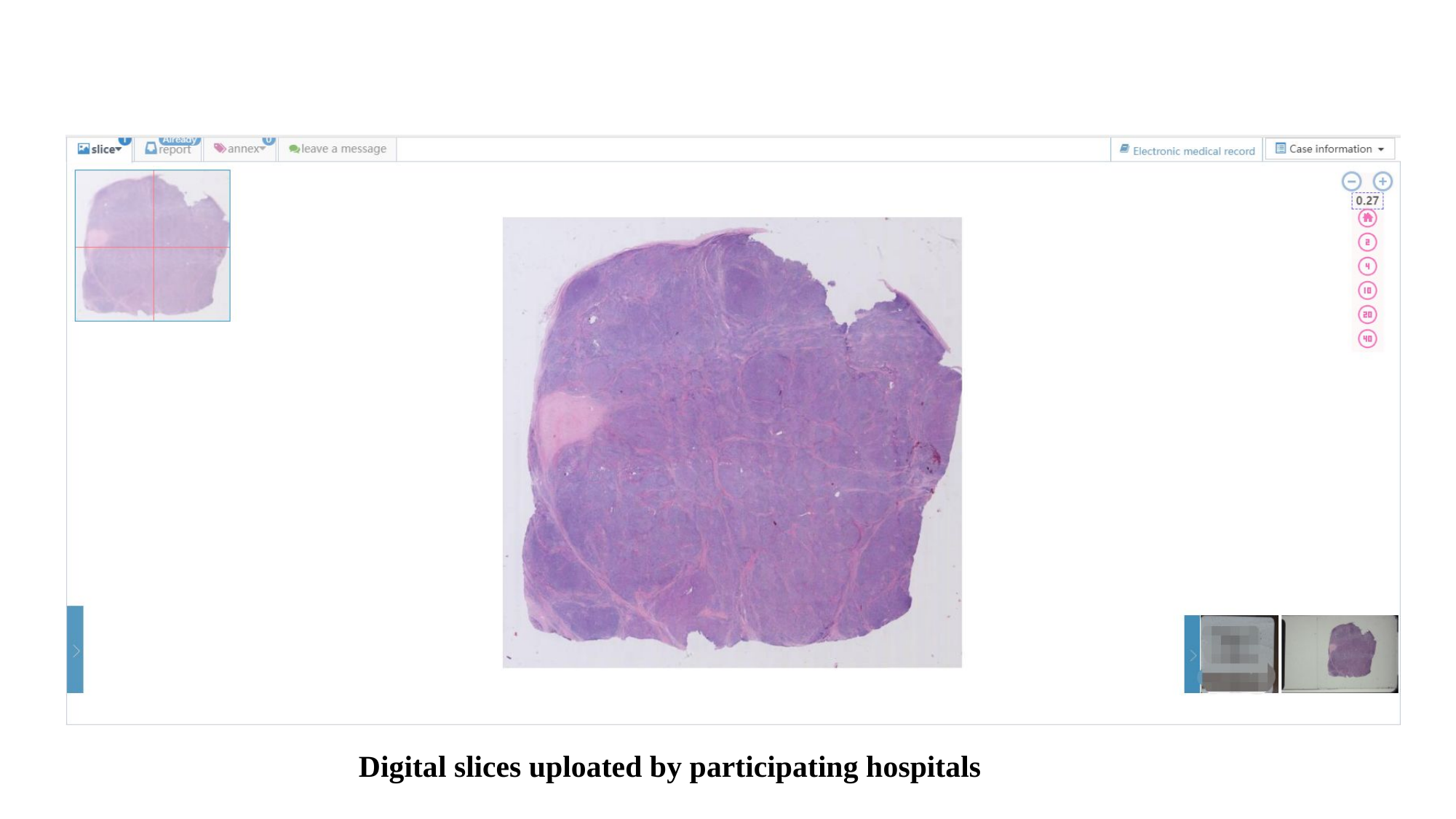

Digital slices uploated by participating hospitals

## Slide 8
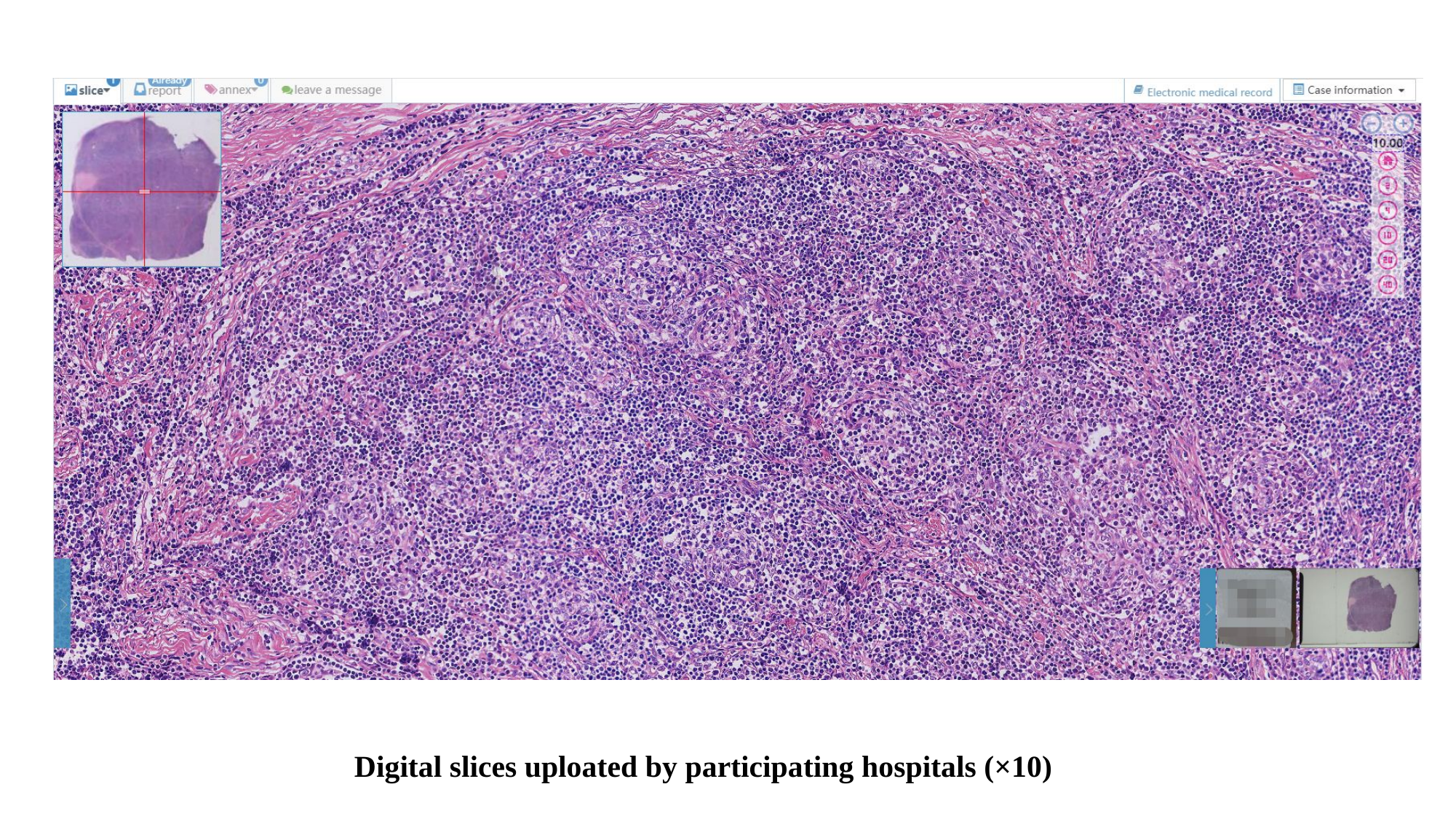

Digital slices uploated by participating hospitals (×10)

## Slide 9
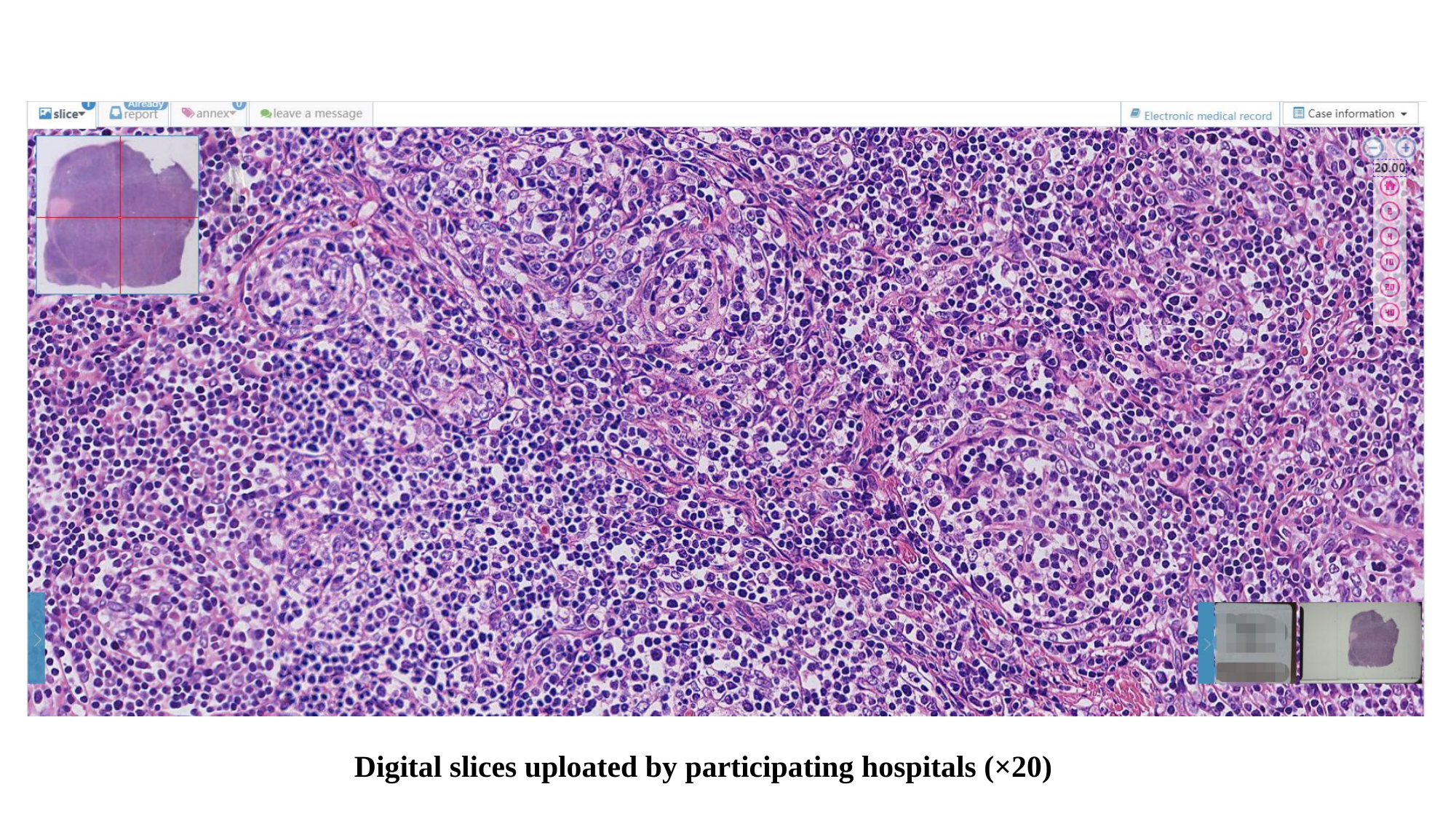

Digital slices uploated by participating hospitals (×20)

## Slide 10
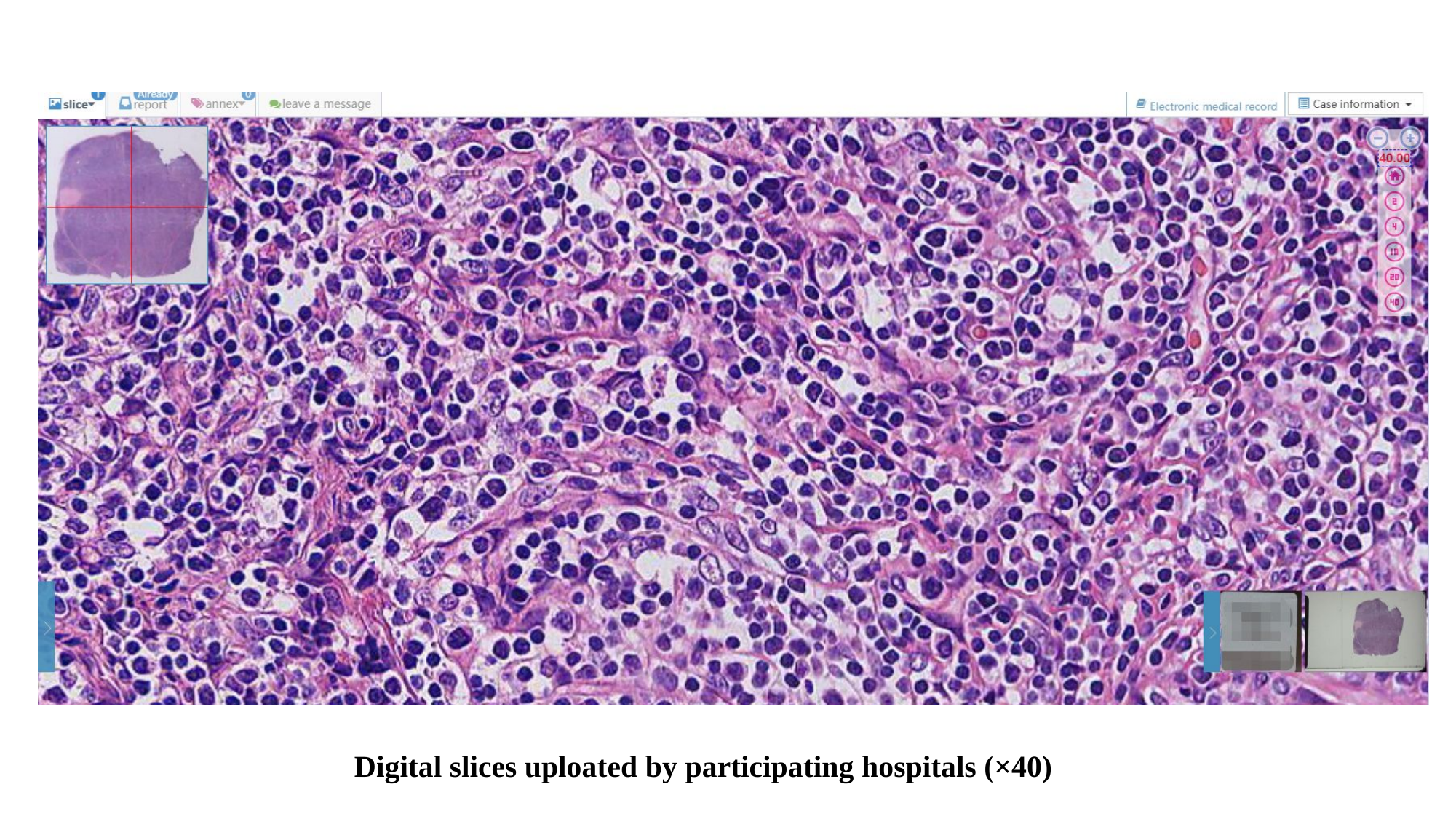

Digital slices uploated by participating hospitals (×40)
