# Supplementary material for: Effectiveness of a Cloud-Based Telepathology System in China: Large-Sample Observational Study
Source: J Med Internet Res. 2021 Jul 29;23(7):e23799. doi: 10.2196/23799 (PMC8367172; doi:10.2196/23799)

常规石蜡切片的制作要点及问题切片的注意事项

The main points of conventional paraffin sections production and the precautions for problem sections

第一部分

石蜡切片制作技术

过程：宏观-微观-宏观

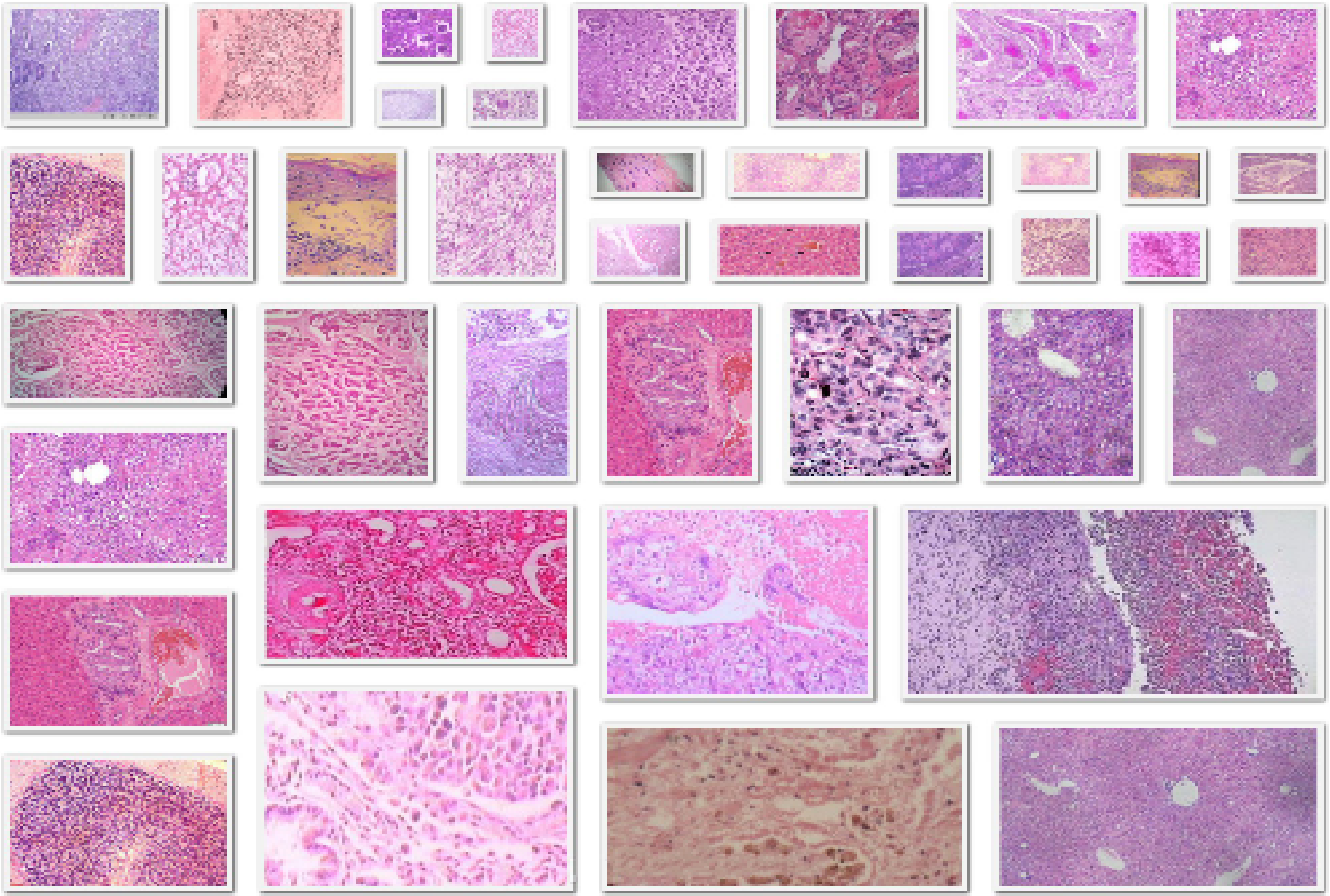

目的：服务于诊断，服务于临床治疗

原理

利用锐利的刀具将组织切成薄的片层，组织须经过一系列特殊的处理，如固定、脱水、包埋等繁琐的过程才能利于切片。

由于组织块较为柔软，直接切削困难，因此必须先用某些特殊物质（如石蜡、火棉胶、明胶、树脂等）深入组织块内部起支撑作用，并将整个组织包裹住，然后再用切片机制作切片，才能获得良好效果。其中，由石蜡为包埋剂的组织制成的切片称为石蜡切片法。

切片法虽工序繁琐，但是它能保持细胞间的正常相互关系，较长时间的保留细胞原貌，是光镜检查的主要制片方法。

轮转式切片机

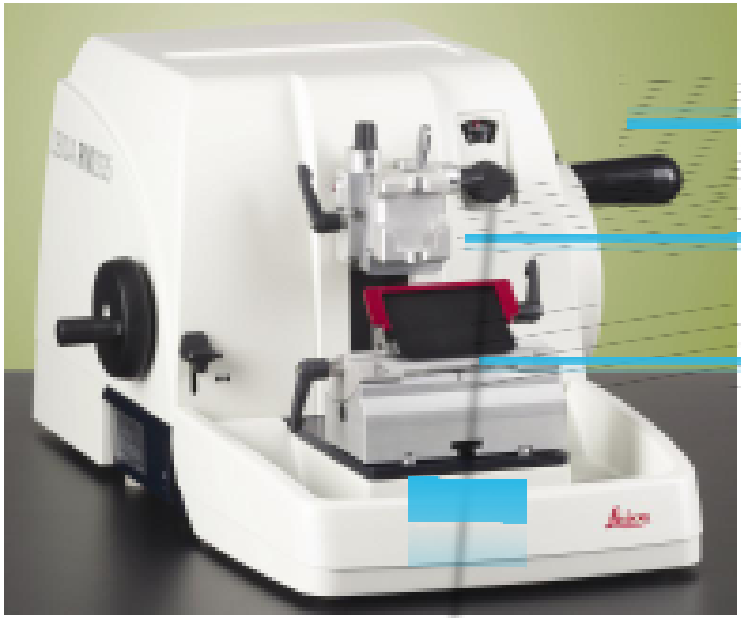

手轮

夹头

刀座

调节切片厚度的旋钮

一次性刀片

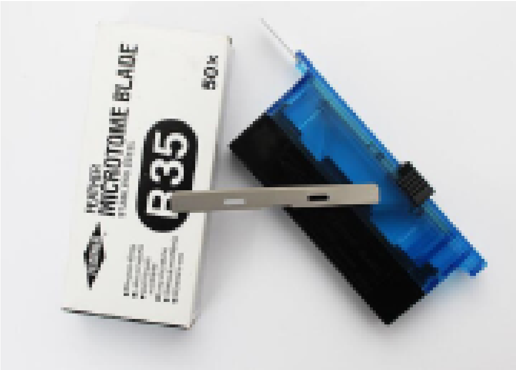

摊片机

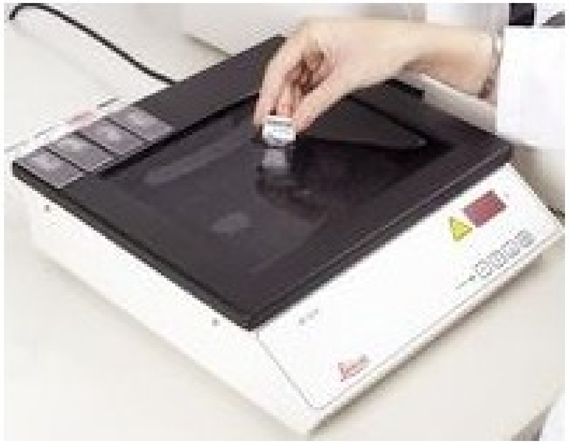

❖ 切片前的准备

包埋好的蜡块，检查一次性刀片是否锋利；

准备充足的清洁载玻片，刷子，铅笔等工具；

检查切片机功能，刀架等旋钮牢固不松动。

切片方法

1. 调节旋钮至合适的切片厚度，调节时应注意指针不可在两个刻度之间，否则容易损伤切片机。上好刀片，使切片刀平面与组织切面呈5°左右的夹角。

2. 将包埋好的组织块固定在样本夹头上，使组织块截面与样本夹头平面平行，将刀座移至近组织下缘，让刀口与组织切面稍微接触。

3.粗修：根据组织性质选择合适的粗修进刀幅度，将蜡块修至暴露组织最大面。一般而言，组织大小及硬度适中者，选择20-30μm进刀幅度，小标本或质地坚硬致密者（如子宫、脱钙不良的骨组织），应将进刀幅度降至10μm甚至更小，同时粗修速度应平稳缓和，以防用力过度导致啃块，对切片机也是一种保护。

4. 细修：蜡块粗修至一定程度后，开始以待切片的厚度为幅度细修，过程中仔细观察，直至切面平整光滑、均匀一致，无发白、卷曲、毛糙等现象。

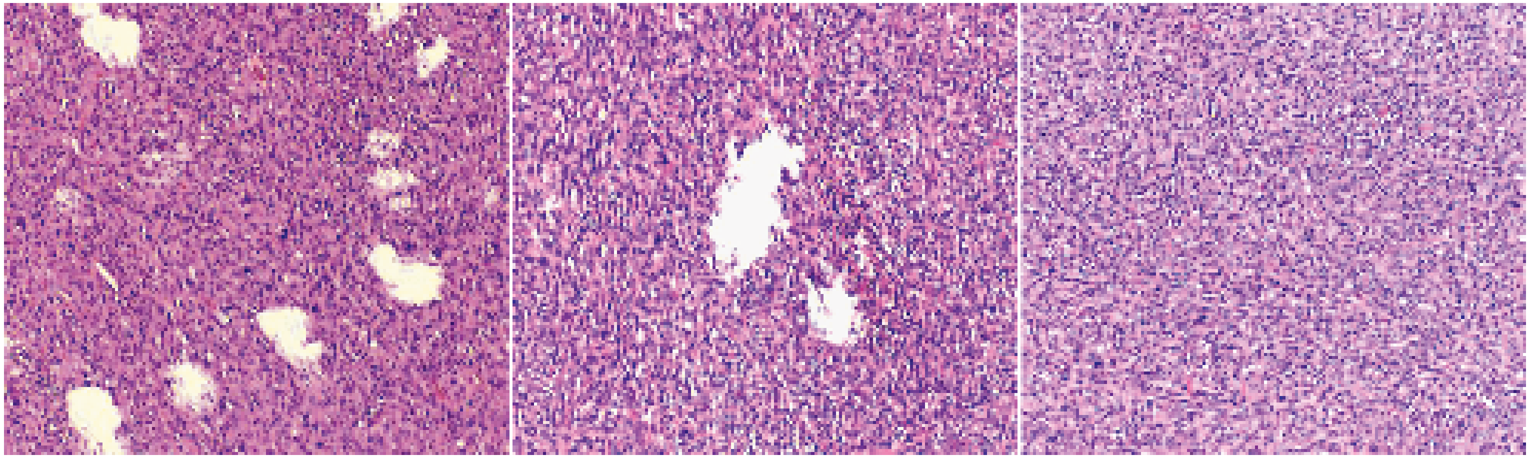

1　　2　　3

1.粗修后直接切片，镜下明显可见大小不一的空洞

2.细修不充分的切片，空洞区仍存在，但面积较图1有所减少

3.充分修切后的切片，镜下组织形态完整，染色均匀无空洞

5. 留取切片：用毛刷将刀架周围的蜡屑清理干净，再以合适的厚度进行切片，过程中保持动作轻柔并用毛刷适当牵拉切出的蜡片，保持蜡片平整光滑，无皱褶，一般切出2-3张蜡片即可，以供在漂片时选取最佳的一张进行捞片。

切片厚度的要求应根据需要选择，具体为：

- 常规HE染色，切片厚度一般为3-5μm，具体组织应该分情况切片。。
- 免疫组化、特染等切片厚度为4-6μm。
- 特殊情况下，如电镜超薄切片切片厚度为0.5-2μm。

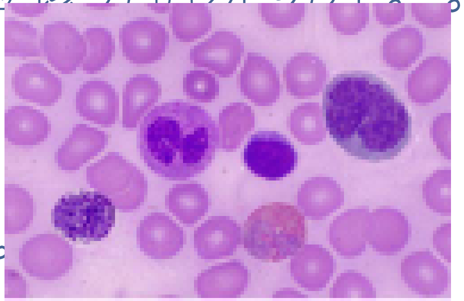

6.展片及捞片：展片水温一般应在50°左右，主要和包埋用石蜡的熔点有关，水温过高，易引起组织细胞散开，水温过低，皱褶不好摊平。捞片时注意保持玻片的清洁，选择完整、无皱褶的蜡片，粘贴于玻片中、下1/3之间。

捞片数量：

小标本捞片时要捞6片蜡片，中等大小组织要捞起2片蜡片，大标本组织捞1片蜡片即可。

切片捞起后，应注意立刻写上病理编号（漂片池中应避免出现两张以上的切片）。也有医院病理科使用的是玻片打号机，或直接在载玻片上粘贴编码贴签。

7.烤片：待玻片上水分充分晾干后，将玻片按照收片顺序收至染色架上，置入75°的恒温箱内烤片15-30min，使组织间隙的石蜡充分融化。

烤片时间应充足，尚未干燥的片子急促烤片，会使组织分散，也可能出现核内假包涵体，见右图

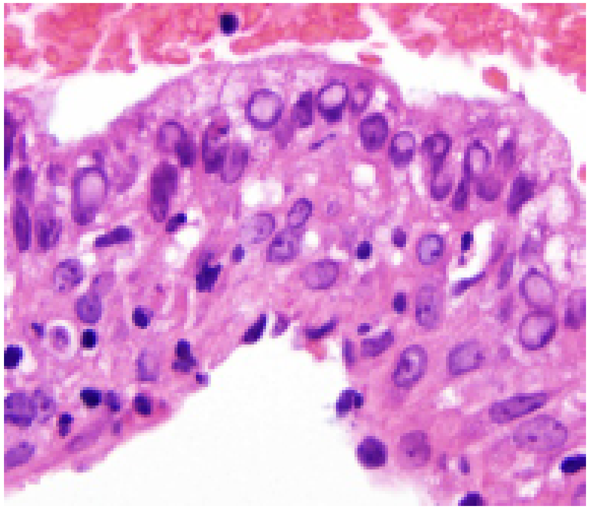

第二部分

常见“问题”HE切片及解决方法

➢ 一.组织固定不佳

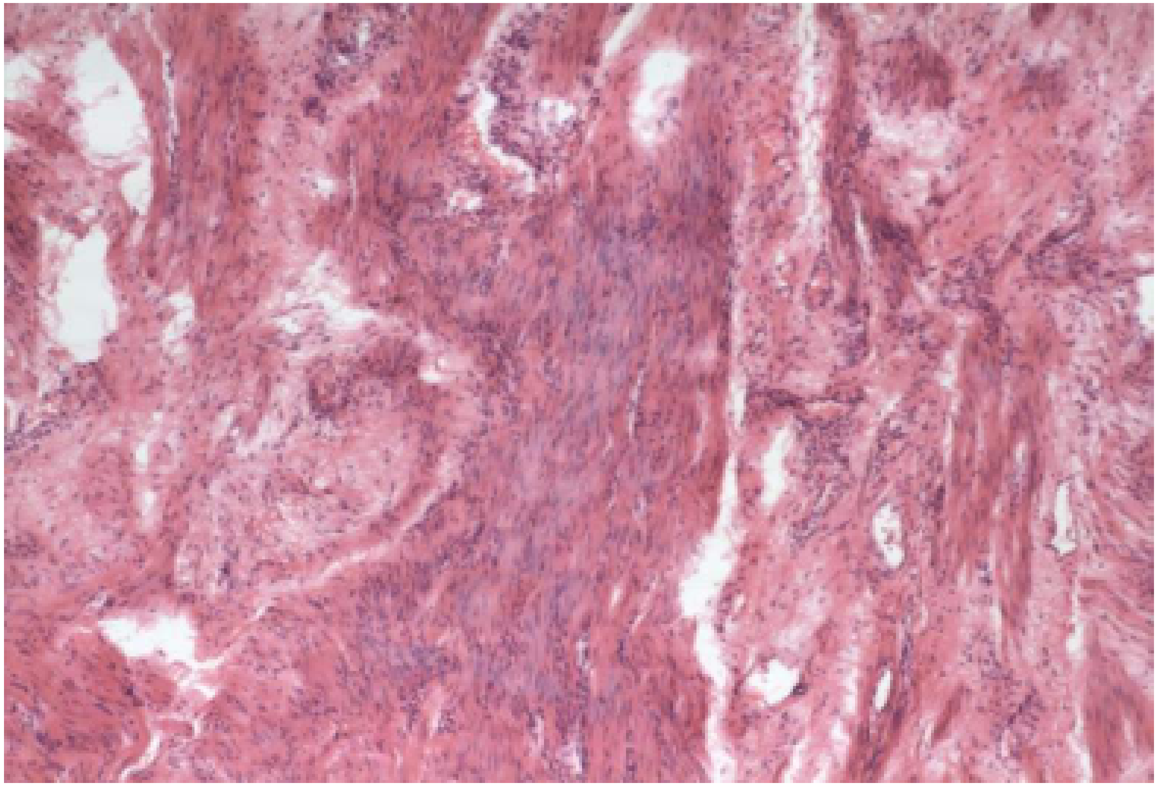

原因：未固定好的组织直接脱水，造成染色模糊不清

解决办法：切片重新脱蜡入水后，进入AFA固定液或丙酮液，固定5分钟，水洗后常规染色即可。

AFA固定液：850ml 95%Alc，100ml Formalin，50ml 醋酸。

➢ 二.切片空洞

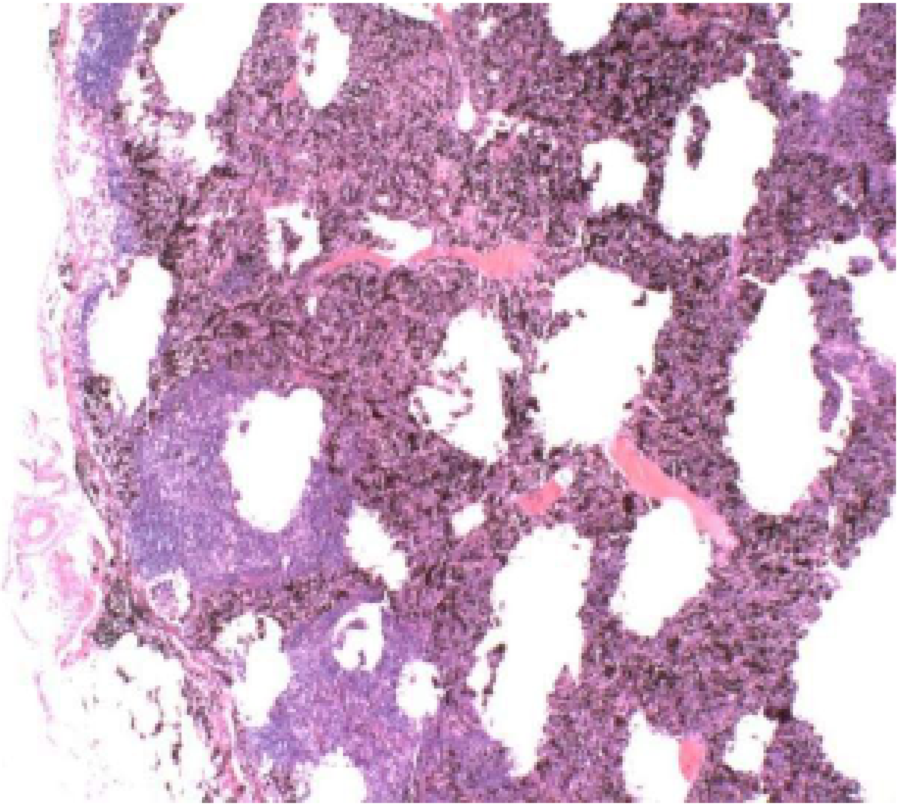

切片有空洞（筛网状）及破碎不齐的现象。

解决办法：多见于淋巴结及软组织，切片时慢进蜡块，轻削组织，提取切片前要轻轻转动数次至无空洞为止。

切片空洞

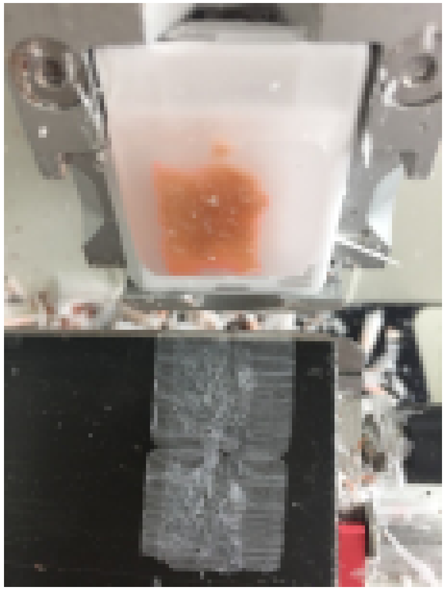

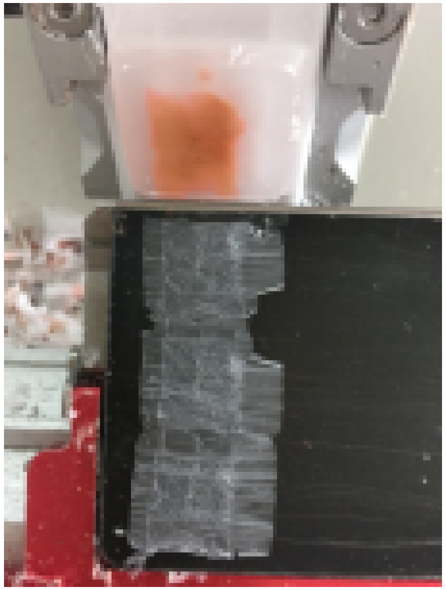

原因：粗修过快过猛，细修不充分。

解决方法：减少进刀幅度，缓慢粗修，细修到位后再留取切片。

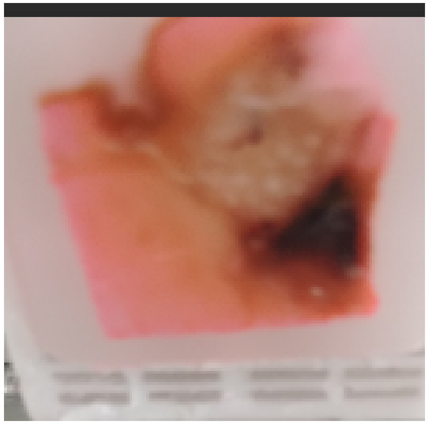

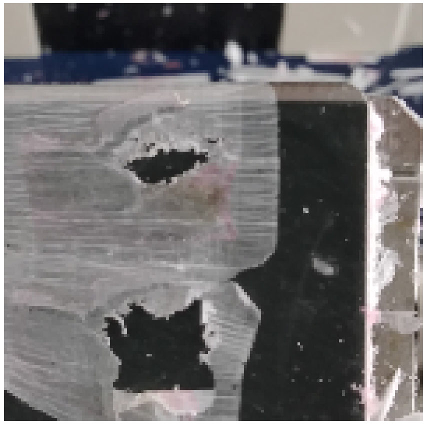

常见于：

1. 组织过硬，如骨组织，有钙化点等。补救方法：重新脱钙处理；

2. 组织中含有脂肪组织，切片困难。补救方法：调节切片厚度；

➢ 三.切片厚薄不均

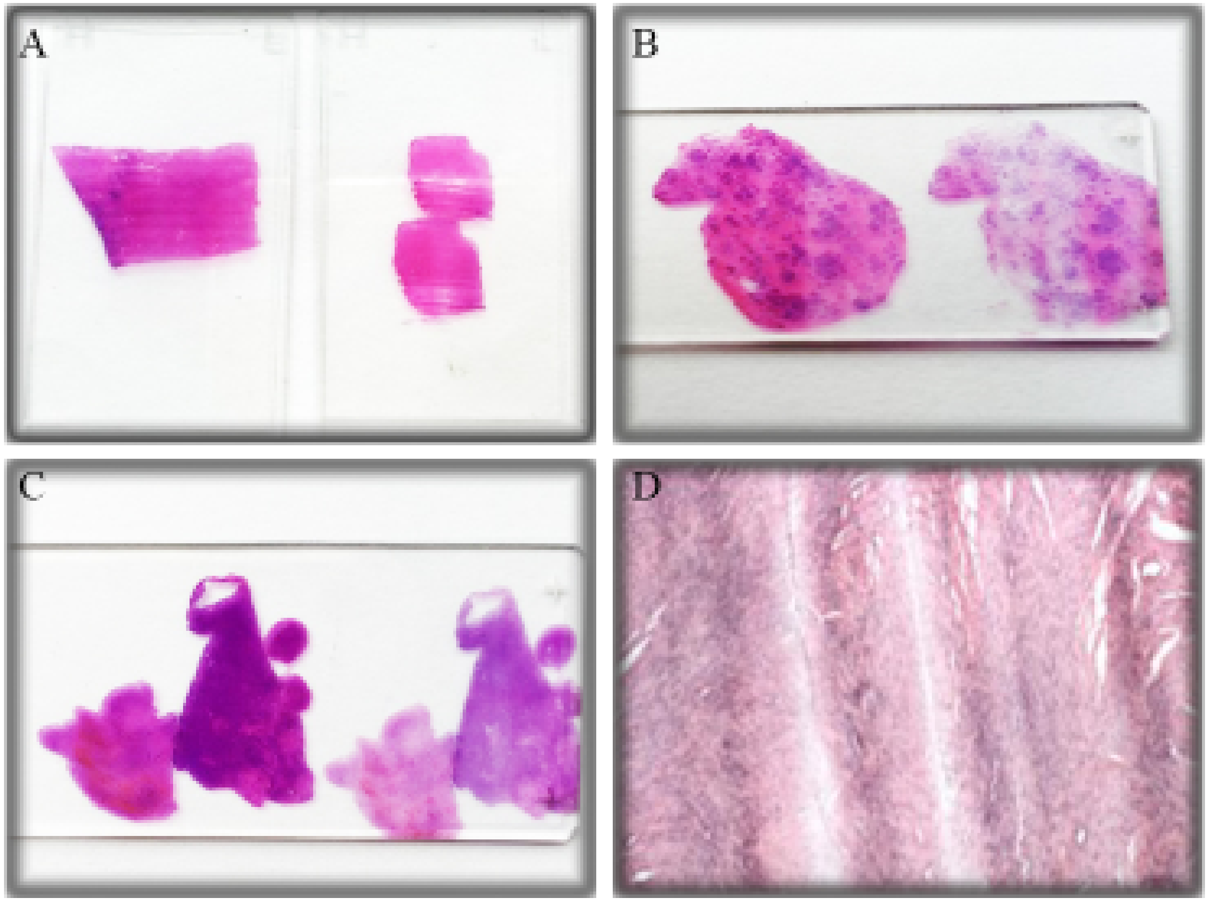

A-C:大体 D：镜下

三.切片厚薄不均

切片厚薄不均或有横纹，染色后呈“梯田”状。

原因：1.刀架损坏，刀片与刀座间有空隙；2. 压板与刀架间有异物；3.遇到较硬的组织（子宫等）切片过快，不匀速；4.包埋盒太小，夹头不牢。

解决方法：1.更换刀架；2.清理压板与刀架间的异物；3.遇到较硬组织（如子宫），可用手指轻抚蜡块使其复温，修块时应小幅度进刀，细修要充分，切片时注意缓慢均匀；4.将包埋盒横着放入夹头中进行切片。

四.组织切片皱褶

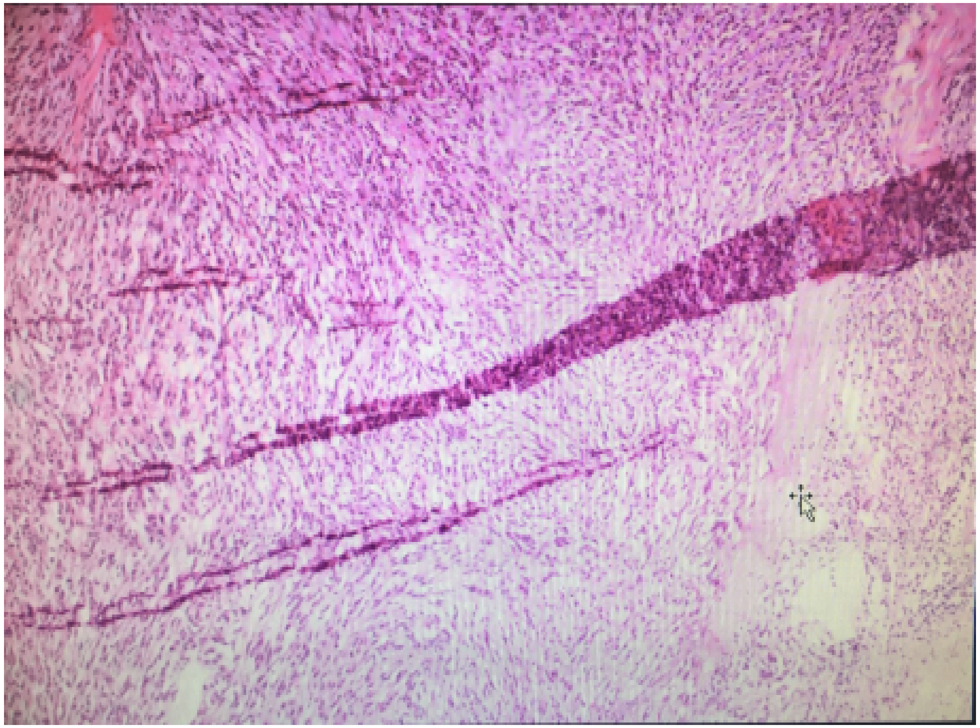

常见于1.蜡块不凉2.切片刀使用次数过多变钝3.切片刀边缘粘有石蜡致刀片不锋利4.包埋用石蜡过硬5.刀架弧度过大。

解决方法1.重新冷冻蜡块2.更换切片刀的位置，或重新更换切片刀。3.用湿纱布顺切片刀刀口方向，轻轻擦拭切片刀4.更换熔点较低的石蜡5.降低切片刀的弧度。6.适当增加漂片池的温度。

➢ 五.切片不完整-没有切全或者过切

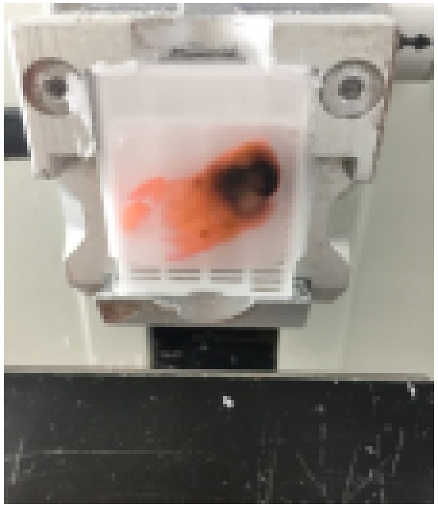
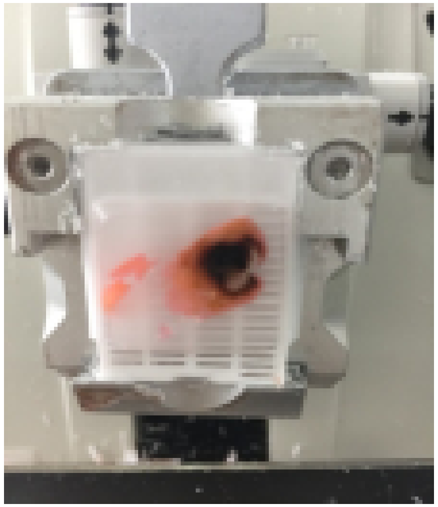
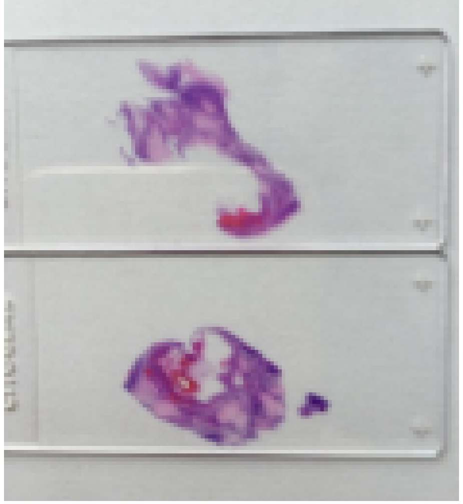

分析原因： 单块或多块包埋时，组织块或包埋盒底面不在一个水平面。

解决方法： 调节通用包埋盒夹头的角度进行切片，或者重新包埋。

➢ 六.切片不能形成完整蜡带

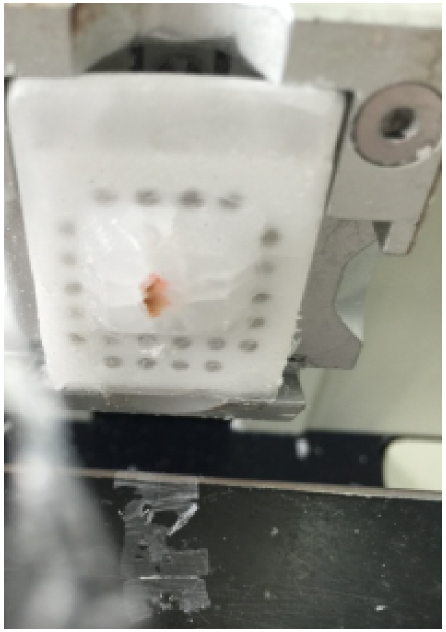

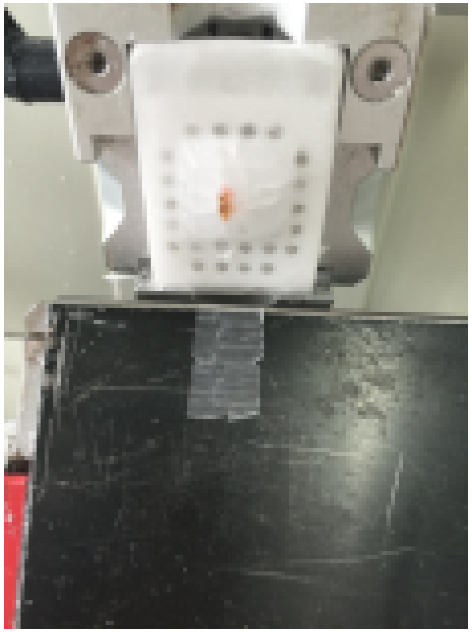

分析原因：蜡块不够凉；包埋石蜡太硬或太软。

解决方法：更换石蜡重新包埋。

➢ 七.切片挤压、堆积在一起

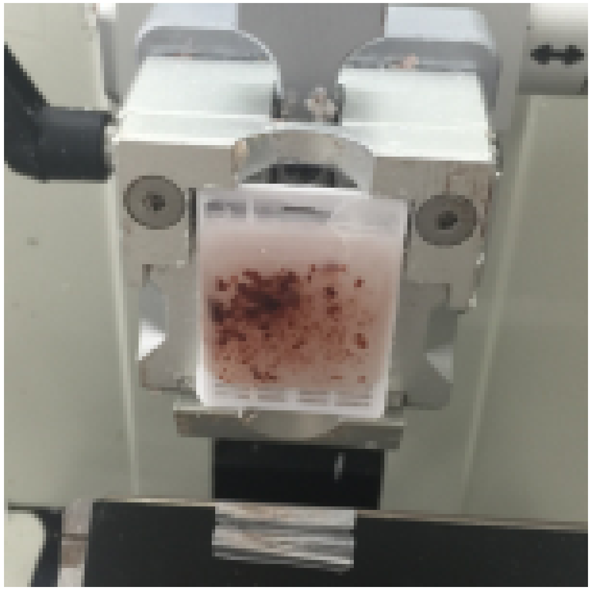

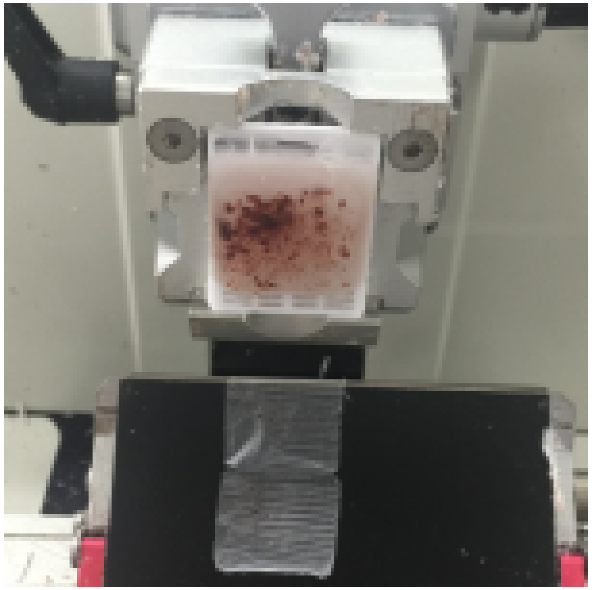

分析原因：蜡块不够凉；包埋石蜡粘度大；静电。

解决方法：切片时尽量薄切，切片时使用加湿器。

厚德、博學、精業、創新

➢ 八.蜡带弯曲呈扇形

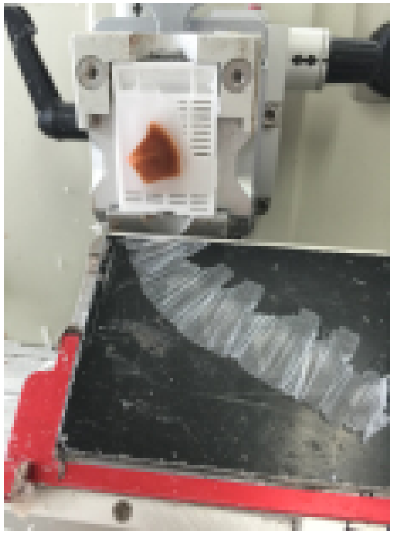

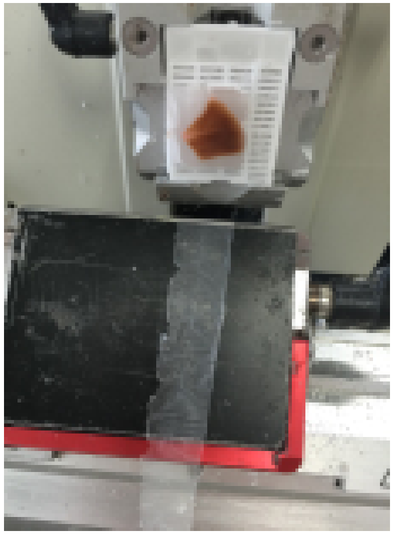

分析原因：1、蜡块不规则；2、切片刀左右两侧锋利程度不同，一侧锋利，另一侧过钝。

解决方法：1、更换刀位，修理蜡块，重新切片；2、更换新的切片刀。

九.切片污染

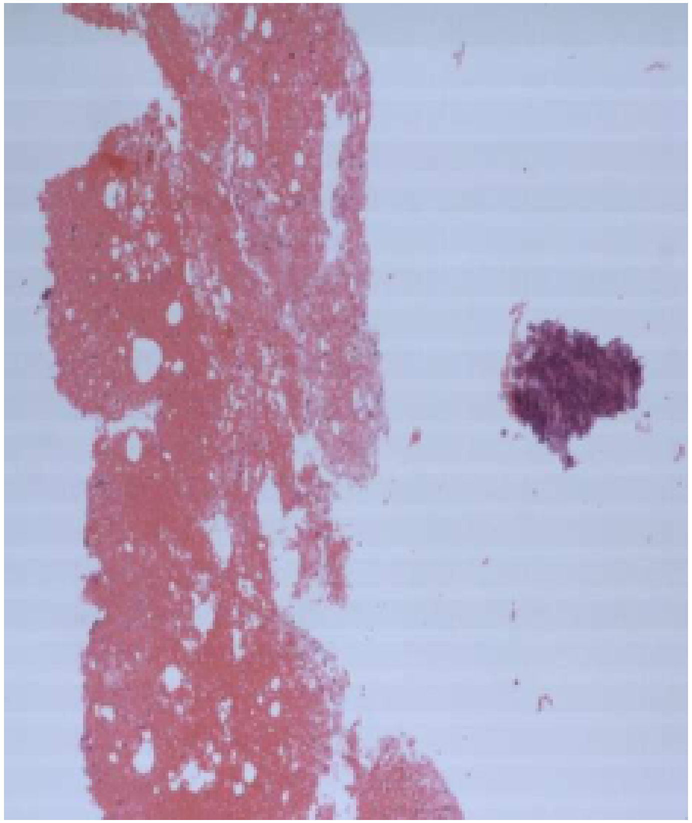

常见于

1.组织碎屑污染：在切片中，蜡带粘上其他组织的碎屑捞在了切片上。补救方法：细修切片时用刷子清扫干净刀台。

2.水面污染：水面不洁净，捞片时将肉眼难以看见的碎组织渣捞在了切片上。补救方法：用拉水纸经常清洁水面，保证漂片水池的干净。

3.烤片时的污染：遇有灰尘落到组织面，或者刷子清扫蜡带力度过大，“飞”到烤片上。补救方法：刷子清扫时，注意力度轻柔，遇到肉眼可见的污染，及时重新切片。

4.染色液和试剂的污染：染色液中的色素颗粒和试剂沉渣，在染色时吸附在切片上。补救方法：经常过滤染色液及更换试剂。

➢ 十.切片上卷不能形成蜡带

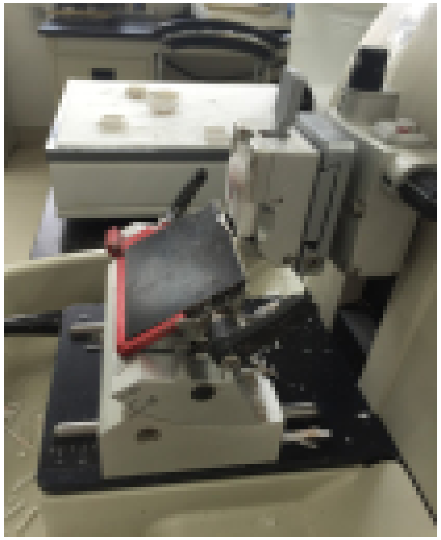

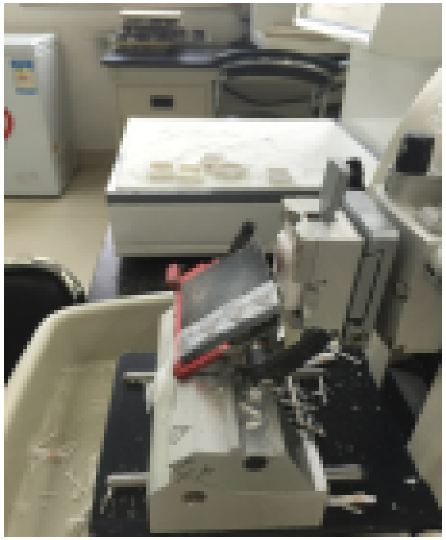

分析原因：1.切片刀不锋利；2.切片刀的角度过大；3.切片过厚；4.蜡块不够凉。

解决方法：1.更换新刀片；2.调整切片刀的角度(5度)；3.切片的厚度一般为3-5μm，视具体组织略有不同：肾脏1-2μm，淋巴结2-3μm，脑4-5μm，脂肪组织6-7μm；4.增加蜡块冷冻的时间。

➢ 十一.黏片

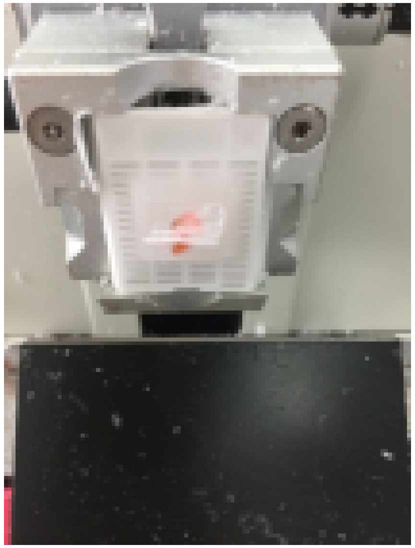

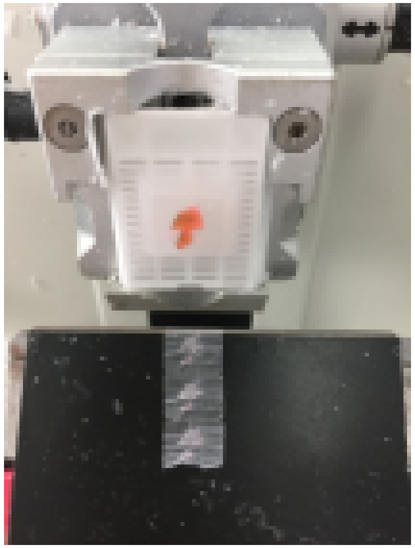

分析原因：室温过高或空气干燥，切片时静电增加，切片与切片刀容易吸附，造成黏片不能形成蜡带。

解决方法：切片时，如果有条件可以使用加湿器；重新冷冻蜡块。

➢ 十二.组织卷边

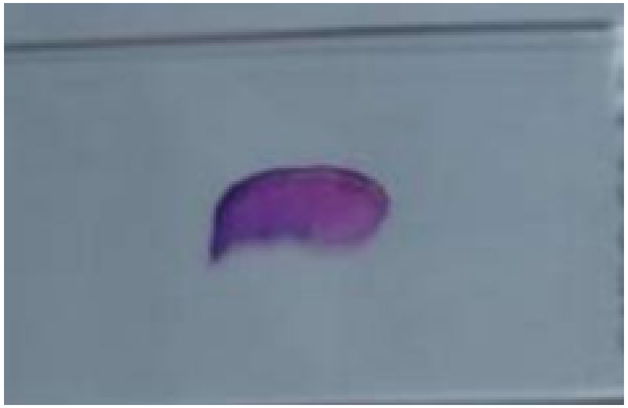

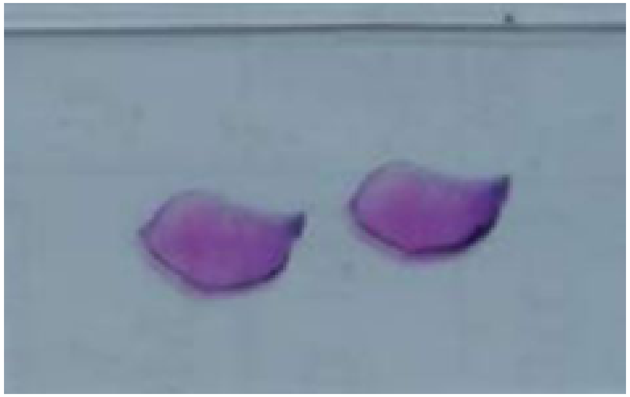

分析原因：常见于较硬的子宫、前列腺及脱钙不良的组织。

解决方案：捞片时用防脱片或蛋清甘油涂片，控干水分后再烤片，染色时注意冲水力度勿太大。

优质切片必须做到：

一 切片组织完整

二 切片厚薄均匀

三 切片染色对比清晰；红、蓝分明

四 切片无刀痕、无裂隙、无颤痕

五 切片平坦、无皱折、无折叠

六 切片无污染

七 切片封胶适中、无气泡、透明度好

八 切片无松散、裱贴位置适当

九 切片整洁、标签端正牢固

正常切片

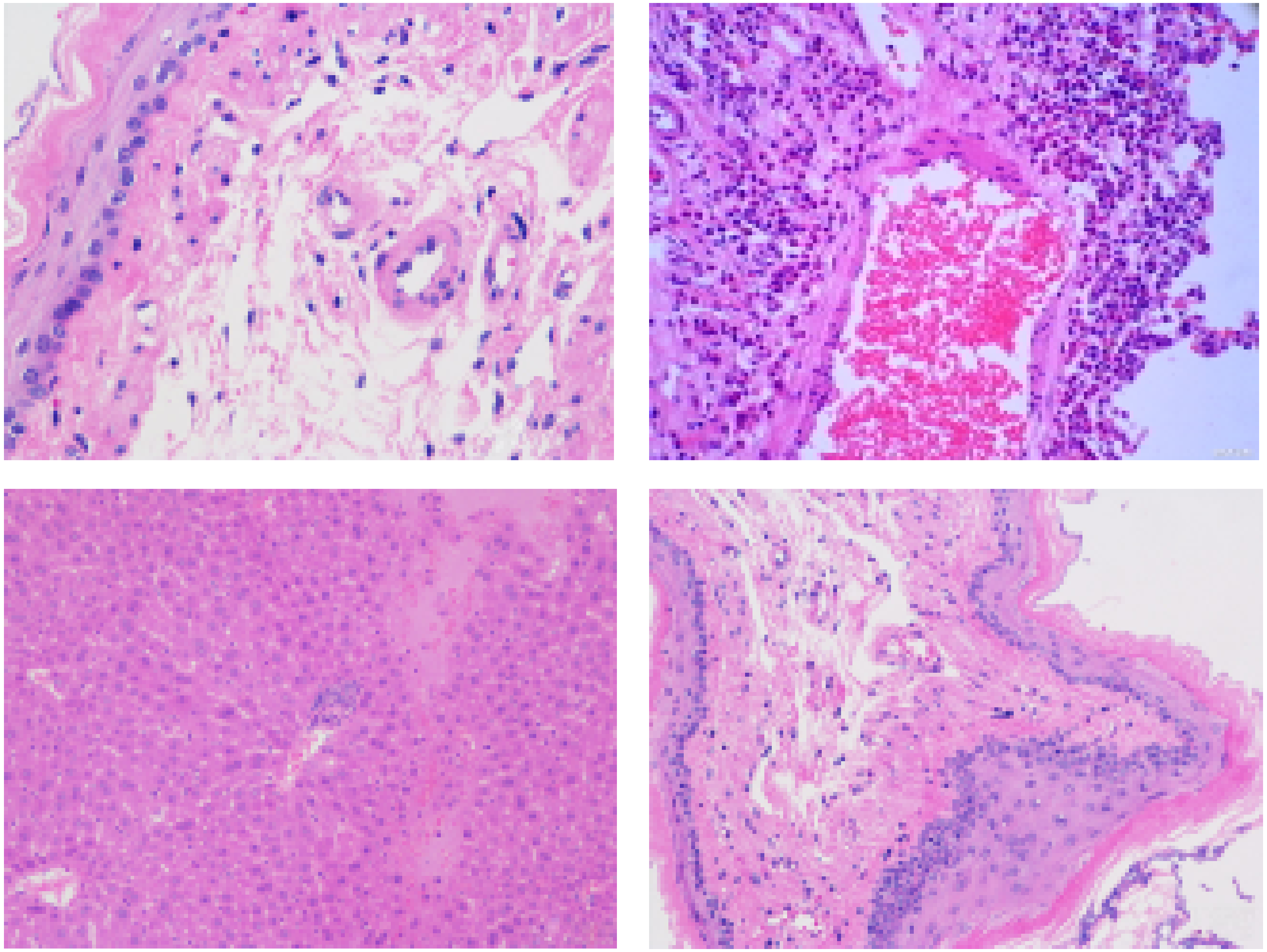

THANK YOU

郑州大学第一附属医院
The First Affiliated Hospital of Zhengzhou University

常规病理组织石蜡包埋技术

郑州大学第一附属医院病理科

Paraffin embedding technique for pathological tissues

郑州大学第一附属医院
The First Affiliated Hospital of Zhengzhou University

一 包埋的定义

二 包埋的工具及准备事项

三 包埋的操作流程

四 包埋的注意事项

五 包埋的常见问题分析

六 包埋机的维护

一 包埋的定义

二 包埋的工具及准备事项

三 包埋的操作流程

四 包埋的注意事项

五 包埋的常见问题分析

六 包埋机的维护

包埋的定义

包埋定义：

组织块经过固定、脱水、透明和浸透剂浸透，用包埋剂包成块的过程称为包埋。

经包埋后，可使组织达到一定的硬度和韧度，有利于切成薄片。

不同的组织，需要不同的包埋剂和不同的包埋方法。

包埋剂的种类：

石蜡、树脂、塑料、火棉胶等。

我们常规技术室用的包埋剂主要是石蜡。

一 包埋的定义

二 包埋的工具及准备事项

三 包埋的操作流程

四 包埋的注意事项

五 包埋的常见问题分析

六 包埋机的维护

包埋的工具及准备事项

常用包埋机：

- 樱花包埋机
- 莱卡包埋机
- 中威包埋机

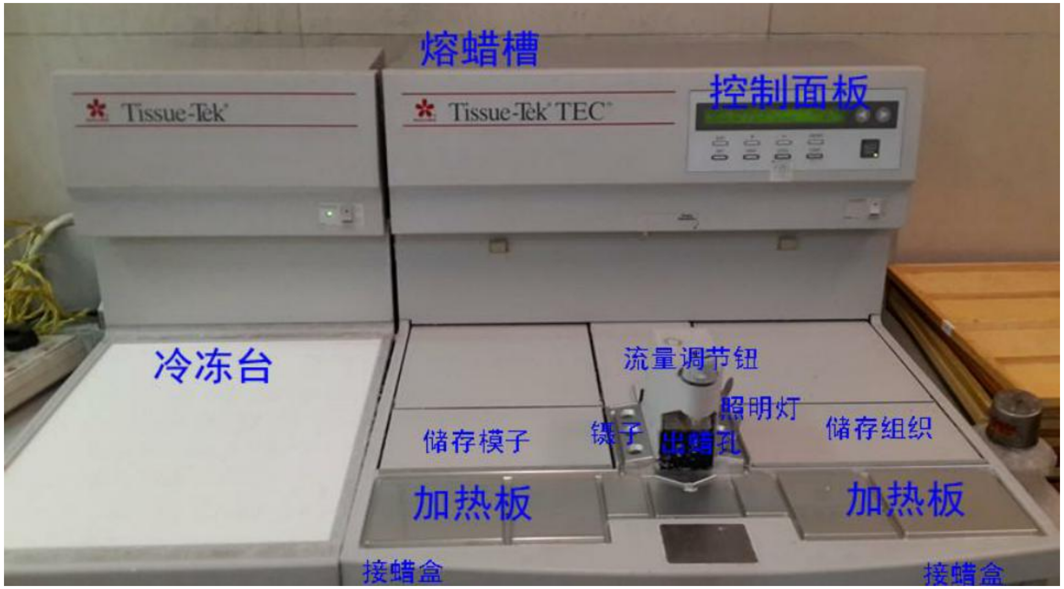

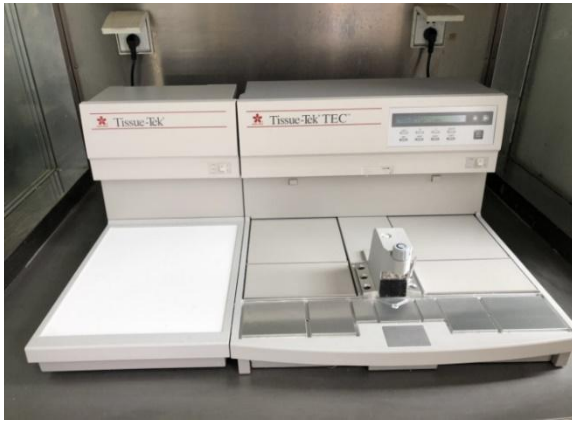

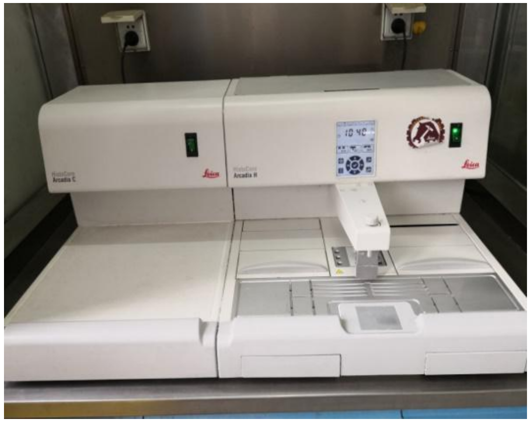

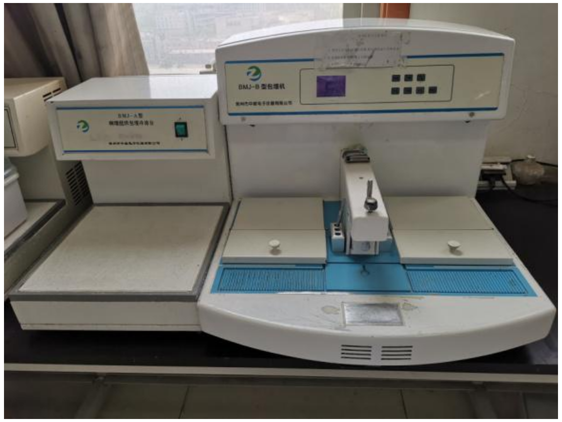

郑州大学第一附属医院
The First Affiliated Hospital of Zhengzhou University

包埋的工具及准备事项

包埋石蜡：

- 包埋所用的石蜡要与脱水机中浸蜡的石蜡保持一致
- 包埋用石蜡的熔点应58℃~62℃之间
- 石蜡要保证高纯度，以防造成标本的污染或刀片的损坏

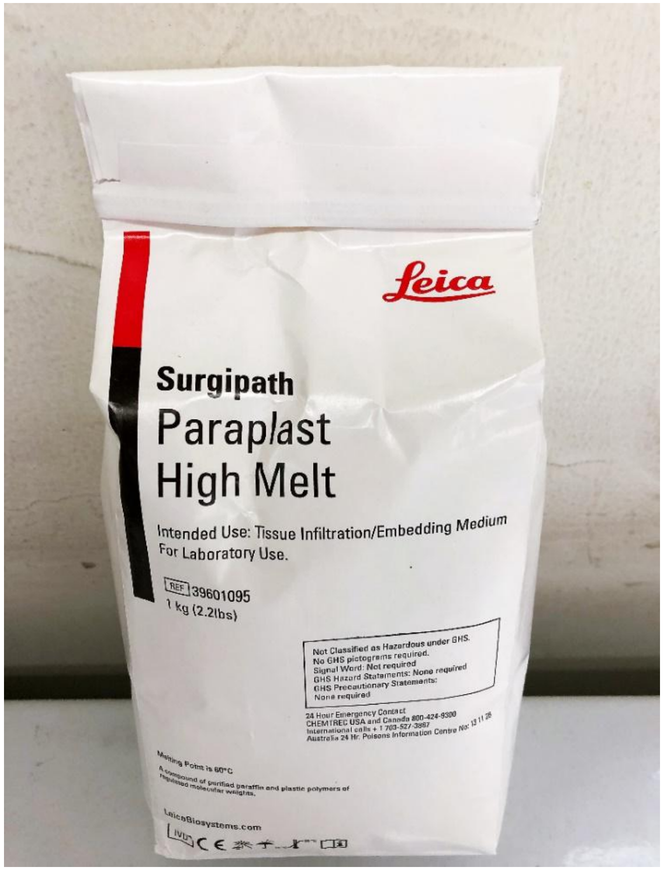

郑州大学第一附属医院
The First Affiliated Hospital of Zhengzhou University

包埋的工具及准备事项

包埋盒：

- 按颜色分为红、绿、白、黄四种。
- 取材时，根据组织类型，选择对应颜色的包埋盒。

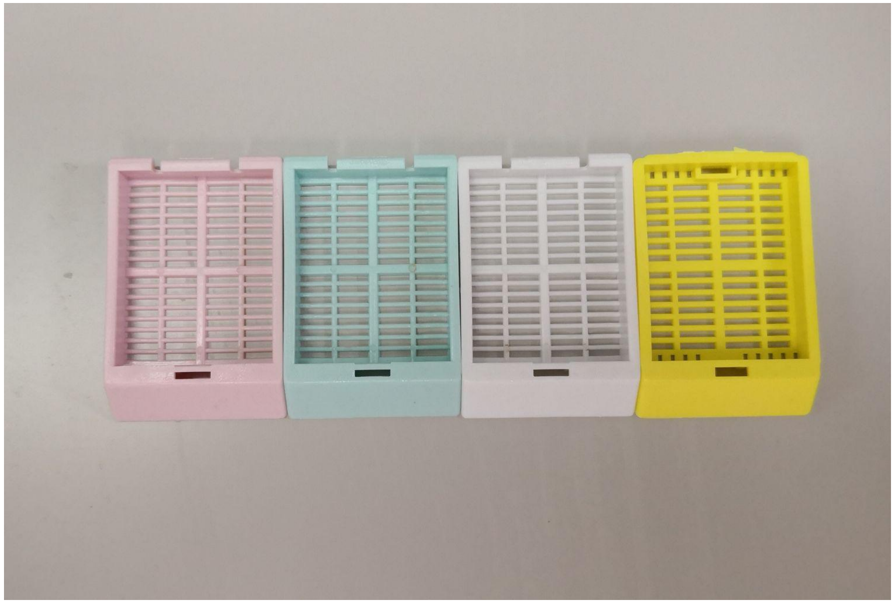

- 红-门诊小标本
- 绿-病房小标本
- 白-病房大标本
- 黄-冰冻余组织

包埋的工具及准备事项

包埋模具：

按型号分为大、中、小三种。

包埋工具：

包埋镊、酒精灯、打火机、废料盒等。

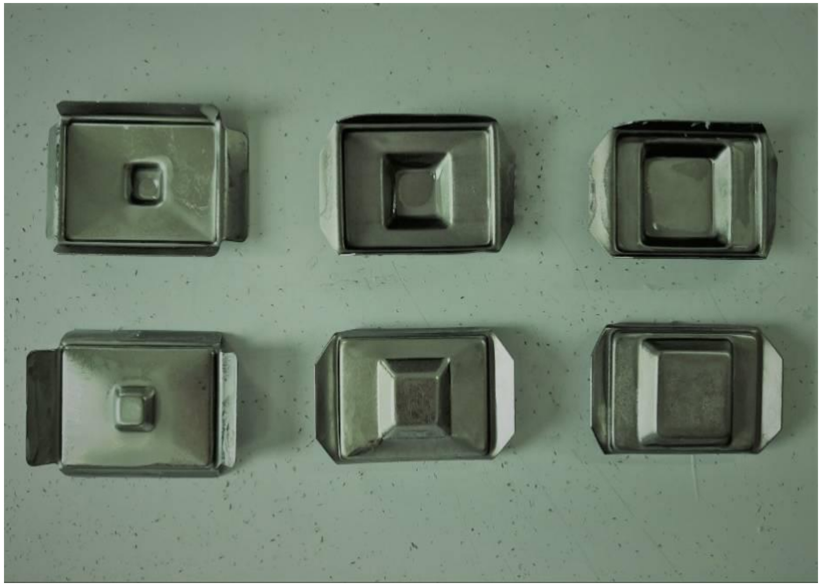

7mm*7mm　15mm*15mm　20mm*20mm

郑州大学第一附属医院
The First Affiliated Hospital of Zhengzhou University

一 包埋的定义

二 包埋的工具及准备事项

三 **包埋的操作流程**

四 包埋的注意事项

五 包埋的常见问题分析

六 包埋机的维护

包埋的操作流程

- 第一步，也是最重要的一步，就是认真核对。保证准确无误后，再进行下一步工作。

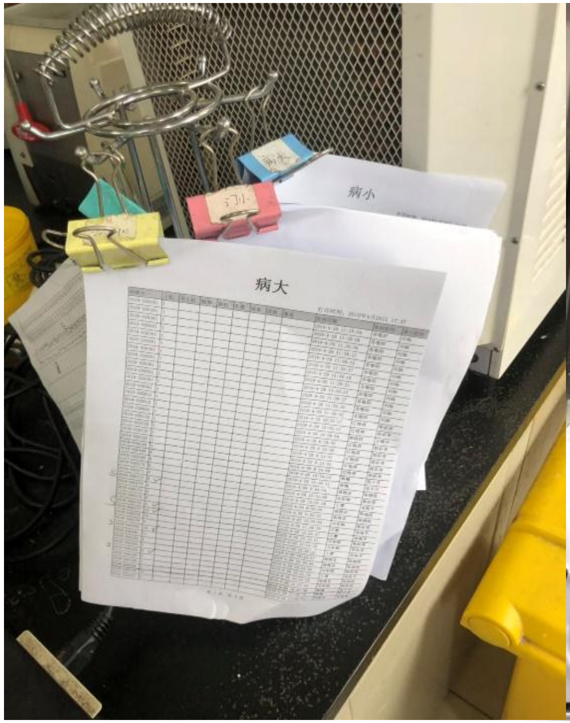

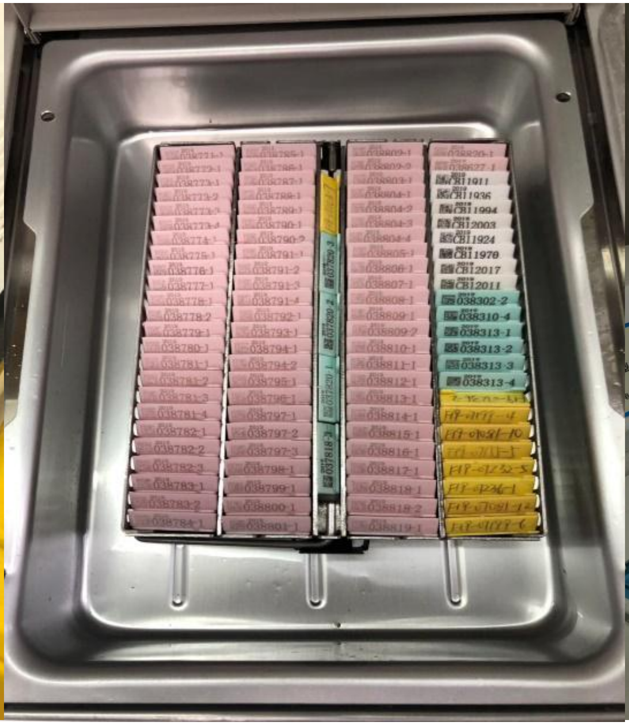
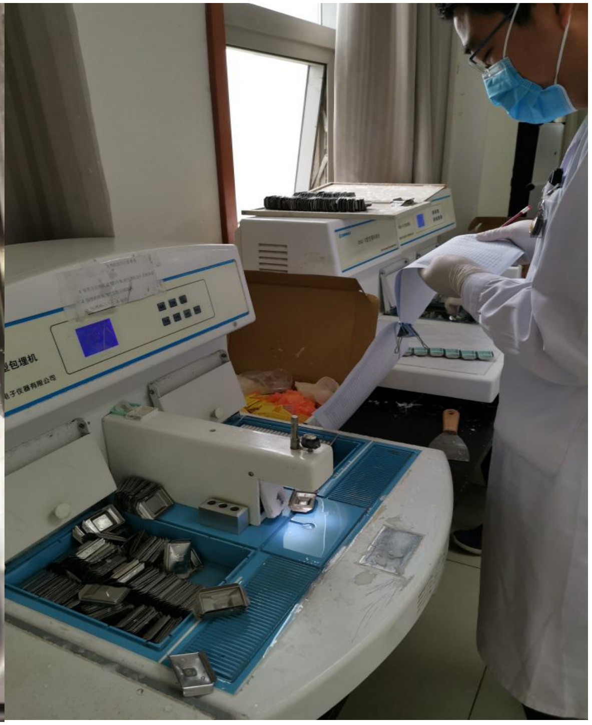

郑州大学第一附属医院
The First Affiliated Hospital of Zhengzhou University

包埋的操作流程

- 第二步，从经过脱水的组织筐中取出一个包埋盒，打开包埋盒，显露出其中的组织。

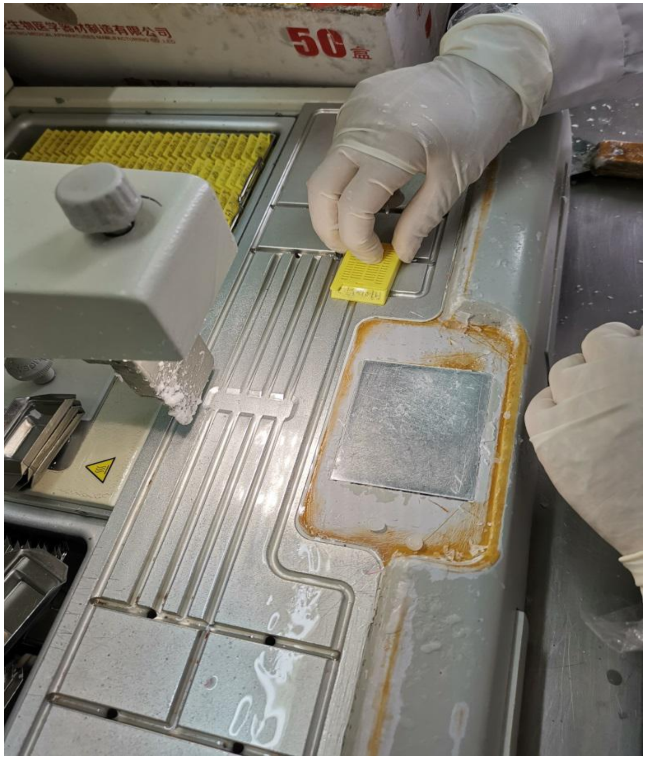

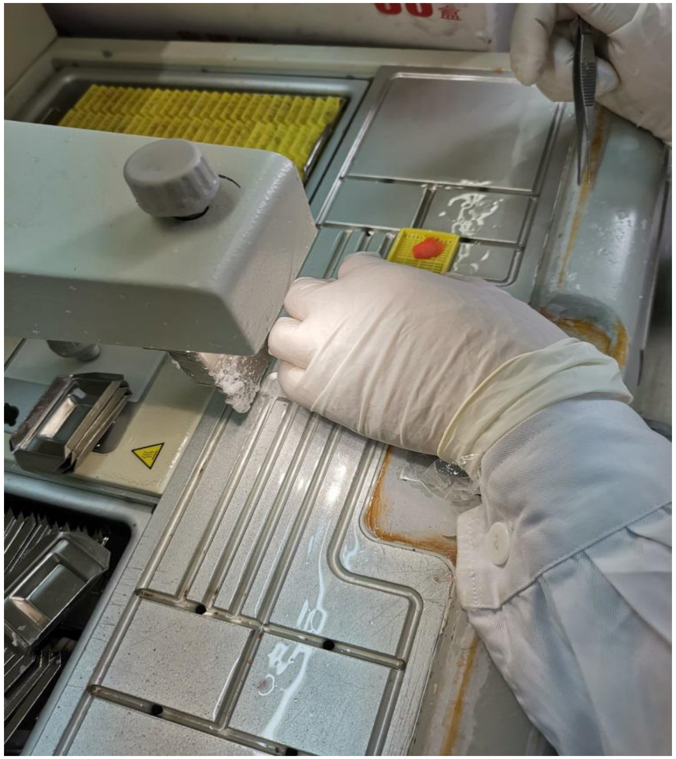

包埋的操作流程

- 第三步，根据组织块大小，选择合适的包埋模具。把模具放在出蜡口下方热台上，将蜡液注入模具，液面刚好与模具的上缘平齐。

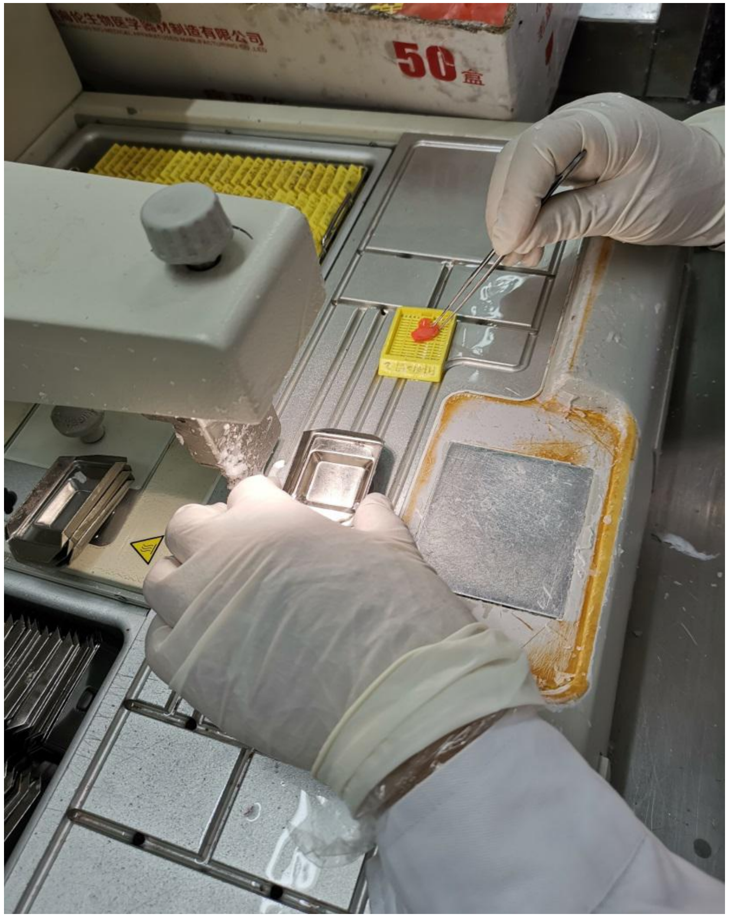

The First Affiliated Hospital of Zhengzhou University
郑州大学第一附属医院

包埋的操作流程

- 第四步，用干净的热的镊子，将包埋盒内所有的组织，全部放入模具的底部。

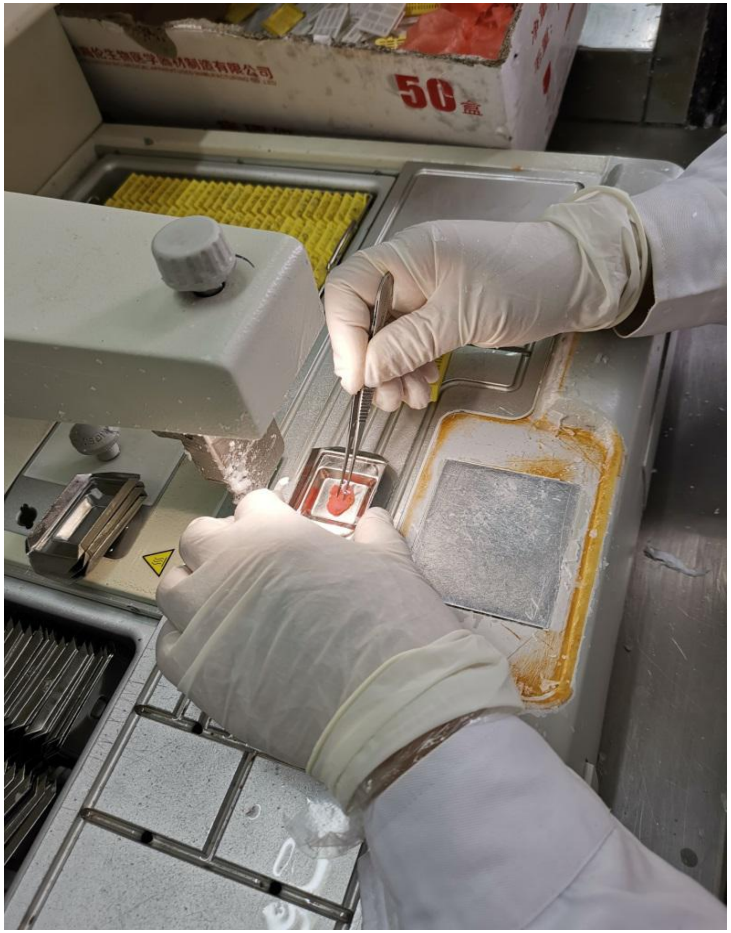

包埋的操作流程

- 第五步，待组织与模具内的蜡液充分融合后，移动模具至小冷台，用镊子轻压组织数秒，直至底部石蜡凝固。

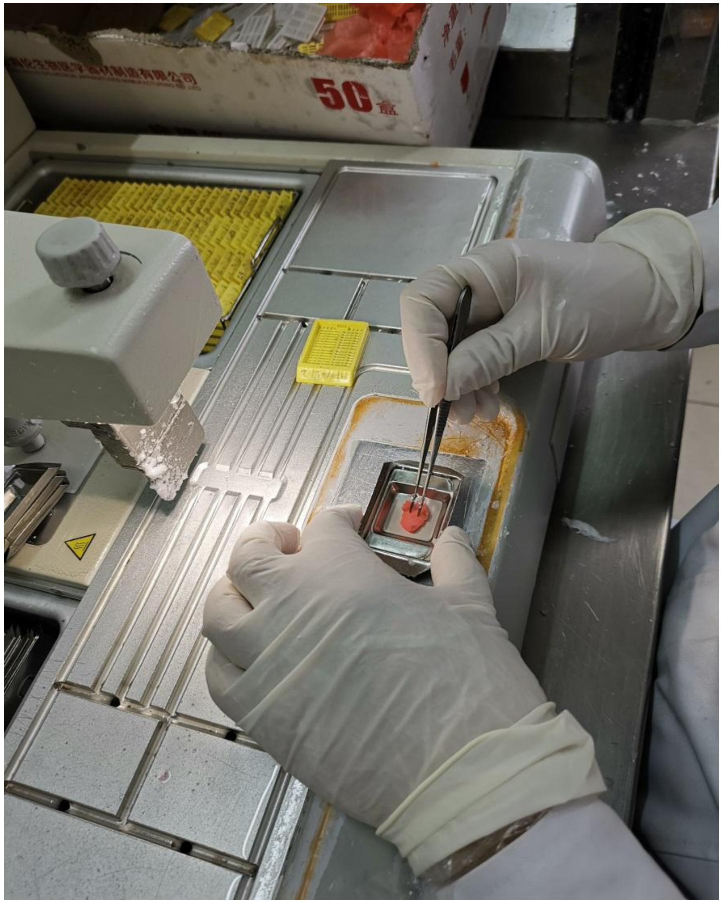

包埋的操作流程

- 第六步，盖包埋盒，应沿一侧贴边缓慢盖上。并二次注入少许蜡液，要从右上角缓慢注入。

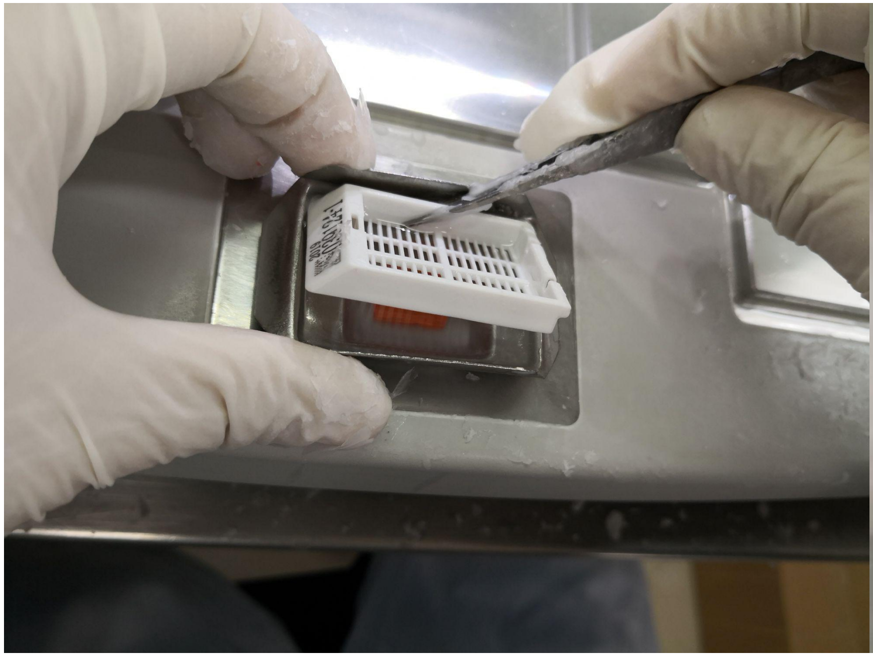

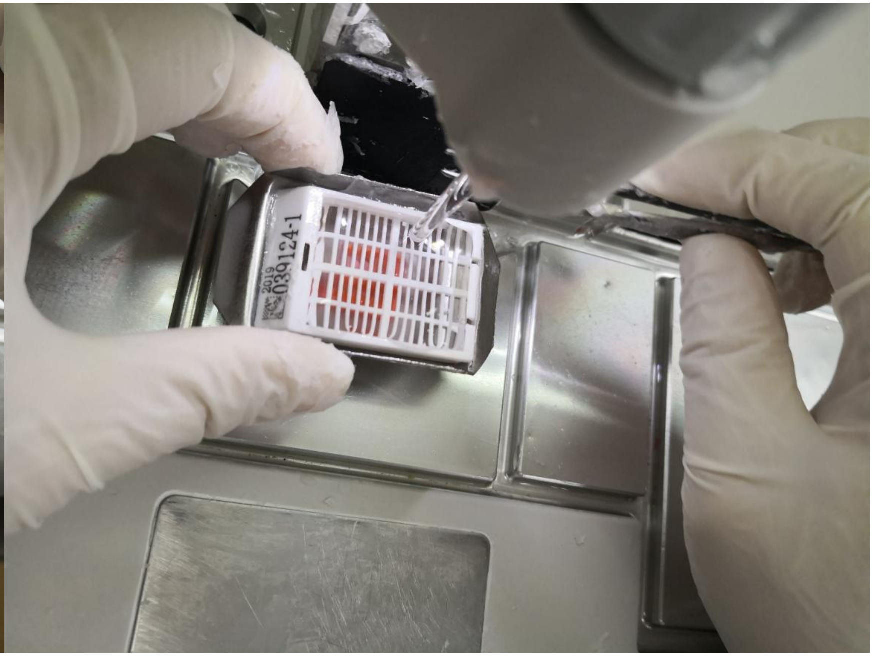

包埋的操作流程

- 第七步，将模具平稳移至冷冻台，按顺序摆放整齐。

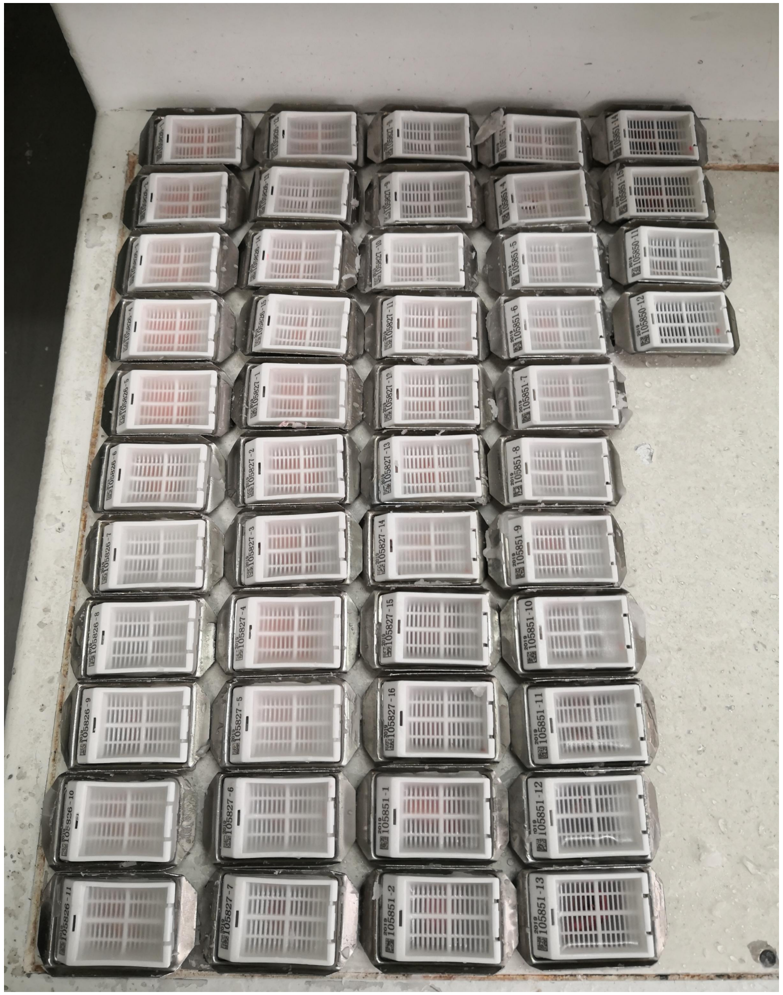

包埋的操作流程

- 第八步，待冷冻台上蜡块完全冷却，从模具中取出蜡块，进入下一个程序---切片。整个包埋操作流程结束。

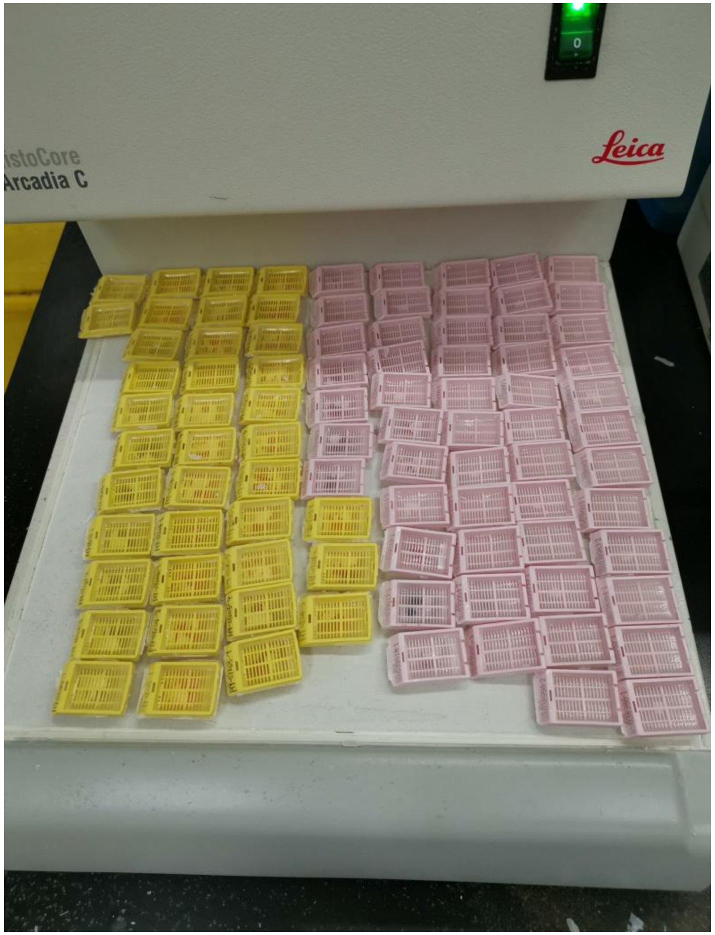

一 包埋的定义

二 包埋的工具及准备事项

三 包埋的操作流程

四 包埋的注意事项

五 包埋的常见问题分析

六 包埋机的维护

包埋的注意事项

- 包埋时，从包埋机右侧槽中拿出**一个**包埋盒，进行包埋，包埋完后，再从右边槽里拿下一个包埋盒。

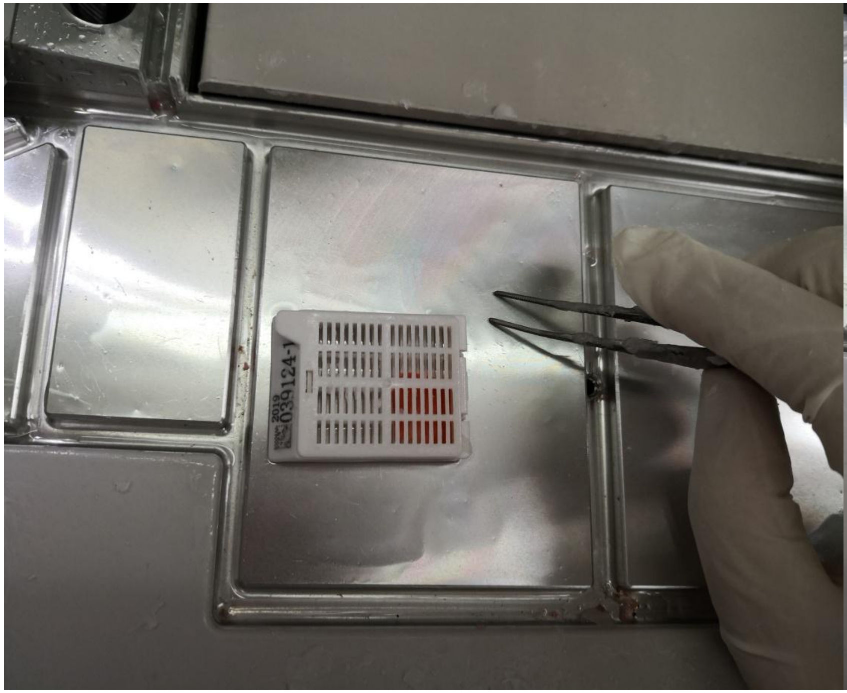

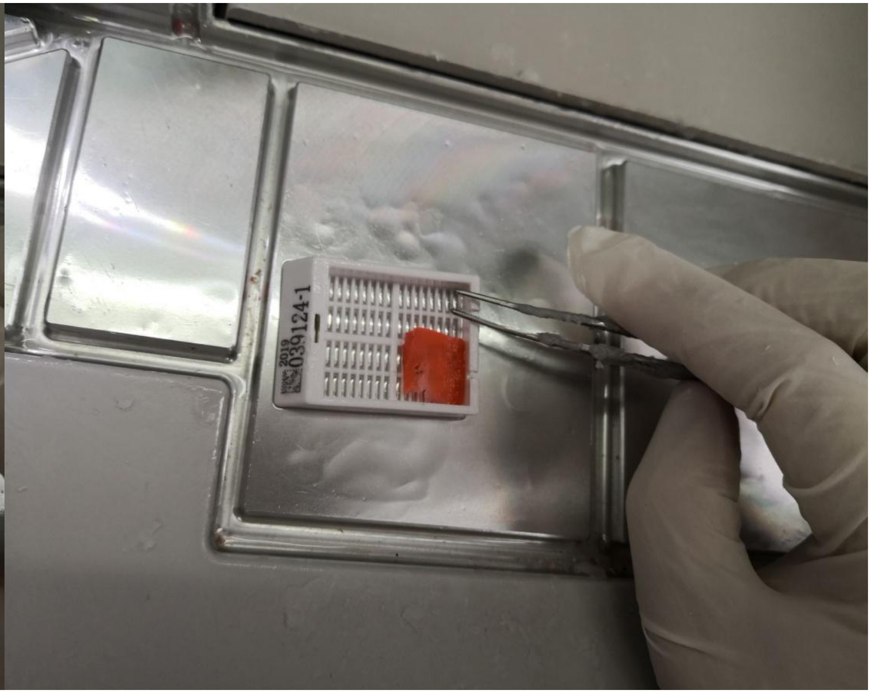

包埋的注意事项

- 管腔、囊壁、皮肤、脾脏等组织，应立埋。
- 如有多块小颗粒组织，应保证包埋在一个平面上。

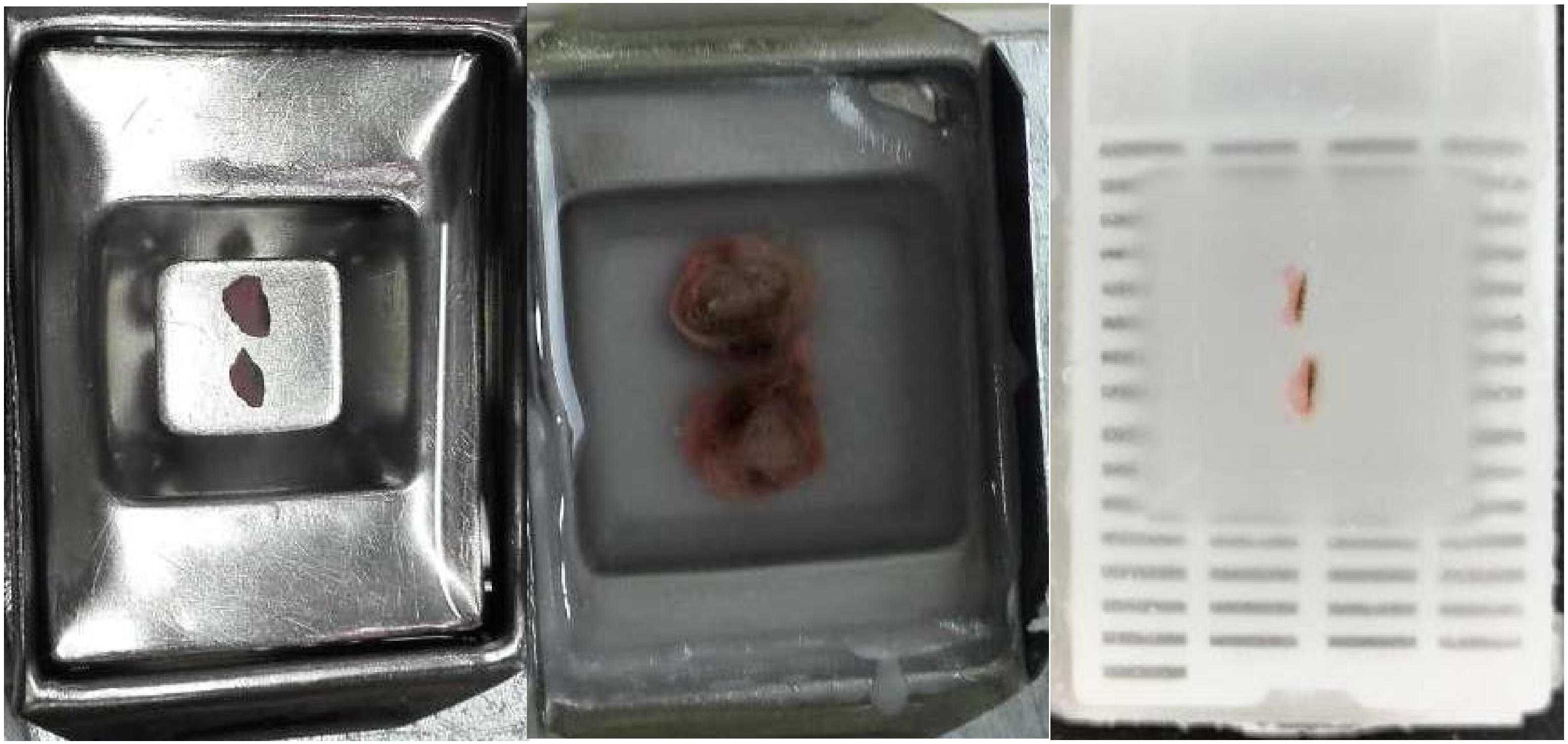

包埋的注意事项

- 胃黏膜活检标本应采用窄面立埋的方式。

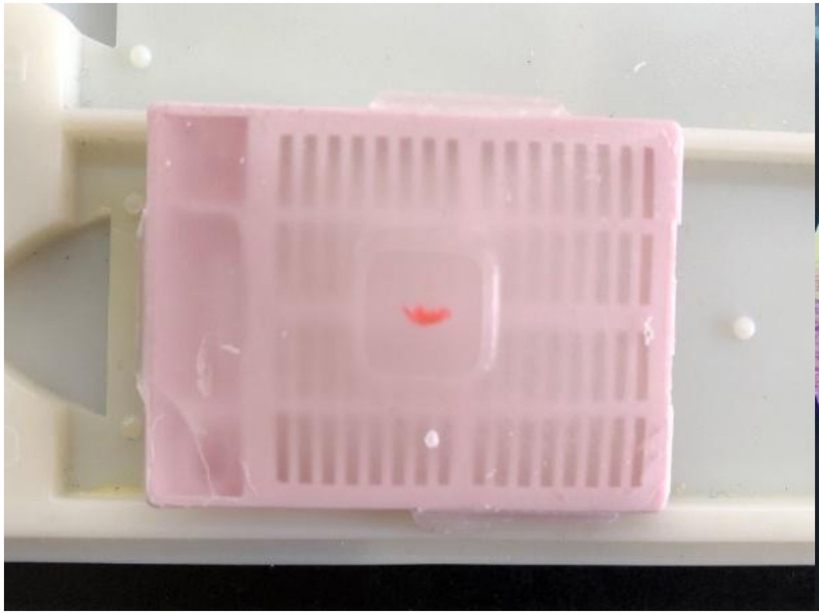

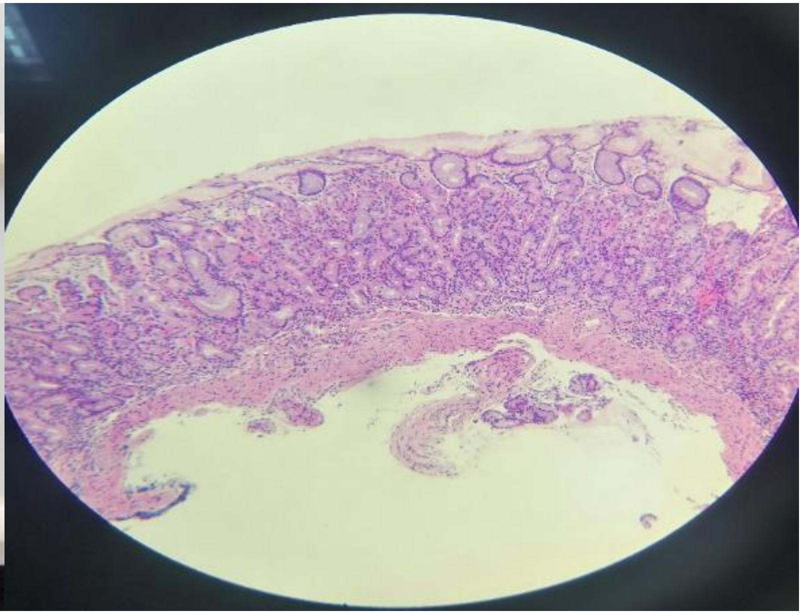

郑州大学第一附属医院
The First Affiliated Hospital of Zhengzhou University

包埋的注意事项

- 乳腺旋切标本组织易聚集成团，包埋时需注意展开每一条组织。
- 遇到冰余组织时，要观察组织已切过的面，使已切过的面朝下包埋，可保证冰冻片子与常规片子的统一性。

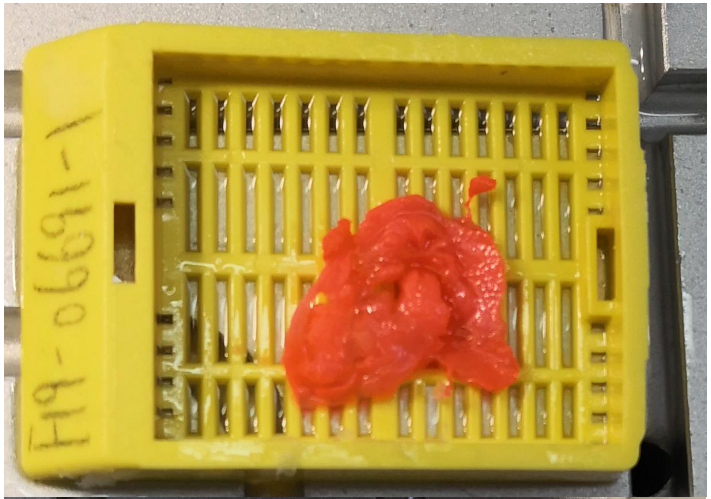

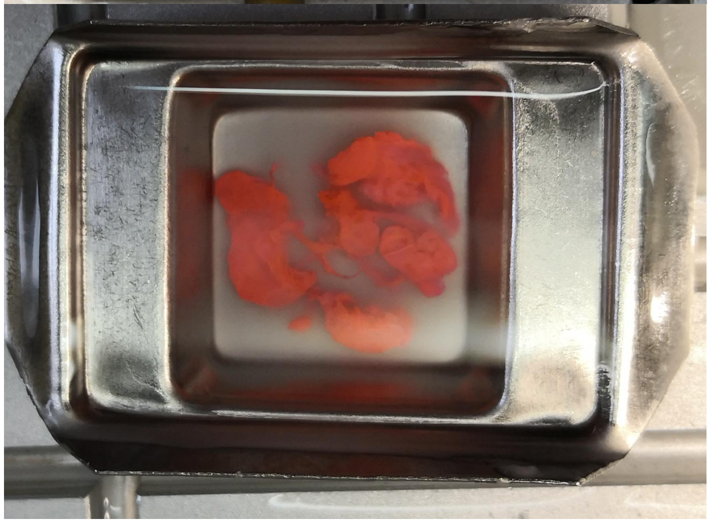

包理的注意事项

- 组织里有异物时一定要清理掉，否则会对切片造成很大影响，难以切出完整片子。
- 对于难于处理的，我们应联系取材医师。

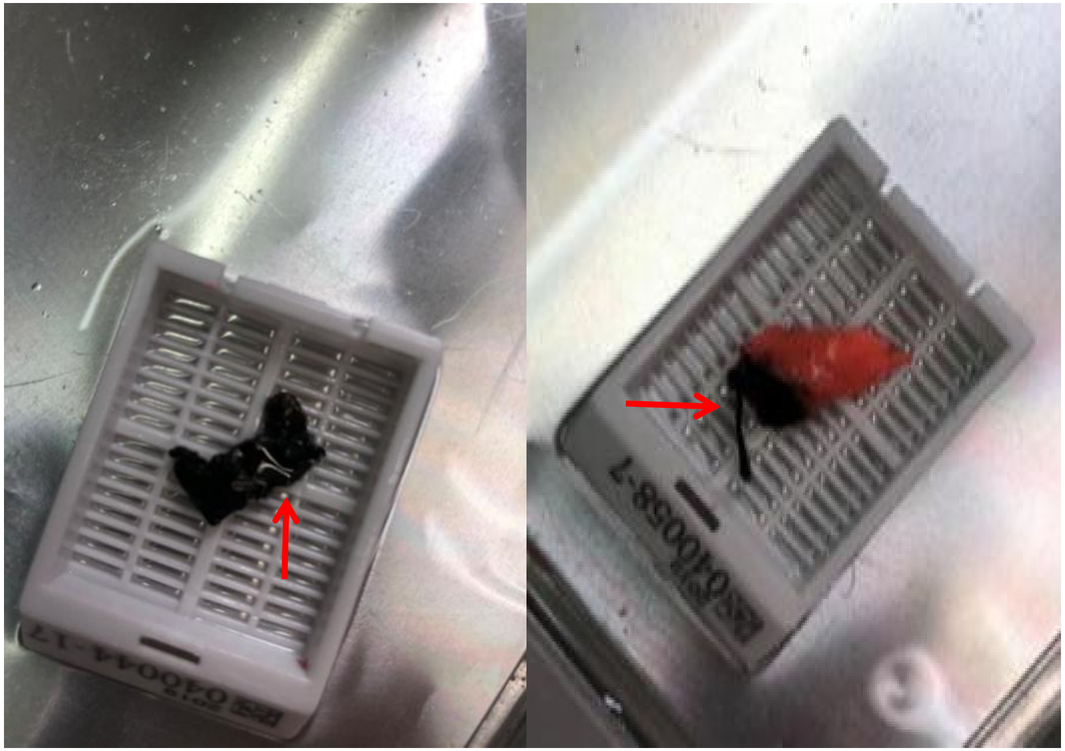

包埋的注意事项

- 包埋块取下模具后，侧面观看，组织蜡块与包埋盒平行。

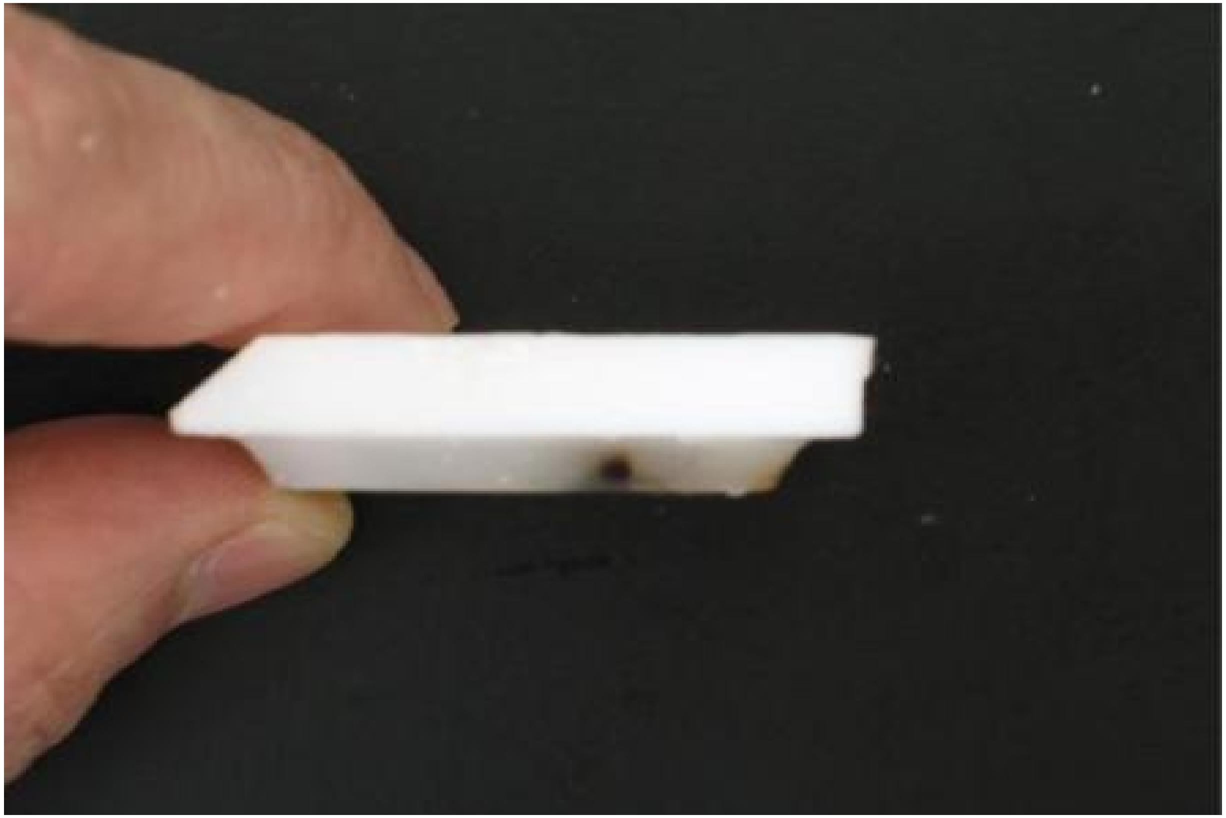

一 包埋的定义

二 包埋的工具及准备事项

三 包埋的操作流程

四 包埋的注意事项

五 **包埋的常见问题分析**

六 包埋机的维护

包埋的常见问题分析

拿出多个组织包埋：

- 如图，技术员拿出好几个包埋盒，并且打开好几个包埋盒，这是错误的。
- 这样很容易造成包埋时，组织放错盒子，以及盒中组织的丢失。还可造成组织过冷，与蜡液不能很好的融合。

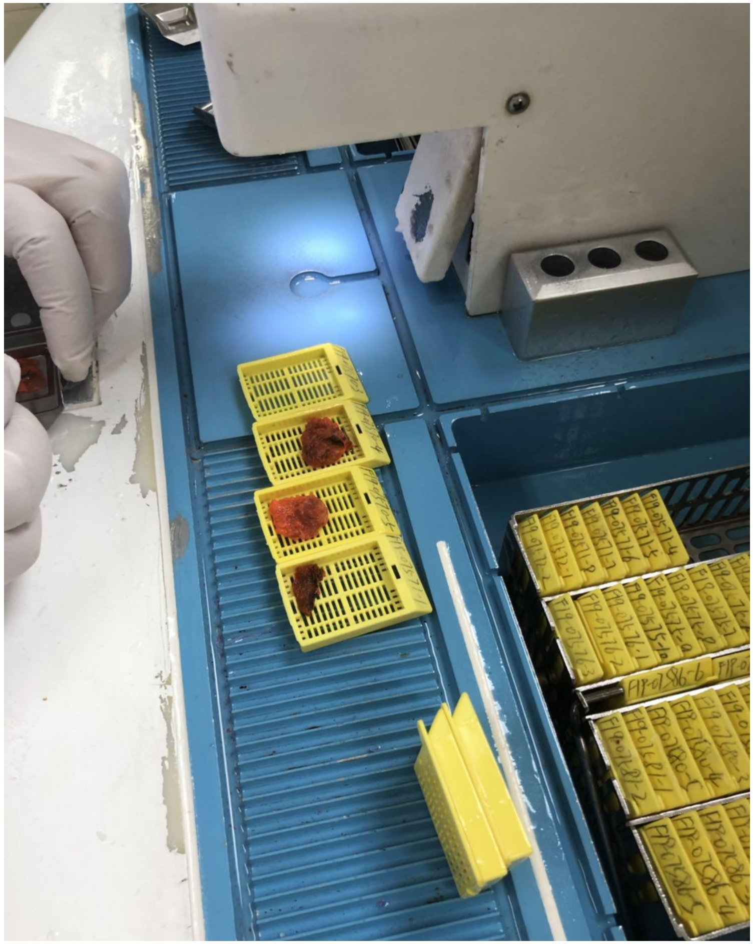

包埋的常见问题分析

组织蜡块与包埋盒不平行：

- 模具没有提前预热，内外有很多蜡，使包埋盒放不平整。
- 包埋时动作过慢，蜡凝固后加盖包埋盒。
- 冷冻台上结冰，台面凹凸不平，模具倾斜，导致蜡液流出，包埋盒随之侧滑。

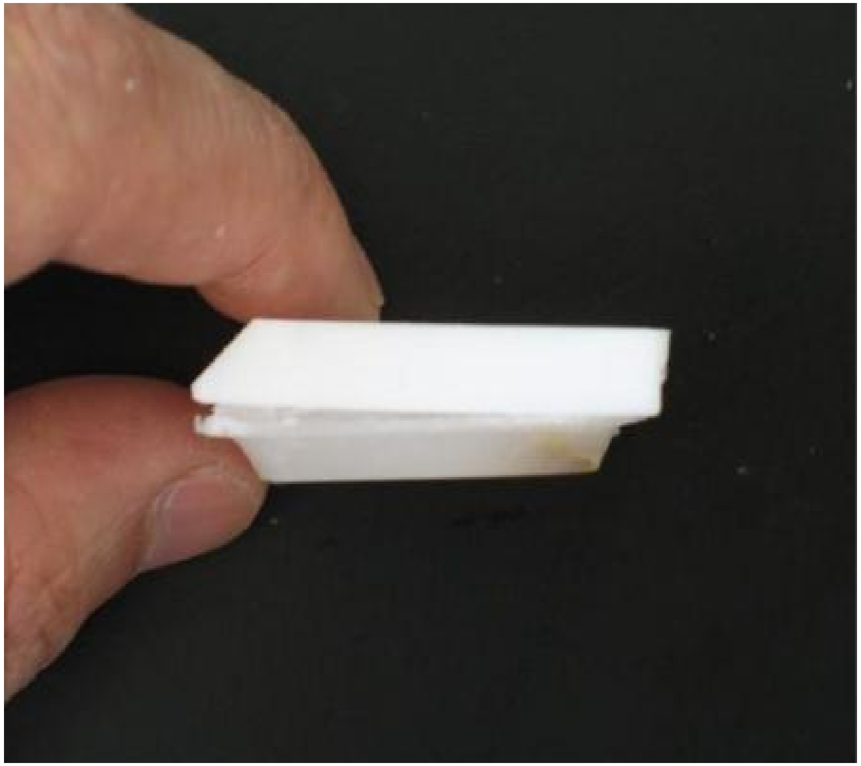

包埋的常见问题分析

空洞：

- 操作时包埋盒盖从上面盖的太猛，未缓慢贴边盖。
- 第一次注入蜡液过少。
- 二次加蜡时，没有从右上角慢慢注入。

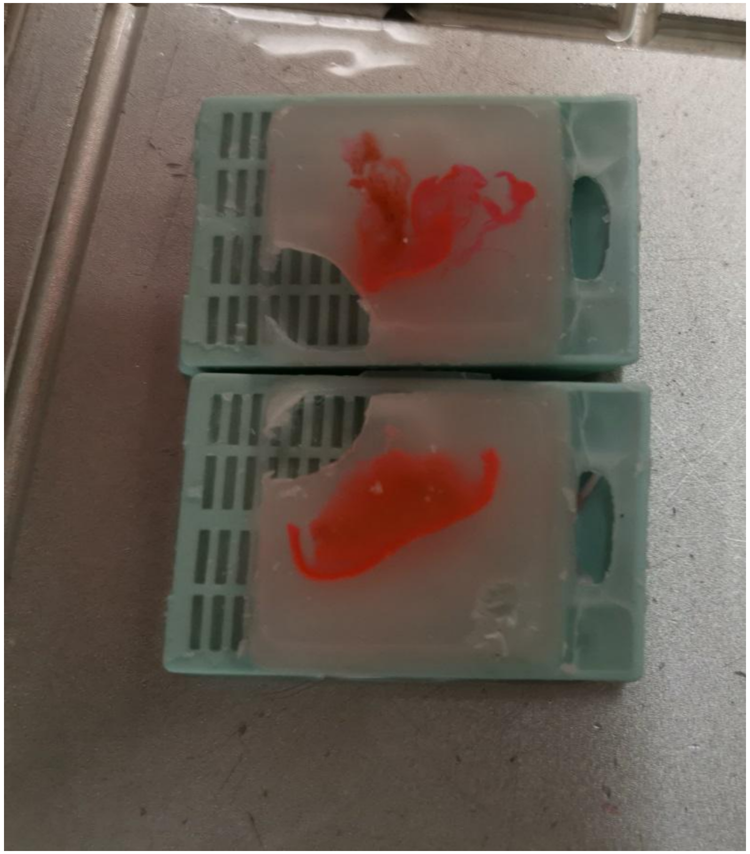

包埋的常见问题分析

石蜡与组织分离：

- 包埋时，一下拿出好多包埋盒，未包埋组织在外放置时间长变凉，不能与较热蜡液很好的融合。
- 放组织蜡块的盒子温度设置偏低，组织蜡块很容易变凉，不能与蜡液很好的融合。

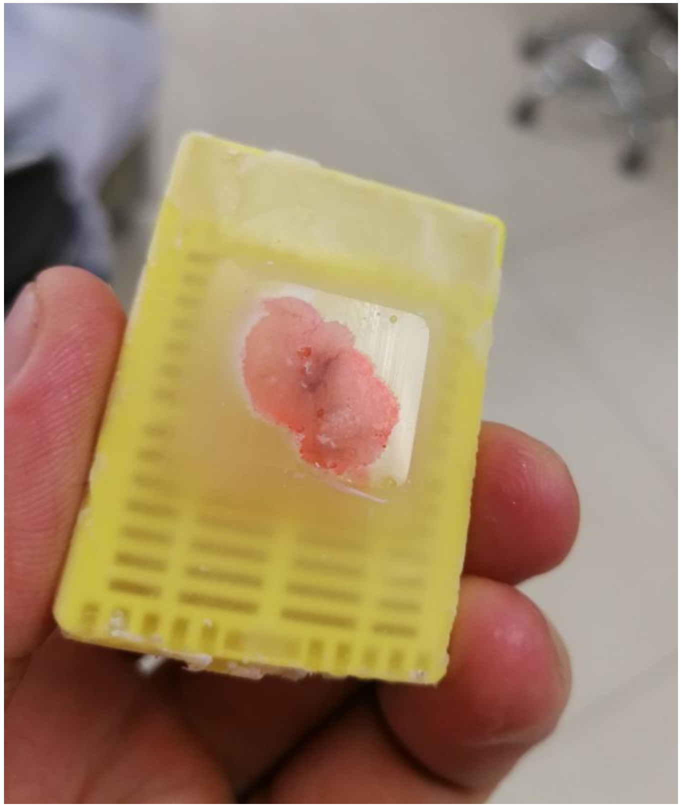

郑州大学第一附属医院 The First Affiliated Hospital of Zhengzhou University

包埋的常见问题分析

组织分散不集中：

- 操作时，没等小冷台上模具中的石蜡凝固，就把模具移至冷冻台，在移动的过程中使组织偏离。
- 冷冻台上结冰，台面凹凸不平，模具放置时倾斜，导致蜡液流出，组织随之偏离。

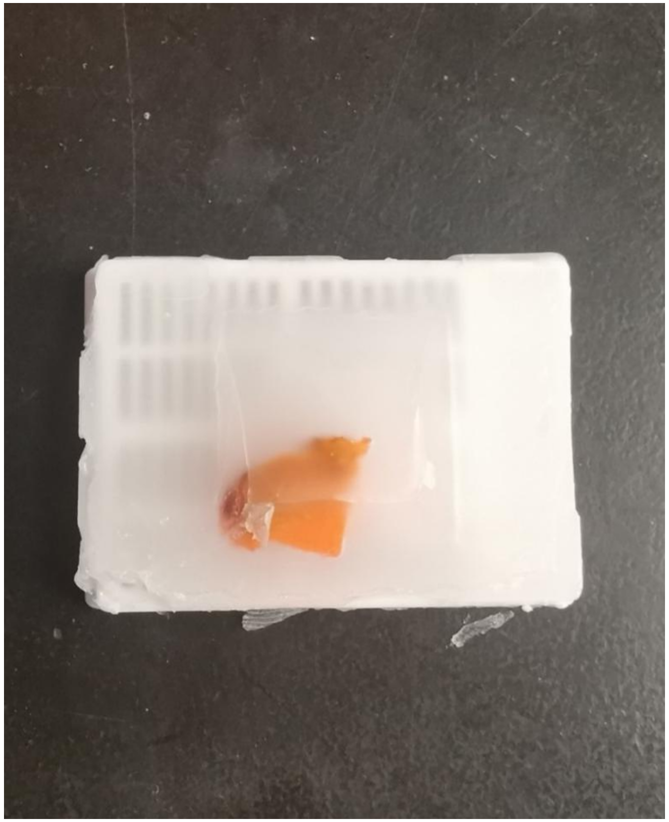

郑州大学第一附属医院
The First Affiliated Hospital of Zhengzhou University

一 包埋的定义

二 包埋的工具及准备事项

三 包埋的操作流程

四 包埋的注意事项

五 包埋的常见问题分析

六 **包埋机的维护**

包埋机的维护

- 包埋完毕要在熔蜡槽里加满石蜡，为第二天的包埋做好准备，并且要定期清理过滤网。
- 废蜡缸要做到及时清理。

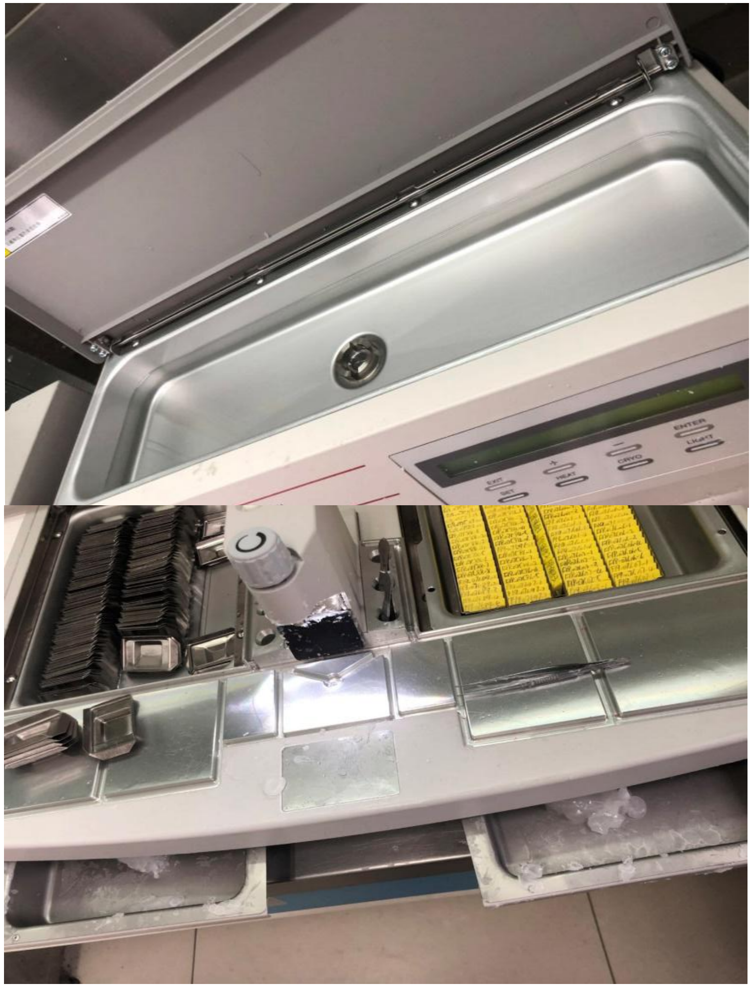

包埋机的维护

- 日常工作中要保证包埋机定时开关机。
- 使用完毕后，将包埋槽中放好包埋模具，供下次使用。大小冷台和照明灯用完后要及时关闭，以保证机器的使用寿命。
- 包埋机、冷台和桌面，应及时清理，保持干净整洁。

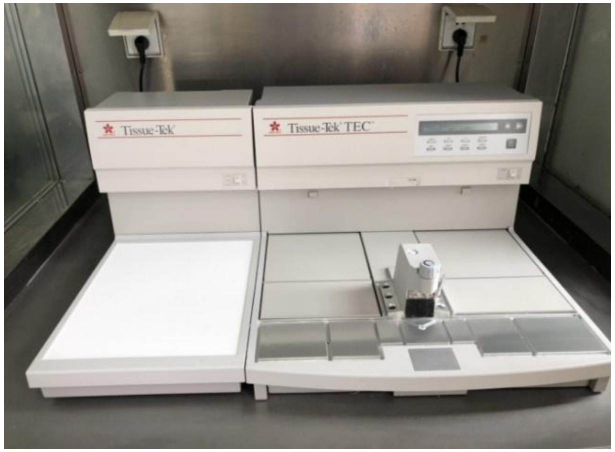

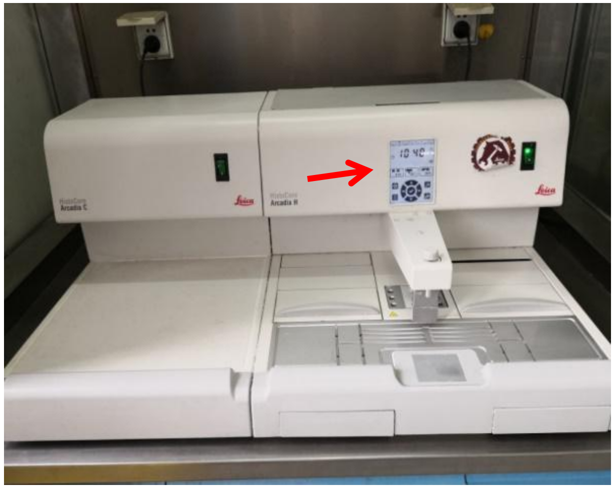

包埋，是病理常规技术制片过程中，非常重要的一个环节，需要每一位技术人员，认真核对每一个标本和每一个步骤。

工作时，保持认真和静心的态度，才能最终制作出精美的切片！！！

常规技术室HE切片全流程质量控制

The quality control of HE slice in the conventional technology room

病理科质量控制指标

- 每百张病床病理医师数
- 每百张病床病理技术人员数
- 标本规范化固定率 √
- HE染色切片优良率 √
- 免疫组化染色切片优良率 √
- 术中快速病理诊断及时率 √
- 组织病理诊断及时率
- 细胞病理诊断及时率
- 各项分子病理检测室内质控合格率 √
- 免疫组化室间质评合格率 √
- 各项分子病理室间质评合格率 √
- 细胞学病理诊断质控符合率
- 术中快速诊断和石蜡诊断符合率

- 病理常规技术是病理科工作中极其重要的一部分
- 常规病理技术工作是一个环环相扣的过程，任一环节的不足或处理不当，都会对最终的制片质量造成影响
- 目前各医院的病理样本量急剧增加，对HE切片质量的要求也逐渐提高。

流程化管理
规范每一环节

→

保证并提高常规病理制片质量

病理科工作流程

预处理
↓
取材
↓
固定脱水浸蜡
↓
包埋
↓
切片
↓
染色
↓
出片
↓
HE切片 → 免疫组化/分子病理
↓
诊断

常规

技术室

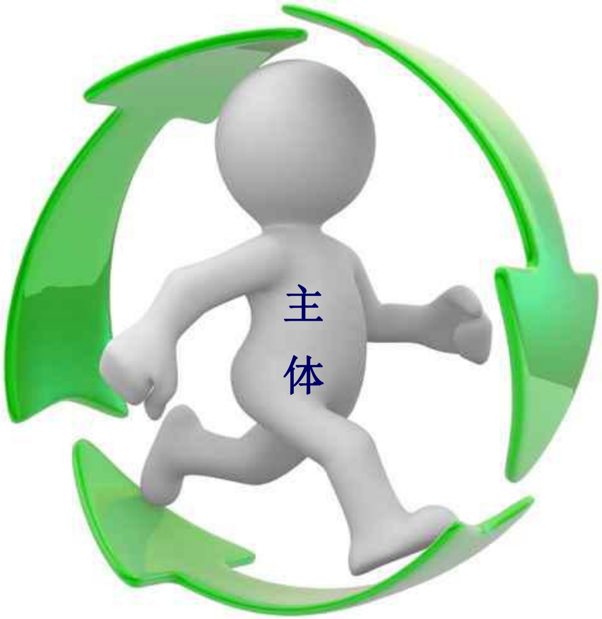

病理技术人员建设

- 培养技术人员专业素养以及责任心
- 技术专家进行技术培训
- 不断引进新技术新方法
- 多组织多参加学会协会的学习，科室每周举行技术学习

1 预处理及固定

2 取材

3 固定、脱水与浸蜡

4 包埋

5 切片

6 染色

7 出片

预处理及固定

- 预处理为病理检查工作的第一步，对后续病理工作至关重要
- 防止标本固定不充分，避免组织自溶
  - 大标本沿最大剖面切开，肝脾肾等实性器官书页状切开，　　并于切面夹滤纸，方便固定液渗入
  - 空腔器官剪开，平铺，订于石蜡板
  - 淋巴结等致密组织，沿长轴从中央做纵切面

预处理及固定

- 固定为组织处理第一步，也是最重要的一步，一旦出现问题，后续的组织处理步骤将无法补救
- 措施
  - 组织一旦离体，及时固定
  - 固定液充足，至少为组织体积的8-10倍
  - 固定时间充足而不过度，约12-48小时
  - 使用达到质量控制标准的固定液，10%中性缓冲福尔马林

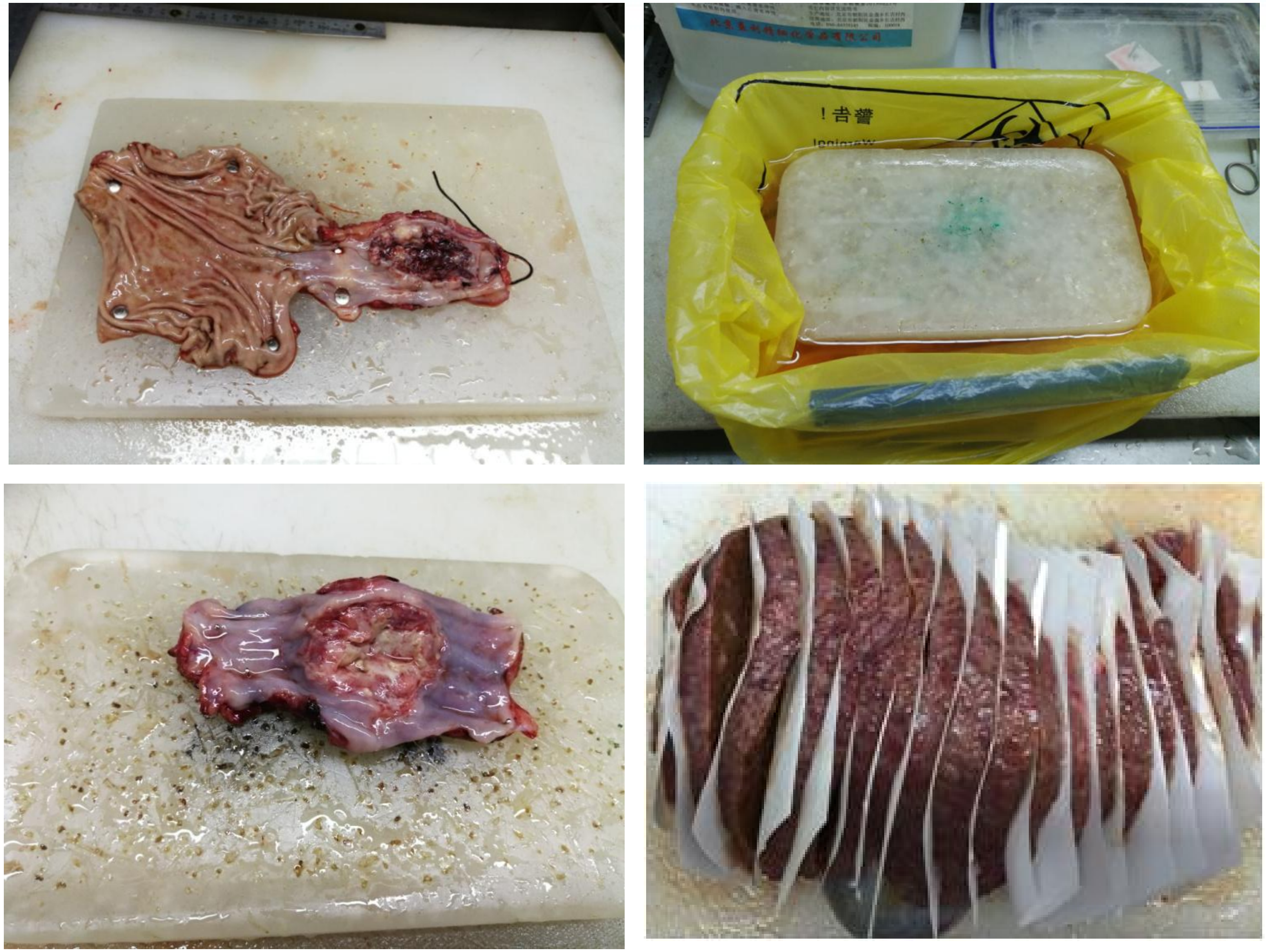

预处理操作示例

取材

- 取材标准统一，制定取材标准作业程序（SOP）
- 要求厚薄均匀，不能超过2cm*2cm*0.3cm,组织过大过厚均导致脱水不佳，影响制片

包埋工作人员对这一步骤的取材工作进行质量反馈，一旦出现问题及时反馈沟通，避免类似问题再次出现

组织固定、脱水、浸蜡

- 固定 → 脱水 → 透明 → 浸蜡，前一步骤处理不彻底均会影响下一步骤，过度脱水和浸蜡还会影响抗原暴露
- 措施
  - 针对使用的脱水机制定标准作业程序
  - 及时更换脱水试剂

后续的切片及染色步骤的工作人员对此步骤进行质量反馈，一旦发现脱水不良，及时查找原因

①号莱卡脱水机挂机明细及换液记录

年月	上机块数	总数	门小	病小	病大	冰冻	开始	结束	F	75	85	95	95	无水	无水	苯	苯	苯	蜡	蜡	蜡	清洗	签名
1	303	909	✓		✓		15:30	6:30	✓	✓													[illegible]
2	303	1212																					
3	303	1515			✓		16:00	6:30															
4	[illegible]	1568	✓		✓		17:00	6:40															
5	296	1864	✓		✓		16:00	6:30											✓		✓		[illegible]
6	303	303	✓				16:30	6:30	✓	✓	✓		✓		✓		✓	✓				✓	[illegible]
7	303	606		✓		✓	18:40	7:29															
8	303	909	✓	✓			16:30	6:30															
9	606	1515		✓	✓				✓	✓													
10	303	1818		✓		✓	18:00	7:50															
11																							
12 (-)	303	2121	✓		✓		16:57	6:30												✓			
13	303	303	✓	✓			16:45	6:30	✓	✓	✓	✓		✓		✓						✓	[illegible]
14	303	606	✓			✓	[illegible]	10:35															
15	303	909	✓		✓		16:00	6:30															
16	394	1303			✓	✓	19:15	8:49	✓	✓													
17	303	1606		✓			[illegible]	6:10															
18	60	1666	✓				[illegible]	6:30															
19									✓										✓				[illegible]
✓20	303	303	✓		✓		16:15	6:30	✓	✓	✓		✓		✓		✓					✓	[illegible]
21	303	606	✓	✓	✓		16:10	6:30															
22	303	909	✓		✓		19:30	9:02															
23	303	1212	✓	✓			16:50	6:30	✓	✓													[illegible]
24	303	1515		✓	✓		19:20	8:51															
25	75	1590																					
26	291	1881	✓	✓	✓		16:00	6:30													✓		[illegible]
27	303	303	✓	✓			16:30	6:30	✓	✓	✓	✓		✓				✓				✓	[illegible]
28	303	606			✓		19:37	9:10															
29																							
30																							
31																							

记录每日挂机量，及时换液，确保组织脱水效果

包埋

- 制定包埋标准
  - 方位正确，胃肠内镜小标本、囊壁、管腔以及皮肤等组织立埋
  - 最大组织面包埋
- 病理技术人员必须严格执行包埋标准，定期培训与考核

切片工作人员对这一步骤的包埋工作进行质量反馈，一旦出现包埋问题及时反馈沟通，重新包埋

切片

- 制定切片标准
  - 组织完整，厚薄均匀
  - 无折痕刀痕
- 病理技术人员必须严格执行切片标准，定期培训与考核

每日质控人员对切片工作进行质量反馈以及抽查打分，一旦出现问题及时反馈沟通，重新切片

包埋与切片

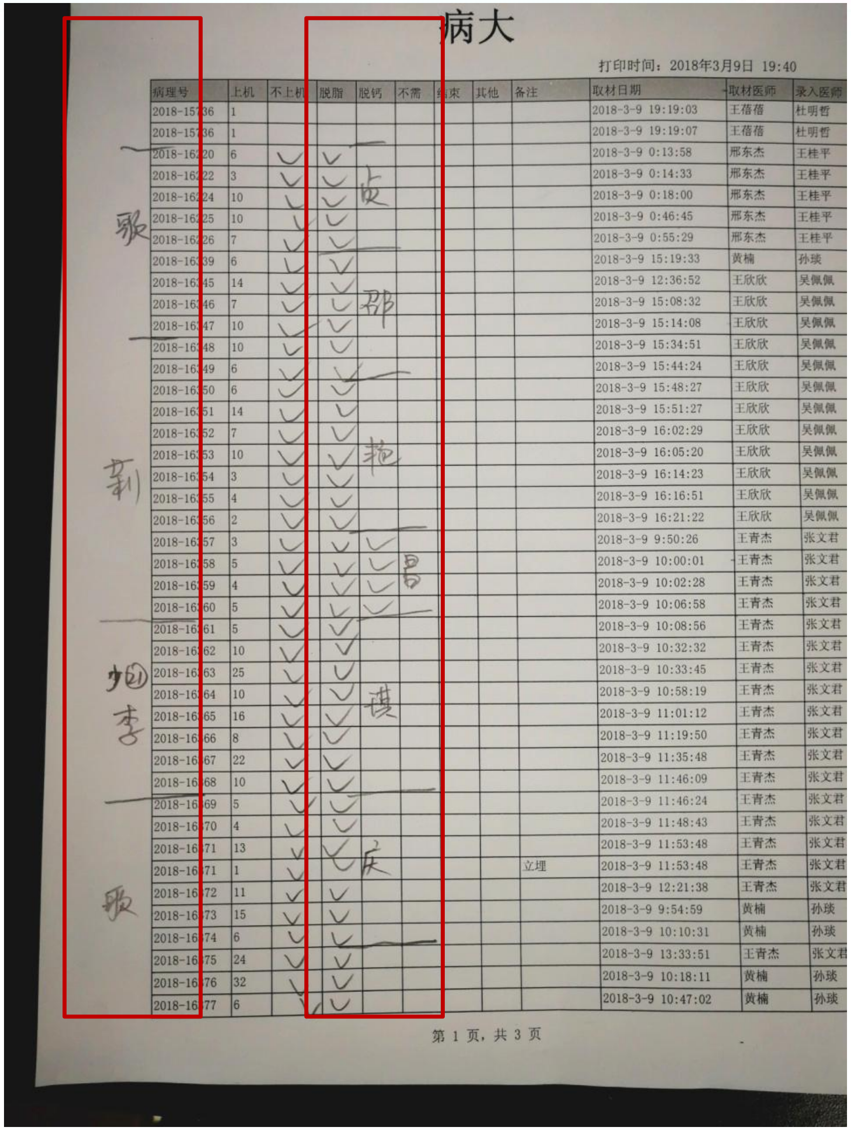

病大

打印时间：2018年3月9日 19:40

病理号	上机	不上机	脱脂	脱钙	不需	结束	其他	备注	取材日期	取材医师	录入医师
2018-15[illegible]36	1								2018-3-9 19:19:03	王蓓蓓	杜明哲
2018-15[illegible]36	1								2018-3-9 19:19:07	王蓓蓓	杜明哲
2018-16[illegible]20	6	✓	✓						2018-3-9 0:13:58	邢东杰	王桂平
2018-16[illegible]22	3	✓	✓						2018-3-9 0:14:33	邢东杰	王桂平
2018-16[illegible]24	10	✓	✓						2018-3-9 0:18:00	邢东杰	王桂平
2018-16[illegible]25	10	✓	✓						2018-3-9 0:46:45	邢东杰	王桂平
2018-16[illegible]26	7	✓	✓						2018-3-9 0:55:29	邢东杰	王桂平
2018-16[illegible]39	6	✓	✓						2018-3-9 15:19:33	黄楠	孙琰
2018-16[illegible]45	14	✓	✓						2018-3-9 12:36:52	王欣欣	吴佩佩
2018-16[illegible]46	7	✓	✓						2018-3-9 15:08:32	王欣欣	吴佩佩
2018-16[illegible]47	10	✓	✓						2018-3-9 15:14:08	王欣欣	吴佩佩
2018-16[illegible]48	10	✓	✓						2018-3-9 15:34:51	王欣欣	吴佩佩
2018-16[illegible]49	6	✓	✓						2018-3-9 15:44:24	王欣欣	吴佩佩
2018-16[illegible]50	6	✓	✓						2018-3-9 15:48:27	王欣欣	吴佩佩
2018-16[illegible]51	14	✓	✓						2018-3-9 15:51:27	王欣欣	吴佩佩
2018-16[illegible]52	7	✓	✓						2018-3-9 16:02:29	王欣欣	吴佩佩
2018-16[illegible]53	10	✓	✓						2018-3-9 16:05:20	王欣欣	吴佩佩
2018-16[illegible]54	3	✓	✓						2018-3-9 16:14:23	王欣欣	吴佩佩
2018-16[illegible]55	4	✓	✓						2018-3-9 16:16:51	王欣欣	吴佩佩
2018-16[illegible]56	2	✓	✓						2018-3-9 16:21:22	王欣欣	吴佩佩
2018-16[illegible]57	3	✓	✓	✓					2018-3-9 9:50:26	王青杰	张文君
2018-16[illegible]58	5	✓	✓	✓					2018-3-9 10:00:01	王青杰	张文君
2018-16[illegible]59	4	✓	✓	✓					2018-3-9 10:02:28	王青杰	张文君
2018-16[illegible]60	5	✓	✓	✓					2018-3-9 10:06:58	王青杰	张文君
2018-16[illegible]61	5	✓	✓						2018-3-9 10:08:56	王青杰	张文君
2018-16[illegible]62	10	✓	✓						2018-3-9 10:32:32	王青杰	张文君
2018-16[illegible]63	25	✓	✓						2018-3-9 10:33:45	王青杰	张文君
2018-16[illegible]64	10	✓	✓						2018-3-9 10:58:19	王青杰	张文君
2018-16[illegible]65	16	✓	✓						2018-3-9 11:01:12	王青杰	张文君
2018-16[illegible]66	8	✓	✓						2018-3-9 11:19:50	王青杰	张文君
2018-16[illegible]67	22	✓	✓						2018-3-9 11:35:48	王青杰	张文君
2018-16[illegible]68	10	✓	✓						2018-3-9 11:46:09	王青杰	张文君
2018-16[illegible]69	5	✓	✓						2018-3-9 11:46:24	王青杰	张文君
2018-16[illegible]70	4	✓	✓						2018-3-9 11:48:43	王青杰	张文君
2018-16[illegible]71	13	✓	✓						2018-3-9 11:53:48	王青杰	张文君
2018-16[illegible]71	1	✓	✓					立埋	2018-3-9 11:53:48	王青杰	张文君
2018-16[illegible]72	11	✓	✓						2018-3-9 12:21:38	王青杰	张文君
2018-16[illegible]73	15	✓	✓						2018-3-9 9:54:59	黄楠	孙琰
2018-16[illegible]74	6	✓	✓						2018-3-9 10:10:31	黄楠	孙琰
2018-16[illegible]75	24	✓	✓						2018-3-9 13:33:51	王青杰	张文君
2018-16[illegible]76	32	✓	✓						2018-3-9 10:18:11	黄楠	孙琰
2018-16[illegible]77	6	✓	✓						2018-3-9 10:47:02	黄楠	孙琰

第 1 页，共 3 页

每一步骤认真核对

追溯到个人

实行蜡块与切片责任制

染色

- 染色机械化，染液试剂商品化
- 针对每种自动染色机和染液试剂，建立染色标准操作流程
- 每日质量控制人员观察HE染色质量，每染色架均抽取前中后三张评价染色质量，适当调整染色步骤或时间以达最优染色效果
- 每天正式染色前进行预测观察染色效果（约6-8张切片）

及时和诊断老师沟通与反馈，评价切片与染色质量，更好地为诊断服务

出片

核对前

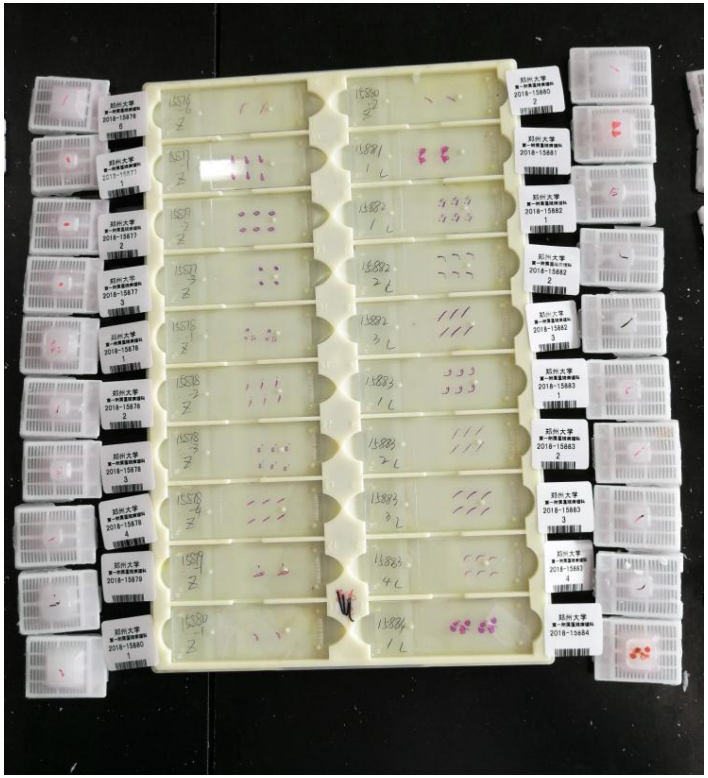

核对后

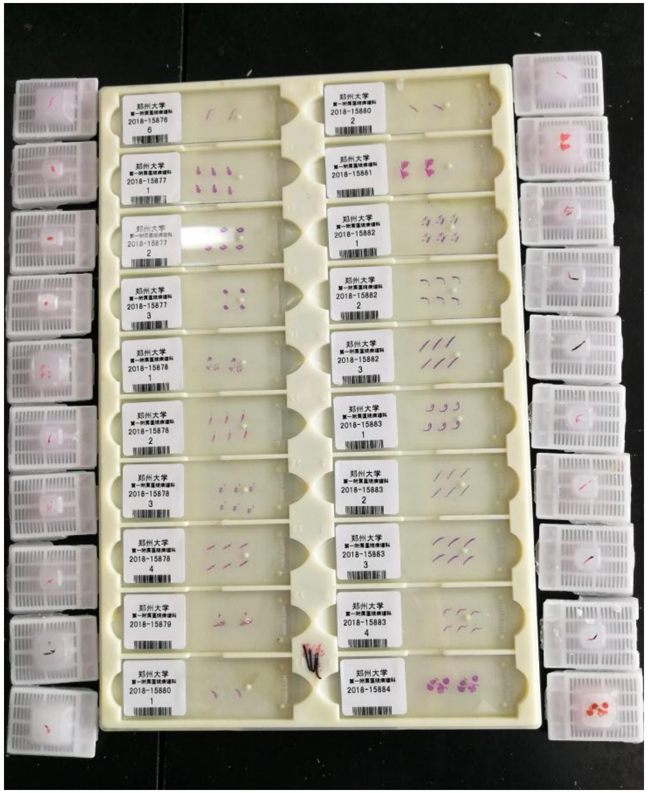

每日质控人员对切片染色进行核对，并对切片进行反馈沟通

出片

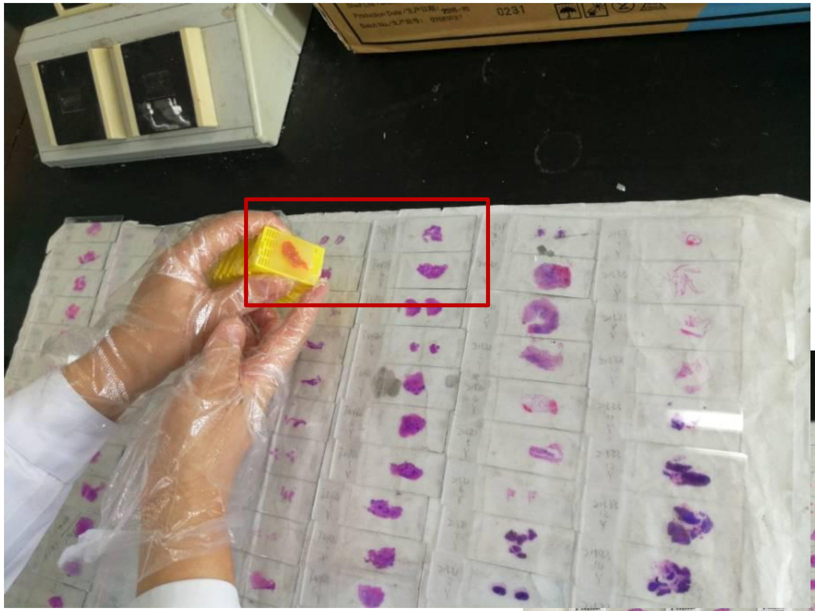

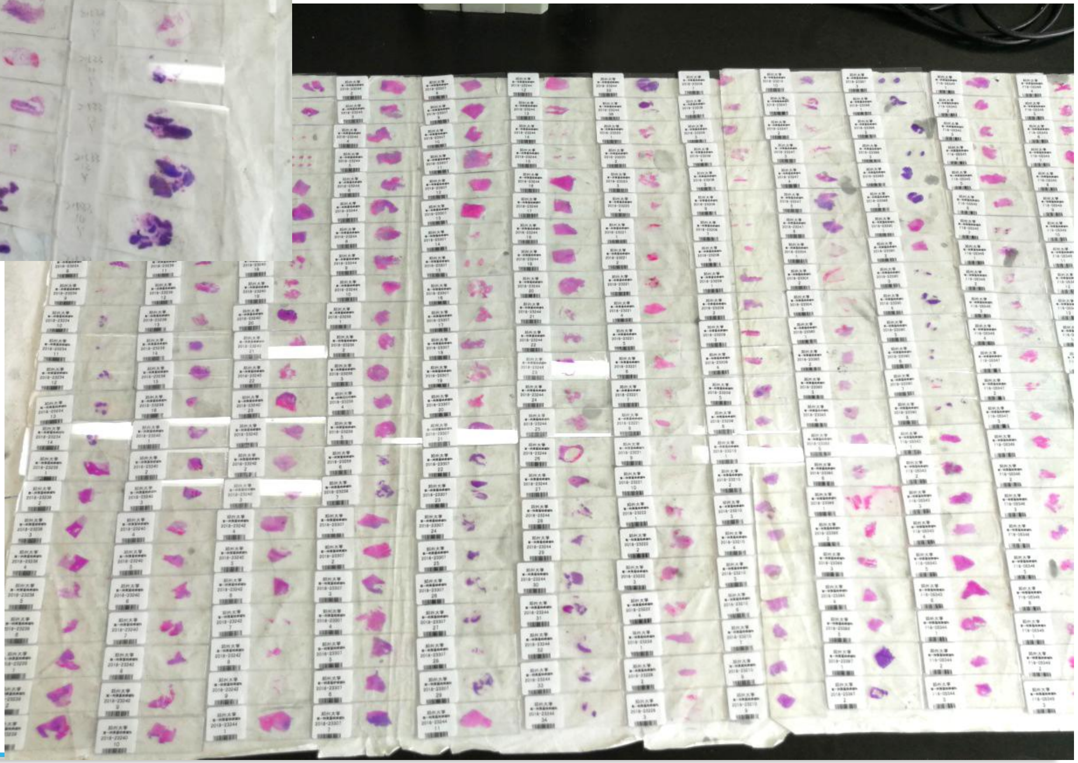

切片与染色

- 每日由诊断医师和技师组成的质控小组根据质控标准对每日切片进行抽取和打分
- 出现问题及时反馈与解决
- 每月对出现问题进行汇总归纳，查找原因

质控依据

- 《病理专业医疗质量控制指标》
- 《病理诊断评价指标体系》
- 《临床技术操作规范-病理学分册》

质控标准

常规石蜡-HE染色切片

$$优良率（\%）=\frac{常规组织病理切片优良数}{常规组织病理切片总数数}\times 100\%$$

分值≥95分为优级切片；分值≥90分为良好切片，90分以上的切片数占总切片数的比率，即为切片优良率

质控评分标准

评分序号	优级标准	满分	质量缺陷减分
1	组织切片完整	10	组织稍不完整：减1-3分；不完整：减4-10分
2	切片厚度适当，厚薄均匀	10	切片厚，影响诊断：减6-10分，厚薄不均减2-5分
3	切片平整，无褶皱、折叠	10	有褶皱或折叠，不影响诊断；各减2分；影响诊断：各减5分
4	切片无刀痕、裂隙、颤痕	10	有刀痕、裂隙、颤痕，不影响诊断：各减2分；影响诊断；各减5分
5	切片无污染物	10	有脏东西不影响诊断：减2分；有污染物：减10分
6	无气泡、盖破片周围无溢胶	10	有气泡；减2分；溢胶：减2分
7	透明度好	10	透明度差：减2分；组织结构不清晰：减5-10分
8	细胞核与细胞质染色对比鲜明	10	细胞和色浅或过蓝：减5分；细胞核浆对比不鲜明：减5分
9	切片无松散，裱贴位置适当	10	切片松散：减5分；裱贴不当：减5分
10	切片整洁、标签端正贴牢，编号清晰	10	切片不整洁：减3分，标签不正：减2分
合计		100	

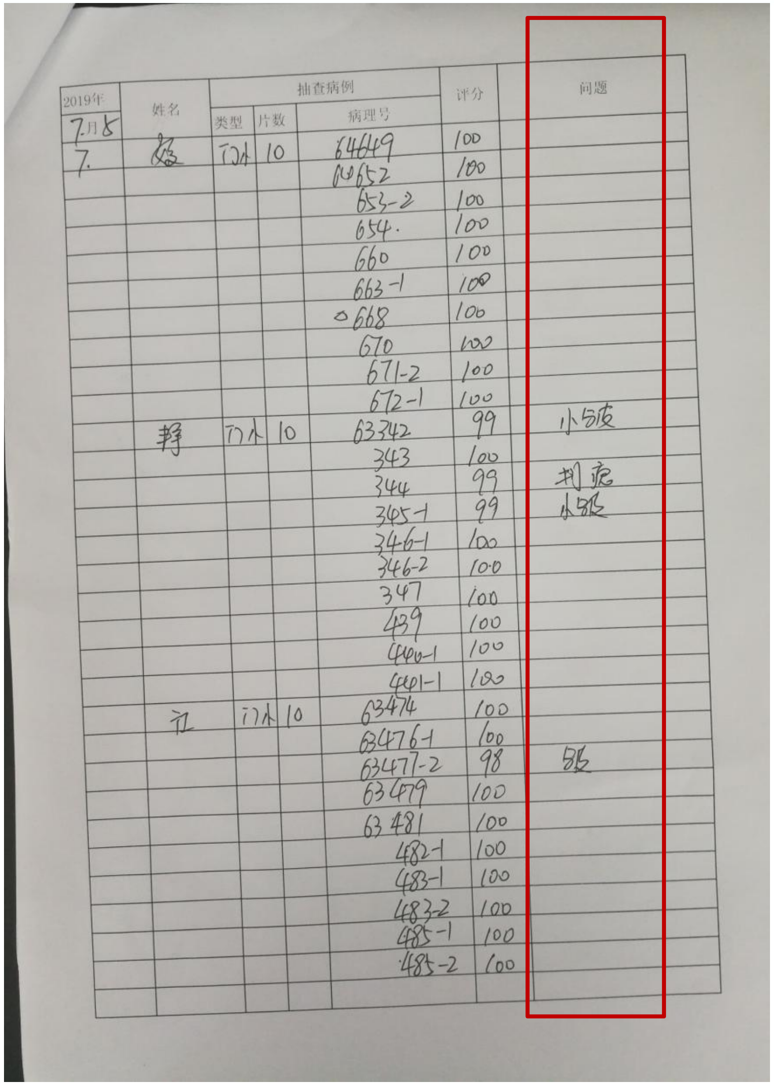

2019年	姓名	抽查病例 类型	片数	病理号	评分	问题
7月5 7.	[illegible]	门小	10	64649	100	
				[illegible]652	100	
				653-2	100	
				654.	100	
				660	100	
				663-1	100	
				668	100	
				670	100	
				671-2	100	
				672-1	100	
	[illegible]	门小	10	63342	99	小5波
				343	100	
				344	99	刀痕
				345-1	99	小5波
				346-1	100	
				346-2	100	
				347	100	
				439	100	
				440-1	100	
				441-1	100	
	江	门小	10	63474	100	
				63476-1	100	
				63477-2	98	5波
				63479	100	
				63481	100	
				482-1	100	
				483-1	100	
				483-2	100	
				485-1	100	
				485-2	100	

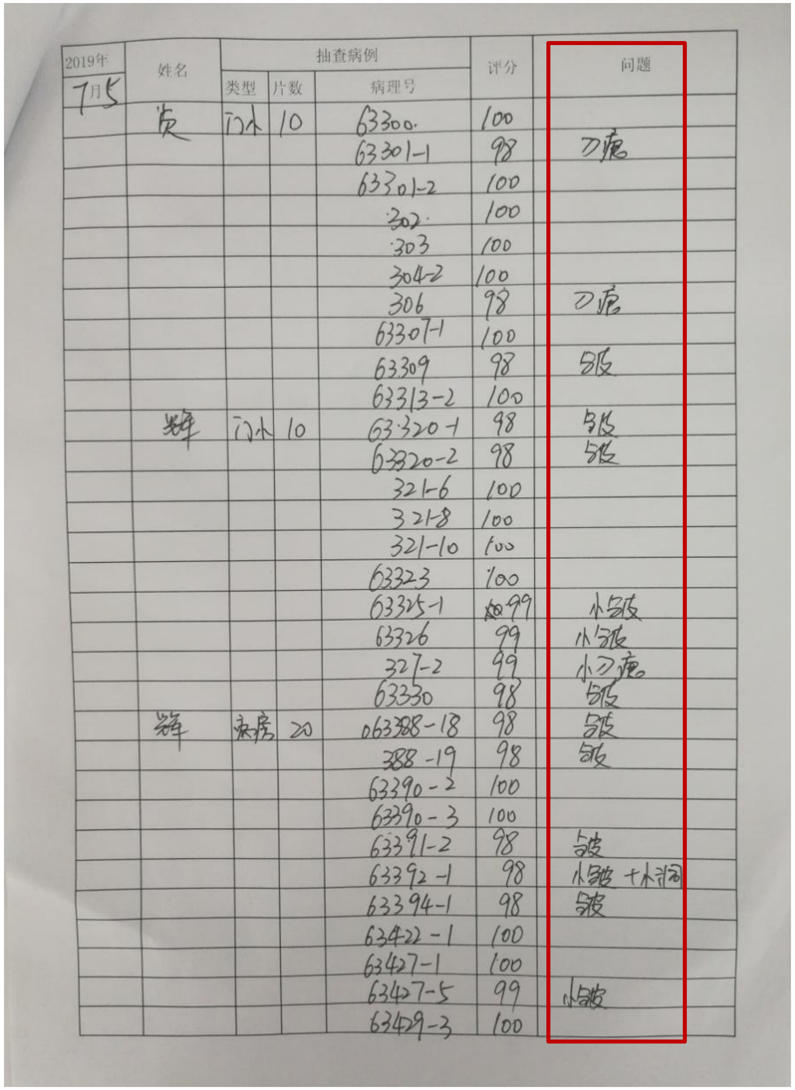

2019年	姓名	抽查病例 类型	片数	病理号	评分	问题
7月5	[illegible]	门小	10	63300	100	
				63301-1	98	刀痕
				63301-2	100	
				302	100	
				303	100	
				304-2	100	
				306	98	刀痕
				63307-1	100	
				63309	98	5波
				63313-2	100	
	辉	门小	10	63320-1	98	5波
				63320-2	98	5波
				321-6	100	
				321-8	100	
				321-10	100	
				63323	100	
				63325-1	99	小5波
				63326	99	小5波
				327-2	99	小刀痕
				63330	98	5波
	辉	病房	20	063388-18	98	5波
				388-19	98	5波
				63390-2	100	
				63390-3	100	
				63391-2	98	5波
				63392-1	98	小5波 +小[illegible]
				63394-1	98	5波
				63422-1	100	
				63427-1	100	
				63427-5	99	小5波
				63429-3	100	

问题	褶皱	刀痕	空洞	切片不干净	厚薄不均	切片不完整	颤痕	封片欠佳	裂隙	颤痕	污染	总量
张数（总1950）	644	142	86	23	9	1	8	2	2	8	0	856
问题发生率	33.03%	7.28%	4.41%	1.18%	0.46%	0.05%	0.41%	0.10%	0.10%	0.41%	0.00%	43.90%

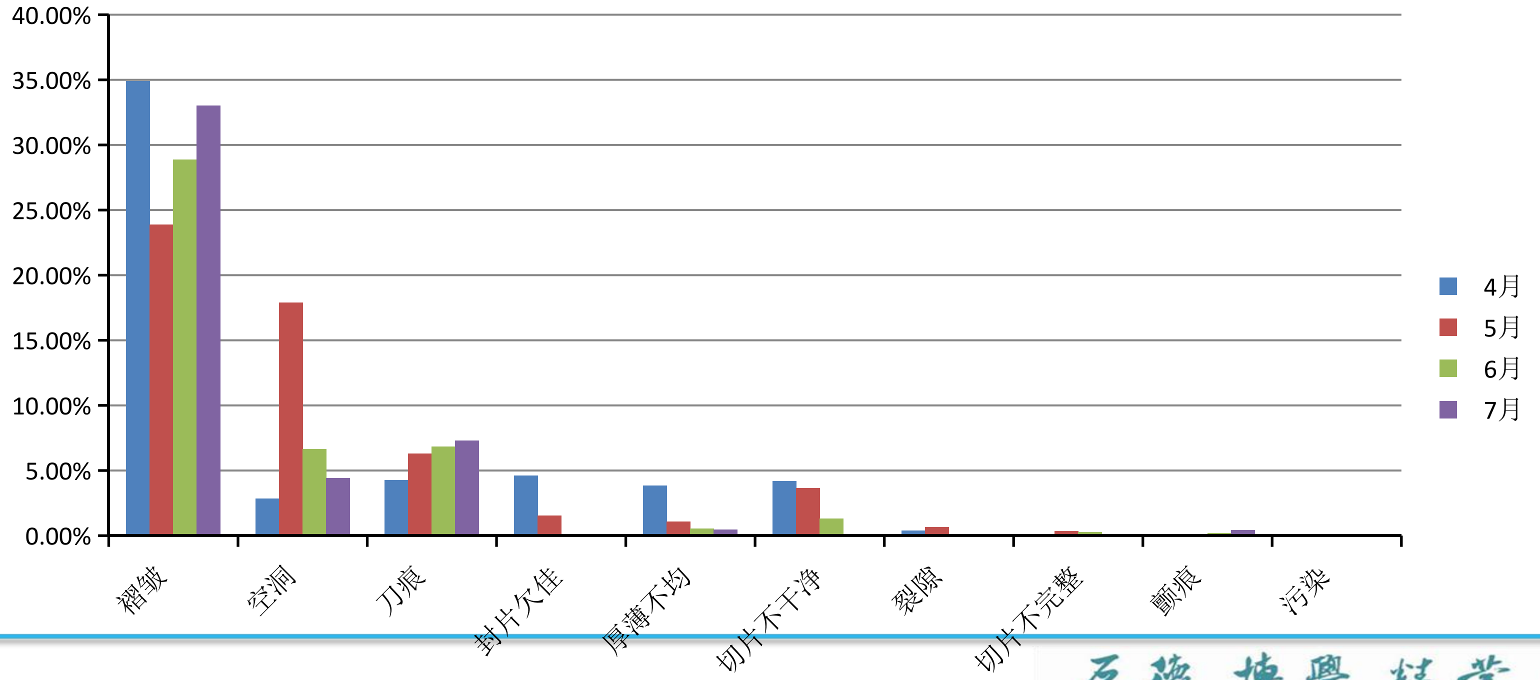

流程总结

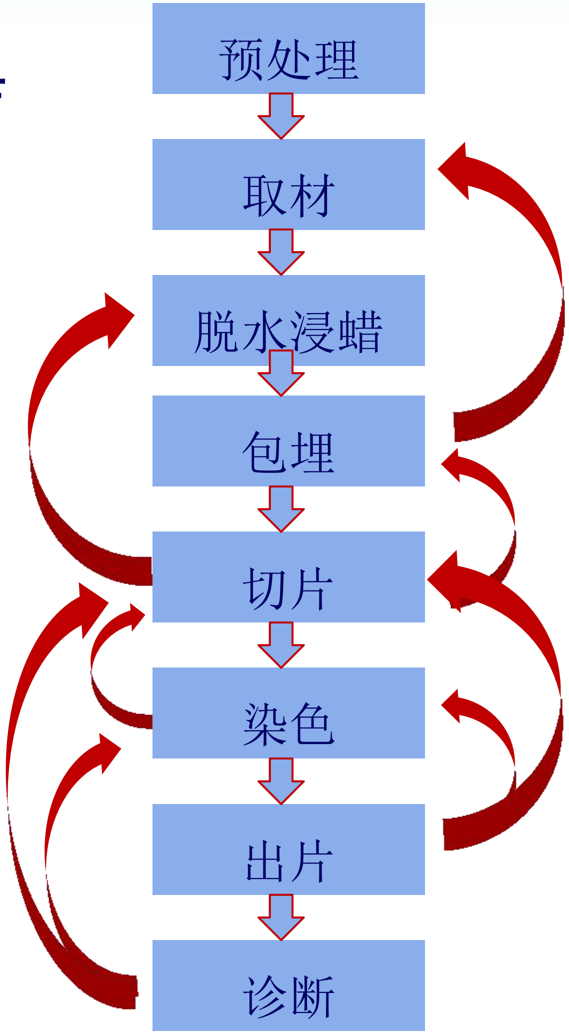

- 各操作均制定SOP
- 每步骤均对上一步骤进行监督反馈
- 追溯到个人，实行样本责任制

总结

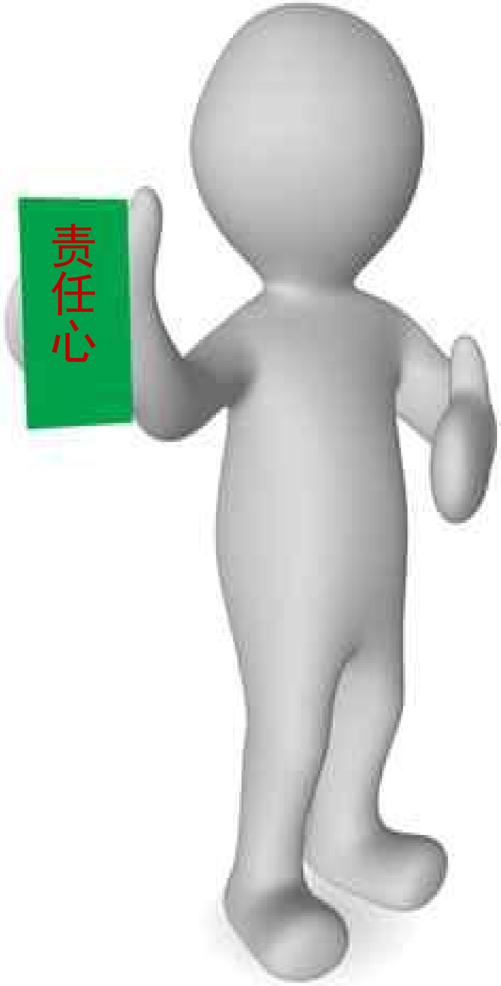

- 培养技术人员专业素养以及责任心
- 技术专家进行技术培训
- 不断引进新技术新方法
- 多组织多参加学会协会的学习

谢谢！

病理科常用设备的使用和维护

Use and maintenance of common equipment in pathology department

1 组织包埋机

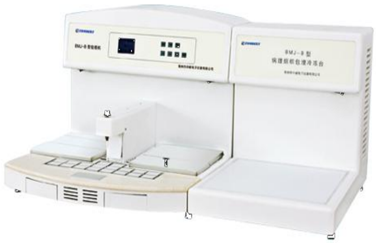

2 切片机

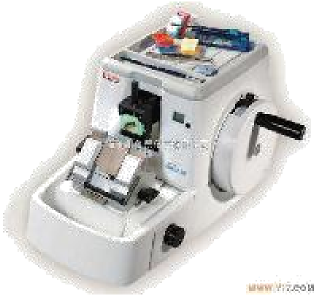

3 自动染色封片机

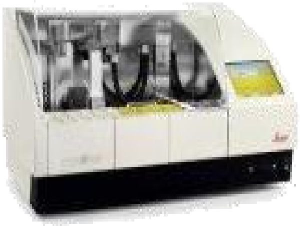

4 全封闭组织脱水机

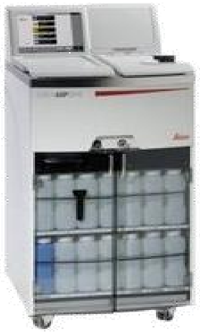

组织包埋机

组织包埋机

包括以下几个方面：

1.设备型号

2.设备组件

3.操作步骤

4.维护和保养

5.常见问题及原因分析

组织包埋机

1.设备型号：

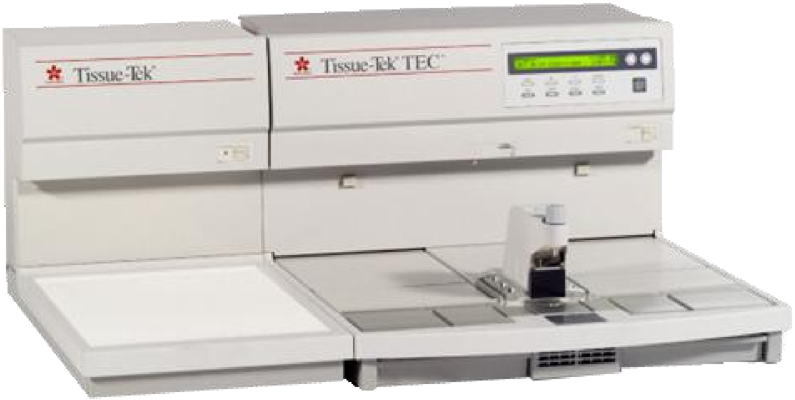

樱花组织包埋机

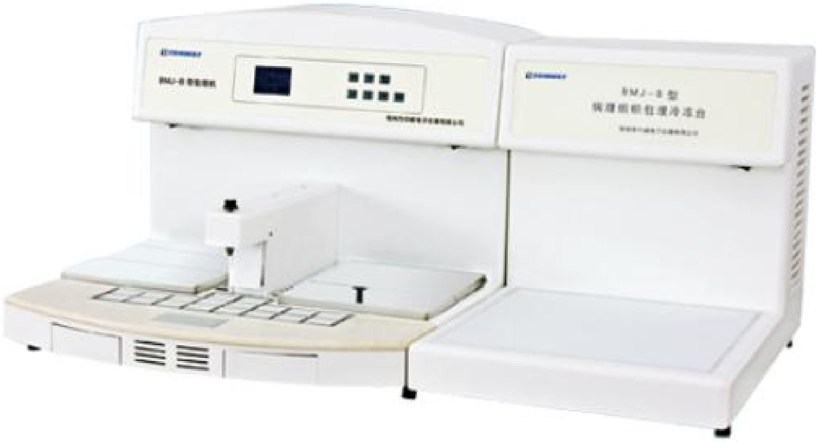

中威组织包埋机

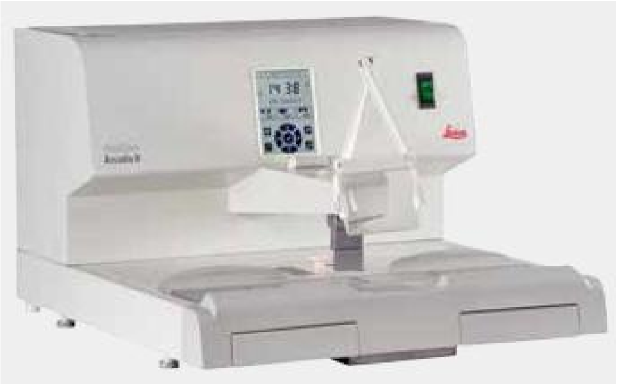

徕卡组织包埋机

组织包埋机

2.设备组件：

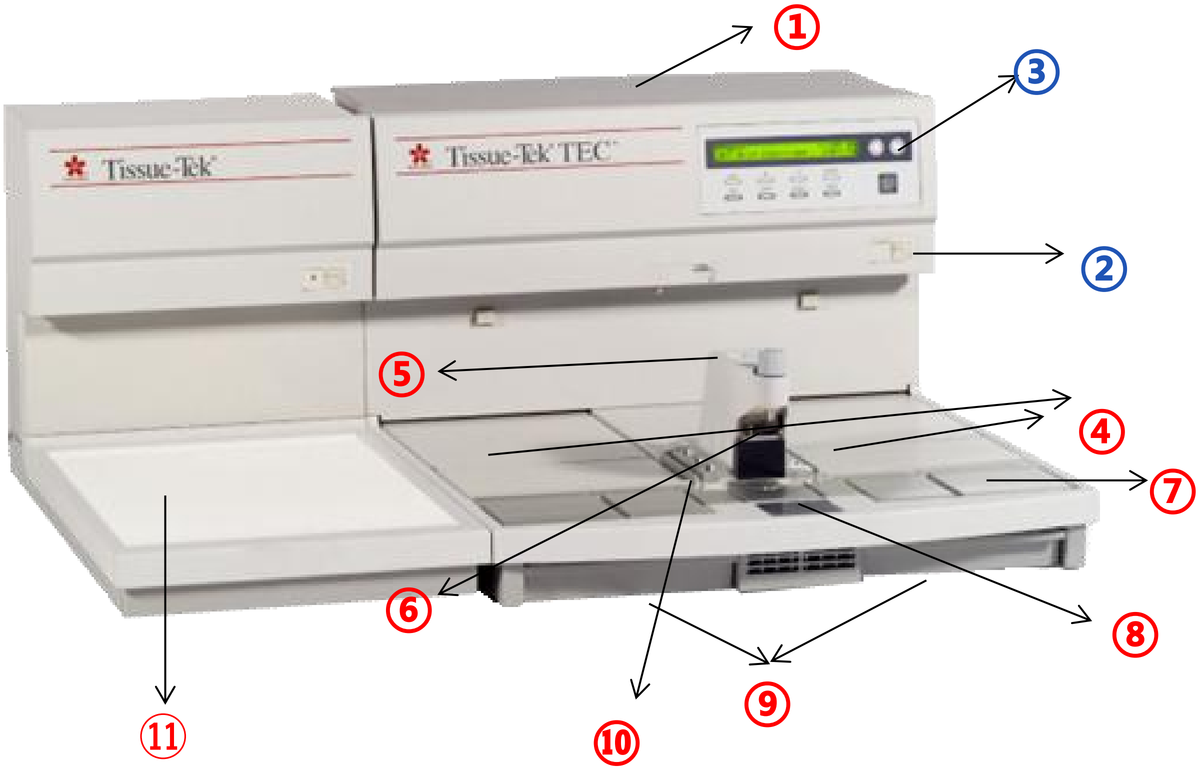

①蜡缸

②总电源开关：关机后不能自动开启。

③控制面板：关机后可以按预设时间自动开启；调节温度；设置时间；预设开机时间；切换自动与手动模式。

④左保存盒和右保存盒

⑤分配器

⑥工作区照明灯

⑦工作台

⑧小冷却台

⑨石蜡收集盘

⑩镊子架

⑪大冷却台

组织包埋机

3.操作步骤：

(1)包埋前三查看：查看机器是否处于开启状态；查看左保存盒有无提前预热的包埋模具；查看是否有两把包埋镊子。

(2)从脱水机取出组织放于右保存盒，开始包埋。

(3)包埋结束后清理废蜡。

(4)加蜡。

(5)包埋后三关闭：关闭照明灯；关闭大小冷台；关闭控制面板电源。

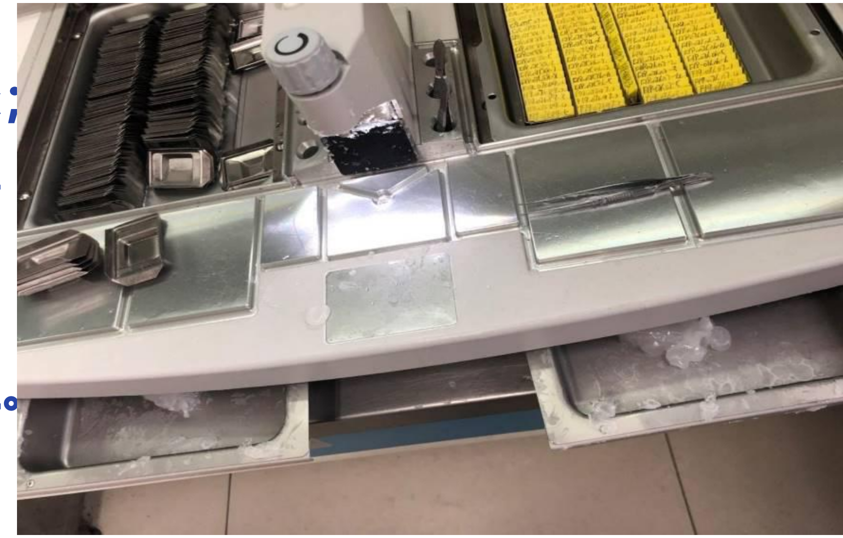

组织包埋机

4.维护和保养：

每次使用完机器用清洁塑料铲清除设备表面滴落的石蜡。

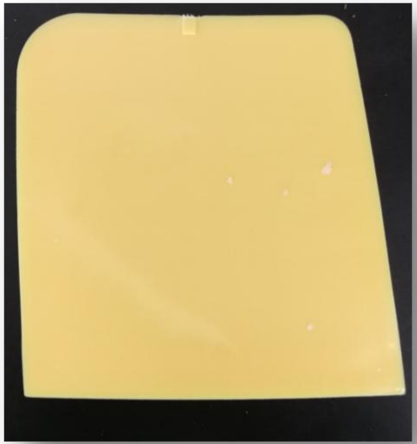

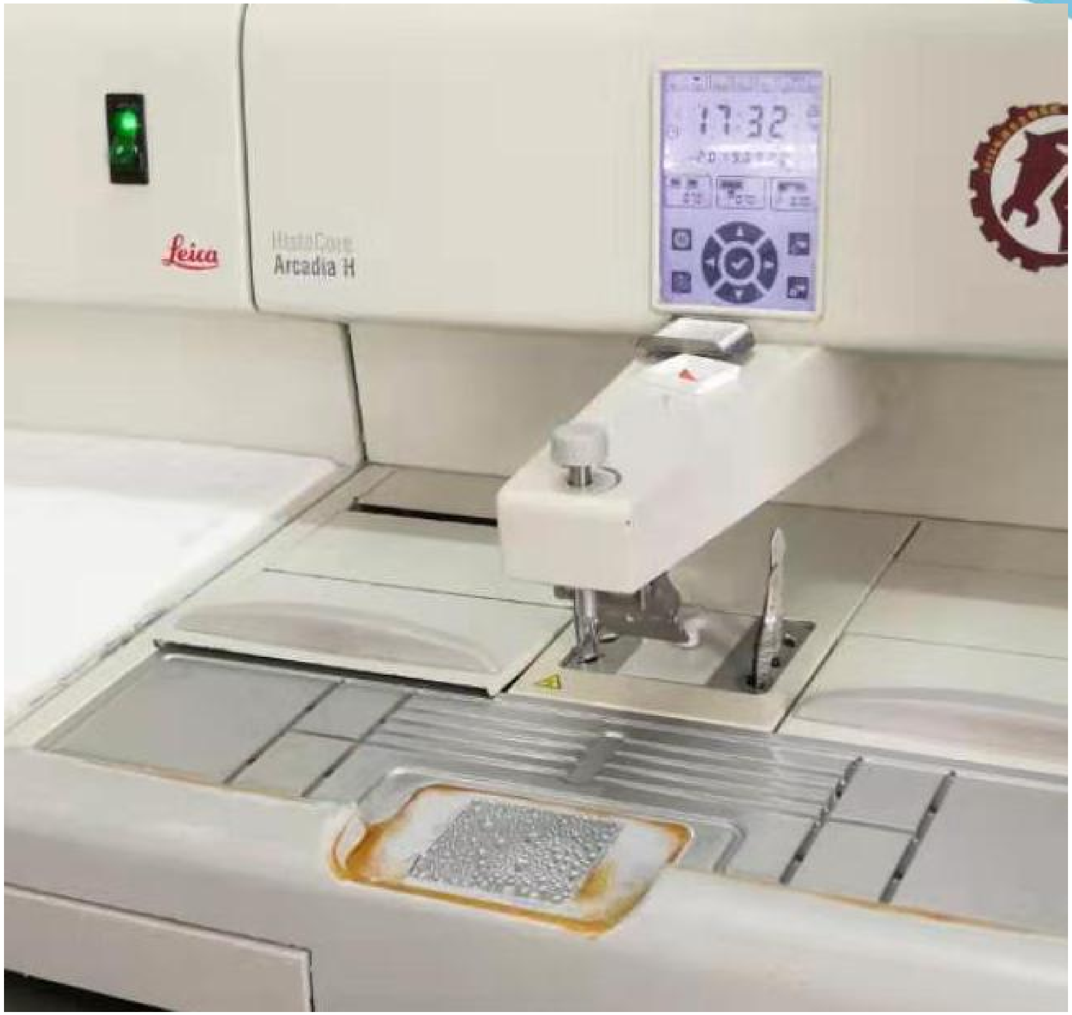

组织包埋机

5.常见问题及原因分析：

(1)包埋结束后直接关闭总电源开关，导致机器不能按照预设的时间自动开启。

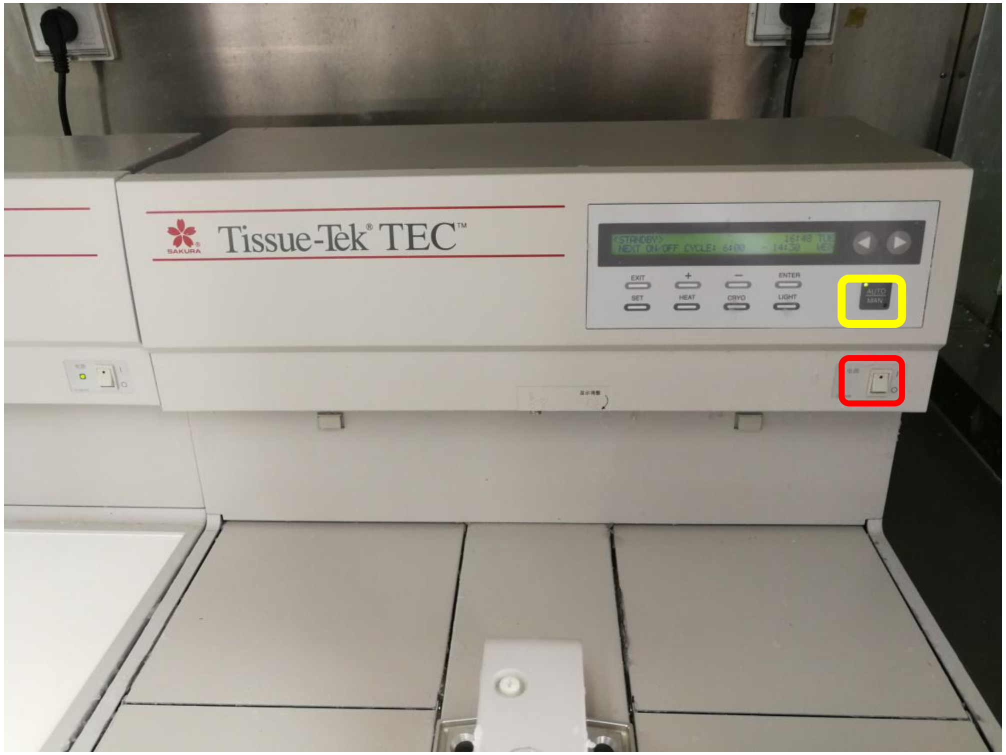

组织包埋机

⑵包埋结束后加蜡量过多，导致溶化的石蜡溢出后烧坏主机（樱花蜡缸的最大容量为6L；徕卡为4L；中威为5.2L）。

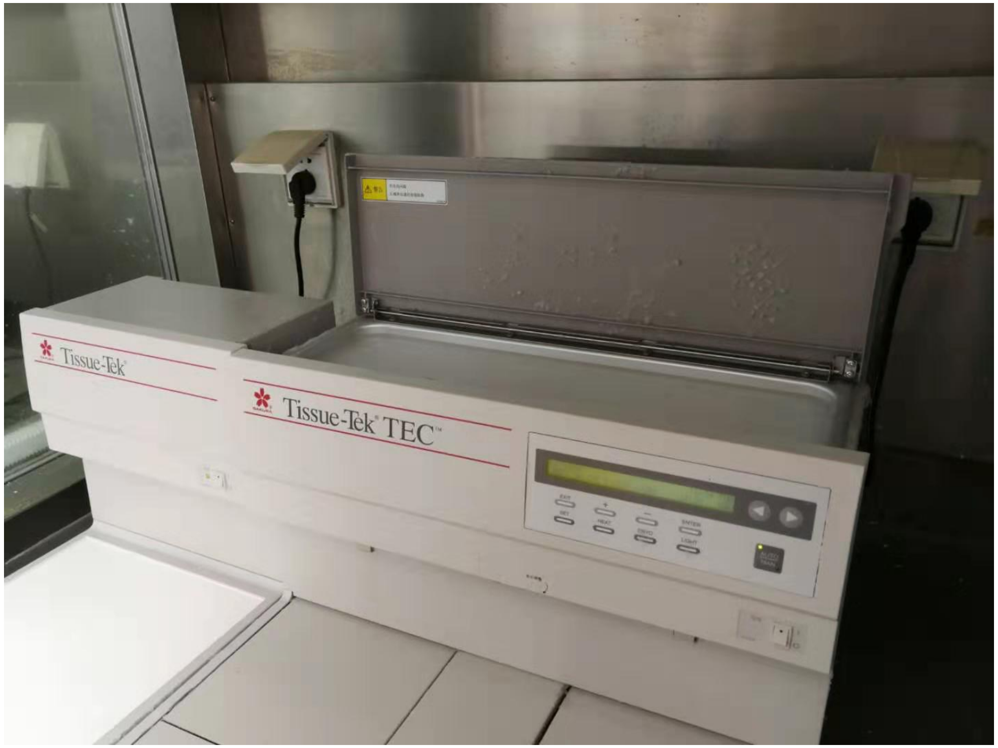

组织包埋机

(3)包埋结束后没有及时清理废蜡，导致小冷却台不致冷。

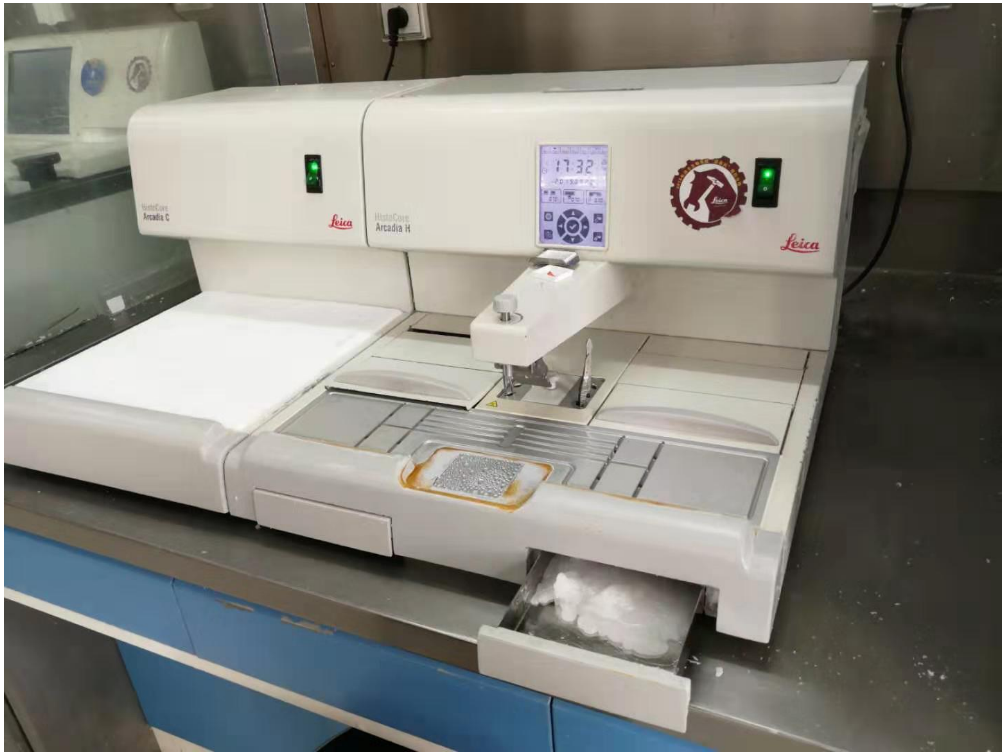

组织包埋机

(4)中威小冷台工作结束后未关闭，小冷台故障不制冷；控制面板电源未关闭。

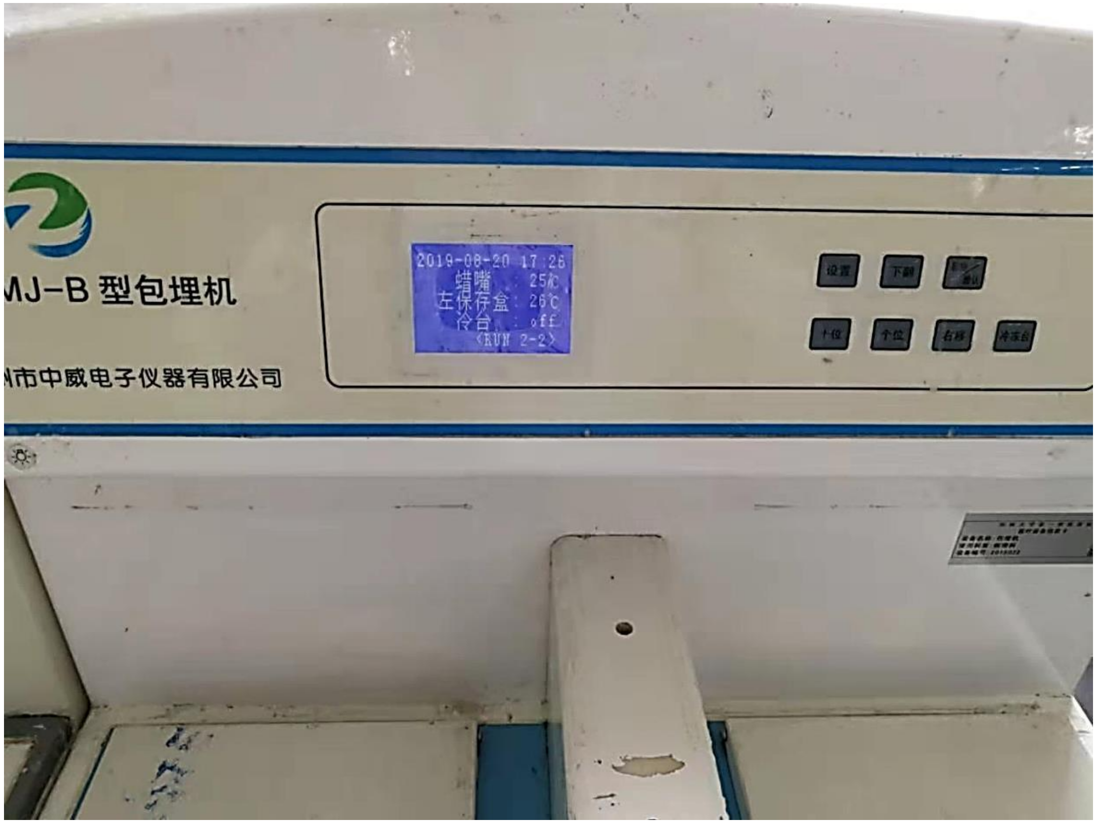

组织包埋机

(5)出厂时间设置错误，预设的开机时间不准确，导致不能准时开启。

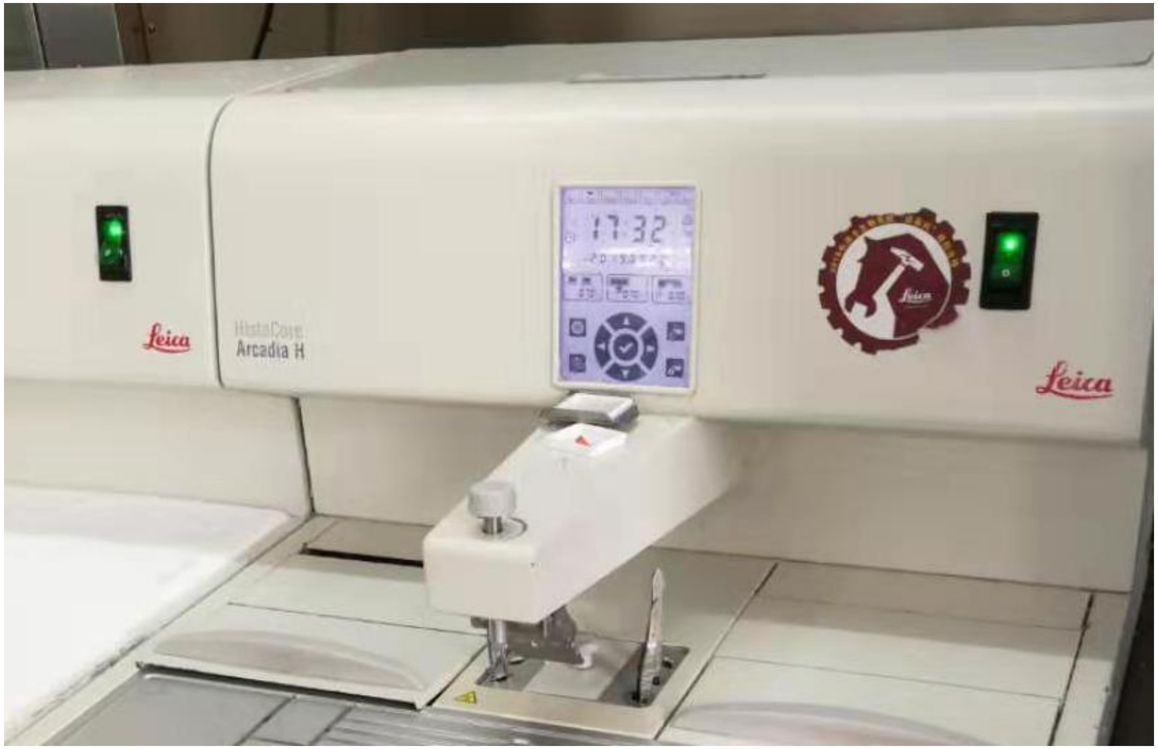

切片机

石蜡切片机

包括以下几个方面：

1. 设备型号
2. 设备组件
3. 操作步骤
4. 维护和保养
5. 常见问题及原因分析

石蜡切片机

1.设备型号：

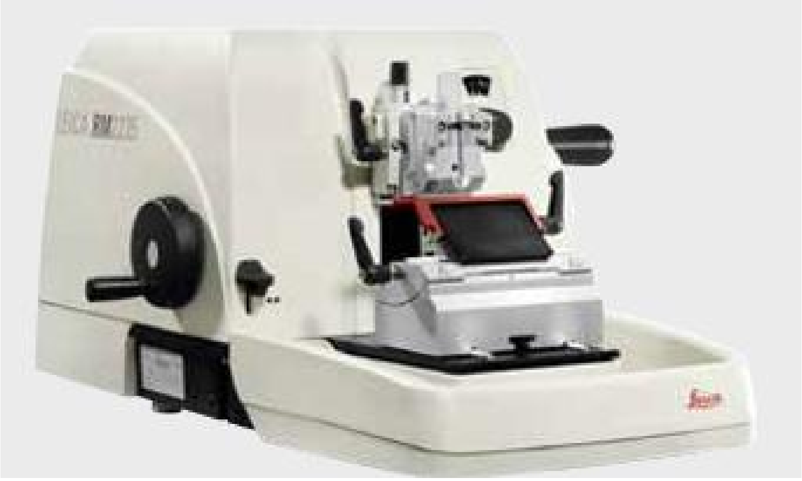

徕卡RM2235

石蜡切片机

2.设备组件:

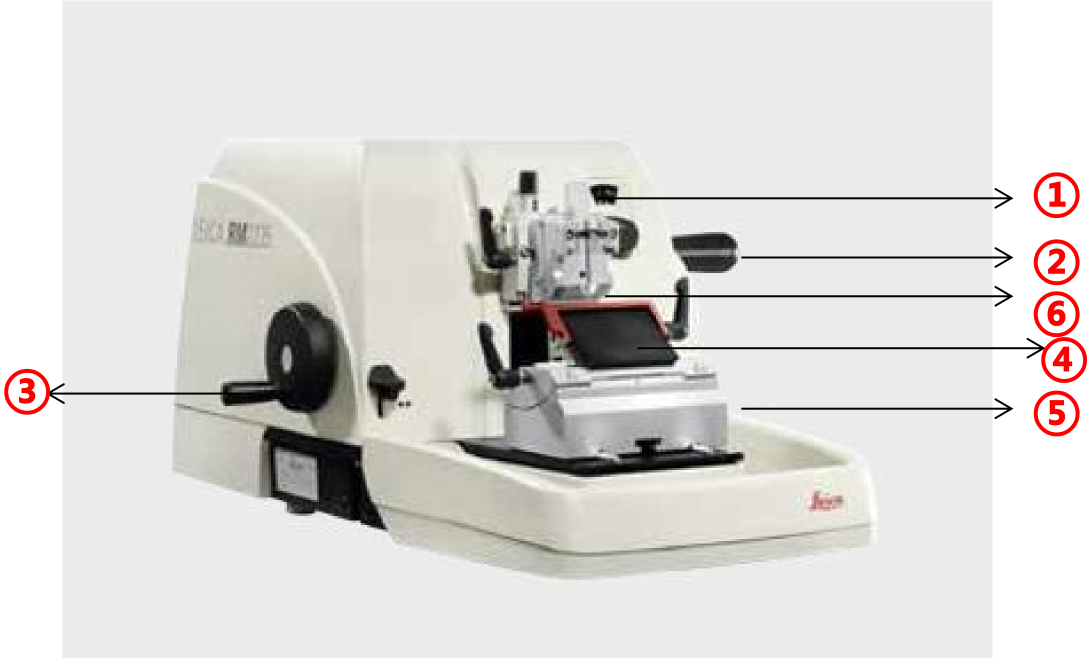

①通用样品头

②平滑旋转手轮

③粗修轮

④刀架

⑤刀架底座

⑥护刀器

石蜡切片机

3.操作步骤：

(1)切片前三查看：查看刀架的角度是否为5度；查看刀架底座安装位置的刻度是否在“2.5”；查看样品头和刀架是否保持有安全距离。

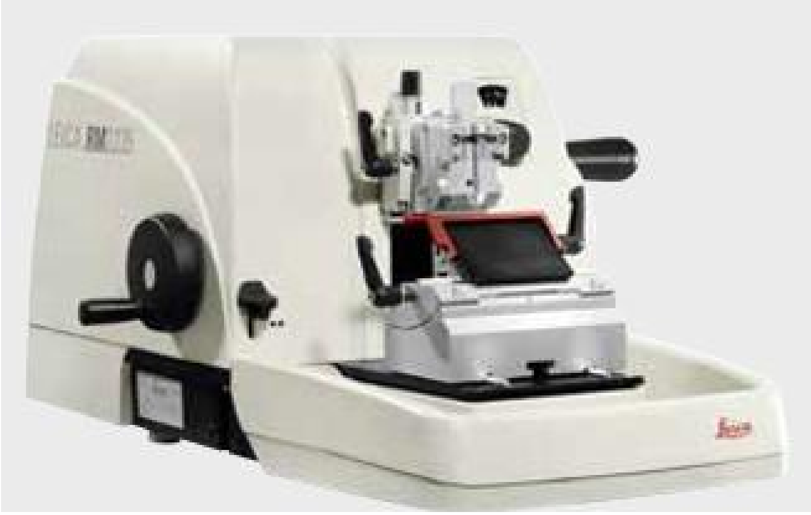

(2)安全工作口诀:

安蜡块-摇手轮，护刀，安蜡块；

取蜡块-锁手轮，护刀，取蜡块；

扫机器-摇手轮，取刀片，扫机器。

石蜡切片机

4.维护和保养：

⑴机器使用完毕后，清理组织碎屑（拆开刀架清理），除蜡去污剂深度清洁。

⑵样品头可定期用热水冲洗，但必须晾干或者烘干（最高65度），但不可用热水浸泡。也可烤箱烘烤，趁热放入二甲苯中彻底除蜡。

石蜡切片机

5.常见问题及原因分析：

(1)安全问题：

安放或取下蜡块时没有盖上护刀器，导致手指割伤。

打扫机器没有去除刀片，导致手指割伤。

(2)打扫机器后，刀座的安放位置在样品头的下方，导致刀座磕碰有缺口，影响切片的精确度。

(3)拧螺杆力气过大，导致小部件损坏，不能卡紧刀片。

(4)样品头积蜡多，无法卡紧蜡块，切片跳刀。

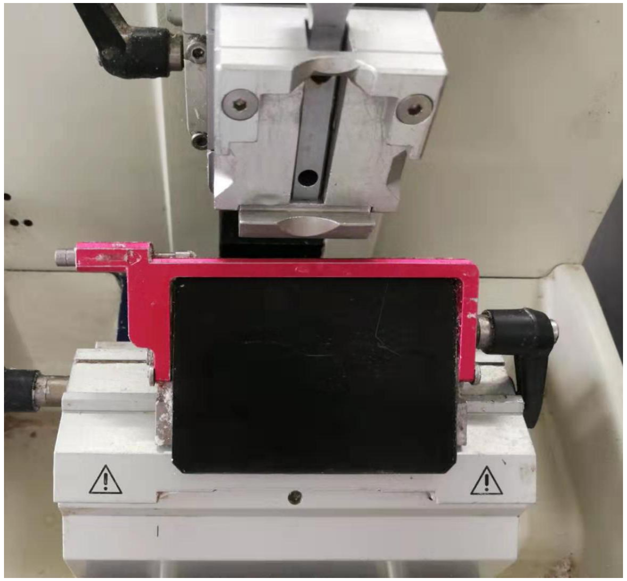

冰冻切片机

包括以下几个方面：

1.设备型号

2.设备组件

3.操作步骤

4.维护和保养

5.常见问题及原因分析

冰冻切片机

1.设备型号：

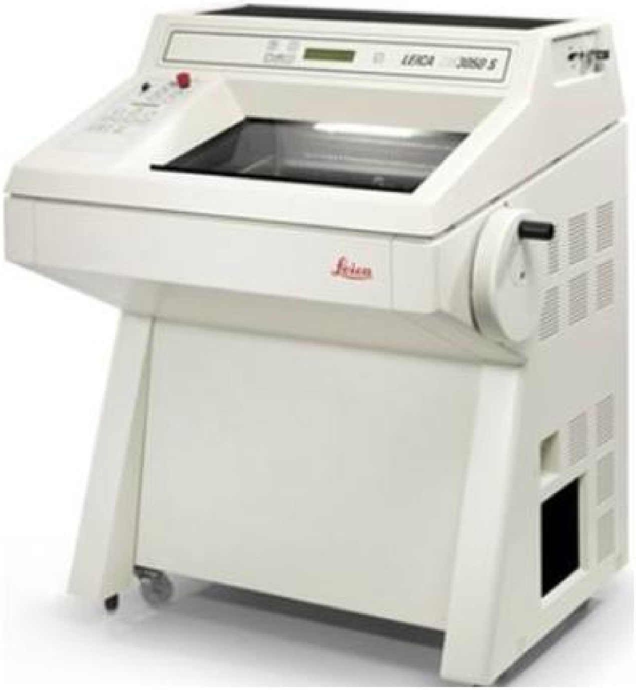

徕卡 CM3050S

冰冻切片机

2.设备组件：

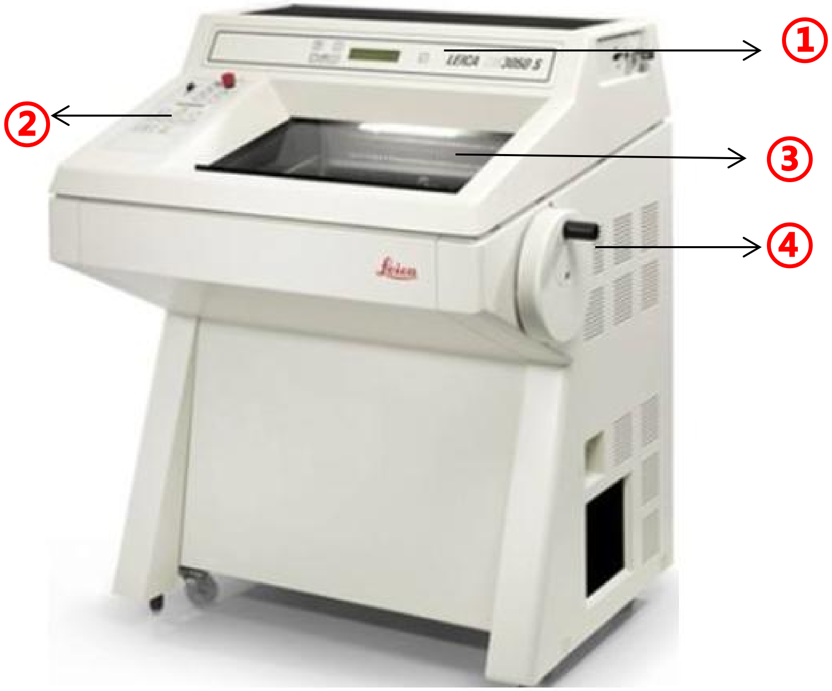

①主控制面板：调节箱体温度和样品头的温度。

②辅控制面板：调节切片厚度；调节粗修厚度。

③样品头

④平滑旋转手轮

冰冻切片机

3.操作步骤：

(1)切片前三查看：查看机器是否处于开机状态；查看控制面板上显示的箱体温度是否正常。查看毛笔镊子、样品托有无提前放于箱体内预冷。

(2) 安样品托：摇手轮，护刀，安样品托；

取样品托：锁手轮，护刀，取样品托；

扫机器：摇手轮，取刀片，扫机器。

(3)使用完毕后查看玻璃门是否关闭。

(4)下班时不要关机。

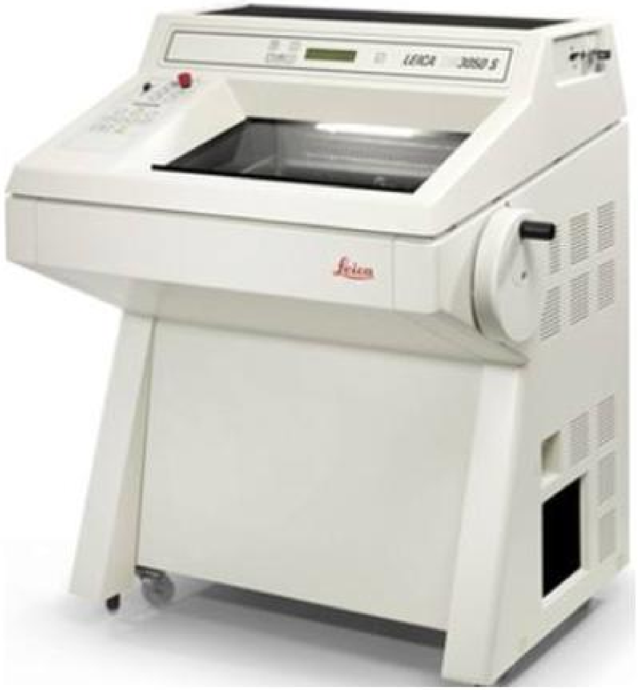

冰冻切片机

4.维护和保养：

(1)定期除霜。

(2)定期关闭机器，彻底清理残存的碎屑。

冰冻切片机

5.常见问题及原因分析:

(1)安全问题:

安放或取下蜡块时没有盖上护刀器，导致手指割伤。打扫机器没有去除刀片，导致手指割伤。

(2)样品夹内组织碎屑过多，导致样品夹不能灵活调节方向。

(3)下班后关机，第二天开启后箱体温度恢复至少需要两个小时。

(4)下班后机器的玻璃门没有完全关闭，机器一直处于化霜状态，导致机器温度无法下降，不能正常使用。

自动染色封片机

自动染色机

包括以下几个方面：

1.设备型号

2.设备组件

3.操作步骤

4.维护和保养

5.常见问题及原因分析

自动染色机

1.设备型号：

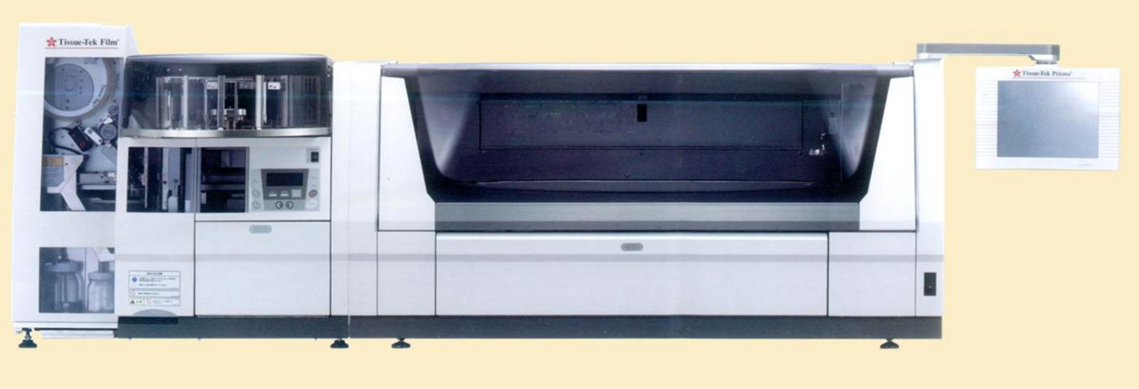

樱花染封一体机

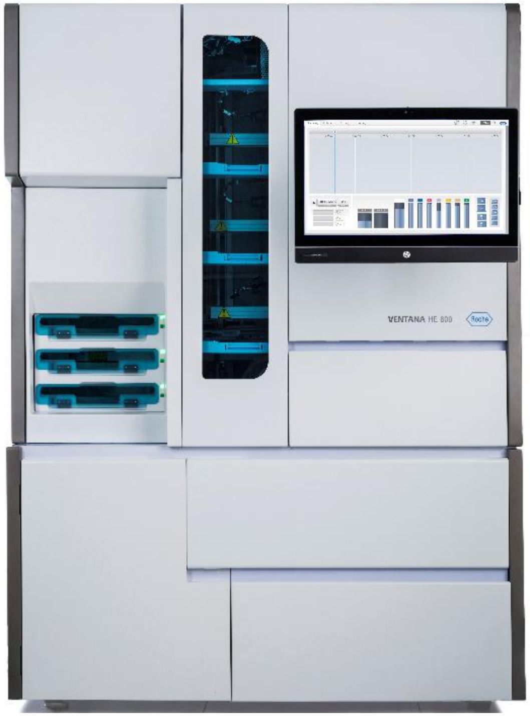

罗氏HE600

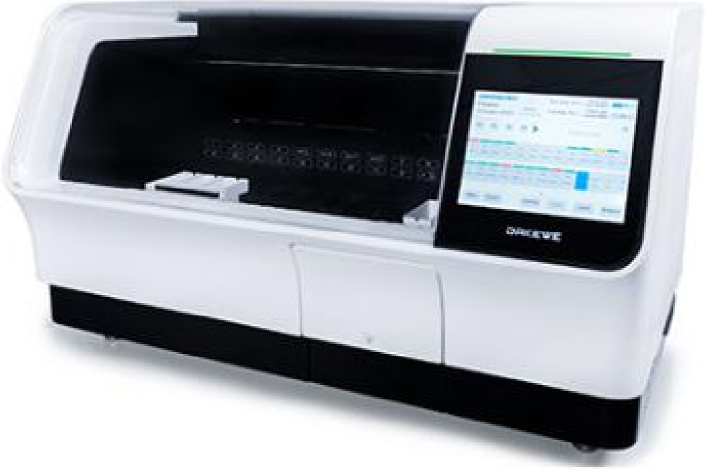

达科为染色机

自动染色机

2.设备组件：

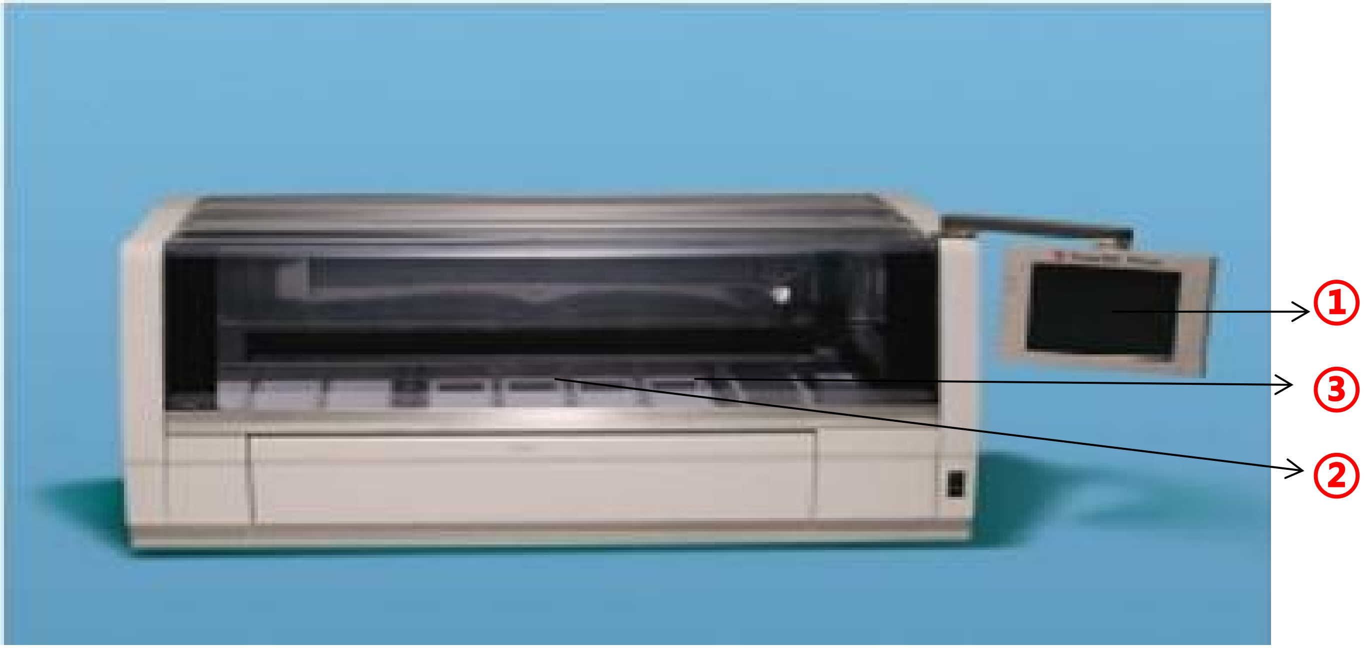

①触摸屏

②药液槽

③干燥槽

自动染色机

3. 操作步骤：

(1)染色前三查看：查看试剂缸的盖子是否全部去除；查看药液槽的试剂情况：是否有漏加试剂，是否有试剂量少，苏木素是否有氧化膜等；查看染色架切片方向。

(2)选择对应的程序开始染色。

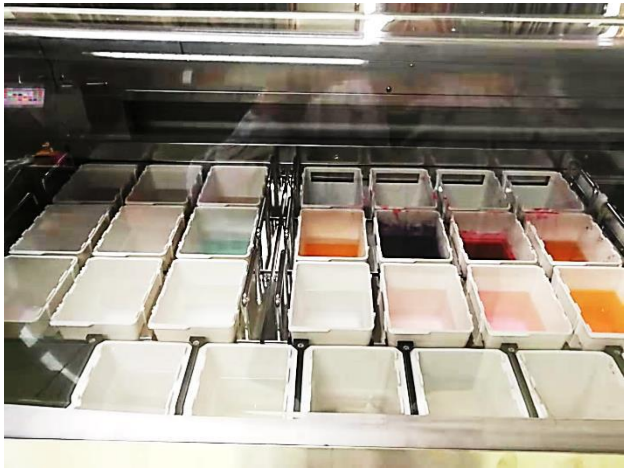

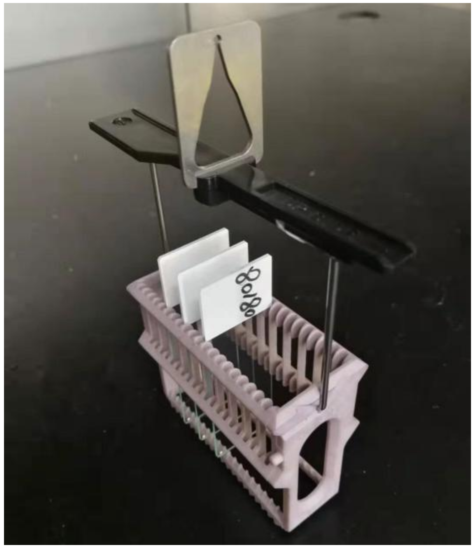

自动染色机

4. 维护和保养：

(1)每天的维护：用软布擦拭机器表面。

(2)每月一次的维护：干燥槽应每月清洁一次。药液槽和染色架应该在水龙头下用温和清洁剂每月清洗一次。定期清洁有助于保持这些部件无永久性的污渍。

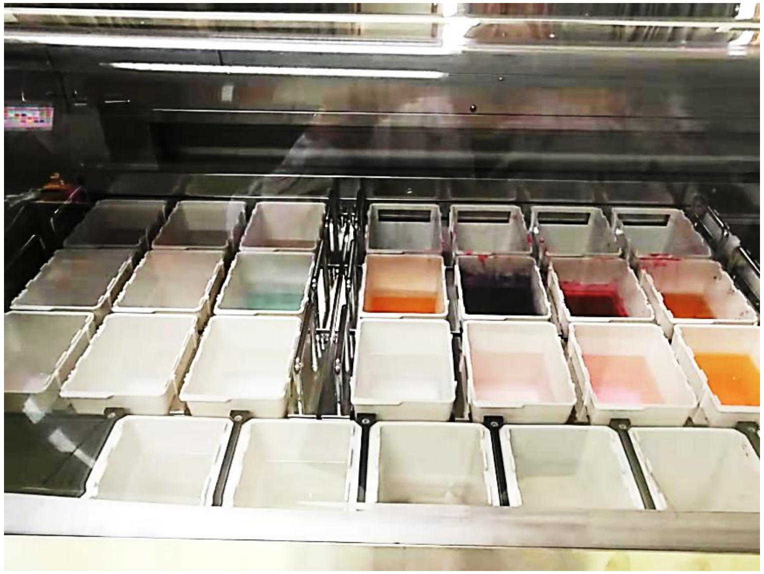

自动染色机

5. 常见问题及原因分析：

(1)染色前，没有完全去除试剂缸的盖子，导致染色架损坏。

(2)染色前没有查看药液槽的试剂情况，比如漏加试剂，导致染色失败。

(3)染色架贴了标记，机械臂不识别，染色失败。

(4)染色架切片方向插反，导致组织处未封片。

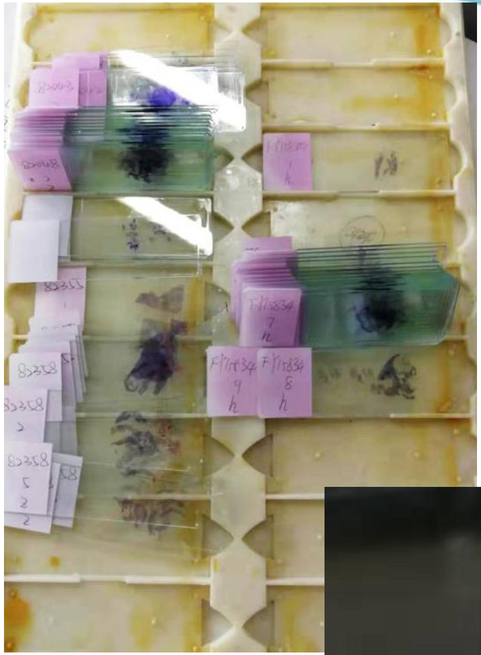

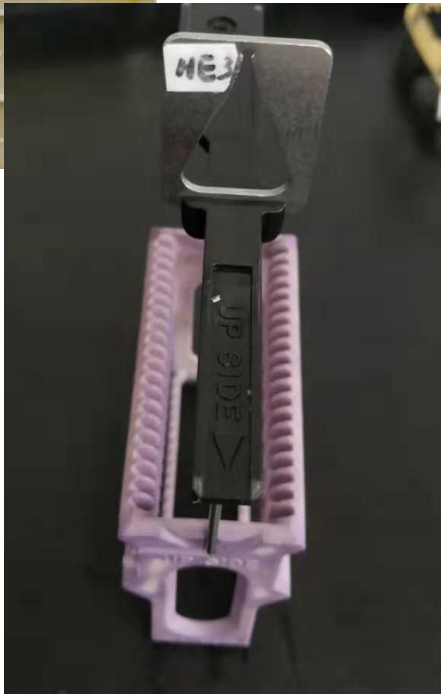

自动封片机

包括以下几个方面：

1.设备型号

2.设备组件

3.操作步骤

4.维护和保养

5.常见问题及原因分析

自动封片机

1.设备型号：

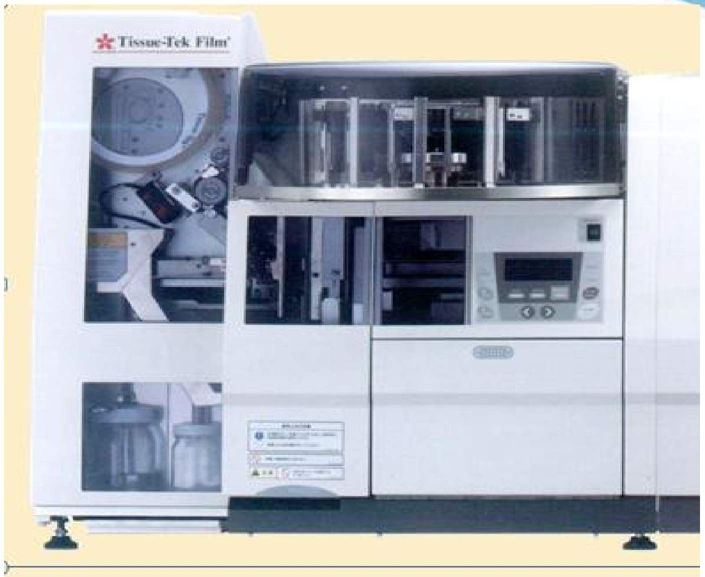

樱花Tissue Tek Film™

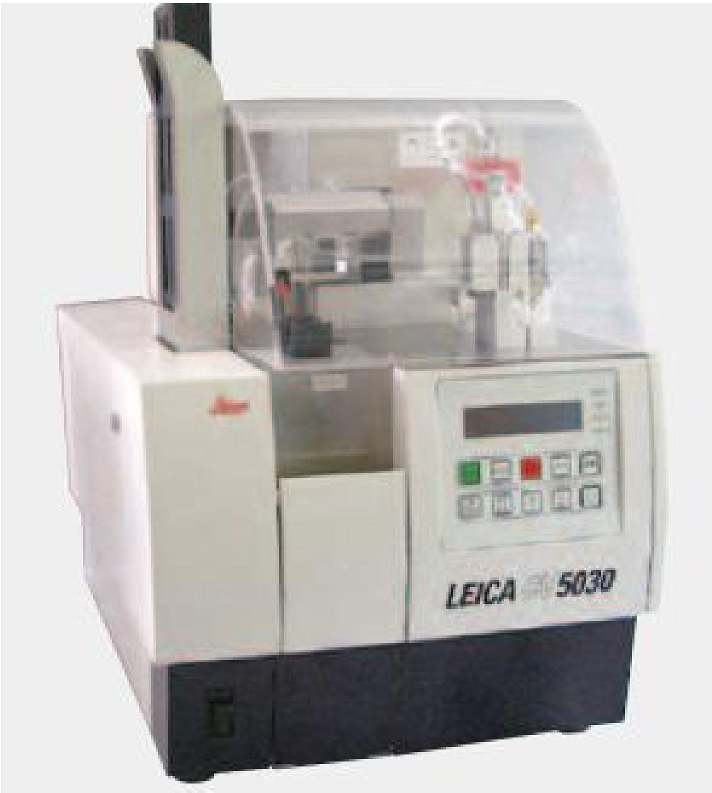

徕卡 CV5030

自动封片机

2.设备组件：

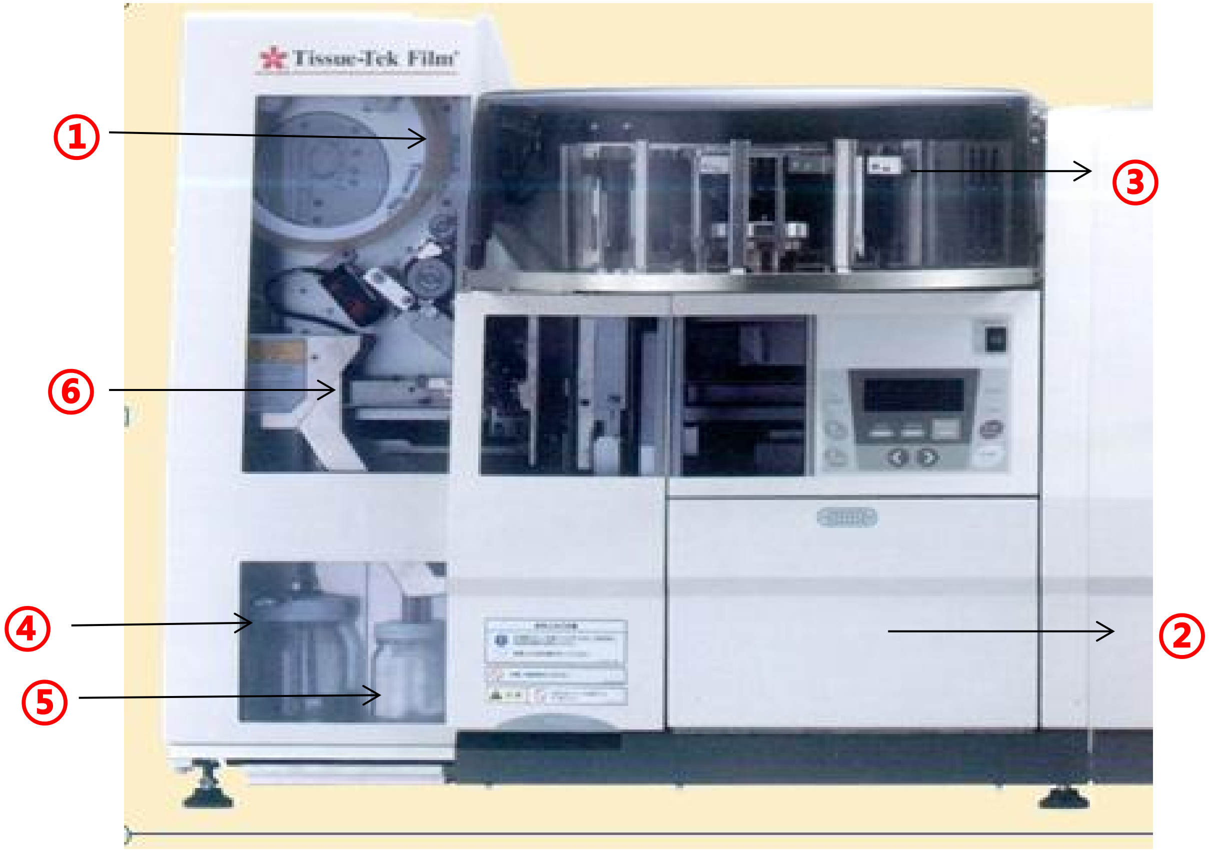

①胶带

②加载槽（里面为装载抽屉）

③收纳框

④二甲苯瓶

⑤废液瓶

⑥胶带碎片接收器

自动封片机

3.操作步骤：

(1)封片前三查看：查看封片机胶带的方向，带字母为正；查看装载抽屉二甲苯是否充足；查看染色架提篮的方向是否正确。

(2)操作界面显示“STANDBY “既可开始封片。

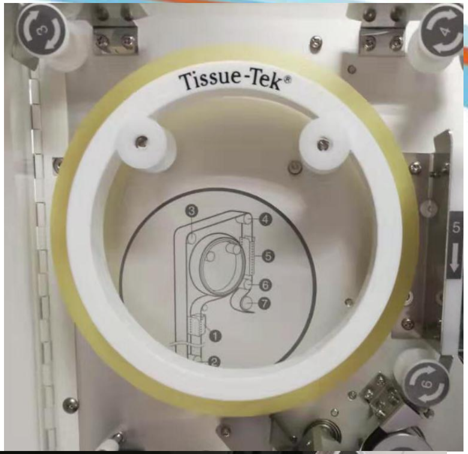

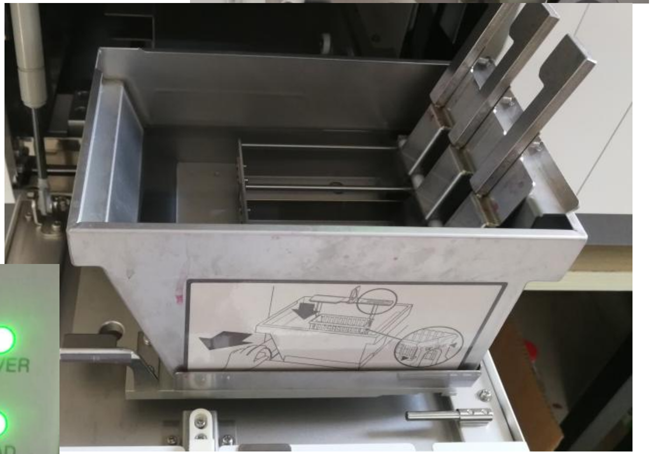

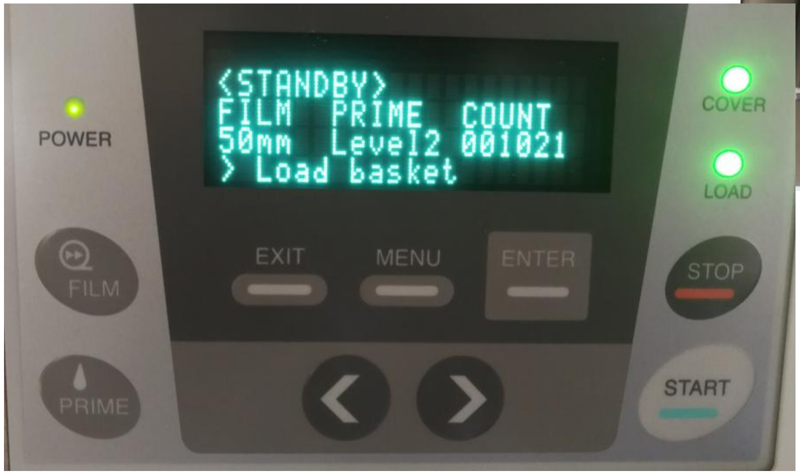

自动封片机

4.维护和保养：

(1)用软布擦拭机器的表面。

(2)每天要倒掉装载抽屉的二甲苯，第二天换新。

(3)胶带碎片接收器定时清空。

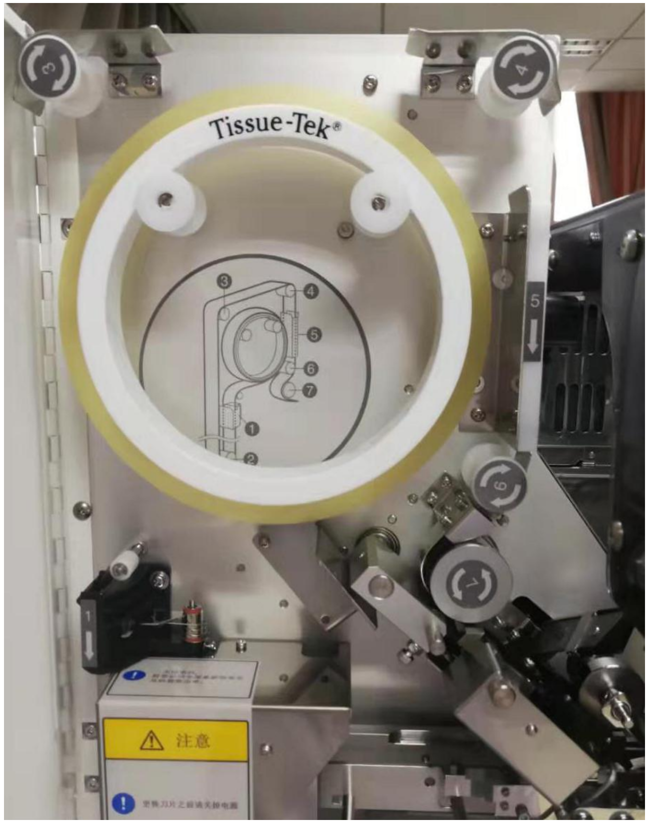

自动封片机

5.常见问题及原因分析：

(1)胶带安装错误，字母面朝内，封片失败。

(2)装载槽二甲苯过少，片子干封。

(3)随意改变染色架提蓝方向，封片中止。

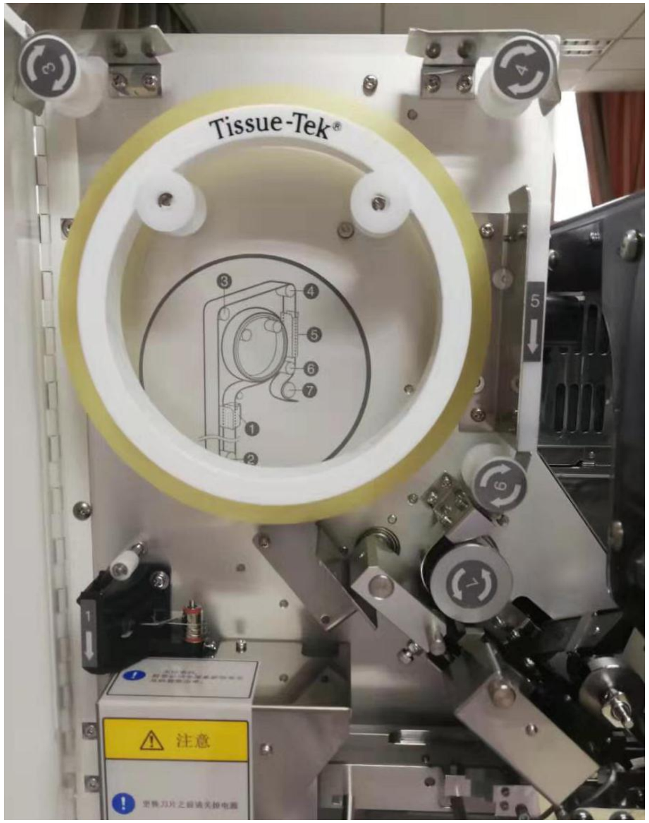

罗氏染封一体机

1.设备型号：罗氏HE600

2.设备组件：

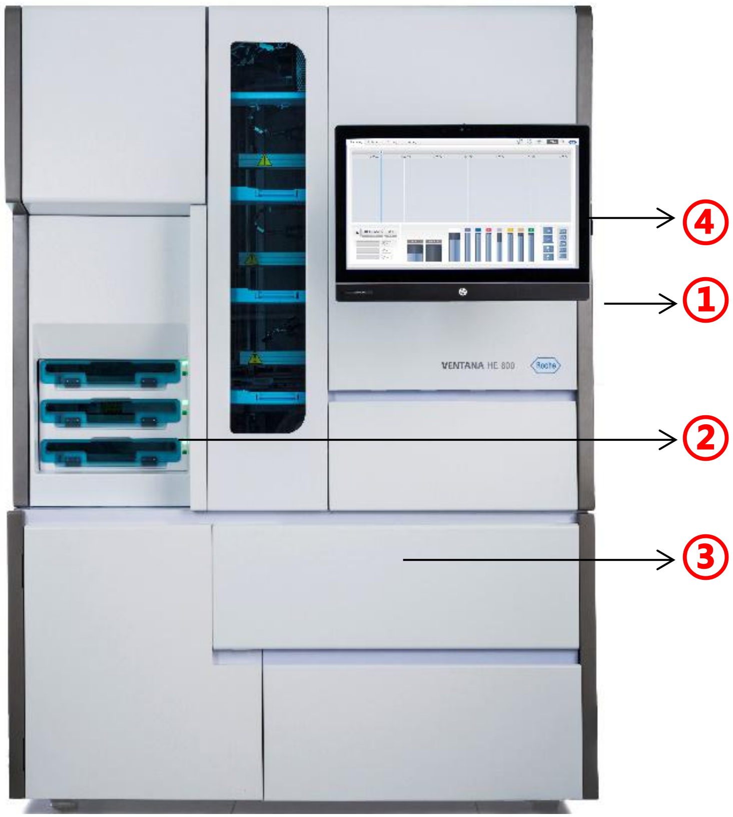

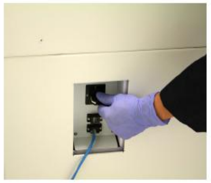

①电源开关

②托盘入口

③试剂仓门

④控制面板

罗氏染封一体机

3. 操作步骤：

(1)开机操作:逆时针旋转仪器左侧的电源开关旋钮，仪器启动并初始化，自动进入HE600操作界面。

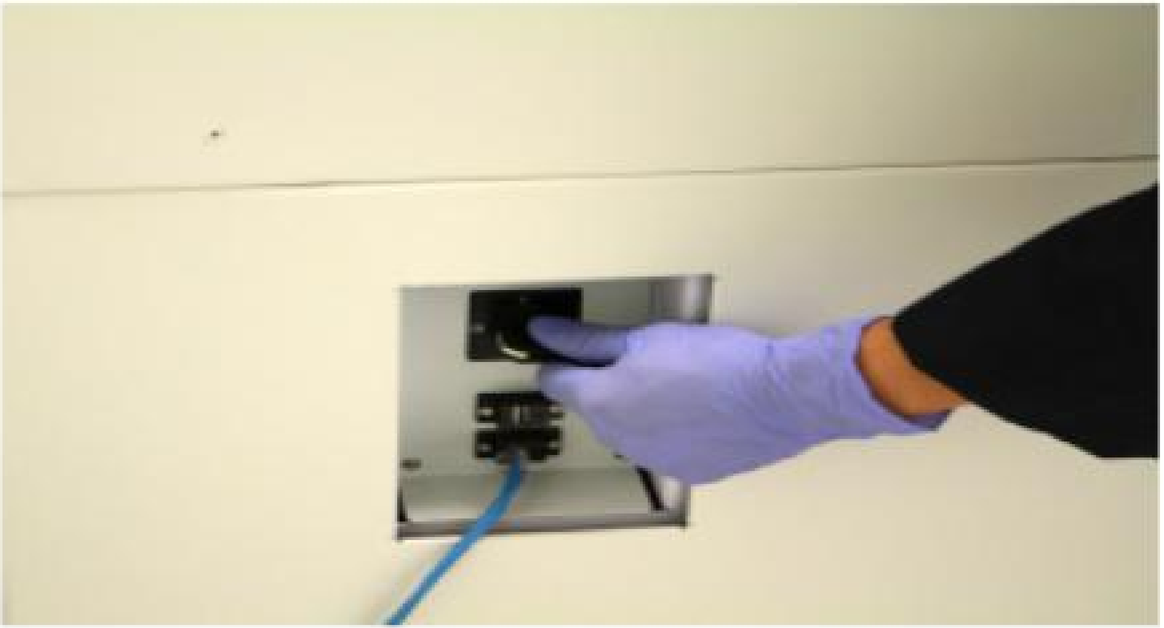

罗氏染封一体机

⑵关机操作：当天染色结束后，在Instrument界面点击Stop，机器停止运行，然后再点击Shut Down，提示框出现，点击“Yes”，继续关闭机器。仪器会在关闭之前，对苏木素管路进行清洗，清洗结束后，操作软件自动关闭。最后手动关闭电脑，最后顺时针旋转仪器左侧旋钮，关闭仪器。

罗氏染封一体机

(3)染色操作:点击左下角 ⏏ 图标，托盘入口打开后，迅速放入托盘（按照托盘上指示箭头放置）。在上述图标处可以显示下一个托盘的染色结束时间；可以显示仪器中托盘的数量，最多可以放满10个托盘；还能显示可以使用的托盘入口。在控制面板试剂状态区，如果某类试剂显示红色，代表该试剂已用完，需要更换新的试剂。

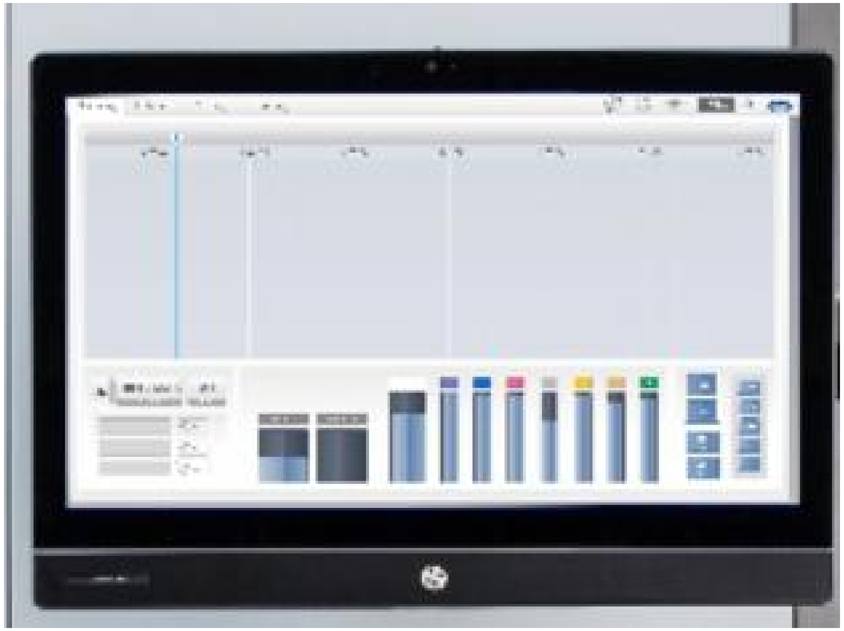

罗氏染封一体机

4. 维护和保养：

⑴日保养：清空盛放废盖玻片的槽；擦拭试剂桶架与仪器接口处的残留试剂。

⑵每日苏木素管路清洗：关机时，点“shut down”后，会弹出管路保养信息，点击“Yes”后，管路清洗自动开始。仪器关闭后，管路充满清洗液并浸泡1h。

⑶半年保养：由硬件工程师完成。包括整台仪器检测和苏木素管路的保养维护。后者包括储液罐和管路的清洗和过滤器的更换。

罗氏染封一体机

5. 常见问题及原因分析：

未按照要求关机，第二天机器启动要先清洗管路，影响染片。

提 纲

1. 包埋机
2. 切片机
3. 染色机及封片机
4. 全封闭组织脱水机

全封闭
组织脱水机

全封闭组织脱水机

包括以下几个方面：

1.设备型号

2.设备组件

3.操作步骤

4.维护和保养

5.常见问题及原因分析

全封闭组织脱水机

1.设备型号：

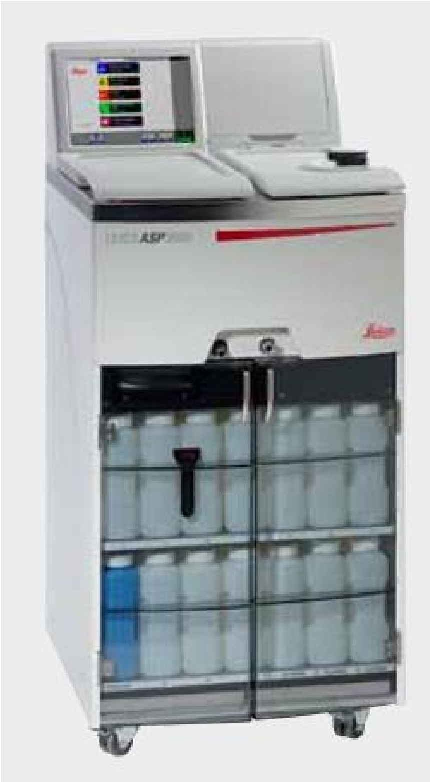

徕卡ASP300S

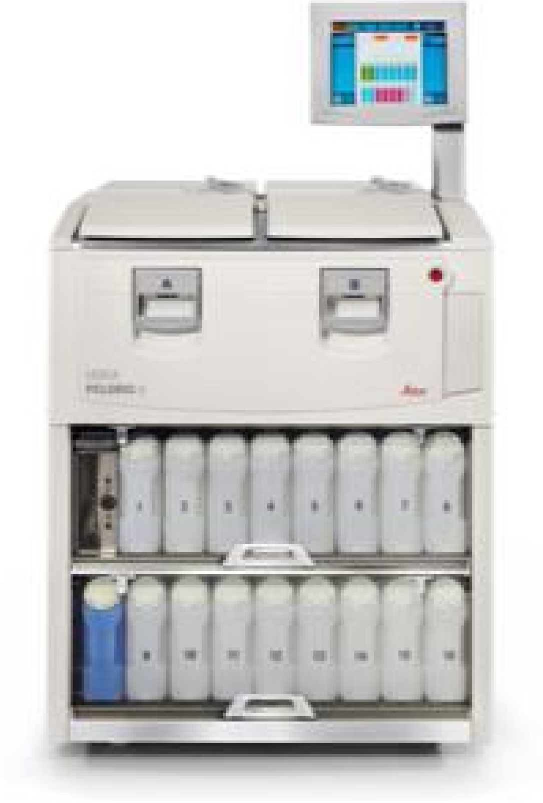

徕卡Leica PELORISⅡ

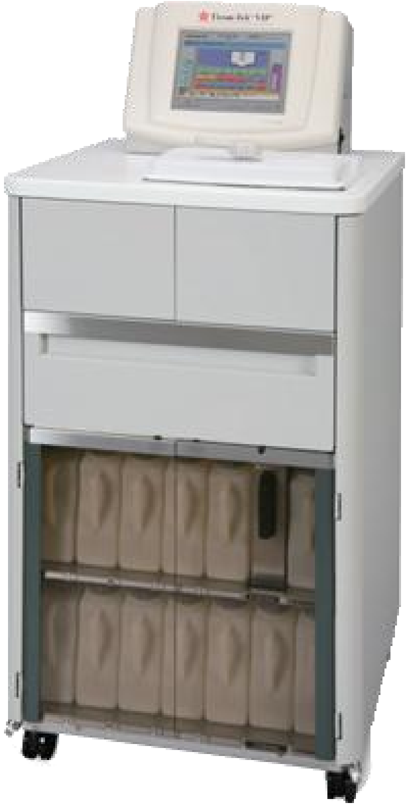

樱花VIP6

全封闭组织脱水机

2.设备组件：

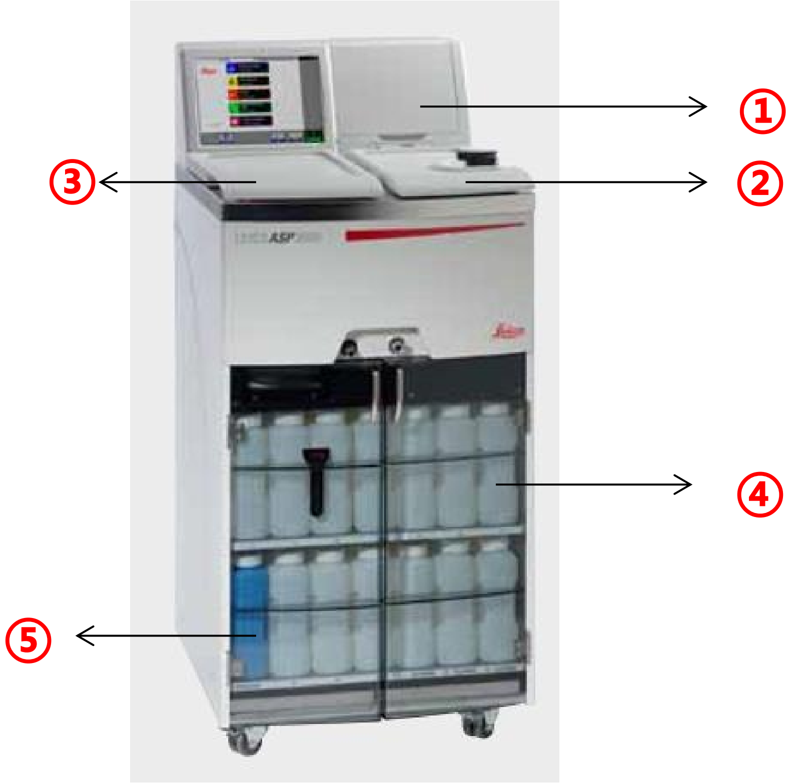

徕卡ASP300S

①控制面板

②脱水缸

③蜡缸（3.5L)

④试剂瓶

⑤冷凝瓶

全封闭组织脱水机

3.操作步骤：

(1)挂机前五查看一擦拭：查看机器有无清洗；查看蜡缸石蜡的量；查看试剂瓶内试剂的量；查看试剂瓶的位置；查看脱水缸密封圈。用二甲苯擦拭探头。

(2)控制面板选择对应的脱水程序。

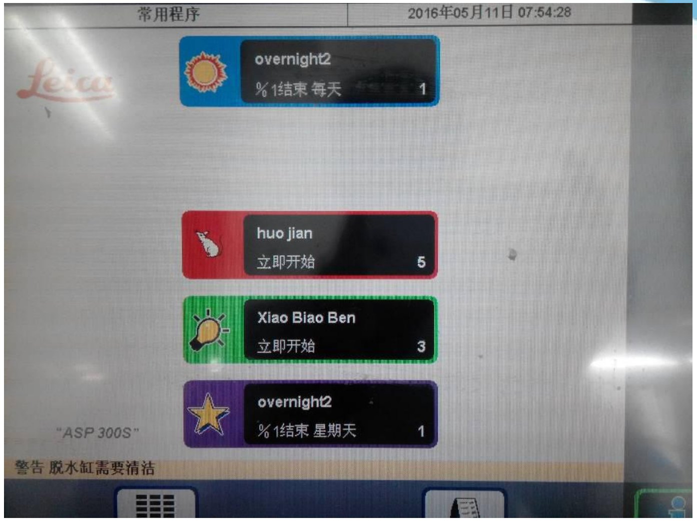

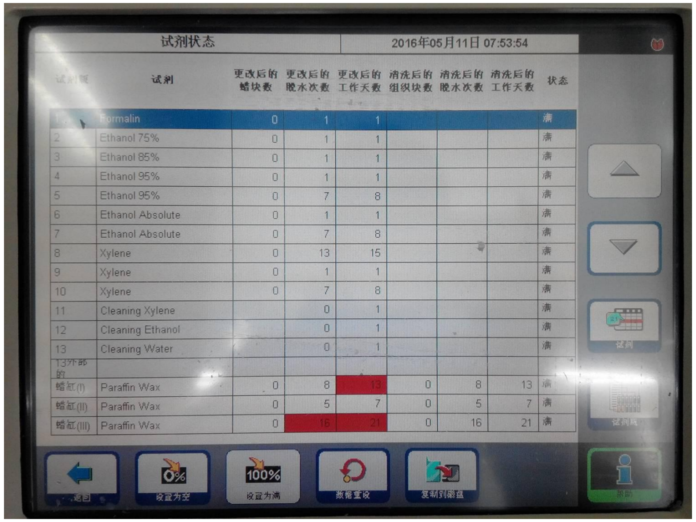

全封闭组织脱水机

4.维护和保养：

机身清洁：每次使用完机器用清洁塑料铲清除设备表面滴落的石蜡。

密封清洁：清洁密封圈及其密封位置的余蜡；清洁脱水缸密封接触面的余蜡。

气孔的检查：加蜡时排查是否有异物堵塞气孔，并及时清理。

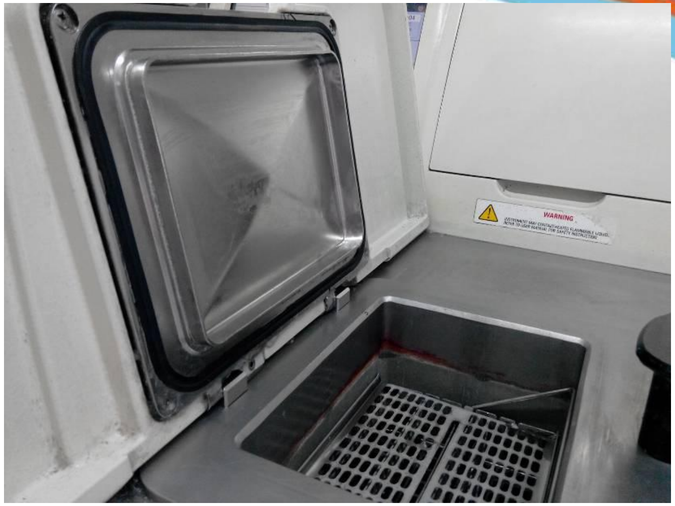

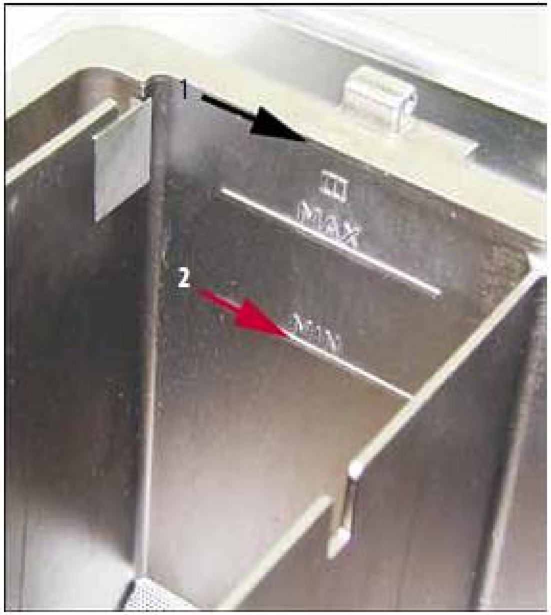

全封闭组织脱水机

5.常见问题及原因分析：

(1)显示器程序选择错误导致脱水结束时间提前或延迟。

(2)脱水缸密封圈脱落导致程序中途中止报警。

(3)蜡缸石蜡液面低于最低位置导致程序中途中止报警。

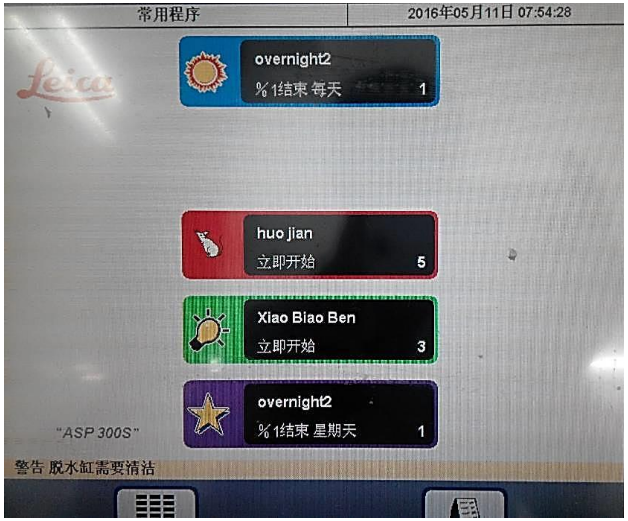

Supplement: Multimedia Appendix 2 [file jmir_v23i7e23799_app2.pdf]
