# Supplementary material for: Effectiveness of a Cloud-Based Telepathology System in China: Large-Sample Observational Study
Source: J Med Internet Res. 2021 Jul 29;23(7):e23799. doi: 10.2196/23799 (PMC8367172; doi:10.2196/23799)
Supplement: Multimedia Appendix 3 [file jmir_v23i7e23799_app3.pdf]

第九章 病理诊断系统的设计及其应用

Design and application of telepathology system

9.1 引言

随着网络信息技术的快速发展，远程医疗平台已逐渐成为现代医学不可缺少的一个平台。其中远程医疗平台病理诊断系统，可对数字切片进行远程诊断或会诊，申请会诊单位将制成的数字切片和相关病例资料打包，通过该系统选择国内外著名病理学专家，系统将数字切片和相关临床病理资料自动上传到会诊平台，专家登录平台，通过浏览器进行数字切片浏览、分析和诊断，并发送病理咨询诊断报告。

1.1.1 编写目的

河南省远程医疗云平台病理诊断系统将传统切片进行数字化，集成显微影像处理、Web 图像浏览等技术，整合多年的病理领域经验、专家资源，为广大病理医生与患者，提供省时、省力、便捷、快速的专家咨询服务，为全省乃及全国甚至全球病理医生，提供无时间与空间限制的数字切片交流机会。改变了原来传统就诊的单一模式，改善了专家资源分布不均衡的现状，让更多的边远患者及医护人员更经济、高效地共享专家资源、教育资源、医学科技成果资源等医疗资源，提高了边远地区的医疗技术水平，使医学领域的高科技真正成为人类的福音。

本章节详细论述了河南省远程医疗云平台病理诊断系统的系统设计、系统应用操作说明、常见问题及其解决方案等，用于指导病理医生、技术维护人员等相关人员正确使用河南省远程医疗云平台病理诊断系统。

1.1.2 阅读对象

本章节的阅读对象为接触、使用河南省远程医疗云平台病理诊断系统的相关人员，例如病理医生、技术维护相关人员等。

9.2 系统设计

1.2.1　系统设计目标

远程医疗云平台病理诊断系统设计的主要目标是将系统分析阶段提出的反映用户信息需求的系统逻辑方案转换成可以实施的基于计算机与通信系统的物理（技术）方案。

从系统的总体目标出发，基于系统分析阶段对系统逻辑功能的要求，并结合考虑经济、技术和运行环境等各方面的条件，确定系统的总体结构和系统各组成部分的技术方案，合理选择计算机和通信的软、硬件设备，提出系统的实施计划，确保总体目标的实现。

1.2.2　系统设计原理

本系统采用了 B/S 结构（Browser/Server，浏览器/服务器模式）。这种模式统一了客户端，将系统功能实现的核心部分集中到服务器上，简化了系统的开发、维护和使用。客户机上只要安装一个浏览器，服务器安装 SQL Server、SQLite 等数据库。浏览器通过 Web Server 同数据库进行数据交互。

本系统具有维护和升级方式简单、成本降低、选择更多、应用服务器运行数据负荷较重等特点。系统建立在广域网之上，不必是专门的网络硬件环境，例如电话上网、租用设备等，信息由自己进行管理，适应范围比 C/S 更强，一般只要有操作系统和浏览器即可满足要求。

1.2.3　系统设计架构

（1）　总体功能框图

远程医疗云平台病理诊断系统总体可分为两个用户模块，分别为医院申请端和专家诊断端，主要流程是医院扫描后切片绑定病例资料做会诊申请，由医学平台分配至专家端，专家诊断操作完成后签发报告医院端可下载查看报告，总体系统功能框图如图 9-1 所示。

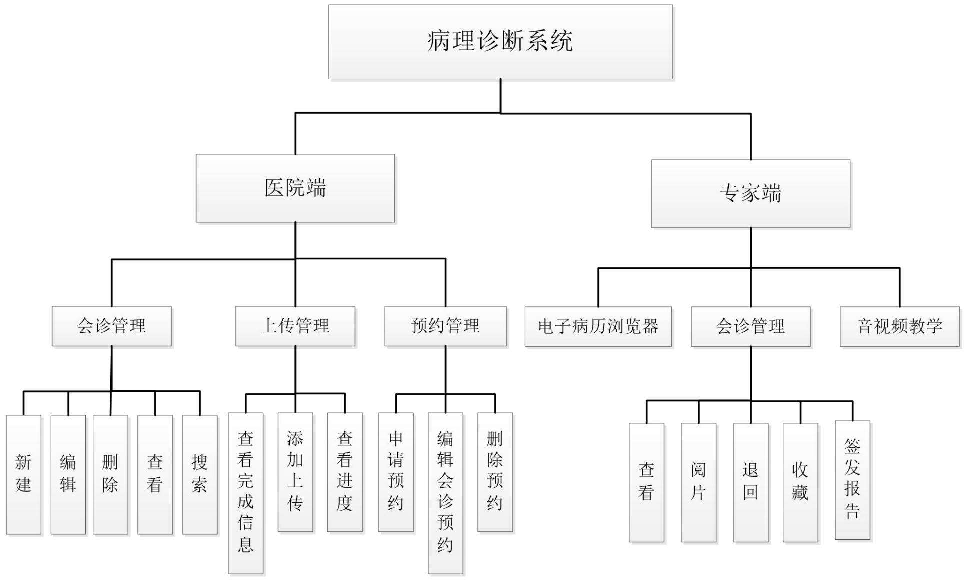

图 9-1 系统功能框图

（2） 技术架构

UI(表现层)：主要是指与用户交互的界面，用于接收用户输入的数据和显示处理后用户需要的数据。

BLL(业务逻辑层)：UI 层和 DAL 层之间的桥梁，实现业务逻辑。业务逻辑具体包含：验证、计算、业务规则等。

DAL(数据访问层)：与数据库打交道。主要实现对数据的增、删、改、查。将存储在数据库中的数据提交给业务层，同时将业务层处理的数据保存到数据库。

远程医疗云平台病理诊断系统的技术架构视图，如图 9-2 所示。

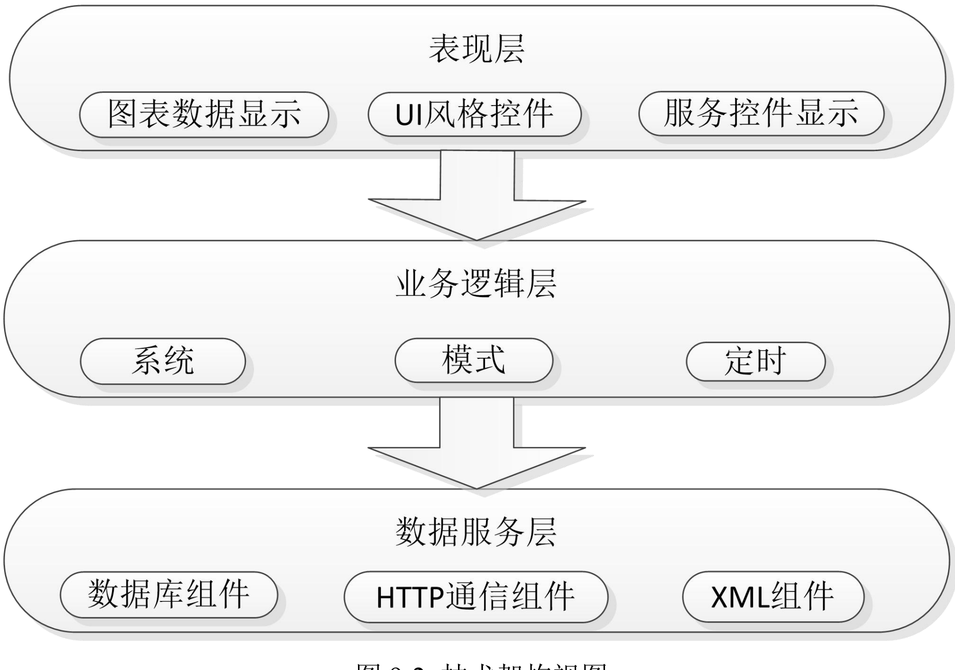

图 9-2 技术架构视图

9.3 系统应用

1.3.1 登录与退出

（1） 用户登录

推荐使用 IE9+、谷歌浏览器等，打开浏览器，在地址框内输入：http://www.htcc.org.cn/，转到河南省远程医学中心网站，点击网站中【病理诊断】按钮进入病例诊断系统，如图 9-3 所示。

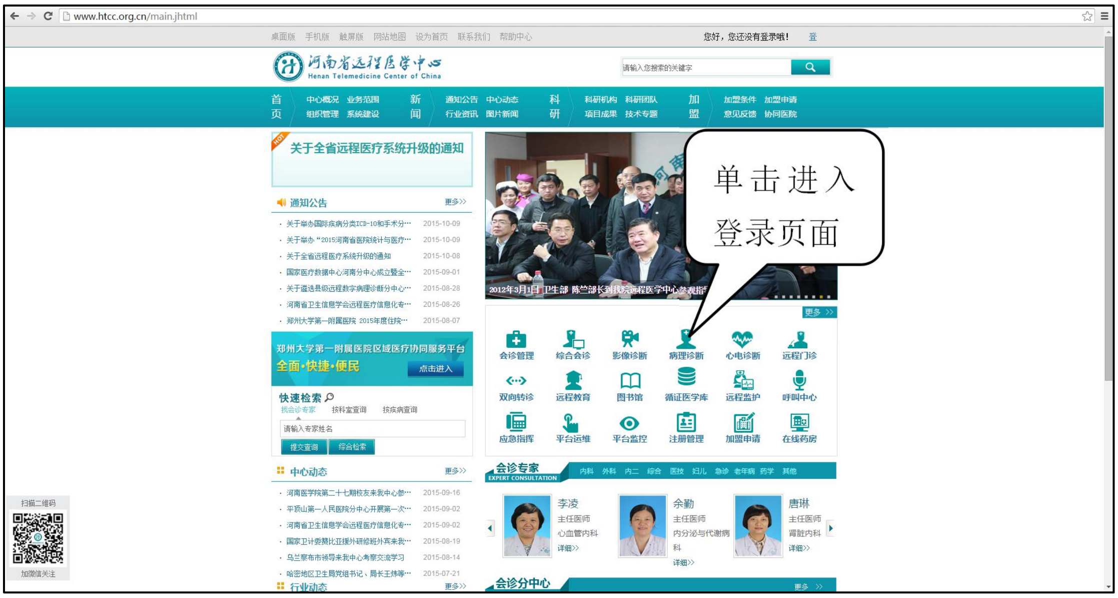

图 9-3 河南省远程医学中心网站

进入系统登录页面，输入帐号、密码、验证码后，点击登录按钮，进入系统，其中可以勾选用户记住按钮方便登录，登录框底部有常见问题的解答可点击查看，登录界面如图 9-4、图 9-5 所示。

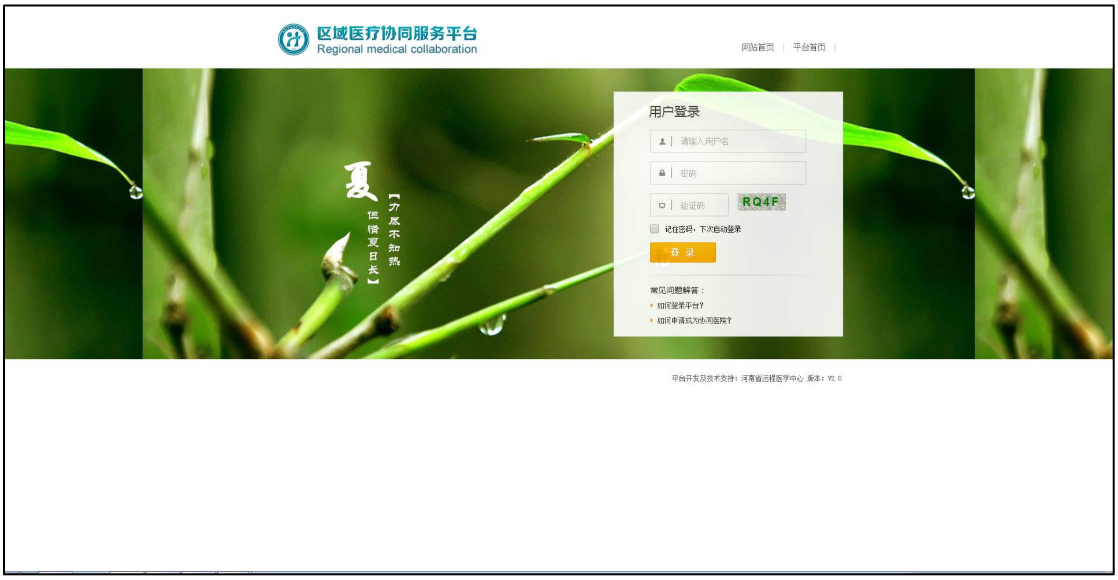

图 9-4 病理诊断系统登录界面

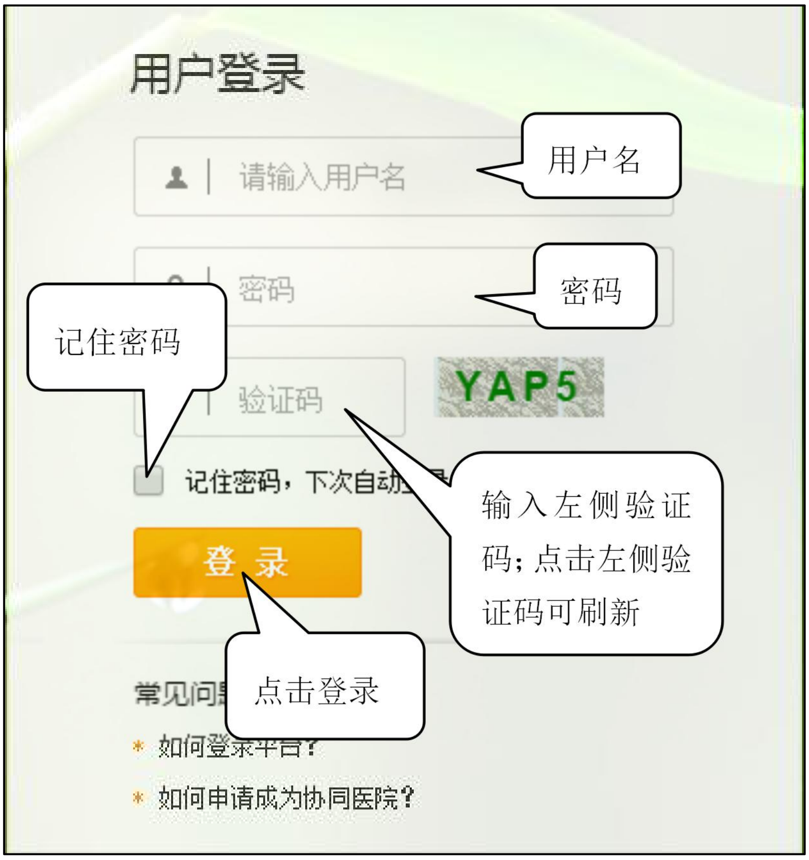

图 9-5 病理诊断系统登录界面局部放大图

（2） 退出系统

用户若要退出系统，点击页面右上角下拉框，可看到【注销】按钮，点击可退出，如图 9-6 所示。

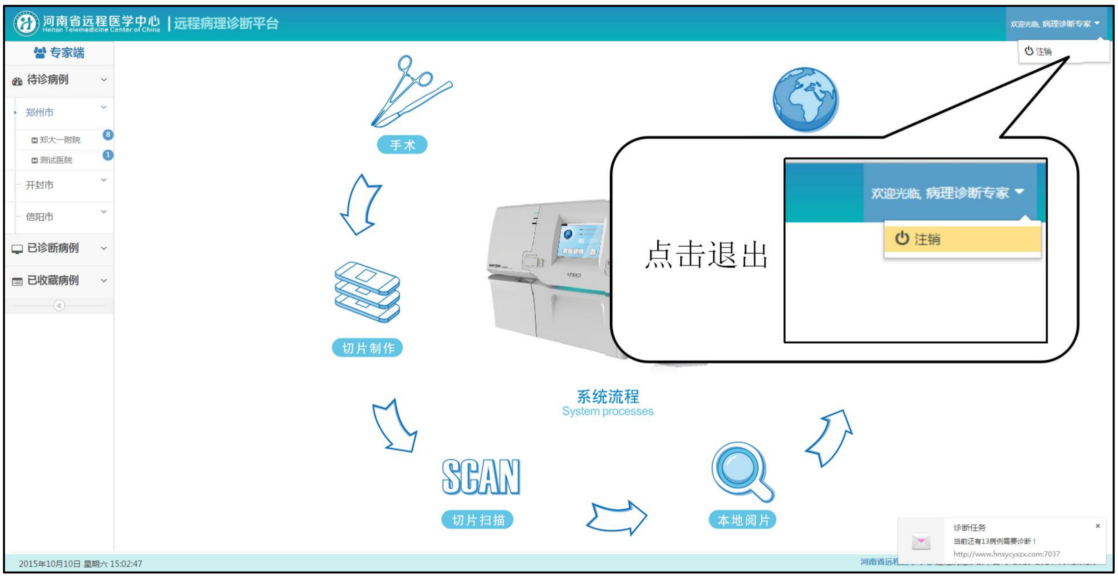

图 9-6 退出系统操作示意图

1.3.2 医院申请端管理

通过扫描软件系统，将传统的玻璃切片进行扫描和无缝拼接，生成包括传统玻璃切片内所有信息，即整张全视野的数字化切片。之后，申请会诊单位将制成的数字切片和相关病例资料打包，通过互联网上传到会诊平台。

医院申请端主页包括左侧导航栏目录、右侧数据及操作界面、上侧信息显示区域和注销退出，具体如图 9-7 所示。

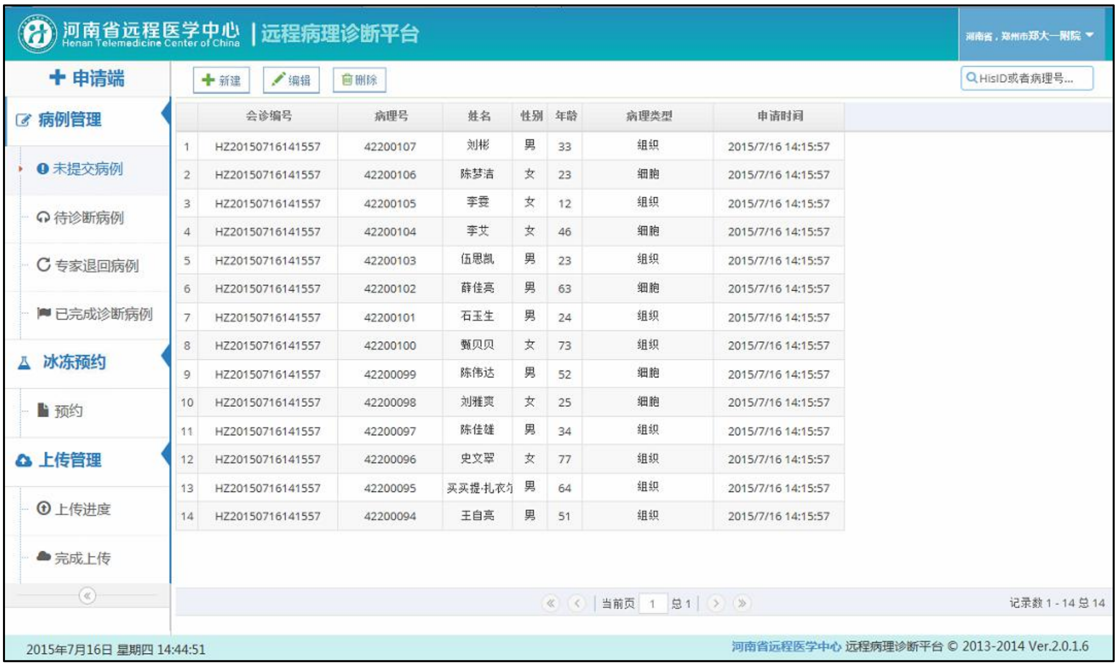

图 9-7 医院申请端主页

（3） 未提交病例模块

① 新建会诊，病历资料录入功能

导航栏选中【未提交病例】，点击【新建】，弹出新建框，选中【病例信息】可进行会诊病例资料录入，如患者基本信息、临床病史、大体所见、免疫组化等，如图 9-8 所示。

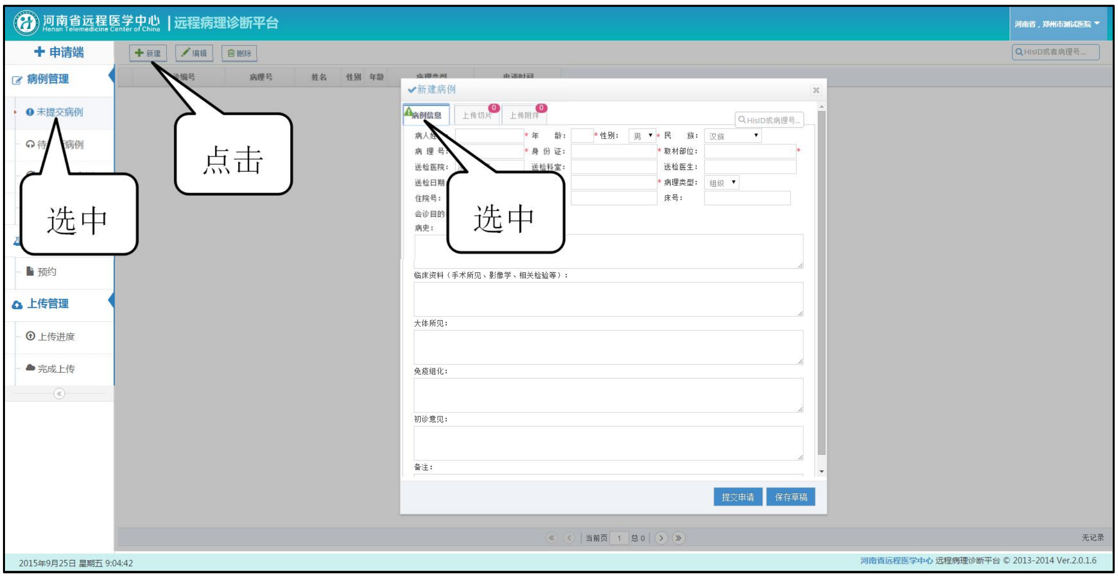

图 9-8 技术架构视图

② 上传切片功能

✓ 加入至上传队列功能

选中【上传切片】，鼠标移动到切片上，点击【+】，可把切片上传到列表中，每个诊断或会诊病例可选择多张切片进行上传，如图 9-9 所示。

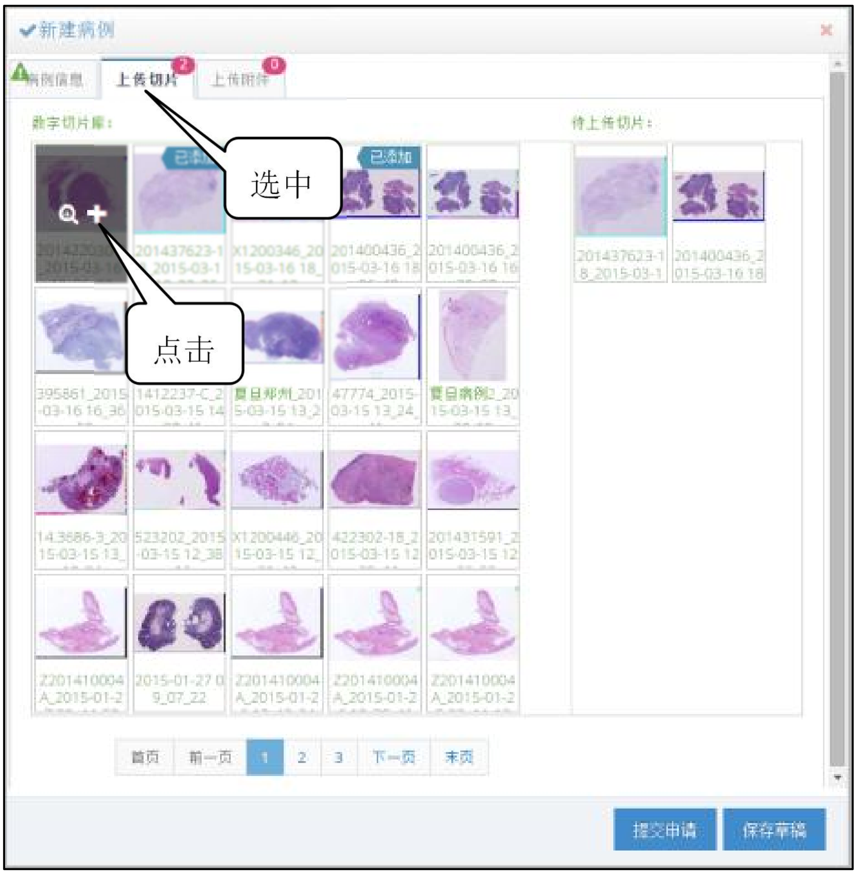

图 9-9 添加上传切片

✓　取消加入上传队列

鼠标移至待上传切片列表，点击【-】移除按钮，即可把切片移除，如图 9-10 所示：

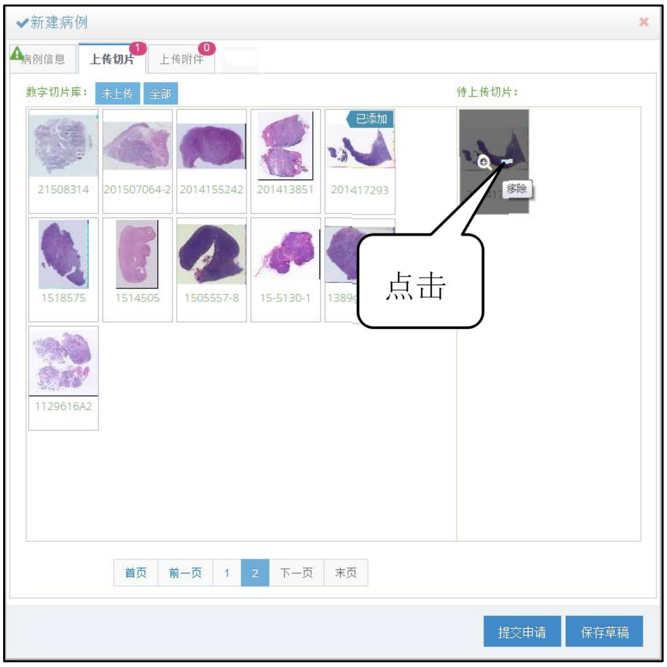

图 9-10 移除上传列表

✓　阅览切片功能

点击【放大镜图标】阅览按钮，如图 9-11 所示。弹出新页面，可阅览切片，如图 9-12 所示。

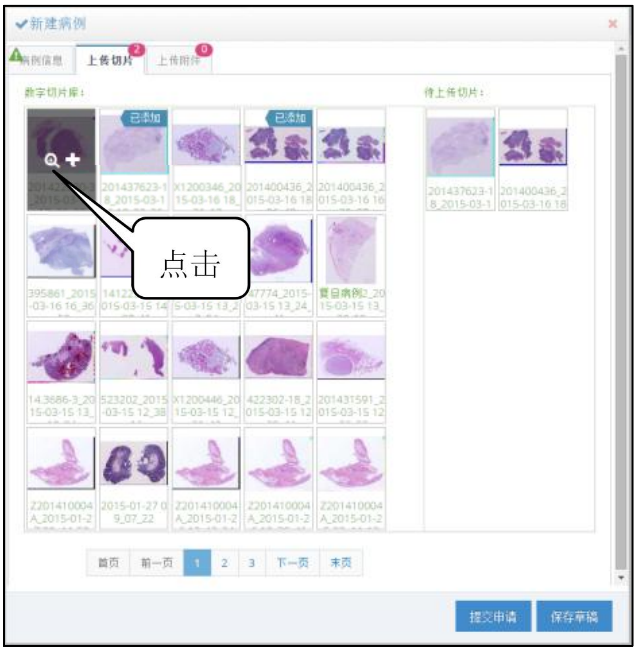

图 9-11 预览切片选择

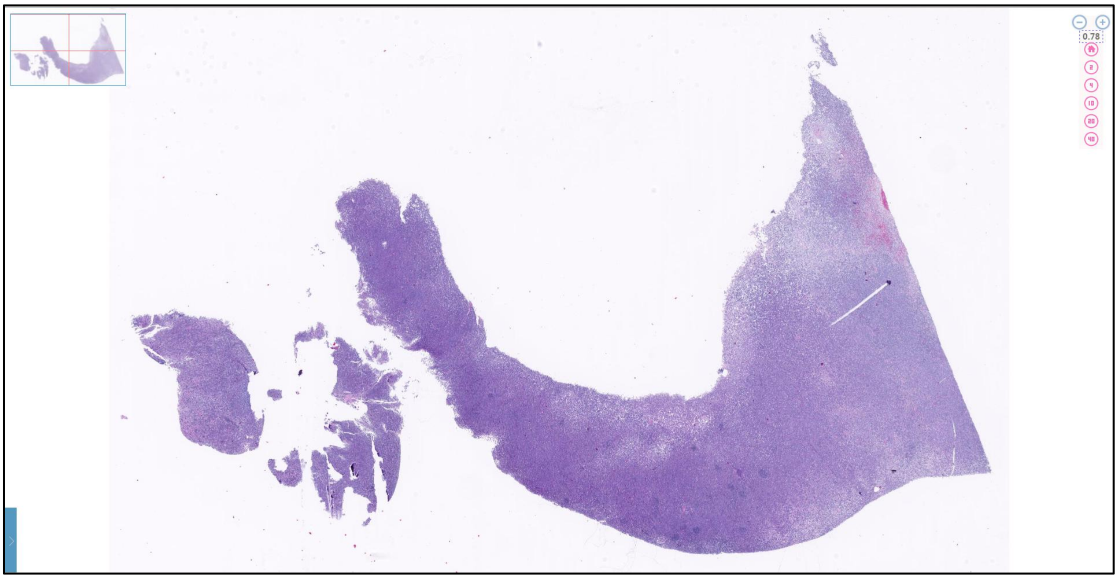

图 9-12 新页面阅览切片

③ 上传附件功能

会诊病例需要上传一些有关附件，在新建病例时可以选择上传附件。列表是由后台限定的，文件筛选后只能上传如.jpg、.pdf 等格式的文件。

✓ 阅览附件功能

打开附件列表，【右键】【浏览】，新页面打开预览附件；或者点击右侧待上传列表【放大镜图标】，新页面打开预览附件。

✓ 加入至上传队列功能

打开附件列表，【右键】【添加上传】，右侧待上传列表中显示该文件信息。

✓ 查看附件信息

右侧显示待上传附件基本信息，包括名称、路径、大小。

✓ 取消加入上传队列

右侧待上传列表，点击【垃圾桶图标】，即可取消上传队列。

具体界面如图 9-13 所示。

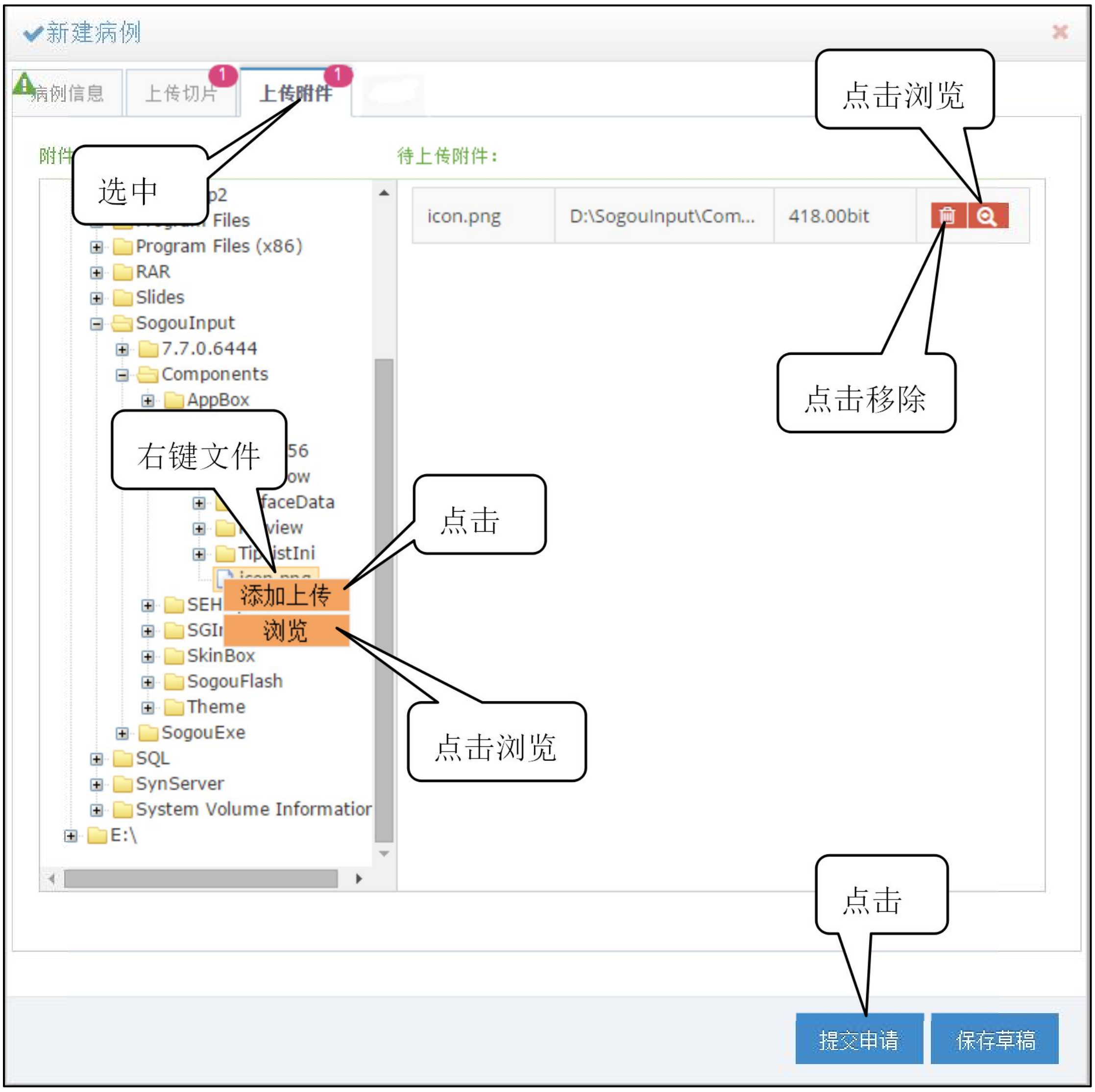

图 9-13 上传附件

④ 编辑和删除操作

病例【保存草稿】后，在未提交病例中，可以对病例信息进行编辑、删除等操作。

✓ 编辑

选中新建时未提交而进行了保存草稿操作的病例并进行编辑，进行病例信息，上传切片，上传等操作，而后选择提交申请则上传至中心，若仍选择保存草稿，则不上传，仍可进行修改。

点击病例，点击【编辑】按钮，弹出编辑框进行编辑操作；或者直接【双击】，病例直接打开，如图 9-14 所示。

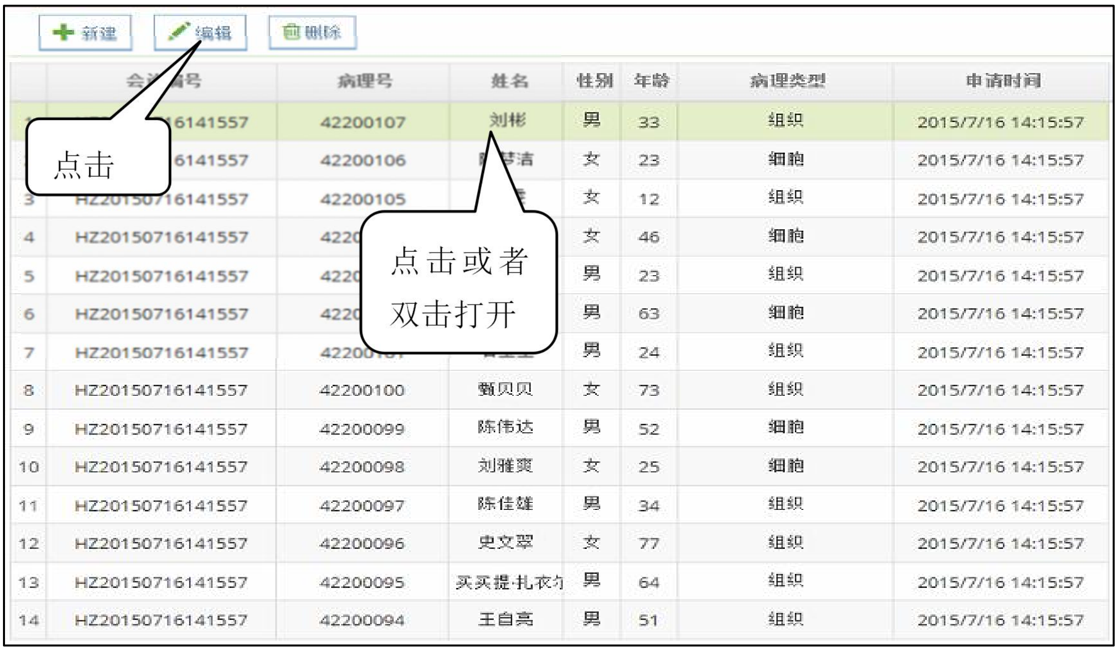

图 9-14 编辑会诊

✓ 删除

选中新建时未提交而进行了保存草稿操作的病例并进行删除，如图 9-15 所示。

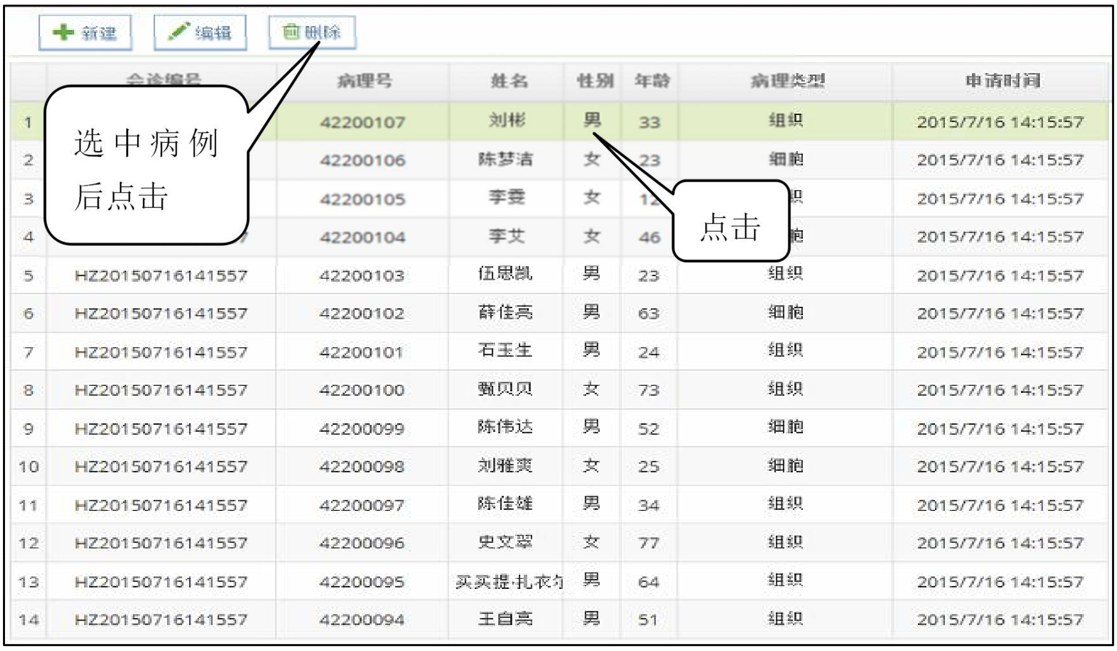

图 9-15 删除未提交会诊

⑤ 搜索

根据病理号对未提交的病例进行搜索，在【搜索输入框】输入相关病理号，按【Enter】键，只显示符合该病理号的病例，如图 9-16 所示。

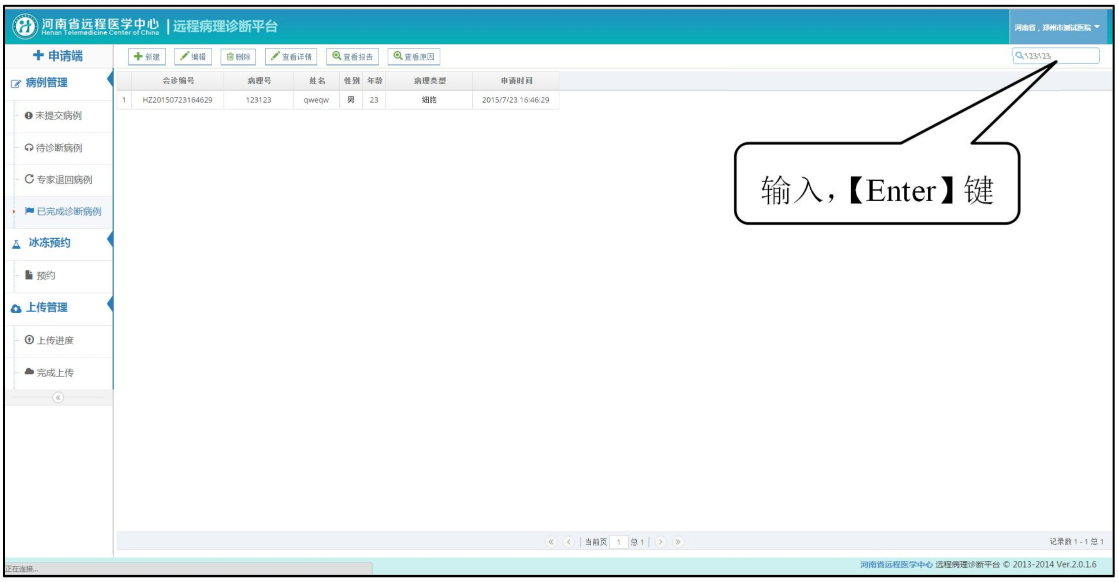

图 9-16 搜索

⑥ 翻页

若病例数超过每页最大数则会显示在第二页，用户用以下按钮进行翻页，说明如图 9-17 所示。

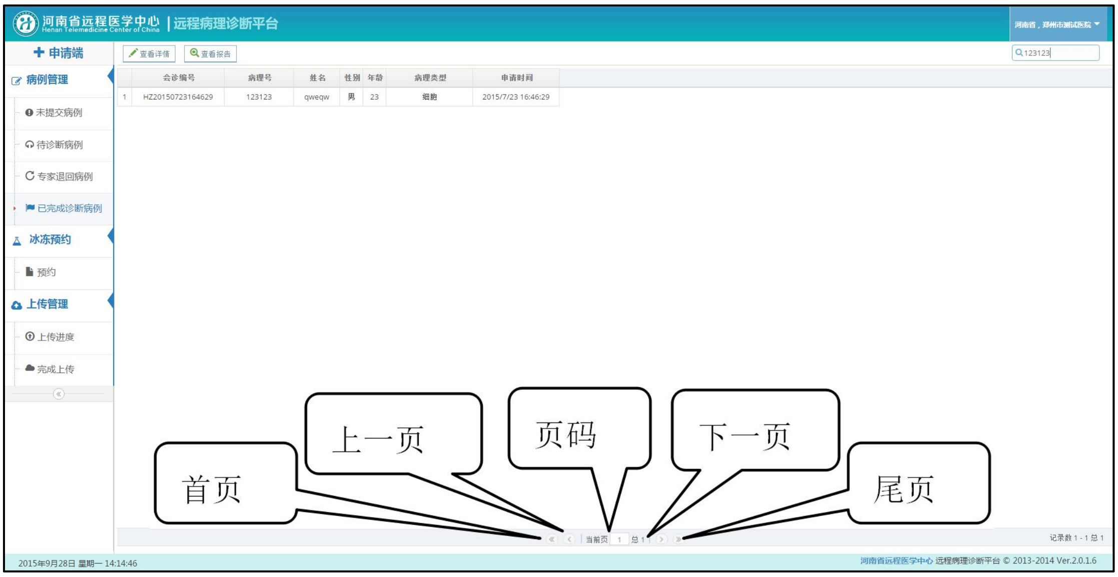

图 9-17 翻页

（4）　待诊断列表模块

①　查看已经上传会诊病例信息

病例提交申请后，可点击导航栏【待诊断病例】，选择病例后点击【查看详情】；或者直接双击病例，可查看病例上传信息。如图 9-18 所示。

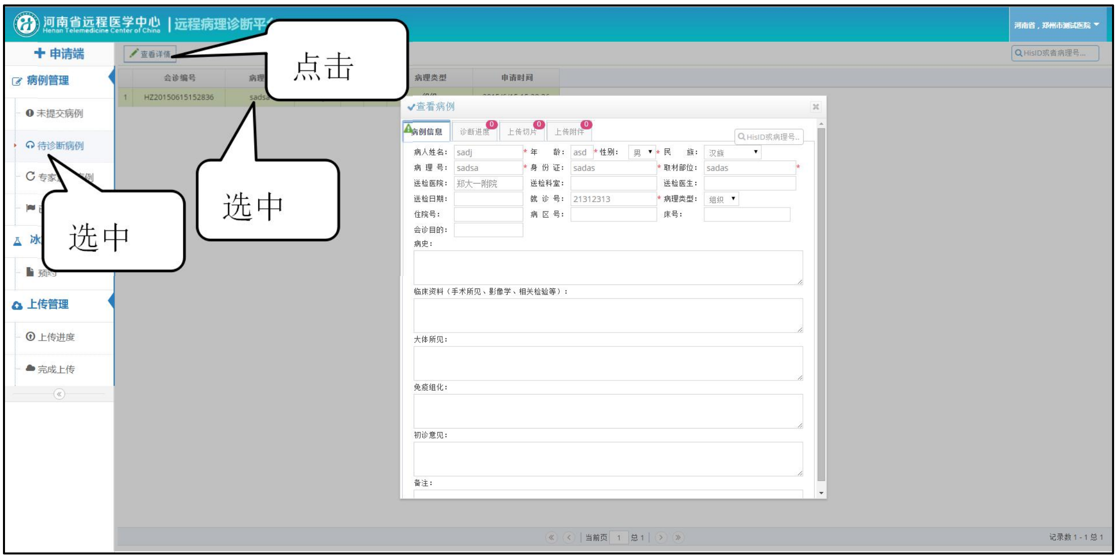

图 9-18　查看待诊断病例

②　查看诊断进度

病例上传后，可以查看起诊断进度，点击【查看详情】选择【诊断进度】，如图 9-19 所示。

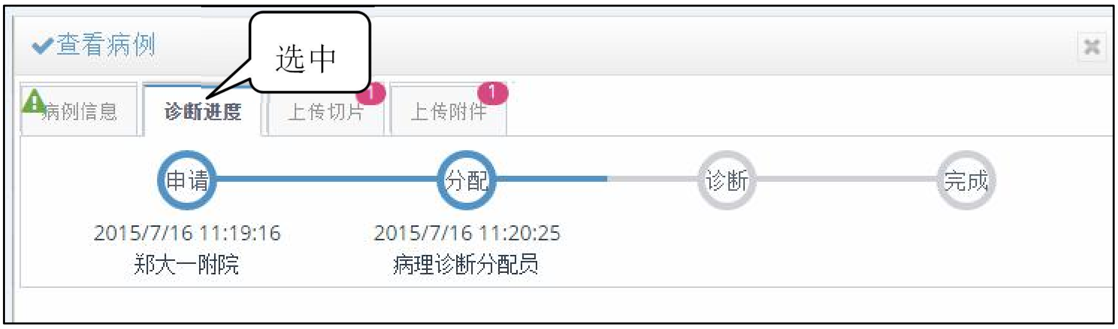

图 9-19　查看诊断进度

③ 查看已经上传切片

点击【查看详情】选择【上传切片】，病例上传后，可以查看上传切片信息，如图 9-20 所示.

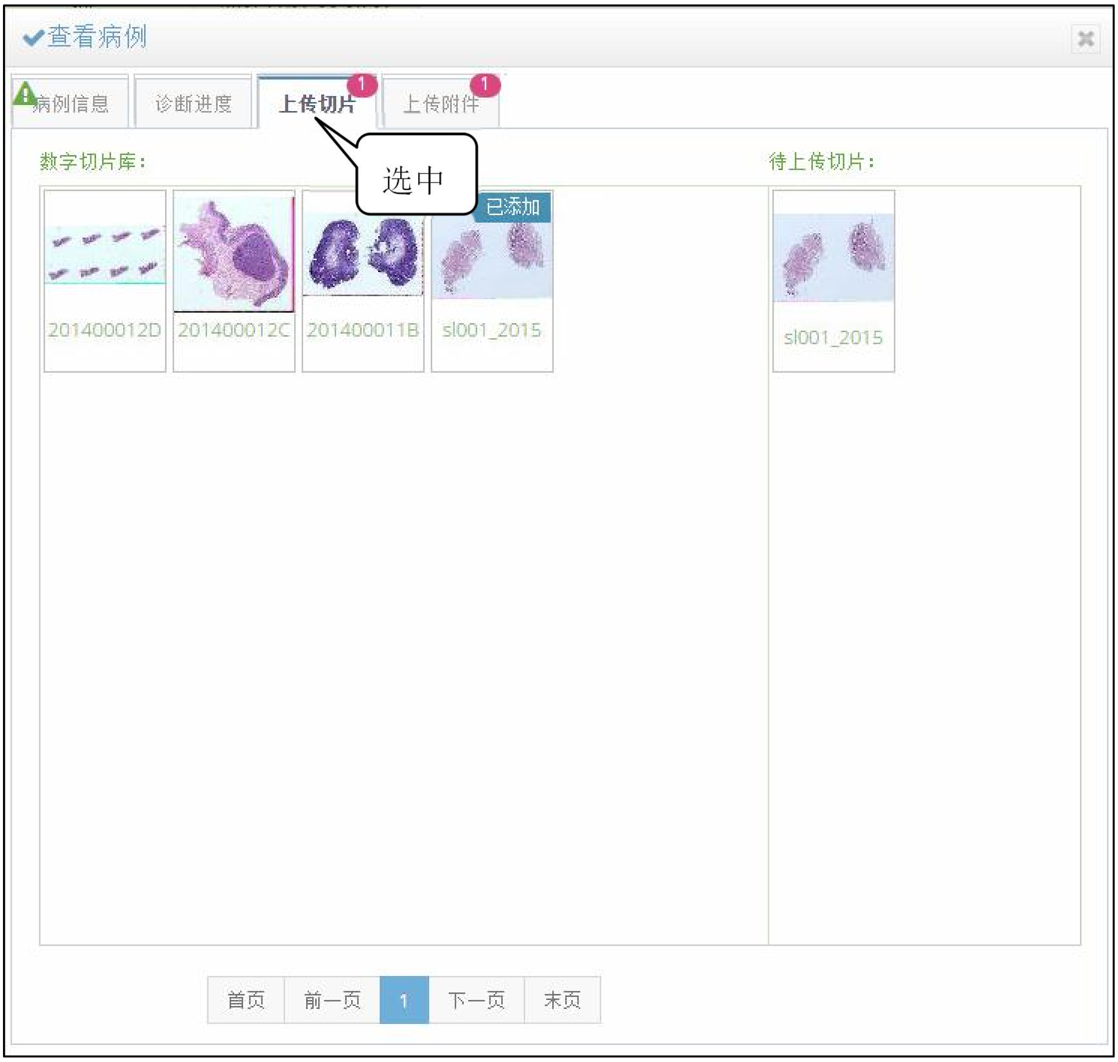

图 9-20 查看上传切片

④ 查看已经上传附件

点击【查看详情】选择【上传附件】，病例上传后，可以查看上传附件信息，如图 9-21 所示。

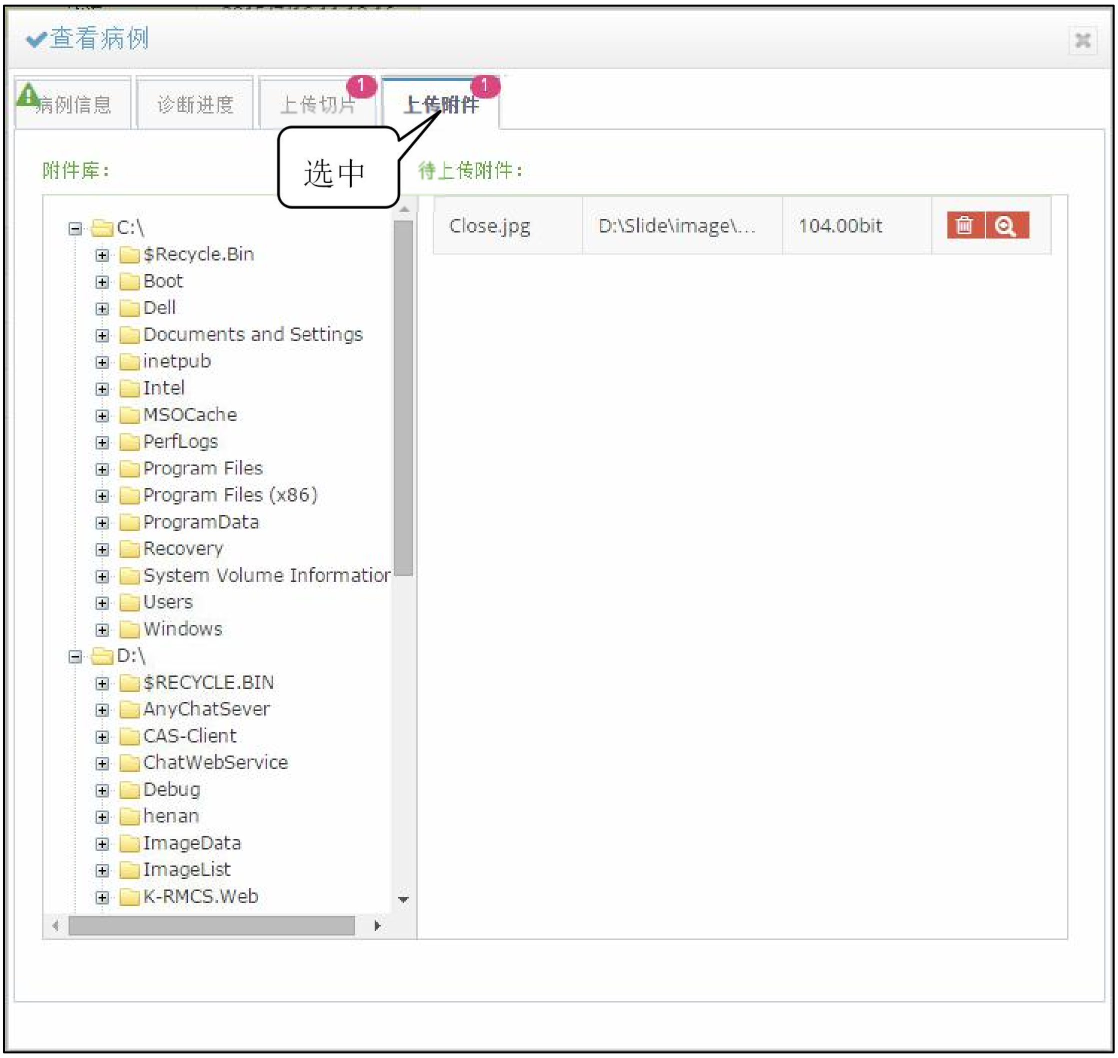

图 9-21 查看上传附件

（5） 专家退回列表模块

① 查看退回原因

选中【被退回病例】，选中病例，点击【查看原因】按钮，弹出退回理由窗口，如图 9-22 所示。

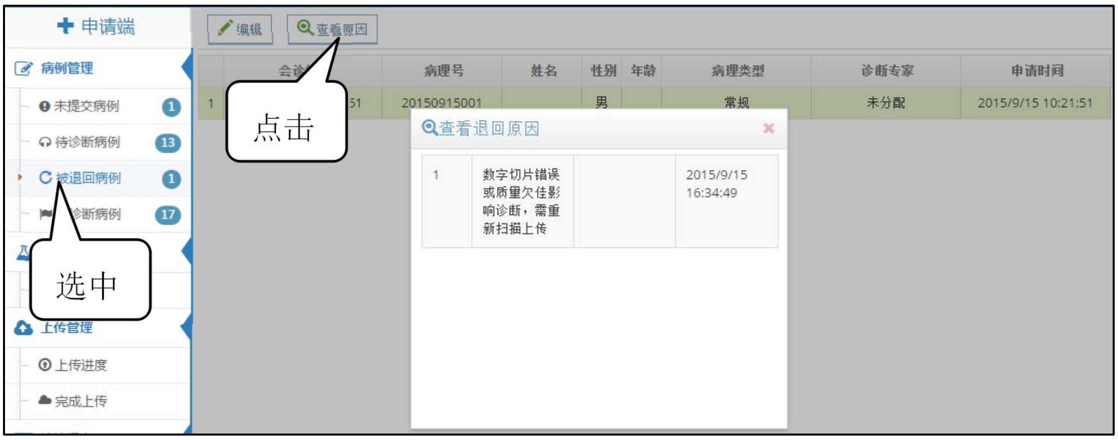

图 9-22 查看退回病例退回理由

② 重新编辑会诊病例

若要编辑此病例可选中病例后点击【编辑】按钮进行再次编辑上传，如图 9-23 所示。

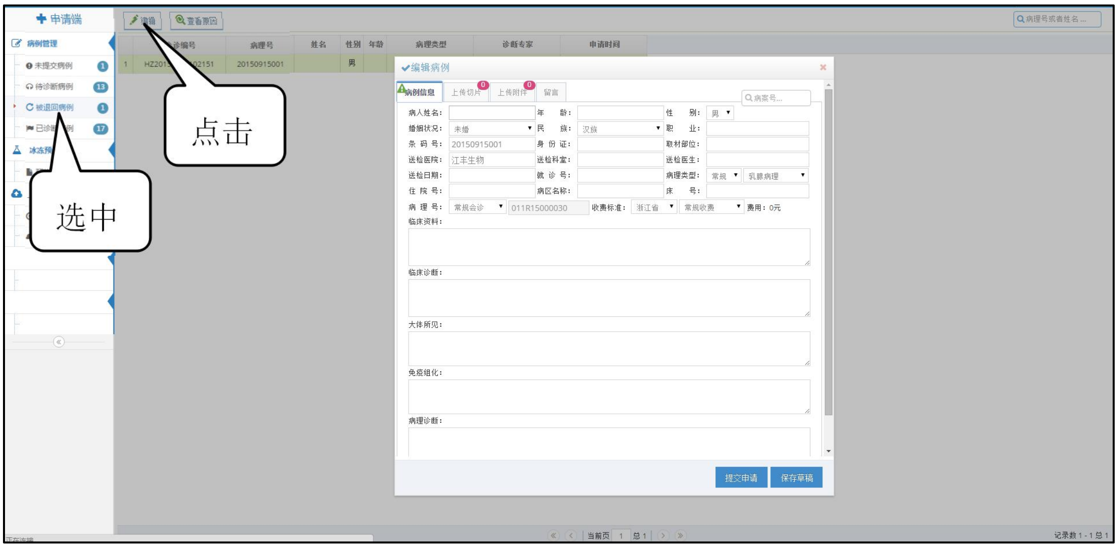

图 9-23 退回病例再次编辑

（6） 已完成诊断模块

① 查看详情

已完成病例，选中病例后点击【查看详情】，可看到病例的相关信息。

② 查看报告

选中病例后点击【查看报告】，弹出新页面可查看诊断报告，如图 9-24、图 9-25 所示。

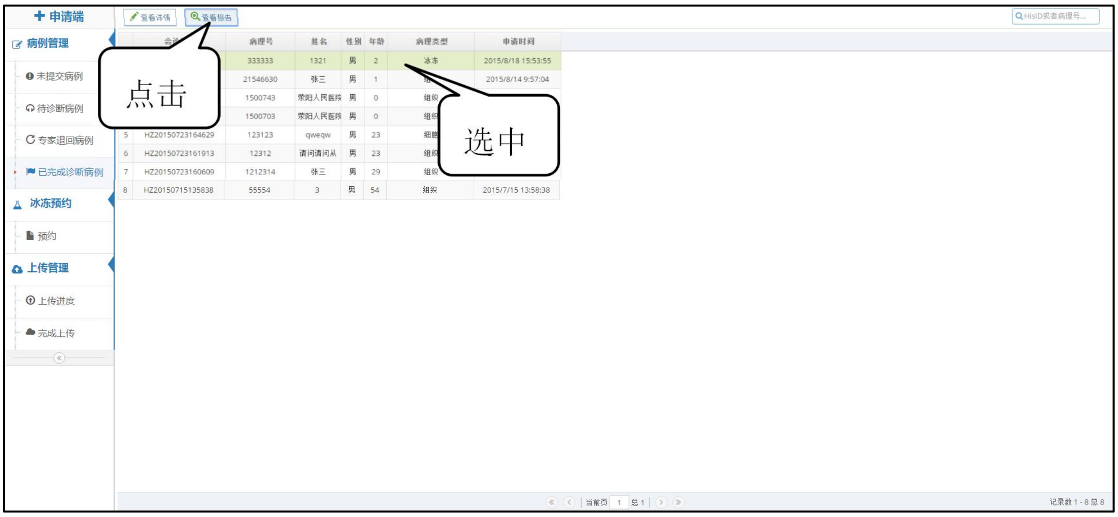

图 9-24 查看诊断报告

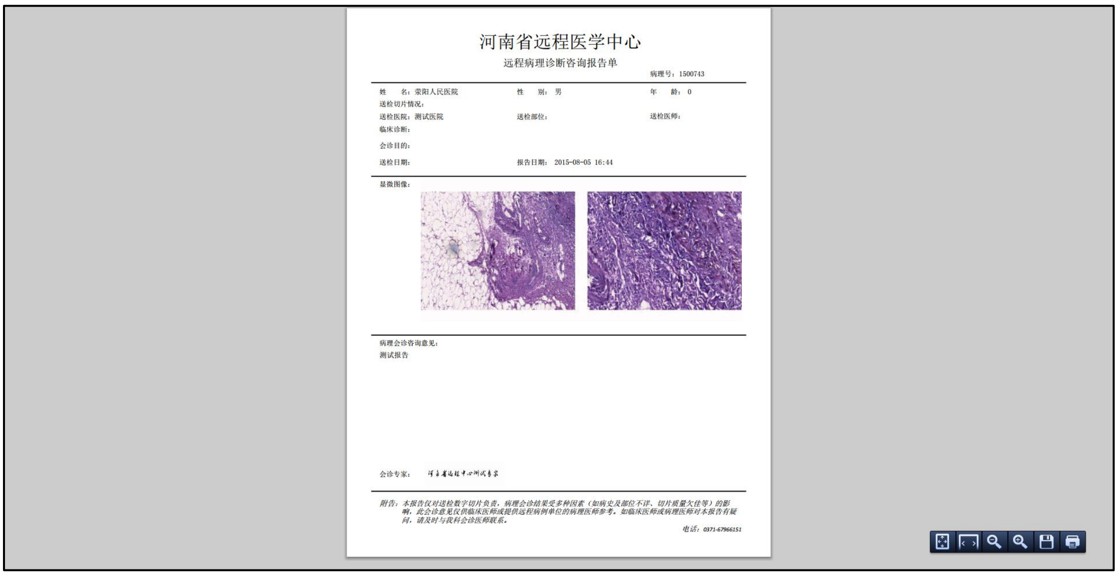

图 9-25 诊断报告页面

（7） 预约模块

① 申请预约

申请冰冻预约，填写资料提交申请。申请成功后才能编辑会诊信息，否则将一直处于申请状态，如图 9-26 所示。

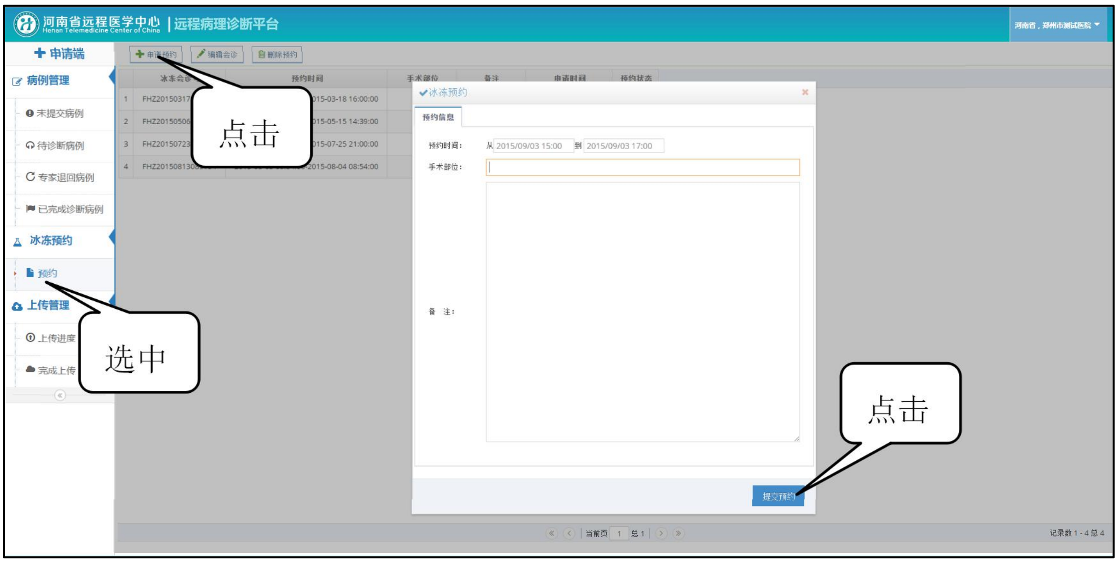

图 9-26 预约申请

② 编辑会诊

在中心通过确认，并分配专家后，预约状态会变为预约成功，此时选中双击或点击【编辑会诊】按钮，可以进行上传会诊操作，如图 9-27 所示。

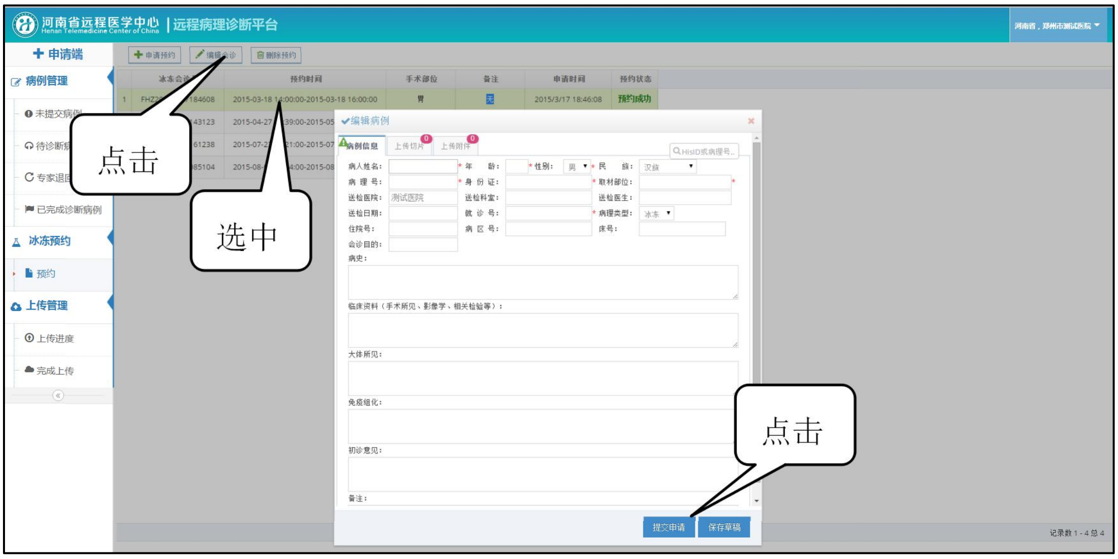

图 9-27 编辑成功预约

③ 删除预约

点击【删除预约】，将选中的病例进行删除，删除预约只能在状态为申请中，其他状态无法删除，如图 9-28 所示。

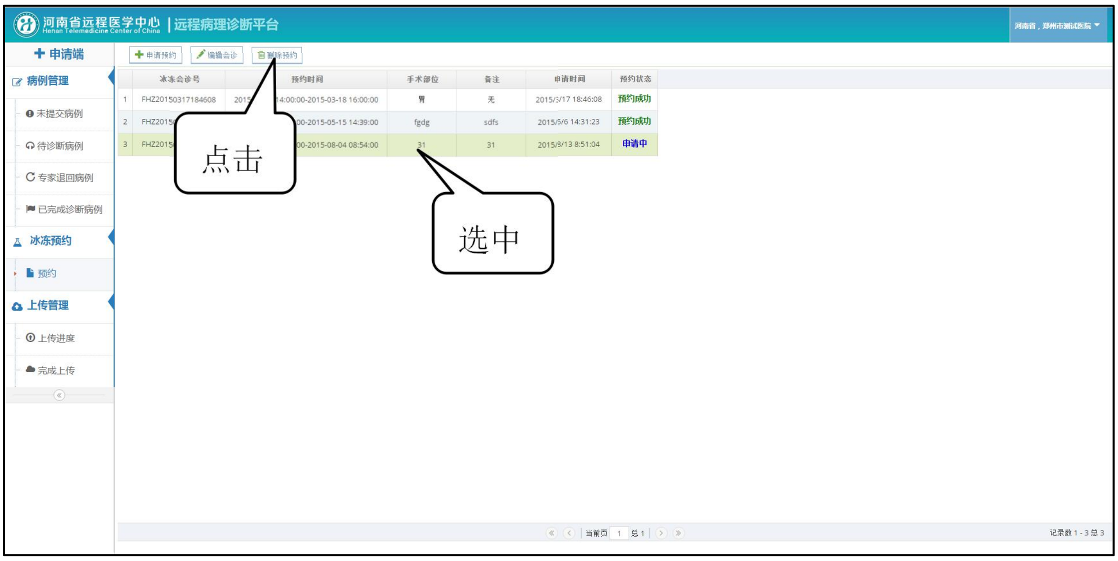

图 9-28 删除预约申请

（8）　上传模块

① 上传工具

在会诊提交后，上传服务会将选择的切片与附件上传至 Ftp 中，传输速度与进度可以在悬浮窗中查看，如图 9-29 所示。

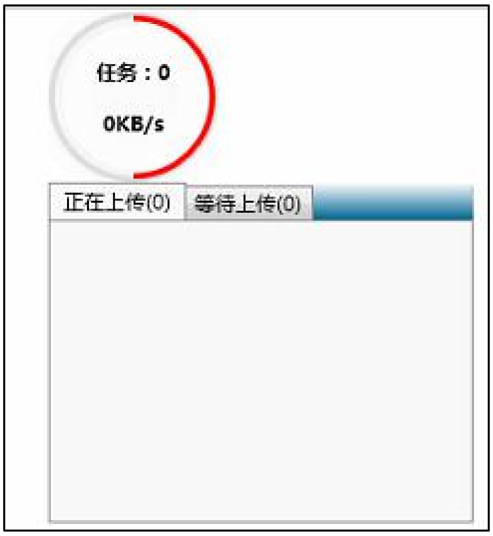

图 9-29 上传服务

② 查看上传进度

在左侧功能栏中选择【上传进度】，可以查看所有上传切片和附件的上传进度与状态，如图 9-30 所示。

序号	名称	来源	目标存储	大小	进度	速度	状态
1	sl001_2015	D:\ImageList\201400008A.kfb	12312		100.00%	0KB/s	已上传
2	sl001_2015	D:\ImageList\201400008A.kfb	2503146		100.00%	0KB/s	已上传
3	sl001_2015	D:\ImageList\201400008A.kfb	sl001		100.00%	0KB/s	已上传
4	2014002.pdf	D:\K-RMCS.Web\Report\2014002\2014002.pdf	sl001/Annex	28.70Kb	100.00%	0KB/s	已上传
5	sl001_2015	D:\ImageList\201400008A.kfb	sl002		100.00%	0KB/s	已上传

图 9-30 上传进度

③ 查看完成上传信息

在左侧功能栏中选择完成上传，可以查看所有已经上传的切片和附件的信息，如图 9-31 所示。

序号	名称	来源	目标存储	大小	完成时间
1	sl001_2015	D:\ImageList\201400008A.kfb	sl002		2015/7/16 11:11:48
2	2014002.pdf	D:\K-RMCS.Web\Report\2014002\2014002.pdf	sl001/Annex	28.70Kb	2015/7/16 9:41:02
3	sl001_2015	D:\ImageList\201400008A.kfb	sl001		2015/7/16 9:41:02
4	sl001_2015	D:\ImageList\201400008A.kfb	2503146		2015/7/15 19:50:24
5	sl001_2015	D:\ImageList\201400008A.kfb	12312		2015/7/15 18:51:56

首页 前一页 1 下一页 末页

图 9-31 上传完成信息界面

1.3.3　专家端管理

专家可利用数字病理远程会诊平台，通过浏览器，进行数字病理远程诊断或会诊，不受时间与空间限制。同时支持 Pad 平板电脑浏览切片、截图以及生成报告功能，如图 9-32 所示。

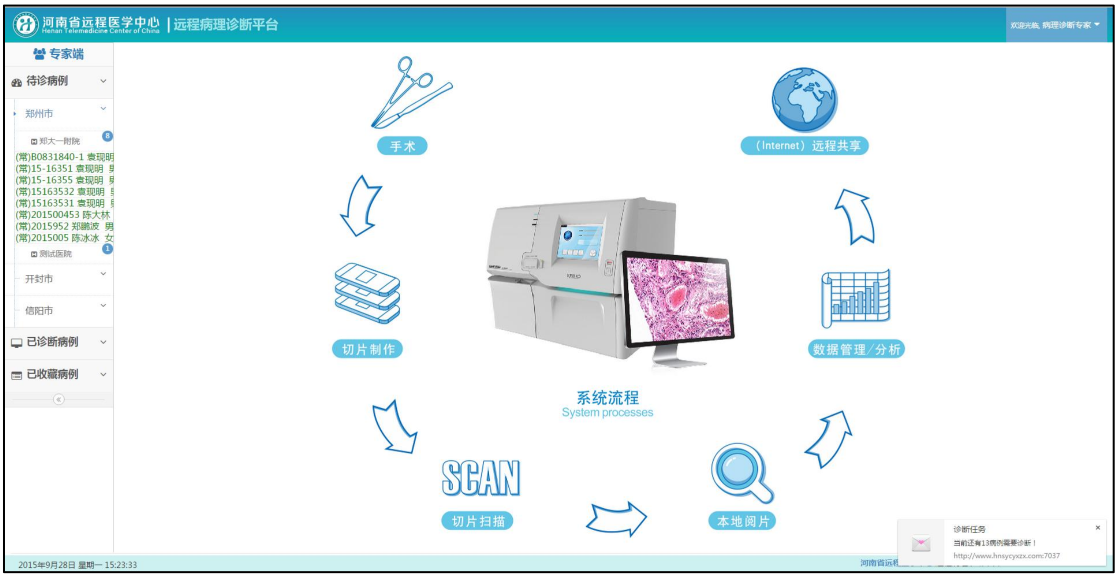

图 9-32　专家诊断界面

（1）　病例列表模块

①　待诊病例列表

根据市级行政区域将医院进行分类，在医院内显示该医院所申请的病例，如图 9-33 所示：

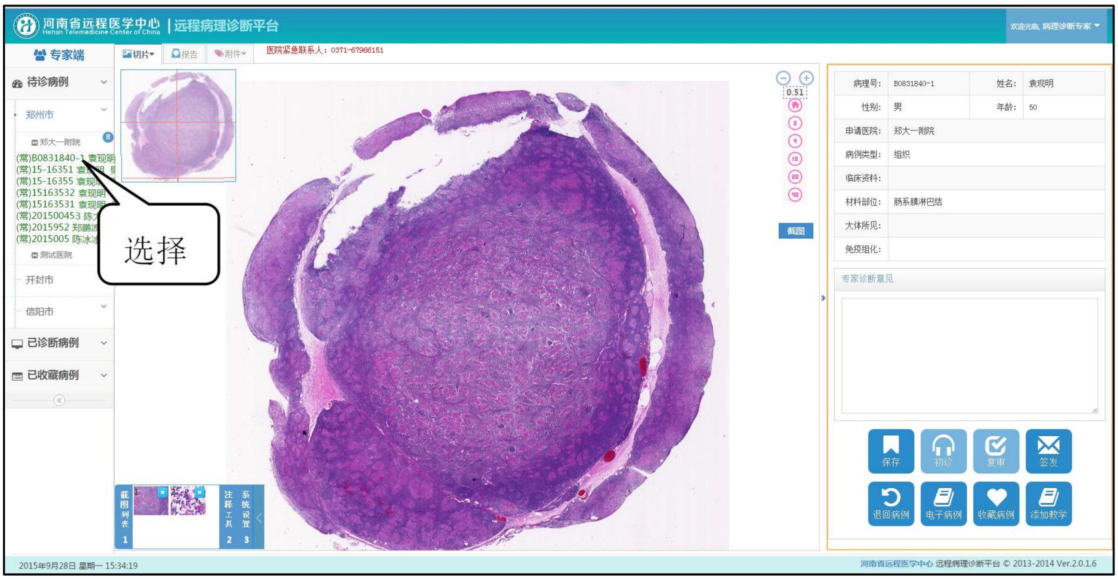

图 9-33　诊断待诊病例

②　已诊断病例列表

根据省份与市区将医院进行分类，在医院内显示该医院所申请的并且已经诊断完成的病例，如图 9-34 所示。

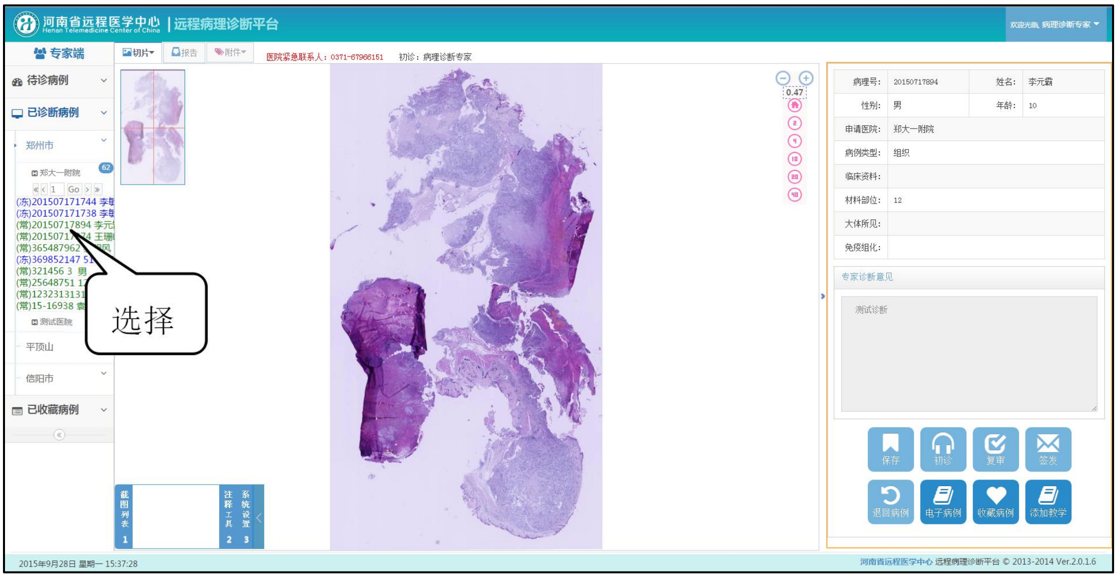

图 9-34 已诊断病例

③ 已收藏病例列表

在病例中点击【收藏】按钮，则在收藏病例里可以根据医院显示专家所收藏的病例，如图 9-35 所示。

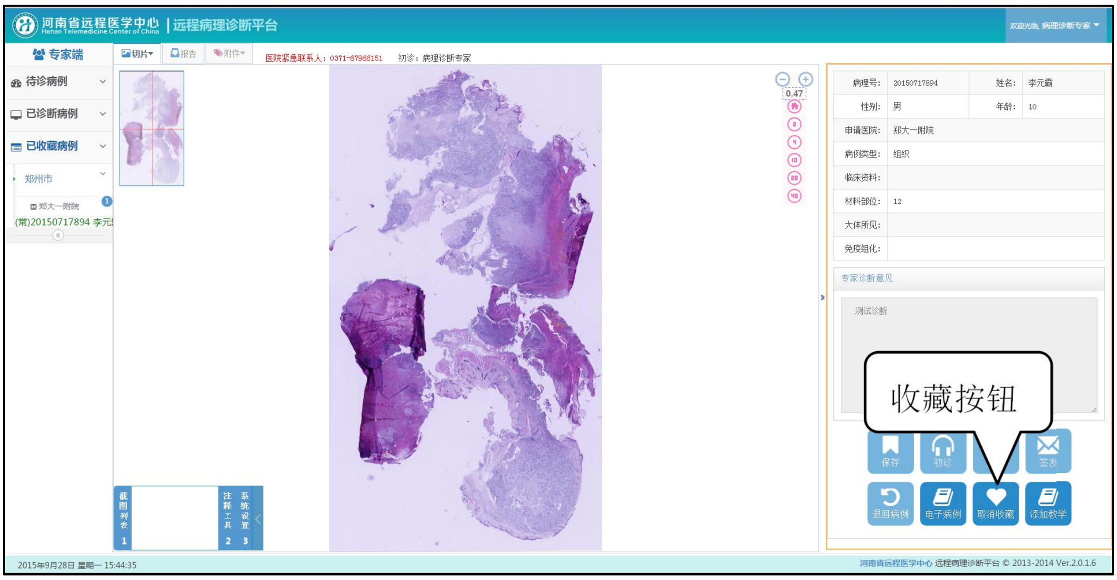

图 9-35 已收藏病例

（2） 专家阅片模块

专家登录首页后，点击右侧的会诊，打开阅片界面，进行浏览切片

① 基本操作功能

放大、缩小操作，鼠标滚轮上下滚动；鼠标按住切片图像可以拖动；导航图在左上角可以定位切片大致位置；倍率工具可以显示倍率信息，点击倍率数可以快速指定图像倍率，可以放大缩小，如图 9-36 所示。

② 截图功能

截图、删除截图、截图框打开/隐藏，如图 9-36 所示。

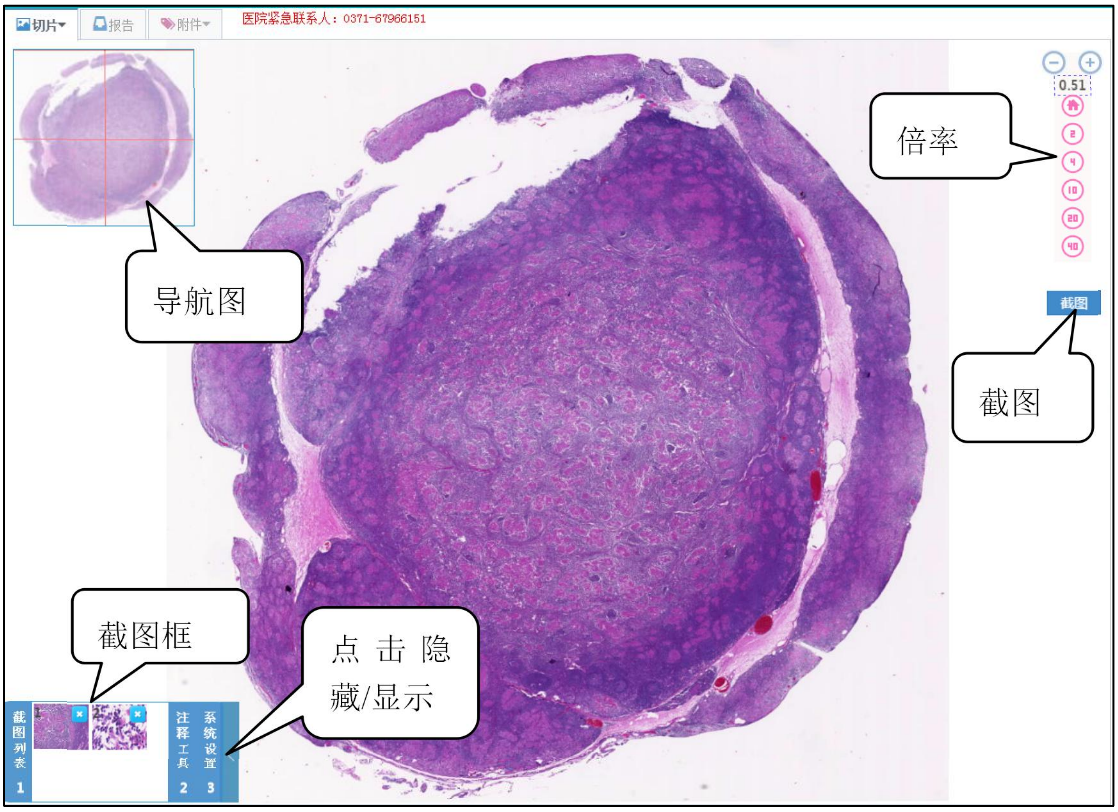

图 9-36 阅片界面

③ 注释功能

在阅片区域【右键】【注释】，在阅片区直接画注释，会弹出注释编辑界面，可以改变注释的颜色大小等，如图 9-37、图 9-38 所示。

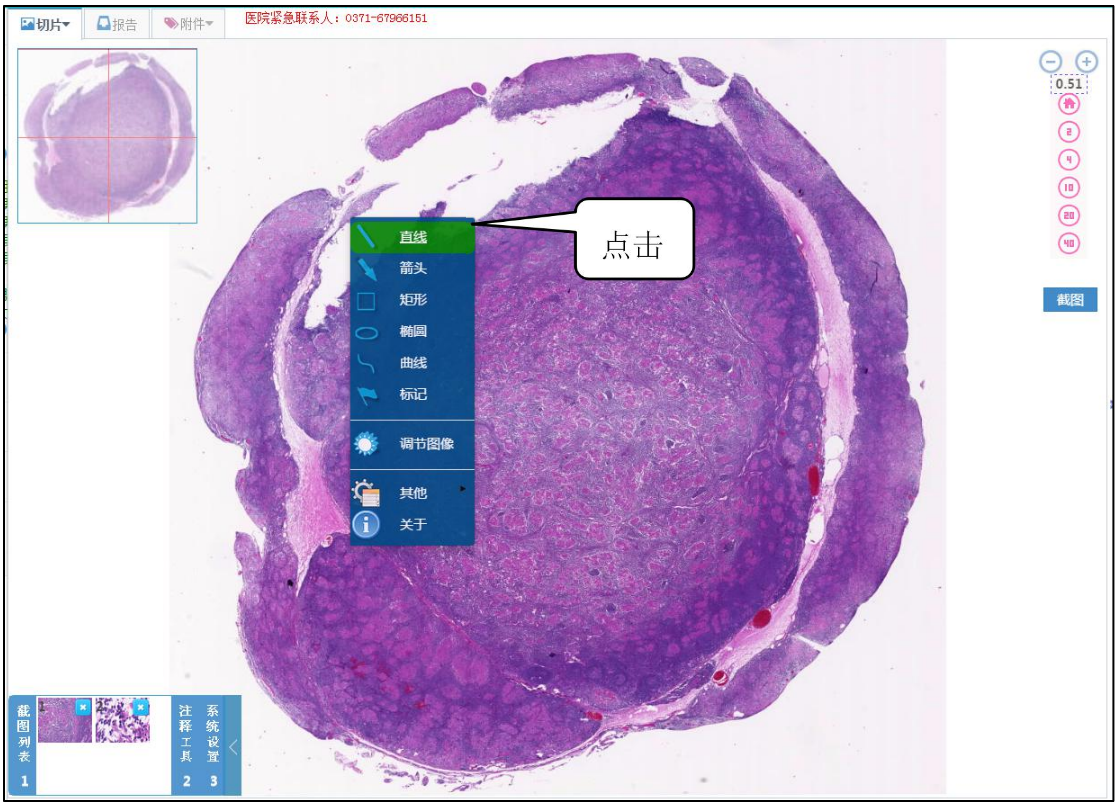

图 9-37 注释界面

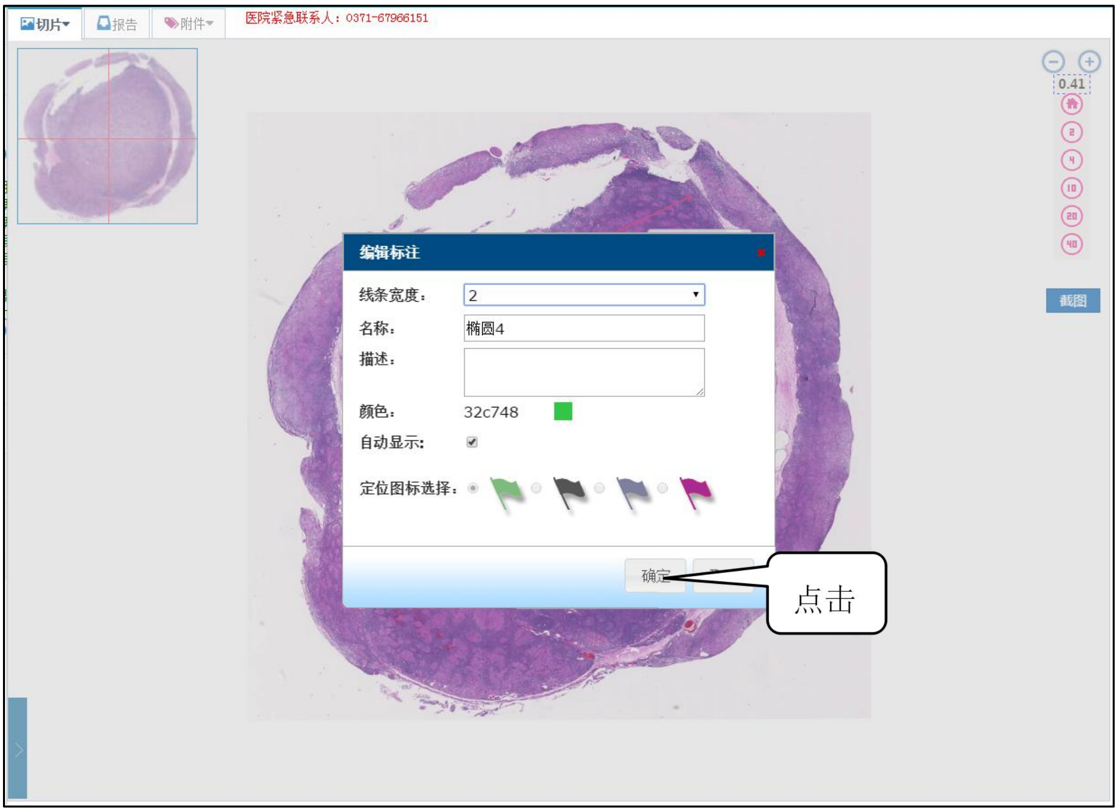

图 9-38 注释界面

在阅片区域选择注释后【右键】【编辑】，可看到所有注释的编辑操作，可以改变名称、大小、描述、颜色、显示与否等，如图 9-39 所示。

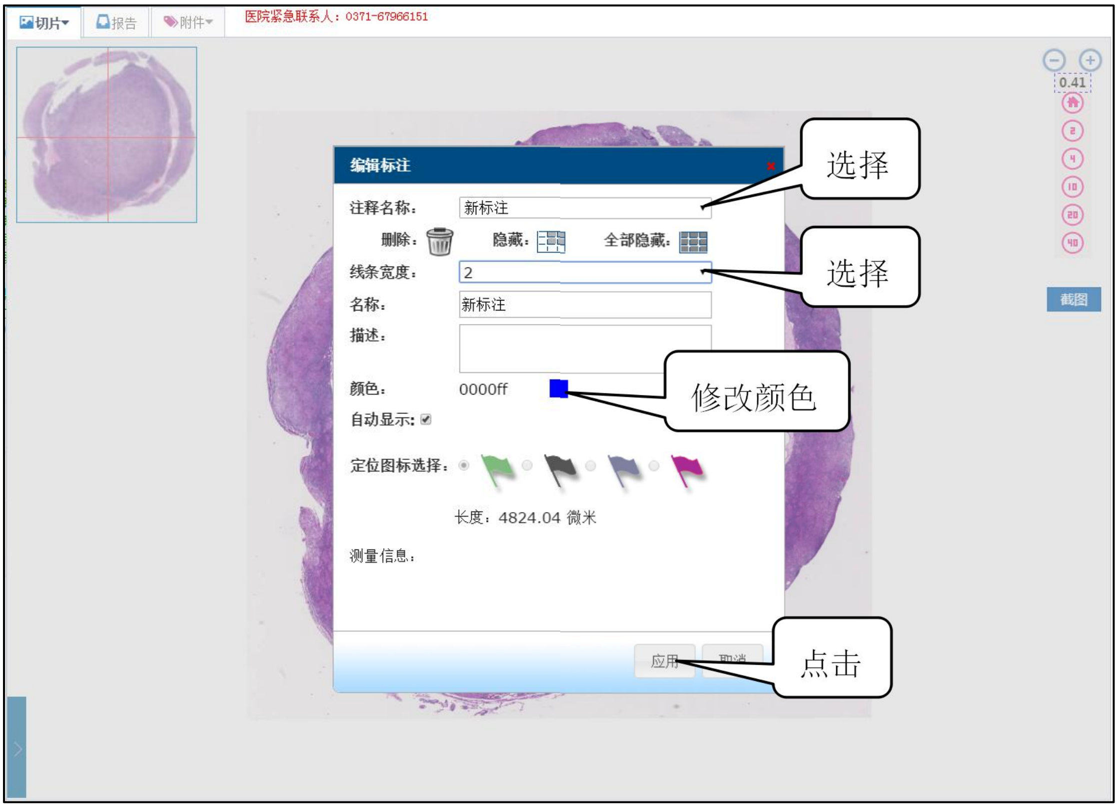

图 9-39 注释编辑

在阅片区域选择后【右键】【调节图像】，可看到对图像的具体操作，伽马因子、对比度、亮度、红绿蓝颜色通道应用修改，如图 9-40 所示。

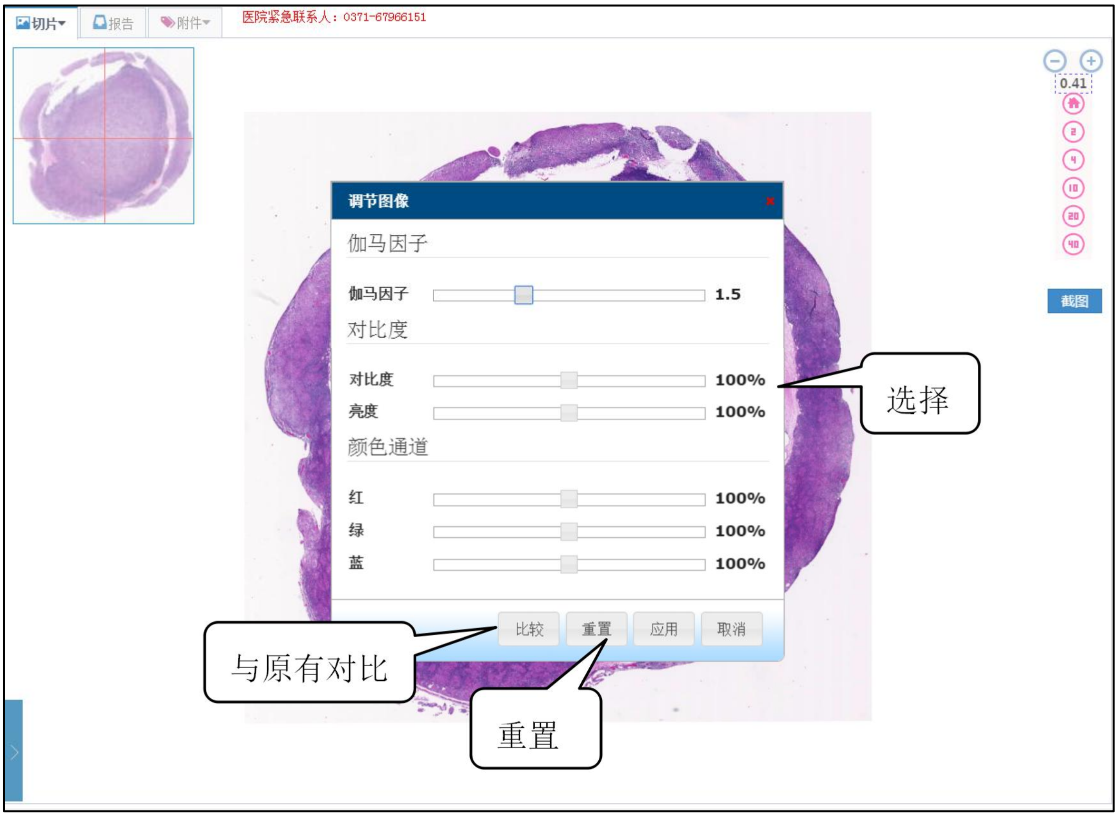

图 9-40 调节图像

（3） 病例信息模块

用来显示医院申请时所输入的各种信息，如姓名、性别、年龄、病史等信息，如图 9-41 所示。

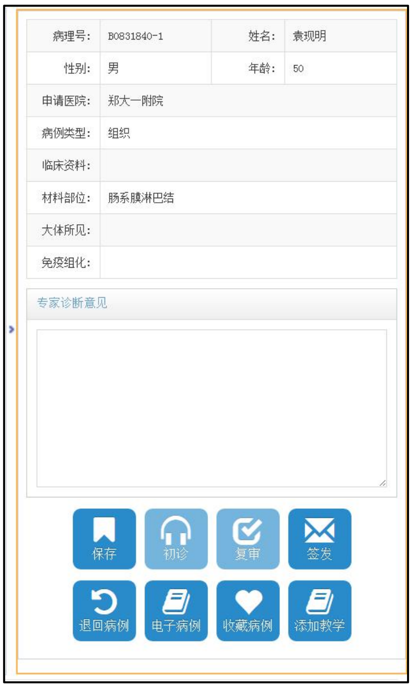

图 9-41 病例信息

（4） 退回模块

将专家认为不能够进行病理诊断的会诊退回医院，使其改正后重新申请。可以选择退回理由填写退回理由，也可以查看历史退回原因，如图 9-42 所示。

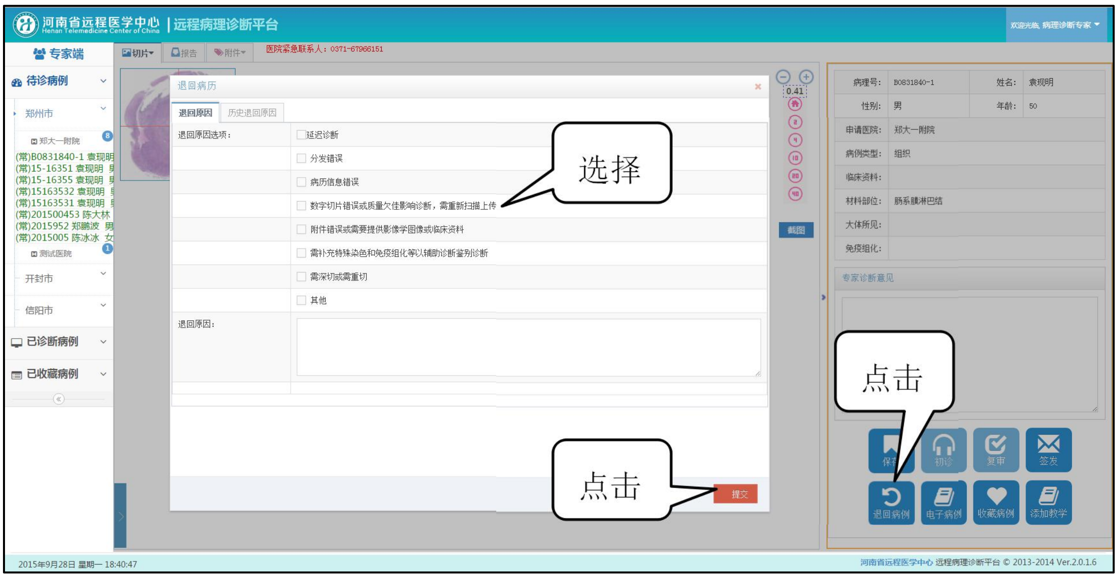

图 9-42 退回病例

（5） 生成报告模块

在阅片过程中对需要进行记录的地方进行截图，在专家诊断意见中输入诊断意见，点击【保存】按钮，点击【签发】生成病理报告，若检查无误后点击【提交报告】按钮即诊断成功，如图 9-43 所示。

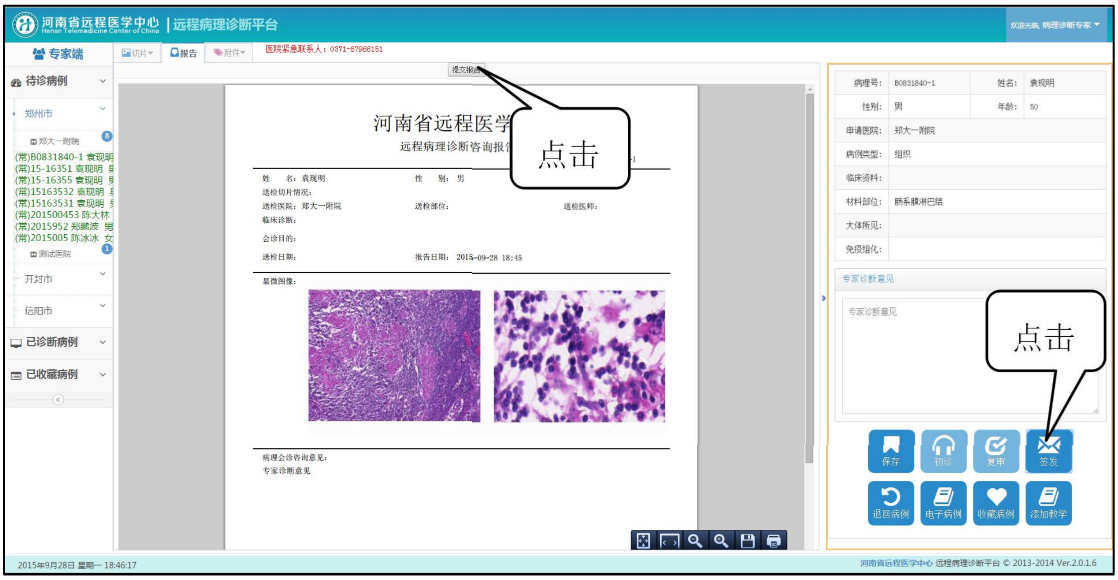

图 9-43 签发报告

（6） 查看附件模块

点击附件下拉菜单，选中后打开附件，如图 9-44 所示。

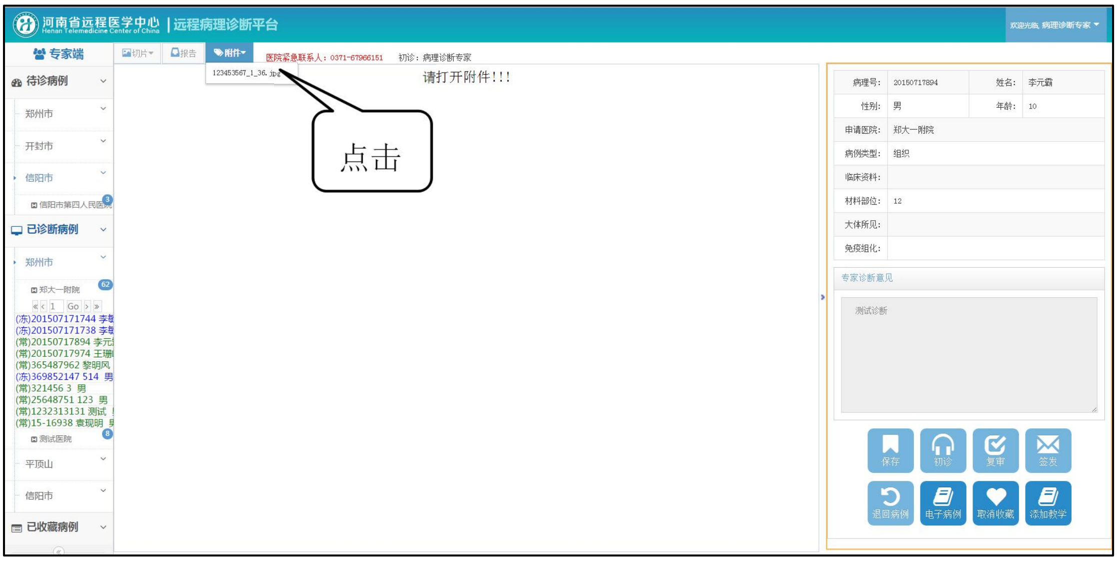

图 9-44 附件

（7） 电子病历浏览器模块

点击【电子病历】按钮，弹出电子病历浏览器，如图 9-45 所示。

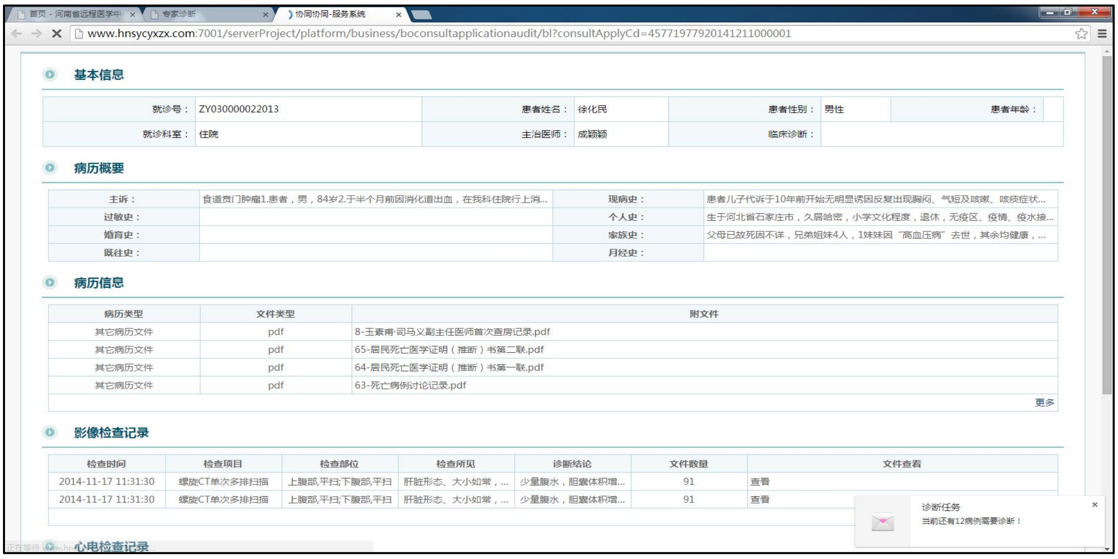

图 9-45 电子病历

（8） 音视频模块

点击【添加教学】按钮，将病例添加至音视频教学系统中。若添加成功后打开音视频软件则可看到此病例已存在病例中，如图 9-46 所示。

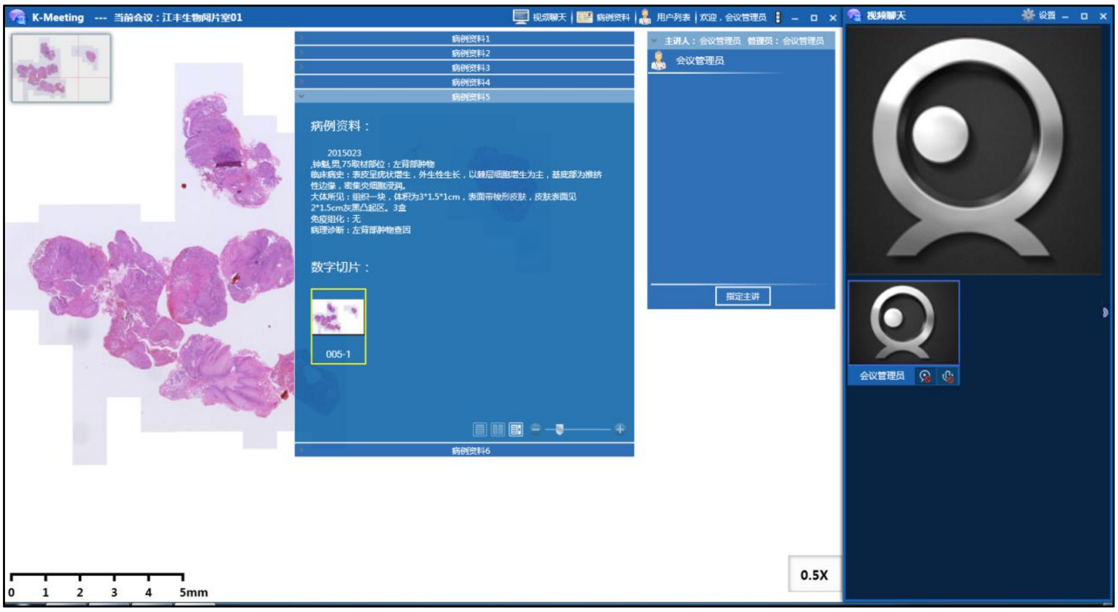

图 9-46 添加教学

9.4 常见问题及说明

病理诊断系统数据来自河南省远程医疗云平台，用户帐号（包括申请医院账号、专家账号）由河南省远程医学中心提供。

病例信息提交后需要分配病例，此功能也由河南省远程医疗云平台提供，主要是完成常规病例提交后根据专家列表分配、进行冰冻预约确认及联系专家。

使用本平台签发的报告可根据医院不同上传。

在阅片过程中切片可以本地（医院内网）不上传阅片，也可上传到云端供外网阅片，其中上传服务工具要另外安装。所以若专家阅片时发现打开数字切片提示服务器未开是因为上传切片的机器电脑没有打开，打开机器及电脑就可以正常阅片。

9.5 数字切片扫描仪操作说明

数字病理远程会诊的诊断对象是数字病理切片，数字切片扫描仪的主要功能是把实物病理切片转为数字切片。会诊申请医院或医务人员在申请数字病理远程会诊之前，需要通过数字切片扫描仪将实物病理切片扫描为数字病理切片，连同患者病历资料一起提交至远程病理会诊专家，由专家给出诊断意见。所有需要提出数字病理远程会诊请求的医务人员，都要学会如何操作数字切片扫描仪。操作人员可通过扫描软件操作数字切片扫描仪，下面详细介绍具体操作方法。

9.5.1　切片缩略图列表区

软件只有一个界面，主界面可以分为 9 个区域，首先介绍切片缩略图列表区。切片缩略图列表区域显示的是移动平台上五个切片槽的切片缩略图，缩略图中包含的信息有：切片标签、预览图、切片状态，如图 9-47 所示。

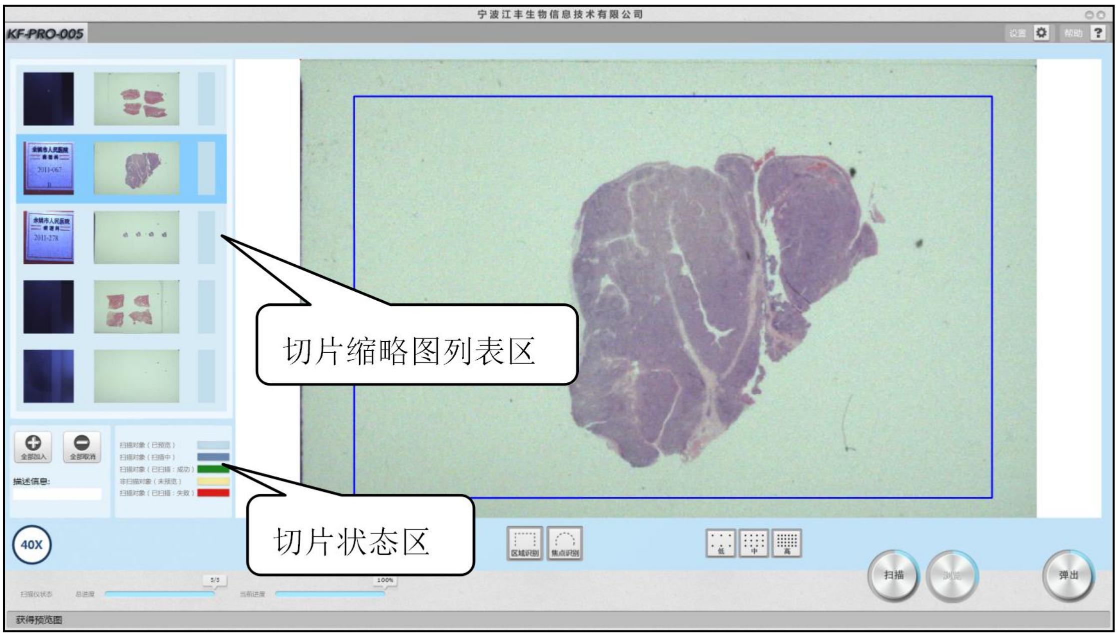

图 9-47 切片缩略图列表区

切片状态有以下五种：已预览、扫描中、扫描成功、未预览、扫描失败。不同的状态对应不同的颜色表示，表示方法如图 9-48 所示。

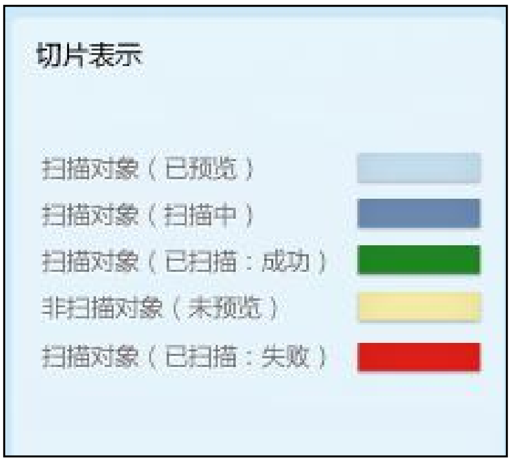

图 9-48 切片状态区

9.5.2　基础操作功能区

基础操作功能区包括【全部加入】、【全部取消】、【描述信息】按钮。

【全部加入】是将所有切片加入成为扫描对象，即所有切片呈现被红框选中的状态。

【全部取消】是取消当前所有状态为扫描对象的切片。

【描述信息】当进行多片或单片扫描时，用户可以对要扫描的切片进行相关的描述。扫描信息的描述是在加入扫描对象之前进行的，当前的扫描信息只能赋给当前加入扫描对象的切片，一张或者多张。如图 9-49 所示。

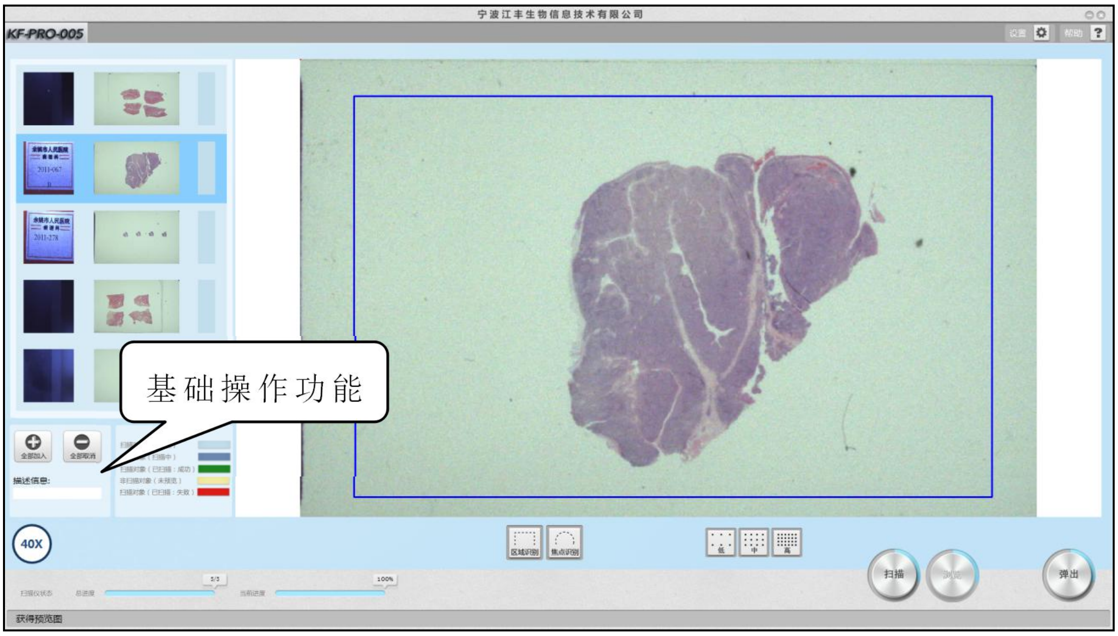

图 9-49 基础操作功能区

注：扫描信息的描述中不能出现一些特殊的字符，比如：\ / : * ? " < > |。且扫描描述信息的长度不能超过 50 个字符（即 25 个汉字）。

9.5.3　倍率切换

【倍率切换】用户在开始扫描或预览等操作之前，可以通过机器左侧板的 20 倍与 40 倍档位来切换倍率。每次切换倍率后，机器将会做初始化操作。

注意：在扫描过程、预览过程或者其他操作过程中，切换倍率后，软件会自动退出。所以用户在扫描工作过程中，切勿进行倍率切换的操作。如图 9-50 所示。

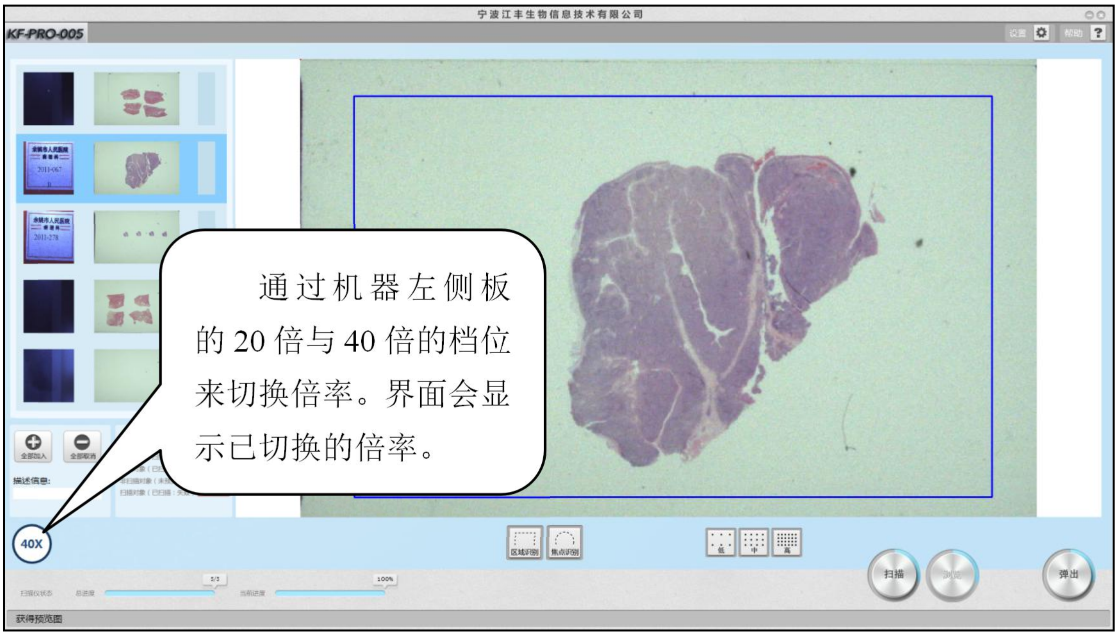

图 9-50 倍率切换

9.5.4　扫描状态显示区

扫描仪状态表示的是总进度和当前进度。总进度表示的当前已操作结束的扫描对象与总共的扫描对象数量的比值；当前进度表示的是当前正在扫描的切片的扫描进度百分比。如下图所示：对一个扫描对象进行对焦操作，且对焦进行中，总进度为 0/1；当前进度表示对焦进行了 45%。

左下角的扫描状态显示为对焦中…。

常见的一些扫描状态有：连接成功、扫描中、区域识别完成、焦点识别完成、对焦中、对焦完成、补光中、线扫描中、扫描结束、弹出、预览中、预览结束等。

当扫描仪出现异常状态时，常见的提示有：主控板连接失败、LED 灯未开等。

以上一些提示状态都会显示在左下角处的扫描状态显示区，如图 9-51 所示。

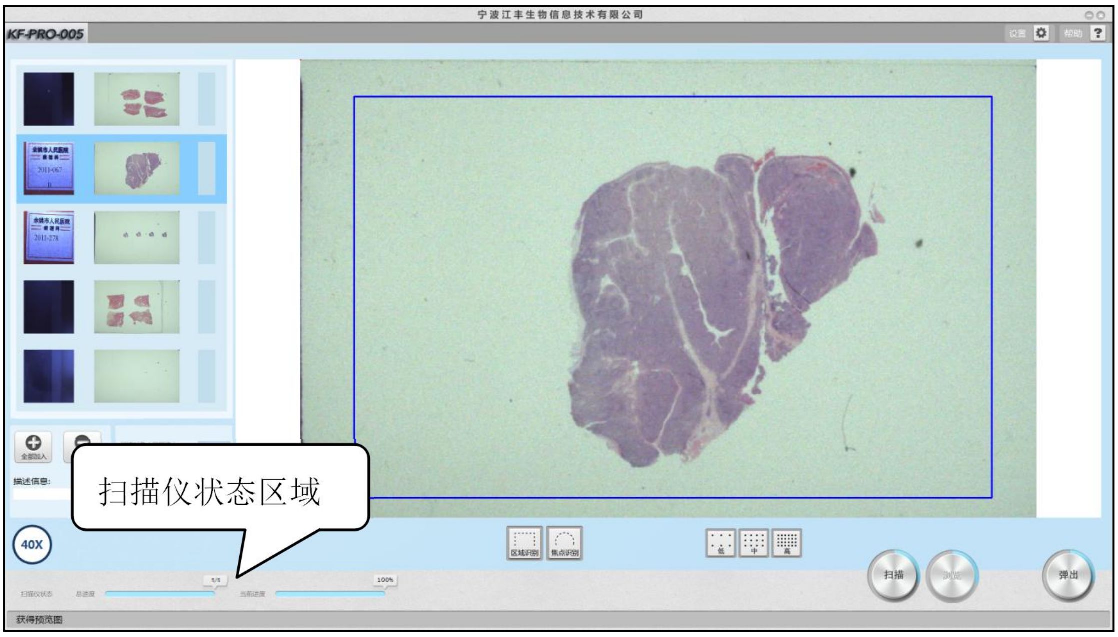

图 9-51 扫描状态

9.5.5 预览图区

预览图区显示的是用户在缩略图列表区点击过的切片的预览图。图 9-52 表示的是缩略图列表中第 5 张切片的缩略图。若点击的位置无切片缩略图存在，则预览图区显示背景。

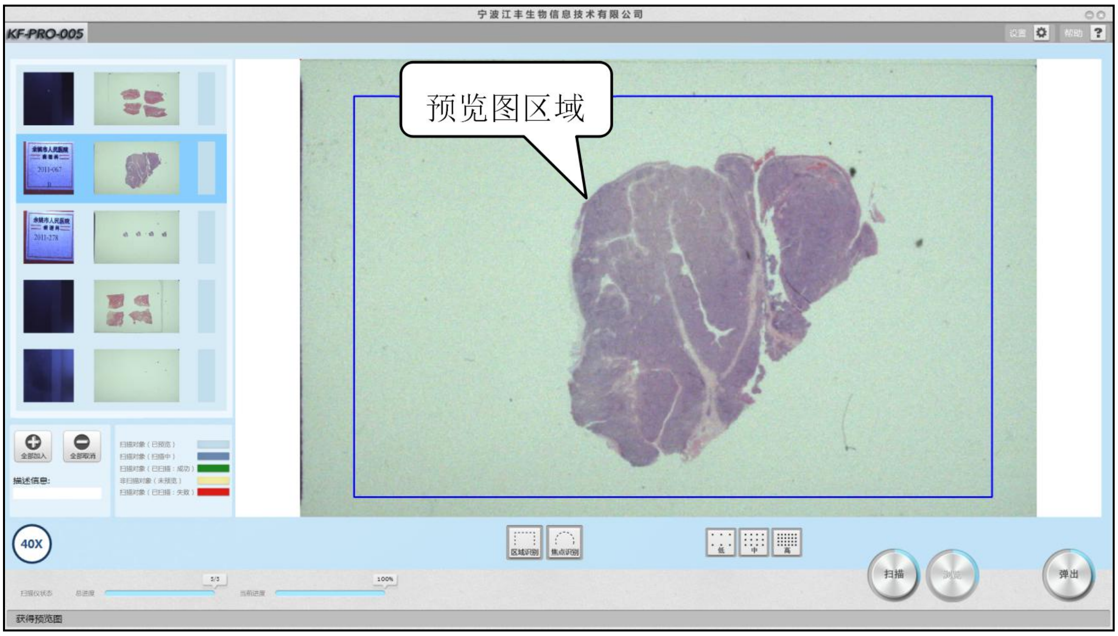

图 9-52 预览图区

9.5.6 分步扫描按钮区

分步扫描功能按钮区包括以下几个按钮：【区域识别】、【焦点识别】，如图 9-53 所示。

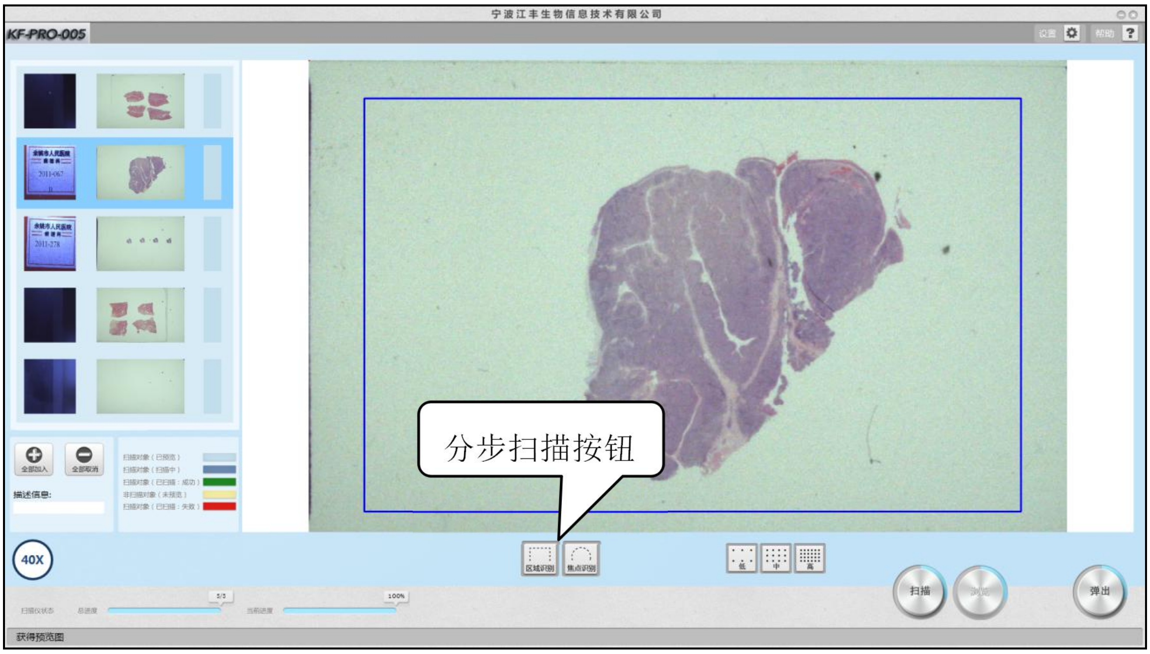

图 9-53 分步扫描按钮

【区域识别】实现的功能就是将切片的组织部分自动识别并通过红框框选出来。然后用户可以通过红框改变区域的大小。

【焦点识别】系统自动识别出焦点后，并在预览图上显示这些焦点的位置。焦点的个数和分布位置由系统自动识别得出。用户可以通过鼠标右键的菜单对焦点进行基本的删除、增加操作，还可以对焦点进行拖动操作。

9.5.7 密度选项区

系统对焦点的分布状态设定了三个程度：【低】、【中】、【高】密度。用户在焦点识别结束后，可以点击上述的三个按钮，来确定焦点的密度。密度越高，对焦的时间会越长，但是扫描得到的图像质量会较清晰，用户可以根据自身的要求来设定。选择了一种密度后，在后面的扫描对象中将会默认选择此次选中的焦点密度，如图 9-54 所示。

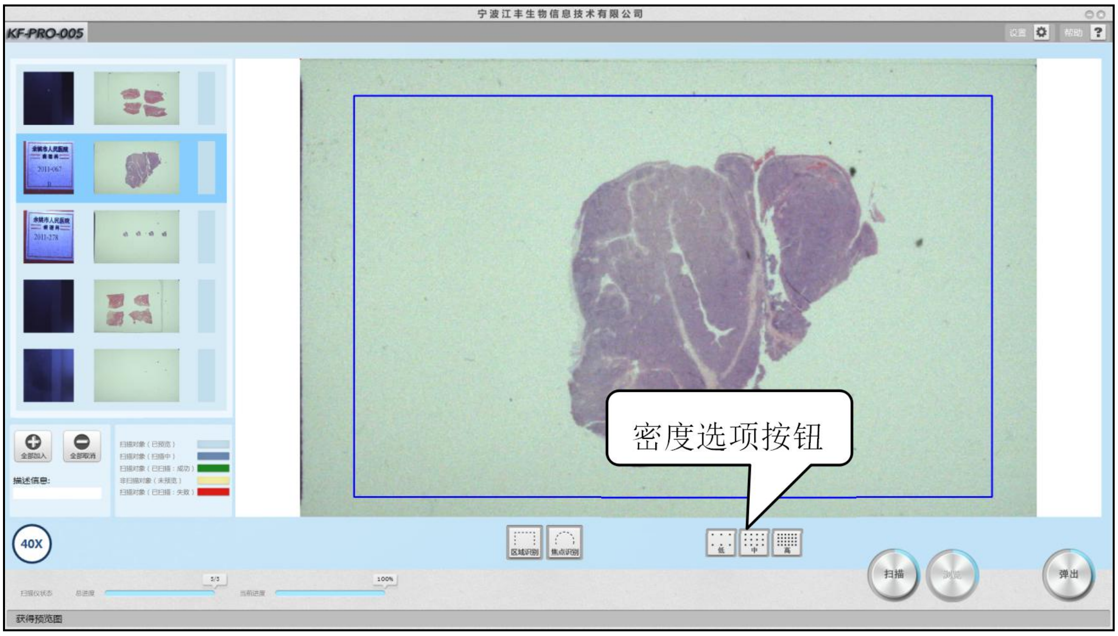

图 9-54 密度选项

9.5.8 弹出和扫描、浏览按钮

主界面右下角的三个按钮为【扫描】按钮、【浏览】和【弹出】按钮，如图 9-55 所示。关于这三个按钮的具体操作将会在下面的章节中讲到。

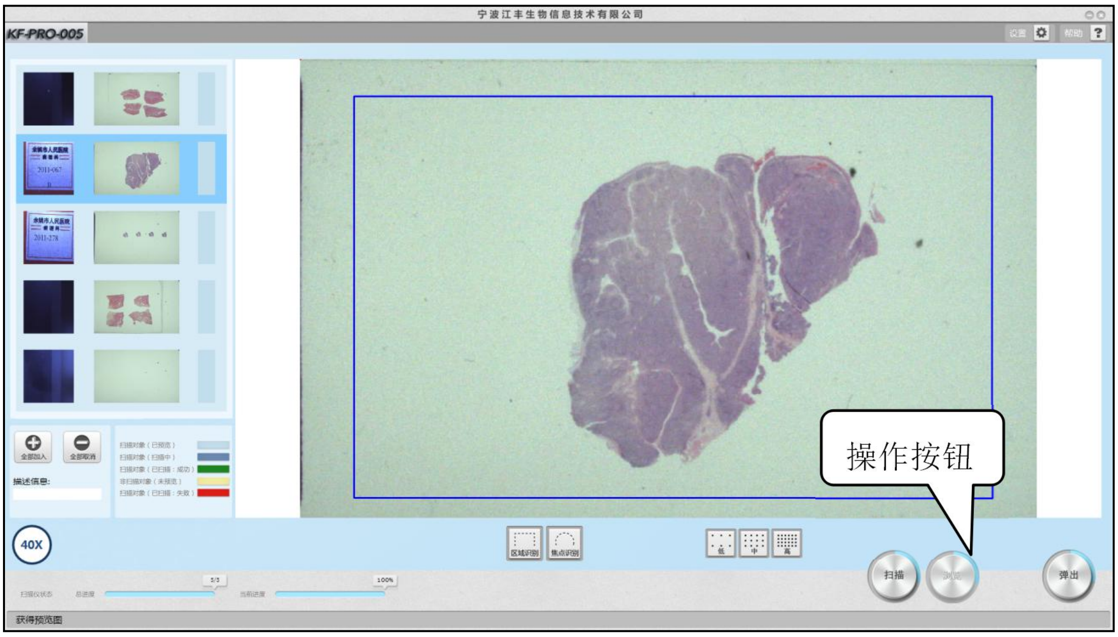

图 9-55 操作按钮

【扫描】按钮实现两种扫描方式：一键扫描和分步扫描。

【浏览】完成对当前预览区内扫描结束的图像的浏览查看操作。只有预览区内打开扫描结束的切片时按钮是处于可用状态，其他情况下都是禁用的。

【弹出】是弹出移动平台的操作，在扫描过程、预览过程或者其他操作过程中，该按钮是处于禁用状态的。

9.5.9 界面其他按钮

界面窗口控制区域在界面的右上角处：【设置】、【关闭】、【最小化】按钮、【帮助】按钮，如图 9-56 所示。

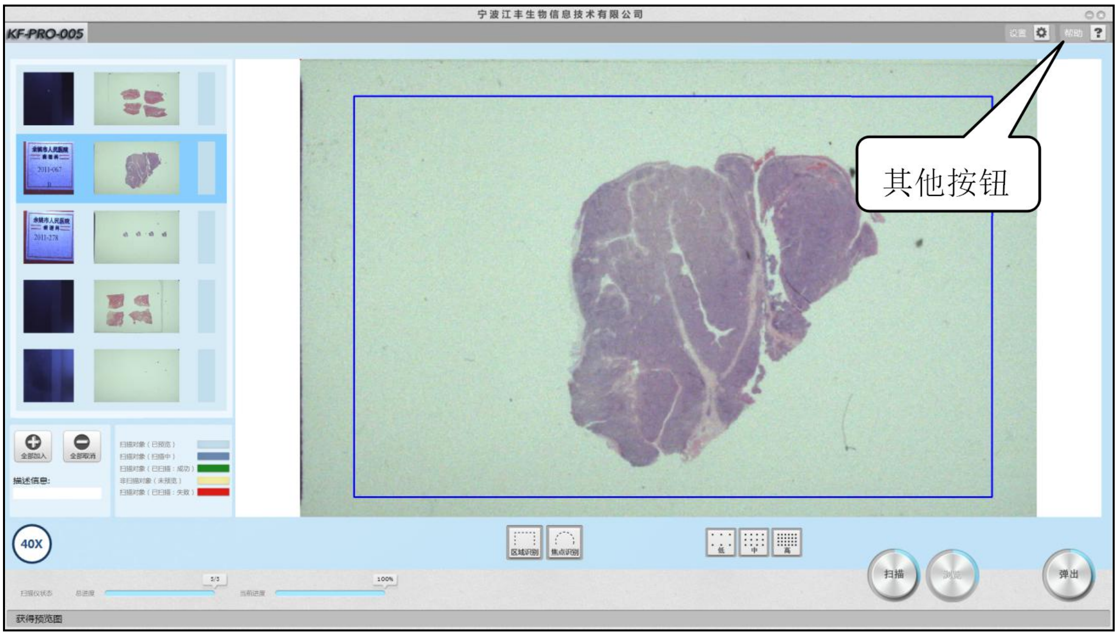

图 9-56 其他按钮

【设置】功能选项中包括了存储路径的选择、扫描方式的选择、切片类型的选择、描述信息的编辑功能以及条码的手动操作。

① 存储路径选择

用户在扫描开始之前可以通过点击如下图所示的【浏览】按钮，弹出如下右图所示的存储路径选择对话框。选中一个路径后，扫描成功的 kfb 文件包 DefaultGroup 将被保存在选中的路径下，如图 9-57 所示。

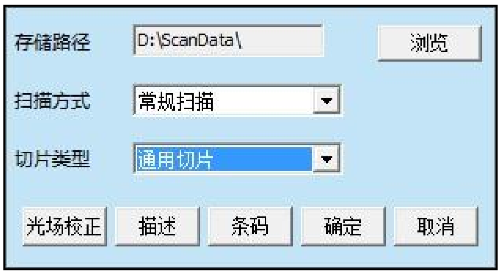

图 9-57 存储路径界面

② 扫描方式选择

扫描方式包括了常规扫描、精细扫描、跳过空白区域扫描三种。

常规扫描：即常规的线扫描方式；

精细扫描：即按照精细调焦的方式对焦后进行线扫描；

跳过空白区域扫描：即对系统识别出的空白区域不扫描，只扫描有组织的区域。

③ 切片类型选择

细胞的切片类型包括组织学、免疫组化两种。

用户在扫描切片的时候根据切片的类型进行设置。

④ 光场校正

用户可以通过该功能实现手动调节 Gain。

⑤ 描述信息

用户点击【描述】按钮后会弹出如下图所示的 5 片切片描述信息的列表，用户在每个编辑框可输入对应的切片信息，如“Test1”是表示对第一张切片的描述信息，扫描结束后，第一张切片的名称将以“Test1”开头，如图 9-58 所示。

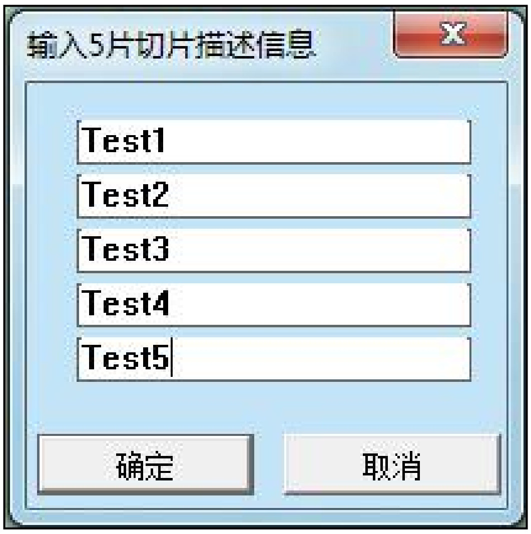

图 9-58 描述信息界面

⑥ 条码

用户点击【条码】按钮后弹出条码的手动输入窗口，如图 9-59 所示。

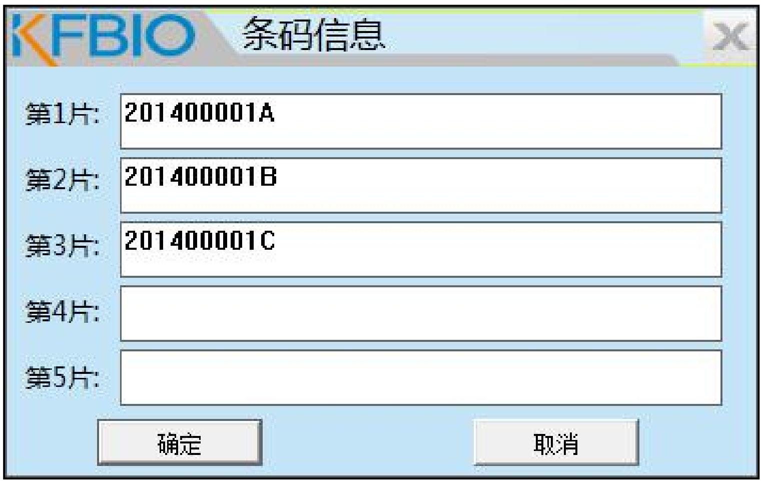

图 9-59 条码界面

用户在每个编辑框可输入对应的条码信息，如“201400001A”是手动输入的第一张切片的条码，扫描第一张切片结束后，切片名称中将会包含输入的条码信息。

注：关于条码，用户可以通过配置参数来设置是否自动识别条码

设置为自动识别条码后，开启软件，当存在系统不能自动识别条码的切片标签时会弹出如下图所示的提示框，如图 9-60 所示。

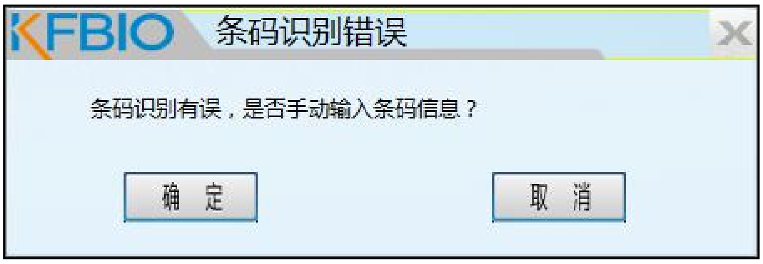

图 9-60 条码提示框

点击【确定】按钮后，弹出手动输入条码信息的窗口；点击【取消】，窗口关闭。

未设置自动识别条码，开启软件后，用户可通过界面右上角的【设置】窗口来完成手动输入条码信息。

以上输入的条码信息会显示生成的 kfb 文件名中。

【关闭】、【最小化】按钮：这两个按钮是对软件窗口的控制，即关闭和最小化窗口的操作。

【帮助】：提供本软件的帮助文档和软件版本。单击【帮助】后，会弹出软件的帮助文档，单击【关于】会弹出软件的版本信息。

【标题栏区域】包括界面中间顶部的公司名称和左上角处的产品系列名称。

9.5.10 如何扫描

在掌握如何扫描之前用户先要了解扫描的具体流程是怎样进行：

首先要加入扫描对象，最后用户可以对当前选择的扫描对象进行预览扫描操作。以上的步骤可以简化为三步：加入→扫描。那么这两步具体是怎么操作的呢？请看下面的内容：

① 如何加入切片

切片的加入可以分为鼠标双击加入单个扫描对象、全部加入所有对象。

鼠标双击加入扫描对象即用户鼠标左键双击缩略图列表区的某一张切片，双击后，对应的切片将被红框框起，即为加入的扫描对象状态。

全部加入即用户点击基础功能按钮区的【全部加入】按钮，所有的切片缩略图被红框框起，表示全部加入到扫描对象。

② 取消扫描对象

在完成了上述的选中之后，用户可以通过以下两种方式来完成取消扫描对象：

- ✓ 点击选择对象功能区域中的【全部取消】按钮来完成取消所有的扫描对象。
- ✓ 另一种取消扫描对象的方式是用户可以通过鼠标双击扫描对象。

③ 如何扫描

在加入扫描对象后，用户就可以进行相关的扫描操作。扫描又可以分为一键扫描和分步扫描两种。在本系列的产品中一键扫描和分步扫描使用的是同一个扫描按钮。

④ 一键扫描

一键扫描是指用户在加入扫描对象后，直接点击主界面右下角处的【扫描】按钮完成的操作。一键扫描适用于对多张切片的连续扫描，用户只需在加入扫描对象后直接点击【扫描】按钮，直到最后一张切片扫描结束为止。

在执行一键扫描的过程中，主界面右下角的【扫描】按钮切换成【停止】按钮。此时用户如果要停止扫描操作，则可以点击【停止】按钮。停止后，机器运转几秒后，界面的按钮恢复到可用状态。

一键扫描的缺陷是在扫描过程中，如果遇到一些切片不干净、制作不合格，自动扫描过程中会出现区域识别不准确，白平衡点有误或者焦点识别不准等现象，这样扫描得到的图像质量一般比较差，出现模糊的情况，大部分不能满足病理医

生的要求，并影响医生的诊断。这时就需要进行人为的扫描操作，即下面将要讲到的分步扫描。

⑤ 分步扫描

分步扫描是指用户通过手动控制扫描的主要流程进行扫描。分步扫描涉及到的主要操作步骤有：区域识别、焦点识别（焦点密度分为低、中、高三种供选择）。

自动预览结束后，用户在左侧的缩略图列表中选择要进行分步扫描的切片，用户只需在缩略图列表区点击该切片对应的缩略图即可在预览区域内显示该切片的预览图。当预览区内显示有预览图后，用户就可以进行分步扫描操作。分步扫描的基本步骤大致可以分为：区域识别→焦点识→扫描。

⑥ 查看切片预览图

自动预览结束后，如果用户想要查看放大的预览图，可以在界面的左侧列表显示所有预览过的切片缩略图，点击某缩略图，右侧的预览区域将会显示对应的预览放大图。

打开某张预览图后，用户就可以进行分步扫描的基本操作，比如：改变扫描区域、焦点识别、扫描等操作。

✓ 第一步：区域识别

预览结束后，用户可以点击【区域识别】按钮，系统会自动执行区域识别操作。系统自动识别的区域是整个切片组织。当用户只需要扫描一小块组织的时候，有两种方式可以改变扫描区域的大小。一种是在自动识别区域结束后，点击鼠标右键弹出一个菜单栏，选择菜单栏中的【改变扫描区域】选项，此时用户按住鼠标左键在预览图上拖动，就可以选择要扫描的区域大小了。另一种是在自动识别区域结束后，拖动红框的四个边，就可以进行扫描区域的改变。

注意：在区域识别的操作中，界面会出现红框和蓝框两种矩形框。红框在蓝框内，不能超出蓝框的范围。红框内的区域是实际要扫描的区域，蓝框是对红框的限制。蓝框的大小也可以通过鼠标拖动来改变。

✓ 第二步：焦点识别

进行过区域识别后的组织就可以进行焦点识别操作，用户点击分步扫描功能区的【焦点识别】按钮后，被框选的区域内显示系统自动识别后的焦点分布状态。用户可以根据对图像质量的需求来选择【低】、【中】、【高】密度（密度越高，扫描得到的图像质量越好，但是密度越高对焦时间会越长，用户可以根据需求来选择焦点密度的高低，系统默认的焦点密度是低密度。高密度适用于组织比较大，扫描难度比较大的切片，低密度适合组织区域比较小，易扫的切片）。

在选择一种模式的密度后，用户可以通过选中某个焦点点击鼠标右键弹出的功能菜单项来进行增加或删除操作。当焦点变黄色十字时代表被选中状态，如图9-61所示。

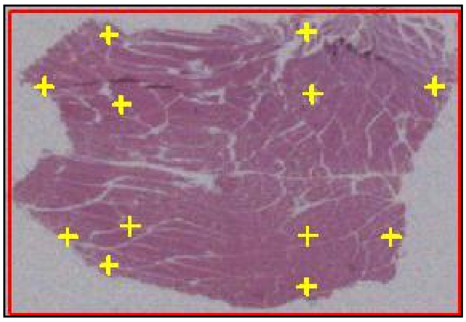

图 9-61 焦点识别界面

然后鼠标右键【删除】该焦点（一般是删除对到空白处或者边缘的焦点）。用户也可以通过鼠标右键直接【增加】焦点，对于增加的焦点或者原本存在的焦点，用户可以通过选中焦点并按住鼠标左键来对其进行拖动操作。

注：【快速删除焦点】——选择右键弹出的【删除焦点】按钮后，键盘按住【Shift】键，鼠标点击某个焦点，即可删除该焦点；【快速增加焦点】——选择右键弹出的【增加焦点】按钮后，键盘按住【Ctrl】键，鼠标点击一下，即可对应的增加一个焦点。

- ✓ 第三步：分步扫描（线扫描操作）

在上面的四步操作完成以后，用户就可以点击预览界面右下角处的【扫描】按钮，下面呈现在用户面前的就是线扫描的动态变化过程，如图 9-62 所示。

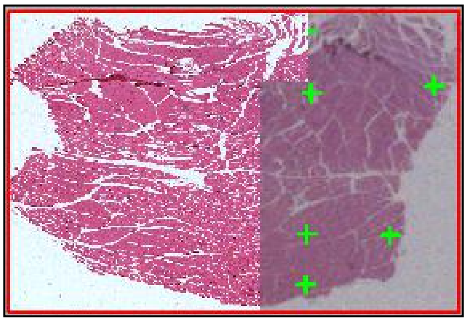

图 9-62 线扫描动态变化过程

说明：以上的操作并不一定要按顺序依次点击完成，用户可以在执行完【区域识别】的操作后，直接点击分步【扫描】按钮，中间的焦点识别、对焦、白平衡相关操作系统会自动执行。同理，直接点击【焦点识别】按钮后，系统会自动执行区域识别操作。

分步扫描结束后，用户通过【视图】按钮将界面切换到第一个界面，可以看到该切片的状态显示为深蓝色，并且该切片还是扫描对象，表示处于扫描中状态，用户在第二个界面还可以对该切片进行重新分步扫描的操作。

在上面的扫描结束后，预览界面的【浏览】按钮处于可用状态，用户点击【浏览】按钮便可以对当前预览区内扫描完的切片进行浏览操作。

9.5.11 图像浏览

扫描结束后，用户可以通过两种方式对图片进行浏览操作：应用程序浏览和网络浏览器浏览。

应用程序浏览又可以有两种途径：

一种是在扫描控制软件的预览界面的分部扫描按钮区域的【浏览】按钮直接查看当前预览区内的扫描图像。（注：如果该预览区内的切片没有扫描则【浏览】按钮处于禁用状态，不能点击），如图 9-63 所示。

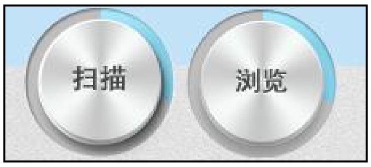

图 9-63 图像浏览按钮

另一种途径就是用户可以在扫描图像的存储文件下，即 D:\ScanData\DefaultGroup 目录下，找到相应的扫描图片，双击该文件即可查看。

说明：以上两种应用程序的查看必须是在本机已安装了应用程序阅片软件的情况下才可以查看。
